# Supplementary material for: Paternal body mass index and offspring DNA methylation: findings from the PACE consortium
Source: Int J Epidemiol. 2021 Jan 29;50(4):1297–315. doi: 10.1093/ije/dyaa267 (PMC8407864; doi:10.1093/ije/dyaa267)
Supplement: dyaa267_Supplementary_Data [file dyaa267_supplementary_data.zip › ije-2020-05-0817-File007.docx]

Table of Contents

[Cohort-specific methods 1](#_Toc34746913)

[ALSPAC 1](#_Toc34746914)

[BiB 3](#_Toc34746915)

[CHAMACOS 4](#_Toc34746916)

[Generation R 6](#_Toc34746917)

[GOYA 7](#_Toc34746918)

[HELIX 8](#_Toc34746919)

[INMA 10](#_Toc34746920)

[MoBa 12](#_Toc34746921)

[Piccolipiù 14](#_Toc34746922)

[Project Viva 15](#_Toc34746923)

[RHEA 16](#_Toc34746924)

[Cohort-specific acknowledgments 18](#_Toc34746925)

[Cohort-specific funding statements 19](#_Toc34746926)

# Cohort-specific methods

## ALSPAC

**Description of cohort**

The Avon Longitudinal Study of Parents and Children (ALSPAC; <http://www.bristol.ac.uk/alspac/> ) is a prospective pregnancy cohort study, which enrolled 14 541 pregnant women residing in Avon, United Kingdom who had expected delivery dates between April 1st, 1991 and December 31st, 1992. As described previously (Boyd et al 2013; Fraser et al, 2013) detailed information has been collected on these participants and their offspring at regular intervals. Please note that the study website contains details of all the data that is available through a fully searchable data dictionary and variable search tool" and reference the following webpage: <http://www.bristol.ac.uk/alspac/researchers/our-data/>.

As part of the Accessible Resources for Integrated Epigenomic Studies (ARIES, <http://www.ariesepigenomics.org.uk/)> project, DNA methylation was generated for 1018 mother-offspring pairs from the ALSPAC cohort, using the Infinium HumanMethylation450 BeadChip array (Illumina Inc., San Diego, United States). ARIES participants were selected based on availability of DNA samples at two time points for the mother (antenatal and at follow-up when the offspring were adolescents) and at three time points for the offspring (neonatal, childhood (age 7), and adolescence (age 17)). The current study used child cord blood at birth and whole blood at age 7.

Ethical approval for the study was obtained from the ALSPAC Ethics and Law Committee and the Local Research Ethics Committees. Consent for biological samples has been collected in accordance with the Human Tissue Act (2004). Informed consent for the use of data collected via questionnaires and clinics was obtained from the participants following the recommendations of the ALSPAC Ethics and Law Committee at the time.

**Definition of phenotype and covariates**

Maternal and paternal age at delivery was derived from the mother’s date of birth. Maternal and paternal body mass index (BMI) was calculated from self-reported height and pre-pregnancy weight, which were collected by questionnaire during the first trimester of pregnancy. Paternal education was determined by a questionnaire to the mother, completed during pregnancy, and was collapsed into the following two categories: less than A level or A level and above. Paternal smoking was defined as any smoking in the three months prior to pregnancy and/or during the first trimester of pregnancy, vs no smoking during this period. It was collected via questionnaire to the father sent during the first trimester. Maternal smoking during pregnancy was defined as smoking at least one cigarette per day beyond the first trimester vs no smoking or smoking in the first trimester only, and was determined by questionnaire at the time of recruitment. Newborn sex was obtained from obstetric records. Participants with non-white European ancestry were excluded from all analyses. Estimation of blood cell type proportions was obtained from methylation data applying the Houseman method (Houseman et al., 2012) and the Bakulski’s (Bakulski et al., 2016) and Reinius’ (Reinius, 2012) reference panels for cord blood and child blood, respectively.

**Generation of methylation data and pre-processing methods**

Methods for methylation measurements in ALSPAC have been described previously (Relton et al., 2015). Briefly, cord blood was collected according to standard procedures. DNA methylation assays and data pre-processing was performed at the University of Bristol as part of the ARIES project. DNA was extracted using standard protocol and was bisulfite-converted using the Zymo EZ DNA MethylationTM kit (Zymo, Irvine, CA). DNA methylation was then measured using the Infinium HM450 BeadChip assay (Illumina Inc, San Diego, CA), according to the standard protocol. Arrays were scanned using an Illumina iScan. An initial review of data quality was assessed using GenomeStudio (version 2011.1). A semi-random approach (sampling criteria were in place to ensure that all time points were represented on each array) was used to distribute ARIES samples across slides to minimize the possibility of potential confounding by batch. Data were normalised using the meffil R package (Min et al, 2018).

**References**

Bakulski et al., 2016, DNA methylation of cord blood cell types: applications for mixed cell birth studies. Epigenetics. 11(5):354-62.

Boyd A, Golding J, Macleod J, Lawlor DA, Fraser A, Henderson J, et al. Cohort Profile: the 'children of the 90s'--the index offspring of the Avon Longitudinal Study of Parents and Children. Int J Epidemiol. 2013;42(1):111-27.

Fraser A, Macdonald-Wallis C, Tilling K, Boyd A, Golding J, Davey Smith G, et al. Cohort Profile: the Avon Longitudinal Study of Parents and Children: ALSPAC mothers cohort. Int J Epidemiol. 2013;42(1):97-110.

Houseman EA, Accomando WP, Koestler DC, Christensen BC, Marsit CJ, Nelson HH, et al. DNA methylation arrays as surrogate measures of cell mixture distribution. BMC Bioinformatics. 2012;13:86

Reinius LE, Acevedo N, Joerink M, Pershagen G, Dahlén SE, Greco D, Söderhäll C, Scheynius A, Kere J.[Differential DNA methylation in purified human blood cells: implications for cell lineage and studies on disease susceptibility.](http://www.ncbi.nlm.nih.gov/pubmed/22848472)PLoS One. 2012;7(7):e41361. doi: 10.1371/journal.pone.0041361. Epub 2012 Jul 25.

Relton CL, Gaunt T, McArdle W, Ho K, Duggirala A, Shihab H, et al. Data Resource Profile: Accessible Resource for Integrated Epigenomic Studies (ARIES). Int J Epidemiol. 2015;44(4):1181-90.

Min J, Hemani G, Davey Smith G, Relton C, Suderman M. Meffil: efficient normalization and analysis of very large DNA methylation datasets. Bioinformatics. 2018 1:34(23):3983-3989.

## BiB

**Description of cohort**

Born in Bradford (BiB; <https://borninbradford.nhs.uk> ) is a longitudinal multi-ethnic birth cohort study aiming to examine the impact of environmental, psychological and genetic factors on maternal and child health and wellbeing (Wright et al., 2013). Bradford is a city in the North of England with high levels of socio-economic deprivation and ethnic diversity. Women were recruited at the Bradford Royal Infirmary at 24-29 weeks gestation. For those consenting, a baseline questionnaire was completed. The full BiB cohort recruited 12,450 women comprising 13,773 pregnancies and 13,858 children between 2007 and 2010. Results of an oral glucose tolerance test (OGTT) and lipid profiles were obtained on the mothers during pregnancy at recruitment (24-29 weeks gestation), and pregnancy serum, plasma and urine samples have been stored. Cord blood samples have been obtained and stored and DNA extraction has been completed on all cord and pregnancy samples. The cohort is broadly characteristic of the city's maternal population. Mean age of the mothers at study recruitment was 27 years old. Researchers are looking at the links between the circumstances of a child's birth, the context in which they grow up, their health and well-being and their educational progress.

For DNAm assays, 1000 mother-child pairs were selected (2000 individuals) from the participants who had completed a pregnancy OGTT (85% of BiB participants completed an OGTT), genome wide data on both mother and offspring (at the time of selection ~65% of those with OGTT) and were of either Pakistani or white British ethnic origin (the two largest homogeneous ethnic groups jointing reflecting 90% of the cohort). Within these criteria 500 Pakistani and 500 white British mother-child pairs were randomly selected. Ethical approval for the data collection was granted by Bradford Research Ethics Committee (Ref 07/H1302/112). On registration with the study, pregnant mothers gave written informed consent for themselves and on behalf of their child.

**Definition of phenotype and covariates**

Maternal and paternal age at delivery was derived from the mother’s/father’s date of birth. Paternal body mass index (BMI) was calculated from self-reported height and weight, which were collected by questionnaire during the first trimester of pregnancy. Maternal BMI was calculated from height measured at recruitment (around 26 weeks gestation) and weight measured at booking (around 12 weeks gestation). Paternal education was determined by a questionnaire to the mother, completed during pregnancy, and was collapsed into the following two categories: less than A level or A level and above. Paternal smoking was defined as any smoking vs no smoking. It was collected via questionnaire to the father sent during the first trimester. Maternal smoking during pregnancy was defined as smoking after the fourth month vs not smoking or smoking only before the fourth month, and was determined by questionnaire at the time of recruitment. Newborn sex was obtained from obstetric records. Analyses were stratified by ethnicity: Pakistani and white British origins. Estimation of blood cell type proportions was obtained from methylation data applying the Houseman method (Houseman et al., 2012) with the Bakulski reference.

**Generation of methylation data and pre-processing methods**

A total of 500 ng high molecular weight DNA was bisulfite-converted using the EZ-96 DNA methylation kit (Zymo Research, Orange, CA, USA). DNAm was quantified using Illumina HumanMethylation EPIC Arrays (Illumina, San Diego, CA, USA). During the data generation process, a wide range of batch variables were recorded in a purpose-built laboratory information management system (LIMS). Sample QC and normalization was performed using with meffil9. Samples failing QC (average probe p > 0.01) were excluded from further analysis. As an additional QC step genotype probes were compared with genotype data from the same individual to identify and remove any sample mismatches. Furthermore, samples failed on control probes (bisulfite 1 and bisulfite 2) were also excluded from the analysis. Finally, 864 samples passed QC. Samples were normalized using functional normalization using meffil (Min et al., 2018). Data was normalised using 7 control probe PCs derived from the technical probes informed by meffil scree plots.

**References**

Bakulski et al., 2016, DNA methylation of cord blood cell types: applications for mixed cell birth studies. Epigenetics. 11(5):354-62.

Houseman EA, Accomando WP, Koestler DC, Christensen BC, Marsit CJ, Nelson HH, et al. DNA methylation arrays as surrogate measures of cell mixture distribution. BMC Bioinformatics. 2012;13:86

Reinius LE, Acevedo N, Joerink M, Pershagen G, Dahlén SE, Greco D, Söderhäll C, Scheynius A, Kere J.[Differential DNA methylation in purified human blood cells: implications for cell lineage and studies on disease susceptibility.](http://www.ncbi.nlm.nih.gov/pubmed/22848472)PLoS One. 2012;7(7):e41361. doi: 10.1371/journal.pone.0041361. Epub 2012 Jul 25.

Min J, Hemani G, Davey Smith G, Relton C, Suderman M. Meffil: efficient normalization and analysis of very large DNA methylation datasets. Bioinformatics. 2018 1:34(23):3983-3989.

Wright J, Small N, Raynor P, Tuffnell D, Bhopal R, Cameron N, et al. Cohort Profile: The Born in Bradford multi-ethnic family cohort study. Int J Epidemiol. 2013 Aug 1;42(4):978–91.

## CHAMACOS

**Description of cohort**

The Center for the Health Assessment of Mothers and Children of Salinas (CHAMACOS; <https://cerch.berkeley.edu/research-programs/chamacos-study> ) study is a longitudinal birth cohort study examining the relationship of pesticide and environmental chemical exposure with development and health of Mexican-American children from Salinas Valley, CA. Additional details on the CHAMACOS cohort have previously been published1, 2. From 1999-2000, 601 pregnant women were enrolled at community clinics and 527 liveborn singletons were born. In addition to pre-pregnancy and delivery assessments, follow up visits occurred at regular intervals throughout childhood. Study protocols were approved by the University of California, Berkeley Committee for Protection of Human Subjects and written informed consent was obtained from all mothers; oral assent was obtained from children beginning at age 7, and written assent at age 12.

**Definition of phenotype and covariates**

Paternal BMI was calculated from measured height and weight around delivery. At this assessment, information on paternal age and smoking status was obtained from fathers. Paternal age was analysed as a continuous variable. Paternal smoking was dichotomized. The two groups included fathers with no smoking in the six months prior to the pregnancy compared to fathers who smoked six months before the pregnancy. Paternal education level was used as a proxy for paternal social class. Paternal education was treated as categorical, with two levels: less than a completed high school education, or having completed high school education or beyond.

Maternal BMI was assessed in early pregnancy using measured height and self-reported pre-pregnancy weight. Height without shoes was measured using a stadiometer. Maternal age and parity were assessed by participant interview at the baseline visit (~13 weeks gestation). Maternal age was treated as a continuous variable. Parity was coded as a binary variable, with 0 as the baseline and ≥1 as the alternative. None of the mothers included in the analysis reported smoking during pregnancy.

We used the Houseman algorithm3 with either a cord blood4 or peripheral blood5 reference panel to estimate relative proportions of six blood cell subtypes (CD4+ T-lymphocytes, CD8+ T-lymphocytes, NK (natural killer) cells, B-lymphocytes, monocytes, granulocytes). Nucleated red blood cells were also estimated using the cord blood reference panel.

**Generation of methylation data and pre-processing methods**

DNA methylation was measured in DNA isolated from the cord blood of CHAMACOS newborns and from blood samples collected from nine year olds by Illumina Infinium HumanMethylation450 (450K) BeadChips. DNA samples were bisulfite converted using Zymo Bisulfite Conversion Kits (Zymo Research, Irvine, CA), whole genome amplified, enzymatically fragmented, purified, and applied to the 450K BeadChips (Illumina, San Diego, CA) according to manufacturer protocol. 450K BeadChips were handled by robotics and analysed using the Illumina Hi-Scan system. DNA methylation was measured at 485,512 CpG sites.

Probe signal intensities were extracted by Illumina GenomeStudio software (version XXV2011.1, Methylation Module 1.9) methylation module and background subtracted. QA/QC procedure were previously described6 and included assessment of assay repeatability batch effects using 38 technical replicates. Additionally, quality was ensured by only retaining samples where 95 % of sites assayed had detection p> 0.01. The same threshold (95% detection at p>0.01) was imposed to CpGs as well (n= 460 removed). We excluded sites with annotated probe SNPs and with common SNPs (minor allele frequency >5%) within 50bp of the target identified in the MXL (Mexican ancestry in Los Angeles, California) HapMap population (n=49,748). A total of 435,369 CpGs remained in the analysis. Minimization of color channel bias, batch effects and difference in Infinium chemistry was achieved by application of ASMN algorithm6, followed by BMIQ normalization7. The final dataset contained information on 435,369 CpGs for 158 cord blood and 108 9-year old blood samples.

**References**

1. Eskenazi, B. et al. CHAMACOS, A Longitudinal Birth Cohort Study: Lessons from the Fields. Journal of Children's Health 1, 3-27 (2003).

2. Eskenazi, B. et al. Association of in utero organophosphate pesticide exposure and fetal growth and length of gestation in an agricultural population. Environ. Health Perspect. 112, 1116-1124 (2004).

3. Houseman, E. A. et al. DNA methylation arrays as surrogate measures of cell mixture distribution. BMC Bioinformatics 13, 86 (2012).

4. Bakulski, K. M. et al. DNA methylation of cord blood cell types: Applications for mixed cell birth studies. Epigenetics 11, 354-362 (2016).

5. Reinius, L. E. et al. Differential DNA methylation in purified human blood cells: implications for cell lineage and studies on disease susceptibility. PLoS One 7, e41361 (2012).

6. Yousefi, P. et al. Considerations for normalization of DNA methylation data by Illumina 450K BeadChip assay in population studies. Epigenetics 8, 1141-1152 (2013).

7. Teschendorff, A. E. et al. A beta-mixture quantile normalization method for correcting probe design bias in Illumina Infinium 450 k DNA methylation data. Bioinformatics 29, 189-196 (2013).

## Generation R

**Description of cohort**

The Generation R Study (<https://generationr.nl/researchers/>) is a population-based prospective cohort study from fetal life onwards in Rotterdam, the Netherlands. A detailed description can be found elsewhere [Kooijman et al, Eur J Epidemiol 2016; Kruithof et al, Eur J Epidemiol 2014]. Briefly, 9,778 pregnant women with a delivery date between April 2002 and January 2006 were included (response rate at baseline 61%). There is ongoing follow-up. The study was approved by Medical Ethical Committee of Erasmus MC, University Medical Center Rotterdam and written consent was obtained for all participants. For this analysis, we used singleton live births with epigenome-wide association arrays and information on paternal BMI and complete covariates, giving total sample sizes of 947 at birth and 335 in childhood.

**Definition of covariates**

Information on maternal and paternal age, maternal education, parity, pre-pregnancy maternal BMI and maternal and paternal smoking was collected by questionnaire at enrollment. Child sex was collected from birth records. Maternal age and BMI were used as continuous covariates. Maternal education was categorized into lower (none, primary or secondary education) and higher (more than secondary education). Maternal active smoking was classified into two categories: no smoking or quitting early in pregnancy versus sustained smoking during pregnancy. Parity was classified into 0 versus 1 or more. Paternal smoking around conception was classified as yes or no.

**Generation of methylation data and pre-processing methods**

DNA, 500 ng per sample, extracted (using the salting-out method) from blood samples taken at birth (cord blood) or at the 5-year follow-up underwent bisulfite conversion using the EZ-96 DNA Methylation kit (Shallow) (Zymo Research Corporation, Irvine, USA). Samples were plated onto 96-well plates in no specific order. Samples were processed with the Illumina Infinium HumanMethylation450 BeadChip (Illumina Inc., San Diego, USA), which analyses methylation at 485,577 CpG sites. Preparation and normalization of the HumanMethylation450 BeadChip array data was performed according to the CPACOR workflow [Lehne et al. 2015] using the package minfi in R [R Core Team, 2013]. Probes that had a detection p-value above background (based on sum of methylated and unmethylated intensity values) ≥ 1E-16 were set to missing per array. Next, the intensity values were stratified by autosomal and non-autosomal probes and quantile normalized for each of the six probe type categories separately: type II red/green, type I methylated red/green and type I unmethylated red/green. Beta values were calculated as proportion of methylated intensity value on the sum of methylated+unmethylated+100 intensities. Arrays with observed technical problems such as failed bisulfite conversion, hybridization or extension, as well as arrays with a mismatch between sex of the proband and sex determined by the chr X and Y probe intensities were removed from subsequent analyses. Additionally, only arrays with a call rate > 95% per sample were processed further.

**References**

Kooijman MN, Kruithof CJ, van Duijn CM, Duijts L, Franco OH, van IJzendoorn MH, de Jongste JC, Klaver CC, van der Lugt A, Mackenbach JP, Moll HA, Peeters RP, Raat H, Rings EH, Rivadeneira F, van der Schroeff MP, Steegers EA, Tiemeier H, Uitterlinden AG, Verhulst FC, Wolvius E, Felix JF, Jaddoe VW. The Generation R Study: design and cohort update 2017. Eur J Epidemiol. 2016 Dec;31(12):1243-1264. doi: 10.1007/s10654-016-0224-9. Epub 2017 Jan 9. PubMed PMID: 28070760; PubMed Central PMCID: PMC5233749.

Kruithof CJ, Kooijman MN, van Duijn CM, Franco OH, de Jongste JC, Klaver CC, Mackenbach JP, Moll HA, Raat H, Rings EH, Rivadeneira F, Steegers EA, Tiemeier H, Uitterlinden AG, Verhulst FC, Wolvius EB, Hofman A, Jaddoe VW. The Generation R Study: Biobank update 2015. Eur J Epidemiol. 2014 Dec;29(12):911-27. doi: 10.1007/s10654-014-9980-6. Epub 2014 Dec 21. PubMed PMID: 25527369.

Lehne B, Drong AW, Loh M, Zhang W, Scott WR, Tan ST, Afzal U, Scott J, Jarvelin MR, Elliott P, McCarthy MI, Kooner JS, Chambers JC. A coherent approach for analysis of the Illumina HumanMethylation450 BeadChip improves data quality and performance in epigenome-wide association studies. Genome Biol 2015;16:37.

R Core Team (2013). R: A language and environment for statistical computing. R Foundation for Statistical Computing, Vienna, Austria. ISBN 3-900051-07-0, URL <http://www.R-project.org/>.

## GOYA

**Description of cohort**

The Genetics of Overweight Young Adults (GOYA) study has been described previously (Paternoster et al. 2011). Briefly, it includes a case-cohort sampled subset of 91,387 pregnant women recruited into the Danish National Birth Cohort (DNBC; <https://www.dnbc.dk> ) between 1996 and 2002. GOYA cases were selected as the 3.6% of DNBC mothers with the largest residuals from the regression of BMI on age and parity (all entered as continuous variables) from the total of 67,853 eligible women who had given birth to a live infant, provided a blood sample during pregnancy, and had BMI information available. BMI for these 2,451 women “cases” ranged from 32.6 to 64.4. A similar number of women (n=2,450) were sampled from the remaining cohort as controls. DNA methylation data were generated for the offspring of 1,000 case and control mothers in the GOYA study. Current analysis among participants with DNA methylation available was restricted to a randomly selected sub-group with a normal BMI distribution to avoid confounding by substructure. Analysis was further restricted to the N = 390 mother-child case pairs with all relevant covariates available.

**Definition of covariates**

Data on paternal and maternal covariates were collected via a telephone interview at around 16 weeks gestation. Maternal pre-pregnancy BMI was calculated from self-reported height and weight. Paternal BMI was reported by the mother 18 months after pregnancy. Active smoking in pregnancy was defined as any/no reported smoking in pregnancy. Age was derived from self-reported date of birth. Socioeconomic status was defined using maternal education or occupation: 1) manager/long or medium education, 2) work requiring a short training period, or skilled manual labour, 3) unskilled or public service. Parity was categorized for this study as nulliparous or parous. Ten surrogate variables were generated and included in models to adjust for technical batch. Estimation of seven different cord white blood cell types (CD8+ T and CD4+ T lymphocytes, CD56+ natural killer cells, CD19+ B cells, CD14+ monocytes, granulocytes, nRBCs) were computed by the Houseman method (Houseman et al. 2012) using the cord blood reference dataset (Bakulski et al.) and the default implementation of the estimateCellCounts function in the *minfi* package (Aryee et al. 2014).

**Generation of methylation data and pre-processing methods**

Cord blood was collected according to standard procedures, spun and frozen at -80˚C. DNA methylation analysis and data pre-processing were performed at the University of Bristol. Following extraction, DNA was bisulfite converted using the Zymo EZ DNA MethylationTM kit (Zymo, Irvine, CA). Following conversion, methylation status was measured using the Illumina Infinium® HumanMethylation450k BeadChip assay according to standard protocol. The arrays were scanned using an Illumina iScan and initial quality review was assessed using GenomeStudio (version 2011.1).

All DNA methylation results were cleaned and normalized in R (version 3.3.0) using the *meffil* package (Min et al. 2018). Samples were excluded from downstream analysis if > 10% of probes had a p-value ≥ 0.01, and CpGs were excluded if their probe detection p-values were ≥ 0.01 across > 10% of samples. Sample sex was assessed by comparing HumanMethylation450k genotype probes to previous SNP-chip data and comparing median chromosome Y and chromosome X probe intensities. Samples were excluded if they failed these checks. Data were normalized using the functional normalization approach in the *minfi* R package (Aryee et al. 2014). Probes with values > 3*interquartile range were also excluded from analyses. Level of methylation measurements were expressed as β-values ranging from 0 (no cytosine methylation) to 1 (complete cytosine methylation).

**References**

Aryee, Martin J, Andrew E Jaffe, Hector Corrada-Bravo, Christine Ladd-Acosta, Andrew P Feinberg, Kasper D Hansen, and Rafael A Irizarry. 2014. “Minfi: A Flexible and Comprehensive Bioconductor Package for the Analysis of Infinium DNA Methylation Microarrays.” *Bioinformatics* 30 (10): 1363–69. https://doi.org/10.1093/bioinformatics/btu049.

Houseman, E A, W P Accomando, D C Koestler, B C Christensen, C J Marsit, H H Nelson, J K Wiencke, and K T Kelsey. 2012. “DNA Methylation Arrays as Surrogate Measures of Cell Mixture Distribution.” *BMC Bioinformatics* 13 (May). https://doi.org/10.1186/1471-2105-13-86.

Min, J L, G Hemani, G Davey Smith, C Relton, M Suderman, and John Hancock. 2018. “Meffil: Efficient Normalization and Analysis of Very Large DNA Methylation Datasets.” Edited by John Hancock. *Bioinformatics*, June. https://doi.org/10.1093/bioinformatics/bty476.

Paternoster, Lavinia, David M Evans, Ellen Aagaard Nohr, Claus Holst, Valerie Gaborieau, Paul Brennan, Anette Prior Gjesing, et al. 2011. “Genome-Wide Population-Based Association Study of Extremely Overweight Young Adults--the GOYA Study.” Edited by Philip Awadalla. *PLoS One* 6 (9): e24303. http://dx.plos.org/10.1371/journal.pone.0024303.

## HELIX

**Description of cohort**

The Human Early Life Exposome (HELIX; <https://www.projecthelix.eu> ) study (Maitre et al. 2018) is a collaborative project across 6 established and ongoing longitudinal population-based cohort studies in Europe: the Born in Bradford (BiB) study in the UK, the Étude des Déterminants pré et postnatals du développement et de la santé de l’Enfant (EDEN) study in France, the INfancia y Medio Ambiente (INMA, Sabadell subcohort) project in Spain, the Kaunus cohort (KANC) in Lithuania, the Norwegian Mother, Father and Child Cohort Study (MoBa), and the Rhea Mother Child Cohort study in Crete, Greece. The aim of HELIX is to investigate the effect of the exposome on health and the underlying molecular mechanisms. For this, HELIX measured a wide range environmental exposures in early-life, molecular phenotypes and health outcomes in children. All cohorts, except for KANC, which did not have data on paternal BMI, participated in the current analysis. Non-European ancestry children, according to diverse questionnaire information, were filtered out.

**Definition of phenotypes**

In BiB, mothers were weighed around 12-13 weeks of gestation and height was obtained around 26-28 weeks of gestation. Paternal weight and height was self-reported in week 26-28.

In EDEN, maternal weight and height before pregnancy was self-reported and collected around week 24-26 of pregnancy. Paternal weight and height was self-reported during pregnancy.

In Rhea, we calculated maternal pre-pregnancy BMI (kg/m2) based on maternal height measured at the first prenatal visit [mean (SD): 12.4 (1.6) weeks] and pre-pregnancy weight reported by the mother. Paternal BMI was based on paternal weight and height reported by the mother, since the study protocol did not include contact with the father of the child.

In MoBa, maternal weight and height was self-reported in Questionnaire 1 answered around week 17 of pregnancy. Paternal height was reported in a questionnaire filled in by the father in week 17-18.

For phenotypes and covariate measured in INMA cohort, see below.

**Definition of covariates**

Paternal and maternal age, parity, paternal education level and maternal smoking were obtained from pre-harmonized variables from the European ESCAPE project. Maternal and paternal age were used as a continuous covariate. Parity was categorized into 3 levels, 0, 1 or ≥2. Parental education level was categorized in low (primary or secondary school) and high (university degree or higher).

Maternal smoking was defined as any smoking at any point during pregnancy. Paternal smoking was recorded and defined slightly different in each cohort. In BIB, paternal smoking was reported by the mother in week 26-28, and classified as yes/no. In EDEN, paternal smoking status was collected by self-questionnaire administered during the pregnancy of the mother. Smoking was reported as non-smoker or smokes at least one cigarette per day. In Rhea, paternal smoking was reported by the mother at 12th and 30th week of pregnancy and non-smokers at both time-points were categorized as “no”. In MoBa, paternal smoking was reported in the questionnaire filled in by the father in week 17-18.

Estimation of blood cell type proportions was obtained from methylation data applying the Houseman method (Houseman et al., 2012) and the Bakulski (Bakulski et al., 2016) and Reinius (Reinius, 2012) reference panels for cord blood and child blood, respectively.

**Generation of methylation data and pre-processing methods**

DNA was extracted from children’s buffy coat using the Chemagen kit (Perkin Elmer, USA), cohort by cohort. DNA concentration was determined in a Nanodrop 1000 UV-Vis Spectrophotometer (Thermo Fisher Scientific, USA) and with the Quant-iTTM PicoGreenâ dsDNA Assay Kit (Life Technologies, USA).

DNA methylation was assessed with the Infinium HumanMethylatio450 beadchip (Illumina, USA) at the University of Santiago de Compostela – Spanish National Genotyping Center (CeGen-USC, Spain). 700 ng of DNA were bisulfite-converted using the EZ 96-DNA kit (Zymo Research, USA) following the manufacturer’s standard protocol. All samples of the study were randomized taking into account sex and cohort. In addition, each plate contained a HapMap control sample and a total of 24 HELIX inter-plate duplicates were included.

After an initial inspection of the quality of the methylation data with the MethylAid package (van Iterson et al. 2014), probes with a call rate <95% based on a detection p-value of 1E-16 (Lehne et al. 2015), and samples with a call rate <98% were removed. Then, samples with discordant sex, duplicates with inconsistent genotypes, and samples with inconsistent genotypes respect to existing genome-wide genotyping array data, were eliminated. Methylation data was normalized using the functional normalization method with prior background correction with Noob (Fortin et al. 2014).

**References**

Houseman EA, Accomando WP, Koestler DC, Christensen BC, Marsit CJ, Nelson HH, et al. DNA methylation arrays as surrogate measures of cell mixture distribution. BMC Bioinformatics. 2012;13:86

Maitre L, de Bont J, Casas M, Robinson O, Aasvang GM, Agier L, et al. Human Early Life Exposome (HELIX) study: a European population-based exposome cohort. BMJ Open [Internet]. British Medical Journal Publishing Group; 2018 Sep 10 [cited 2019 Jan 3];8(9):e021311. Available from: http://www.ncbi.nlm.nih.gov/pubmed/30206078

Reinius LE, Acevedo N, Joerink M, Pershagen G, Dahlén SE, Greco D, Söderhäll C, Scheynius A, Kere J.Differential DNA methylation in purified human blood cells: implications for cell lineage and studies on disease susceptibility.PLoS One. 2012;7(7):e41361. doi: 10.1371/journal.pone.0041361. Epub 2012 Jul 25.

## INMA

**Description of cohort**

Population-based birth cohorts were established as part of the INMA – INfancia y Medio Ambiente [Environment and Childhood] Project in several regions of Spain following a common protocol (<http://www.proyectoinma.org/> ). This project aims to study the associations between pre- and postnatal environmental exposures and growth, health, and development from early foetal life until adolescence and has been described previously in detail (Guxens et al., 2012). Pregnant women were enrolled during the 1st trimester of pregnancy at public primary health care centers or public hospitals. Detailed measurements were performed using ultrasound and physical examinations and biological samples were collected. Informed consent was obtained from all participants and the study was approved by the Hospital Ethics Committees in each participating region. Blood DNA methylation data was assessed with the Infinium HumanMethylation450 BeadChip in INMA Sabadell subcohort, at birth, 4y and 9y. In the current study we participated with methylation data assessed at birth (INMA study) and at age 9y (as part of the HELIX study).

**Definition of phenotypes**

Maternal pre-pregnancy BMI was calculated from measured height and self-reported pre-pregnancy weight collected using a questionnaire at enrolment (week 12 of pregnancy) (Casas et al., 2013) Reported pre-pregnancy weight was highly correlated with measured weight at 12 weeks of pregnancy in INMA (r= 0.96; P < 0.0001). Paternal weight and height were reported by the mother at this time and used to calculate paternal BMI.

**Definition of covariates**

Information on parity and maternal and paternal age was collected by questionnaire at enrolment (week 12 of pregnancy). Maternal and paternal age was used as a continuous covariate. Parity was categorized into 0 or ≥1. Paternal socioeconomic status was based on paternal education level assessed by maternal questionnaire at week 12 of pregnancy and classified as low (primary or less and secondary) and high (university). Pregnant women were asked about maternal and paternal smoking at week 32 of pregnancy. Maternal any smoking was defined as smoking any number of cigarettes at any time during pregnancy. Paternal smoking was defined as “no” if they were non-smoker or quit before week 12 of pregnancy.

Estimation of blood cell type proportions was obtained from methylation data applying the Houseman method (Houseman et al., 2012) and the Bakulski’s (Bakulski et al., 2016) and Reinius’ (Reinius, 2012) reference panels for cord blood and child blood, respectively.

**Generation of methylation data and pre-processing methods**

Cord blood and whole blood collected at age 4y was extracted using the Chemagen kit (Perkin Elmer). DNA concentration was determined by a NanoDrop spectrophotometer (Thermo Scientific) and with the Quant-iT PicoGreen dsDNA Assay Kit (Life Technologies).

Blood methylation data was produced in two laboratories: the Genome Analysis Facility of the University Medical Center Groningen (UMCG) in Holland as part of the MeDALL project (0y and 4y), and the Bellvitge Biomedical Research Institute (IDIBELL) in Barcelona as part of the BREATHE project (0y). Both laboratories randomized the samples in batches and followed the Illumina protocol for the Infinium HumanMethylation450 BeadChip. Briefly, 500 ng of DNA was bisulfite-converted using the EZ 96-DNA methylation kit, and DNA methylation was measured through hybridization on the BeadChips. BeadChips were scanned with an Illumina iScan and image data was uploaded into the Methylation Module of Illumina’s analysis software GenomeStudio, and converted in β-values.

Two blood samples with overall low quality (MethylAid package) (van Iterson et al. 2014), and three blood samples discordant for sex (shinyMethyl package)(Fortin et al., 2014) were removed. After applying a stringent detection p-value of 1.10E-16 (Lehne et al. 2015), 18 blood samples with a call rate <98% were excluded. 7,136 probes with a call rate <95%, control probes and probes designed to detect genetic polymorphisms were removed. Data was normalized with the functional normalization method with prior background correction with Noob implemented in the minfi package (Aryee et al. 2014).

**References**

Aryee et al., 2014. Minfi: a flexible and comprehensive Bioconductor package for the analysis of Infinium DNA methylation microarrays. Bioinformatics. 15:30(10).

Bakulski et al., 2016, DNA methylation of cord blood cell types: applications for mixed cell birth studies. Epigenetics. 11(5):354-62.

Casas M, Chatzi L, Carsin A-E, Amiano P, Guxens M, Kogevinas M, et al. Maternal pre-pregnancy overweight and obesity, and child neuropsychological development: two Southern European birth cohort studies. Int. J. Epidemiol. 2013;42:506–17.

Fortin et al., 2014, Functional normalization of 450k methylation array data improves replication in large cancer studies. Genome Biol. 15(12):503.

Guxens, M., Ballester, F., Espada, M., Fernandez, M.F., Grimalt, J.O., Ibarluzea, J., Olea, N., Rebagliato, M., Tardon, A., Torrent, M. et al. (2012) Cohort Profile: the INMA--INfancia y Medio Ambiente--(Environment and Childhood) Project. Int. J. Epidemiol., 41, 930-940.

Houseman EA, Accomando WP, Koestler DC, Christensen BC, Marsit CJ, Nelson HH, et al. DNA methylation arrays as surrogate measures of cell mixture distribution. BMC Bioinformatics. 2012;13:86

Lehne et al., 2015. A coherent approach for analysis of the Illumina HumanMethylation450 BeadChip improves data quality and performance in epigenome-wide association studies. Genome Biology. 15; 16:37

Reinius LE, Acevedo N, Joerink M, Pershagen G, Dahlén SE, Greco D, Söderhäll C, Scheynius A, Kere J.[Differential DNA methylation in purified human blood cells: implications for cell lineage and studies on disease susceptibility.](http://www.ncbi.nlm.nih.gov/pubmed/22848472)PLoS One. 2012;7(7):e41361. doi: 10.1371/journal.pone.0041361.

Van Iterson M, et al. 2014, MethylAid : visual and interactive quality control of large Illumina 450k datasets, Bioinformatics, 30(23):3435-7.

## MoBa

**Design and study population**

Participants represent three subsets of mother-offspring pairs from the national Norwegian Mother, Father and Child Cohort Study (MoBa) (Magnus et al. 2016; <https://www.fhi.no/en/studies/moba/> ). MoBa is a prospective population-based pregnancy cohort study conducted by the Norwegian Institute of Public Health. The years of birth for MoBa participants ranged from 1999-2009. MoBa mothers provided written informed consent. Each subset is referred to here as MoBa1, MoBa2 and MoBa3. MoBa1 is a subset of a larger study within MoBa that included a cohort random sample and cases of asthma at age three years (Haberg et al., 2011). We previously reported an association between maternal smoking during pregnancy and differential DNA methylation in MoBa1 newborns (Joubert et al., 2012). We subsequently measured DNA methylation in additional newborns (MoBa2) in the same laboratory (Illumina, San Diego, CA) (Joubert et al., 2016). MoBa2 included cohort random samples plus cases of asthma at age seven years and non-asthmatic controls. MoBa3 was designed to evaluate the association between differential cord blood DNA methylation and later childhood cancer status. Methylation measurements for MoBa3 were made at IARC. Years of birth were 2002-2004 for children in MoBa1, between 2000-2005 for MoBa2 and between 200-02008 for MoBa3. The establishment and data collection in MoBa has obtained a license from the Norwegian Data Inspectorate and approval from The Regional Committee for Medical Research Ethics. All three studies were approved by the Regional Committee for Ethics in Medical Research, Norway. In addition, MoBa1 and MoBa2 were approved by the Institutional Review Board of the National Institute of Environmental Health Sciences, USA.

The consent given by the participants does not allow for storage of data on an individual level in repositories or journals. Researchers who want access to data sets for replication should submit an application to [datatilgang@fhi.no](mailto:datatilgang@fhi.no). Access to data sets requires approval from The Regional Committee for Medical Research Ethics in Norway and a formal contract with MoBa.

**Definition of covariates**

Paternal and maternal BMI and paternal socioeconomic position (education) was based on maternal self-report from questionnaire data. Smoking status in both parents were also determined from self-report. Parental age (both parents), maternal parity and child sex was recorded from the Norwegian Medical Birth Registry.

**Generation of methylation data and pre-processing methods**

Details of the DNA methylation measurements and quality control for the MoBa1 participants were previously described and the same protocol was implemented for the MoBa2 participants (Joubert et al., 2012). Briefly, umbilical cord blood samples were collected and frozen at birth at -80°C. All biological material was obtained from the Biobank of the MoBa study (Paltiel et al., 2014). Bisulfite conversion was performed using the EZ-96 DNA Methylation kit (Zymo Research Corporation, Irvine, CA) and DNA methylation was measured at 485,577 CpGs in cord blood using Illumina’s Infinium HumanMethylation450 BeadChip. Raw intensity (.idat) files were handled in R using the minfi package to calculate the methylation level at each CpG as the beta-value (β=intensity of the methylated allele (M)/(intensity of the unmethylated allele (U) + intensity of the methylated allele (M) + 100)) and the data was exported for quality control and processing. Probe and sample-specific quality control was performed in the MoBa1 and MoBa2 datasets separately. Similar protocols were applied to MoBa1 and Moba2, as follows: Control probes (N=65) and probes on X (N=11 230) and Y (N=416) chromosomes were excluded in both datasets. Remaining CpGs missing > 10% of methylation data were also removed (N=20 in MoBa1, none in MoBa2). Samples indicated by Illumina to have failed or have an average detection p value across all probes < 0.05 (N=49 MoBa1, N=35 MoBa2) and samples with gender mismatch (N=13 MoBa1, N=8 MoBa2) were also removed. For MoBa1 and MoBa2, we accounted for the two different probe designs by applying the intra-array normalization strategy Beta Mixture Quantile dilation (BMIQ). After quality control exclusions, the sample sizes were 1,068 for MoBa1 and 685 for MoBa2. For MoBa3, samples were completely randomized, and bisulfite conversion and methylation measurements were performed by the Epigenetics Group at IARC (Lyon, France). Similar data quality control and processing was applied with some slight differences. Methylation features were filtered from (i) cross-reactive probes, (ii) probes mapping to sex chromosomes and (iii) probes overlapping with a known single nucleotide polymorphism (SNP) with an allele frequency of at least 5% in the overall population (all ethnic groups), resulting in the exclusion of 36 231 probes. Data quality was further assessed using box plots for the distribution of methylated and unmethylated signals, and multidimensional scaling plots and unsupervised clustering were used to check for sample outliers. After background correction and color-bias adjustment, type I and type II probe distributions were aligned using the intra-sample BMIQ normalization from the watermelon package. After quality control, the sample size for MoBa3 was 253 samples.

**References**

Magnus P, Birke C, Vejrup K, Haugan A, Alsaker E, Daltveit AK, et al. Cohort Profile Update: The Norwegian Mother and Child Cohort Study (MoBa). Int J Epidemiol. 2016;45(2):382-8.

Haberg SE, London SJ, Nafstad P, Nilsen RM, Ueland PM, Vollset SE, et al. Maternal folate levels in pregnancy and asthma in children at age 3 years. J Allergy Clin Immunol. 2011;127(1):262-4, 4 e1.

Joubert BR, Haberg SE, Nilsen RM, Wang X, Vollset SE, Murphy SK, et al. 450K epigenome-wide scan identifies differential DNA methylation in newborns related to maternal smoking during pregnancy. Environ Health Perspect. 2012;120(10):1425-31.

Joubert BR, Felix JF, Yousefi P, Bakulski KM, Just AC, Breton C, et al. DNA Methylation in Newborns and Maternal Smoking in Pregnancy: Genome-wide Consortium Meta-analysis. Am J Hum Genet. 2016;98(4):680-96.

Paltiel L, Haugan A, Skjerden T, Harbak K, Baekken S, Stensrud NK, et al. The biobank of the Norwegian Mother and Child Cohort Study - present status. Norsk Epidemiologi. 2014;24(29):29-35.

## Piccolipiù

**Description of cohort**

Piccolipiù (<https://www.piccolipiu.it/project.html>) is a multicentric Italian birth cohort set up to investigate the effects of environmental exposures, parental conditions and social factors acting during pre-natal and early post-natal life on infant and child health and development (Farchi et al, 2014). Between 2011 and 2015, 3,358 mother-child pairs were recruited in 5 Italian centers (Turin, Trieste, Florence, Viareggio and Rome). Inclusion criteria were: delivery in one of the selected units participating in the study, maternal age older than 18 years, having a singleton pregnancy, residence in the catchment area of the maternity center, ability to fill out the informed consent and the questionnaire in Italian, having a telephone number to be reached at. Ethical approvals have been obtained from the Ethics committees of the Local Health Unit Roma E (management centre), of the Istituto Superiore di Sanità (National Institute of Public Health) and of each local centre. Parents provided written informed consent. A set of 99 children from the Turin centre of Piccolipiù is part of the EXPOsOMICS children project (Vineis et al, 2017) and have available DNA methylome data that have been used in the present study.

**Definition of covariates**

Maternal and paternal characteristics were recorded using questionnaires completed around delivery. Questionnaire data was used to derive paternal BMI, calculated using height and weight reported by the mother, and maternal BMI, calculated from self-reported pre-pregnancy weight and height. Similarly, the following covariates were derived from questionnaires: sex of the child; maternal and paternal age (years); maternal smoking status during pregnancy (any smoking in pregnancy/no smoking in pregnancy); paternal or mother’s partner smoking status during or prior to pregnancy (any smoking one year before the pregnancy/no smoking one year before the pregnancy); parity (one or more previous children/no previous children); and paternal socioeconomic position (university degree or higher/primary or secondary school). Cell type composition was estimated using the Bakulski method (Bakulski et al, 2016).

**Generation of methylation data and pre-processing methods**

A set of 99 children from the Turin centre of Piccolipiù is included in the EXPOsOMICS children studies, for which data on DNA methylome from cord blood are available. DNA methylation levels were measured at the International Agency for Research on Cancer of Lyon in France. DNA was extracted from buffy coats according to standard protocol, followed by bisulphite conversion using the Zymo EZ DNA methylationTM kit (Zymo, Irvine, CA, USA), hybridization to Illumina HumanMethylation 450K BeadChip arrays and scanning using an Illumina iScan. Data quality was initially reviewed using GenomeStudio. , where DNA was extracted (QIAamp 96 DNA Blood Kit, Qiagen 51161), quantified (Quant-iT PicoGreen dsDNA Assay Kit, Molecular Probes P7589), and bisulfite converted (600 ng of DNA using EZ-96 DNA Methylation kit, Zymo Research D5004). DNA methylation was measured at 485,577 CpGs using the Infinium HumanMethylation450 BeadChip (Illumina Inc., San Diego, USA). The arrays were designed such that batch effects (e.g. sample position and intra- and inter-variability in arrays and chips) would not completely confound with biological covariates of interest. This design allows the retention of biological variation even after correction for technical variation. Raw intensity (.idat) files were handled in R using the minfi package (Aryee at al.) to calculate the methylation level at each CpG as the beta-value (β=intensity of the methylated allele (M)/(intensity of the unmethylated allele (U) + intensity of the methylated allele (M) + 100)), and the data were exported for quality control and processing. Data were normalized using the funnorm normalization of the minfi package (Aryee at al.). Methylation features were filtered from cross-reactive probes and low-quality probes (probes having bead counts < 3 in at least 5% of samples). Additionally, samples having > 1% of CpG sites with a detection p-value > 0.05 were removed. Data were trimmed removing the outliers using Tukey method.

**References**

Aryee, M.J. at al. Minfi: a flexible and comprehensive Bioconductor package for the analysis of Infinium DNA methylation microarrays. Bioinformatics 30,1363-9 (2014)

Bakulski, K. M. et al. DNA methylation of cord blood cell types: Applications for mixed cell birth studies. Epigenetics 11, 354-362, doi:10.1080/15592294.2016.1161875 (2016).

Farchi, S. et al. Piccolipiu, a multicenter birth cohort in Italy: protocol of the study. BMC pediatrics 14, 36, doi:10.1186/1471-2431-14-36 (2014).

Vineis, P. et al. The exposome in practice: Design of the EXPOsOMICS project. International journal of hygiene and environmental health 220, 142-151, doi:10.1016/j.ijheh.2016.08.001 (2017).

## Project Viva

**Description of cohort**

Project Viva (<https://www.hms.harvard.edu/viva/index.html> ) is a longitudinal pre-birth cohort of mother-offspring pairs. We enrolled study participants between 1999 and 2002 from Atrius Harvard Vanguard Medical Associates, a multispecialty group practice in eastern Massachusetts. Exclusion criteria included multiple gestation, inability to answer questions in English, gestational age ≥22 weeks at recruitment and plans to move away before delivery. Details on recruitment and data collection have been published elsewhere. (Oken et al. 2015) All mothers in the study signed informed consents and the institutional review board of Harvard Pilgrim Health Care approved this study protocol. We included 324 children in the analysis of DNA-methylation in cord blood and 276 children in the analysis of DNA-methylation in mid-childhood (median 7.6 years) who had non-missing exposure, outcome, and covariate data and white mothers.

**Definition of covariates**

We accessed information on participant demographics and health related behaviours from Project Viva questionnaires and interviews. Mothers reported their age, race/ethnicity, parity, pregnancy smoking status, height and pre-pregnancy weight. Mothers also reported the father’s age, education status, height and weight. We calculated maternal pre-pregnancy BMI and father’s BMI as kg/m^2^. For maternal smoking during pregnancy, we used a dichotomous variable (smoked during pregnancy v. never/former). For paternal social class, we used a dichotomous variable (≥college graduate yes/no).

White blood cell composition was estimated from DNA methylation measurements using the Houseman projection method (Houseman et al., 2012) from isolated cell types. To estimate cell types composition in cord blood we used a reference panel of nucleated cells isolated from cord blood (leukocytes and nucleated red blood cells) (Bakulski et al., 2016) and an adult leukocyte reference panel for blood samples collected in mid-childhood as implemented in *minfi* (Reinius et al., 2012).

**Generation of methylation data and pre-processing methods**

Trained medical personnel obtained umbilical cord blood samples immediately upon delivery storing them in a dedicated refrigerator at 4 °C and transported to a central location within 24 hours of sample collection. Similarly, whole blood samples collected during mid-childhood were stored at 4 °C and transported to the central storage location for sample processing. Subsequently, trained laboratory staff processed the samples on the same day of arrival, and DNA was extracted using the Qiagen Puregene Kit (Valencia, CA). Aliquots were then stored at −80 °C until analysis.

Upon receiving the DNA-methylation data, we removed technical replicates, samples with low quality, genotype mismatch, and sex mismatch. We also excluded individual probes if they had non-significant detection P-values (P > 0.05) for more than 5% of the samples. Additionally, non-CpG probes (i.e. rs and ch), probes in X and Y chromosomes, SNP-associated probes at either the single base extension or within the target region were removed for SNPs that have a minor-allele frequency of >5%. Previously identified non-specific and cross-reactive probes within the array along with polymorphic CpG loci were also excluded from the analysis (Chen et al., 2013). Background correction and dye-bias equalization was performed via the normal-exponential out-of-band (noob) correction method. Finally, a β-mixture quantile intra sample normalization procedure (BMIQ) was applied to the resulting data to reduce the potential bias that can arise from type2 probes. To control for technical variability across sample plates we applied the R package ComBat.

**References**

Bakulski, K. M. *et al.* DNA methylation of cord blood cell types: Applications for mixed cell birth studies. *Epigenetics* 00–00 (2016).

Chen, Yi-an, et al. "Discovery of cross-reactive probes and polymorphic CpGs in the Illumina Infinium HumanMethylation450 microarray." Epigenetics 8.2 (2013): 203-209.

Houseman, E. A. *et al.* DNA methylation arrays as surrogate measures of cell mixture distribution. *BMC bioinformatics* **13**, 1 (2012).

Oken, E, Baccarelli, AA, Gold, DR, Kleinman, KP, Litonjua, AA, De Meo, D, et al. Cohort profile: Project Viva. Int. J. Epidemiol. 2015; 44 (1), 37–48.

Reinius, L. E. *et al.* Differential DNA methylation in purified human blood cells: implications for cell lineage and studies on disease

susceptibility. *PloS one* **7**, e41361 (2012).

## RHEA

**Description of cohort**

The Rhea study (<https://www.isglobal.org/en/-/mother-child-study-in-crete-the-rhea-study> ) is a prospective mother-child cohort examining environmental exposures in the pregnancy and in early childhood in relation to child development and mother’s outcomes as well as their interaction with genetic make-up (Chatzi et al, 2017). Between 2007 and 2008, 1,458 mother-child pairs from Heraklion (Greece) were enrolled in the study during the pregnancy visits or at the delivery. Inclusion criteria were: residence in the study area, maternal age older than 16 years, having performed first antenatal visit in hospitals or private clinics in Heraklion district, and having no communication handicap. Ethics Committee of the University Hospital at Heraklion approved the study protocols and written informed consent was obtained from the mothers. A set of 99 children from the Rhea cohort is part of the EXPOsOMICS children project (Vineis et al, 2017) and have available DNA methylome data that have been used in the present study.

**Definition of covariates**

At the 12^th^ week of gestation information on parents and children were collected using questionnaires. Questionnaire data were used to derive the paternal BMI calculated using paternal height and weight reported by the mother. Similarly, the following covariates were derived from questionnaire data: sex of the child; maternal and paternal age (years); maternal smoking status during pregnancy (any smoking in pregnancy/no smoking in pregnancy); paternal smoking status during the pregnancy (any smoking at 12^th^ and 30^th^ week of the pregnancy/no smoking at 12^th^ and 30^th^ week of the pregnancy); parity (one or more previous children/no previous children); and paternal socioeconomic position (university degree or higher/primary or secondary school). Maternal pre-pregnancy BMI was based on maternal height measured at the first prenatal visit and pre-pregnancy weight reported by the mother. Cell type composition was estimated using the Bakulski method (Bakulski et al, 2016).

**Generation of methylation data and pre-processing methods**

A set of 101 children from the Rhea cohort was included in the EXPOsOMICs study. DNA methylation levels were measured at the International Agency for Research on Cancer of Lyon in France, where DNA was extracted (QIAamp 96 DNA Blood Kit, Qiagen 51161), quantified (Quant-iT PicoGreen dsDNA Assay Kit, Molecular Probes P7589), and bisulfite converted (600 ng of DNA using EZ-96 DNA Methylation kit, Zymo Research D5004). DNA methylation was measured at 485,577 CpGs using the Infinium HumanMethylation450 BeadChip (Illumina Inc., San Diego, USA). The arrays were designed such that batch effects (e.g. sample position and intra- and inter-variability in arrays and chips) would not completely confound with biological covariates of interest. This design allows the retention of biological variation even after correction for technical variation. Raw intensity (.idat) files were handled in R using the minfi package (Aryee at al.) to calculate the methylation level at each CpG as the beta-value (β=intensity of the methylated allele (M)/(intensity of the unmethylated allele (U) + intensity of the methylated allele (M) + 100)), and the data were exported for quality control and processing.The remaining dataset was normalized using the funnorm normalization of the minfi package (Aryee at al.). Methylation features were filtered from cross-reactive probes and low-quality probes (probes having bead counts < 3 in at least 5% of samples). Additionally, samples having > 1% of CpG sites with a detection p-value > 0.05 were removed. Data were trimmed removing the outliers using Turkey method.

**References**

Aryee, M.J. at al. Minfi: a flexible and comprehensive Bioconductor package for the analysis of Infinium DNA methylation microarrays. Bioinformatics 30,1363-9 (2014)

Bakulski, K. M. et al. DNA methylation of cord blood cell types: Applications for mixed cell birth studies. Epigenetics 11, 354-362, doi:10.1080/15592294.2016.1161875 (2016).

Chatzi, L. et al. Cohort Profile: The Mother-Child Cohort in Crete, Greece (Rhea Study). International journal of epidemiology 46, 1392-1393k, doi:10.1093/ije/dyx084 (2017).

Vineis, P. et al. The exposome in practice: Design of the EXPOsOMICS project. International journal of hygiene and environmental health 220, 142-151, doi:10.1016/j.ijheh.2016.08.001 (2017).

# Cohort-specific acknowledgments

**ALSPAC:** We are extremely grateful to all the families who took part in this study, the midwives for their help in recruiting them, and the whole ALSPAC team, which includes interviewers, computer and laboratory technicians, clerical workers, research scientists, volunteers, managers, receptionists, and nurses. We would like to acknowledge Tom Gaunt, Oliver Lyttleton, Sue Ring, Nabila Kazmi, and Geoff Woodward for their earlier contributions to the generation of ARIES data (ALSPAC methylation data).

**BIB:** Born in Bradford is only possible because of the enthusiasm and commitment of the Children and Parents in BiB. We are grateful to all the participants, practitioners and researchers who have made Born in Bradford happen. 450K DNAm and genotype array data was generated in the Bristol Bioresource Laboratory Illumina Facility, University of Bristol.

**CHAMACOS:** We are grateful to the participants of the CHAMACOS study and the laboratory and field staff. We are thankful to Hong Quach, who helped with 450K methylation lab analyses.

**Generation R:** The Generation R Study is conducted by Erasmus MC, University Medical Center Rotterdam, in close collaboration with the School of Law and Faculty of Social Sciences of the Erasmus University Rotterdam, the Municipal Health Service Rotterdam area, Rotterdam, the Rotterdam Homecare Foundation, Rotterdam and the Stichting Trombosedienst & Artsenlaboratorium Rijnmond (STAR-MDC), Rotterdam. We gratefully acknowledge the contribution of children and parents, general practitioners, hospitals, midwives and pharmacies in Rotterdam. The study protocol was approved by the Medical Ethical Committee of the Erasmus Medical Centre, Rotterdam. Written informed consent was obtained for all participants. The generation and management of the Illumina 450K methylation array data (EWAS data) for the Generation R Study was executed by the Human Genotyping Facility of the Genetic Laboratory of the Department of Internal Medicine, Erasmus MC, the Netherlands. We thank Mr. Michael Verbiest, Ms. Mila Jhamai, Ms. Sarah Higgins, Mr. Marijn Verkerk and Dr. Lisette Stolk for their help in creating the EWAS database. We thank Dr. A.Teumer for his work on the quality control and normalization scripts.

**GOYA:** The authors are grateful to all GOYA (Genomics of Obesity in Young Adults) participants and collaborative team members that have contributed to the study. We are also especially grateful to those funding bodies that have contributed GOYA and the Danish National Birth Cohort (from which GOYA was sampled as a case-cohort study) such as the Danish National Research Foundation, Danish Regional Committees, the Pharmacy Foundation, the Egmont Foundation, the March of Dimes Birth Defects Foundation, the Health Foundation and other minor grants. The DNBC Biobank has been supported by the Novo Nordisk Foundation and the Lundbeck Foundation.

**HELIX:** The authors would like to thank all the participating children, parents, practitioners and researchers in the six countries who took part in this study. The authors would like to thank Sonia Brishoual, Angelique Serre and Michele Grosdenier (Poitiers Biobank, CRB BB-0033-00068, Poitiers, France) for biological sample management and Professor Frederic Millot (Principal Investigator), Elodie Migault, Manuela Boue and Sandy Bertin (Clinical Investigation Center, Inserm CIC1402, CHU de Poitiers, Poitiers, France) for planning and investigational actions. The authors would like to thank Veronique Ferrand-Rigalleau, Céline Leger and Noella Gorry (CHU de Poitiers, Poitiers, France) for administrative assistance (EDEN). The authors would like to thank Silvia Fochs, Nuria Pey, Cecilia Persavente and Susana Gros for field work, sample management and overall management

in INMA. The authors would like to thank Georgia Chalkiadaki and Danai Feida for biological sample management, to Eirini Michalaki, Mariza Kampouri, Anny Kyriklaki and Minas Iakovidis for field study performance and to Maria Fasoulaki for administrative assistance (Rhea). The authors would also like to thank Ingvild Essen for thorough field work, Heidi Marie Nordheim for biological sample management and the MoBa administrative unit (MoBa).

**INMA:** INMA researchers would like to thank all the participants for their generous collaboration. A full roster of the INMA Project Investigators can be found at http://www.proyectoinma.org/presentacioninma/listado-investigadores/en_listado-investigadores.html.

**MoBa:** The Norwegian Mother, Father and Child Cohort Study is supported by the Norwegian Ministry of Health and Care Services and the Ministry of Education and Research. We are grateful to all the participating families in Norway who take part in this on-going cohort study.

**Piccolipiù:** Our thanks go to all the families who took part in this study, to the midwives for their help in recruiting them, and to the whole PICCOLIPIU’ team, which includes doctors, nurses, research scientists and computer/laboratory technicians.

**Project Viva:** We thank the participants and staff of Project Viva.

**RHEA:** We are extremely grateful to all the families who took part in the Rhea study, the midwives, research assistants and psychologists for recruiting and following them, and the whole Rhea team, including research scientists, biologists and technicians for their commitment and their role in the success of the study. We thank Mariona Bustamante (CREAL, Barcelona, Spain) for maintaining the Rhea DNA biobank.

# Cohort-specific funding statements

**ALSPAC:** GCS, DAL, GDS, PY and CLR are members of the MRC Integrative Epidemiology Unit, which receives funds from the University of Bristol and United Kingdom Medical Research Council [MC_UU_00011/1, MC_UU_00011/5 and MC_UU_00011/6]. GCS’s contribution to this work is supported by the Medical Research Council [New Investigator Research Grant, MR/S009310/1] and the European Joint Programming Initiative “A Healthy Diet for a Healthy Life” (JPI HDHL, NutriPROGRAM project, UK MRC MR/S036520/1]. DAL’s contribution to this work is supported by grants from the United States National Institutes of Health [R01 DK1034] and the European Union’s Seventh Framework Programme [FP/2007-2013) / ERC Grant Agreement (Grant number 66945; DevelopObese)]. DAL is a National Institute of Health Research Senior Investigator [NF-SI-0611-10196]. The United Kingdom Medical Research Council and Wellcome (Grant ref: 102215/2/13/2) and the University of Bristol provide core support for ALSPAC. The Accessible Resource for Integrated Epigenomics Studies (ARIES) which generated large scale methylation data was funded by the United Kingdom Biotechnology and Biological Sciences Research Council (BB/I025751/1 and BB/I025263/1). Additional epigenetic profiling on the ALSPAC cohort was supported by the United Kingdom Medical Research Council Integrative Epidemiology Unit and the University of Bristol (MC_UU_12013_1, MC_UU_12013_2, MC_UU_12013_5 and MC_UU_12013_8), the Wellcome Trust (WT088806) and the United States National Institute of Diabetes and Digestive and Kidney Diseases (R01 DK10324). This publication is the work of the authors and Gemma Sharp will serve as guarantors for the contents of this paper. The views expressed in this paper are those of the authors and not necessarily any funders. The funders had no influence on the content of the paper.

**Born in Bradford (BIB):** BiB receives core funding from the Wellcome Trust (WT101597MA), the British Heart Foundation (CS/16/4/32482), a joint grant from the UK Medical Research Council (MRC) and UK Economic and Social Science Research Council (ESRC) (MR/N024397/1) and the National Institute for Health Research (NIHR) under its Collaboration for Applied Health Research and Care (CLAHRC) for Yorkshire and Humber. The research presented in this paper, including obtaining genome-wide and epigenome-wide DNAm data is supported by the US National Institute of Health (R01 DK10324) and European Research Council under the European Union’s Seventh Framework Programme (FP7/2007-2013) / ERC grant agreement no 669545. DAL and GCS work in a unit that receives support from the University of Bristol and UK MRC (MC_UU_00011/6) and DAL is an NIHR senior investigator (NF-SI-330 0611-10196).

**CHAMACOS:** This project was supported by grants from the National Institute of Environmental Health Science (NIEHS) [P01 ESO09605, 5UG30D023356, R01ES021369, R01ES023067, R24ES028529, F31ES027751]; Environmental Protection Agency [RD82670901, RD83451301], and the JPB Foundation. Its contents are solely the responsibility of the authors and do not necessarily represent the official views of NIEHS, EPA, or JPB Foundation.

**Generation R:** The general design of the Generation R Study is made possible by financial support from the Erasmus Medical Center, Rotterdam, the Erasmus University Rotterdam, the Netherlands Organization for Health Research and Development and the Ministry of Health, Welfare and Sport. The EWAS data was funded by a grant to VWJ from the Netherlands Genomics Initiative (NGI)/Netherlands Organisation for Scientific Research (NWO) Netherlands Consortium for Healthy Aging (NCHA; project nr. 050-060-810), by funds from the Genetic Laboratory of the Department of Internal Medicine, Erasmus MC, and by a grant from the National Institute of Child and Human Development (R01HD068437). V.W.J. received a grant from the Netherlands Organization for Health Research and Development (VIDI 016.136.361) and a Consolidator Grant from the European Research Council (ERC-2014-CoG-648916). This project received funding from the European Union’s Horizon 2020 research and innovation programme (733206, LIFECYCLE; 633595 DynaHEALTH; 848158, EarlyCause; 874739, LongITools) and from the European Joint Programming Initiative “A Healthy Diet for a Healthy Life” (JPI HDHL, ZonMw the Netherlands, NutriPROGRAM project, no.529051022 and Precise project no. 529051023).

**GOYA:** Genotyping for the GOYA Study was funded by the Wellcome Trust (Grant ref: 084762MA). Generation of DNA methylation data was funded by the MRC Integrative Epidemiology Unit which is supported by the Medical Research Council (MC_UU_00011/5) and the University of Bristol. PY was supported by a UK Biotechnology and Biological Sciences Research Council and Economic and Social Research Council Research Grant (grant number ES/N000498/1).

**HELIX:** The research leading to these results has received funding from the European Community’s Seventh Framework Programme (FP7/2007-206) under grant agreement no 308333—the HELIX project. Dr Maribel Casas received funding from Instituto de Salud Carlos III (Ministry of Economy and Competitiveness) (MS14/00108). The Norwegian Mother and Child Cohort Study (MoBa) is supported by the Norwegian Ministry of Health and the Ministry of Education and Research, NIH/NIEHS (contract no. N01-ES-75558), and NIH/NINDS (grant no. 1 UO1 NS 047537-01 and grant no. 2 UO1 NS 047537-06A1). The Rhea project was financially supported by European projects, and the Greek Ministry of Health (Program of Prevention of Obesity and Neurodevelopmental Disorders in Preschool Children, in Heraklion district, Crete, Greece: 2011–2014; 'Rhea Plus': Primary Prevention Program of Environmental Risk Factors for Reproductive Health, and Child Health: 2012–2015). The work was also supported by MICINN (MTM2015-68140-R) and Centro Nacional de Genotipado-CEGEN-PRB2-ISCIII. We acknowledge support from the Spanish Ministry of Science, Innovation and Universities through the “Centro de Excelencia Severo Ochoa 2019-2023” Program (CEX2018-000806-S), and support from the Generalitat de Catalunya through the CERCA Program.

**INMA:** Main funding of the epigenetic studies in INMA were grants from Instituto de Salud Carlos III (Red INMA G03/176, CB06/02/0041), Spanish Ministry of Health (FIS-PI04/1436, FIS-PI08/1151 including FEDER funds, FIS-PI11/00610, FIS-FEDER-PI06/0867, FIS-FEDER-PI03-1615) Generalitat de Catalunya-CIRIT 1999SGR 00241, Fundació La marató de TV3 (090430), EU Commission (261357-MeDALL: Mechanisms of the Development of ALLergy), and European Research Council (268479-BREATHE: BRain dEvelopment and Air polluTion ultrafine particles in scHool childrEn). Maribel Casas holds a Miguel Servet fellowship (CP16/00128) funded by Instituto de Salud Carlos III and cofounded by European Social Fund ‘Investing in your future’.

**MoBa:** The Norwegian Mother, Father and Child Cohort Study are supported by the Norwegian Ministry of Health and Care Services and the Ministry of Education and Research, NIH/NIEHS (contract no N01-ES-75558), NIH/NINDS (grant no.1 UO1 NS 047537-01 and grant no.2 UO1 NS 047537-06A1). For this work, MoBa 1 and 2 were supported by the Intramural Research Program of the NIH, National Institute of Environmental Health Sciences (Z01-ES-49019) and the Norwegian Research Council/BIOBANK (grant no 221097). This work was partly supported by the Research Council of Norway through its Centres of Excellence funding scheme, project number 262700. MoBa3 epigenomics data analyses were funded by INCA/Plan Cancer-EVA-INSERM, France, and the International Childhood Cancer Cohort Consortium (I4C), and performed by the Epigenetics Group at the International Agency for Research on Cancer (IARC, Lyon, France). Where authors are identified as personnel of the International Agency for Research on Cancer / World Health Organization, the authors alone are responsible for the views expressed in this article and they do not necessarily represent the decisions, policy or views of the International Agency for Research on Cancer / World Health Organization.

**Piccolipiù:** Piccolipiù cohort was approved and initially funded by the Italian National Centre for Disease Prevention and Control (CCM grant 2010) and by the Italian Ministry of Health (art 12 and 12bis Dl.gs.vo 502/92). Where authors are identified as personnel of the International Agency for Research on Cancer / World Health Organization, the authors alone are responsible for the views expressed in this article and they do not necessarily represent the decisions, policy or views of the International Agency for Research on Cancer / World Health Organization.

**Project Viva:** Grants from the US National Institutes of Health (R01 HD034568, UH3 OD023286, R01 HL111108, R01 NR013945).

**RHEA:** Rhea acknowledge all funding sources for the Rhea study: the European Union H2020 (LIFECYCLE), FP7 (HELIX, CHICOS, EnviroGenomarkers, ENRIECO, ESCAPE) and FP6 (HiWate, NewGeneris) programmes. The National Strategic Reference Framework (ESPA) 2007–13, the General Secretariat for Research and Technology in Greece and the Research Committee of the University of Crete, Greece. Funders had no influence of any kind on analyses or results interpretation. Where authors are identified as personnel of the International Agency for Research on Cancer / World Health Organization, the authors alone are responsible for the views expressed in this article and they do not necessarily represent the decisions, policy or views of the International Agency for Research on Cancer / World Health Organization.
