# Supplementary material for: Paternal body mass index and offspring DNA methylation: findings from the PACE consortium
Source: Int J Epidemiol. 2021 Jan 29;50(4):1297–315. doi: 10.1093/ije/dyaa267 (PMC8407864; doi:10.1093/ije/dyaa267)
Supplement: dyaa267_Supplementary_Data [file dyaa267_supplementary_data.zip › ije-2020-05-0817-File008.docx]

**Literature review search strategy**

All code used to carry out this search is available at: <https://github.com/ammegandchips/PACE_Paternal_BMI/blob/master/Literature%20review/Literature%20review.r>

The R package RISmed was used to search PubMed using terms related to fathers, methylation and obesity, but excluding the names of syndromes that involve obesity and epigenetic mechanisms (Prader Villi, Angelman, Beckwith Wiederman). The terms were as follows:

"paternal AND methylation AND obesity NOT ( Prader* OR Angelman* OR beckwith* )"

"father AND methylation AND obesity NOT ( Prader* OR Angelman* OR beckwith* )"

"paternal AND methylation AND overweight NOT ( Prader* OR Angelman* OR beckwith* )"

"father AND methylation AND overweight NOT ( Prader* OR Angelman* OR beckwith* )"

"paternal AND methylation AND BMI NOT ( Prader* OR Angelman* OR beckwith* )"

"father AND methylation AND BMI NOT ( Prader* OR Angelman* OR beckwith* )"

"paternal AND methylation AND body mass index NOT ( Prader* OR Angelman* OR beckwith* )"

"father AND methylation AND body mass index NOT ( Prader* OR Angelman* OR beckwith* )"

"paternal AND methylation AND fat mass NOT ( Prader* OR Angelman* OR beckwith* )"

"father AND methylation AND fat mass NOT ( Prader* OR Angelman* OR beckwith* )"

The search was initially carried out on the 26^th^ of November 2018 and updated on the 29^th^ of July 2019, 20^th^ September 2019 and 10^th^ February 2020.
