# Supplementary material for: Paternal body mass index and offspring DNA methylation: findings from the PACE consortium
Source: Int J Epidemiol. 2021 Jan 29;50(4):1297–315. doi: 10.1093/ije/dyaa267 (PMC8407864; doi:10.1093/ije/dyaa267)
Supplement: dyaa267_Supplementary_Data [file dyaa267_supplementary_data.zip › ije-2020-05-0817-File012.pdf]

Sensitivity analyses: birth

For each model, at CpGs with P<1e-5, we conducted a leave-one-out analysis (systematically leaving one cohort at a time out of the meta-analysis) and plotted the resulting effect estimate and 95% confidence interval.

Contents:

- Paternal BMI (unadjusted for maternal BMI): pages 3-4
- Paternal BMI (adjusted for maternal BMI): pages 6-7
- Paternal BMI adjusted for maternal BMI – female offspring only: pages 9-10
- Paternal BMI adjusted for maternal BMI – male offspring only: page 12

- Maternal BMI (unadjusted for paternal BMI): pages 14-20
- Maternal BMI (adjusted for paternal BMI): pages 22-27
- Maternal BMI adjusted for paternal BMI – female offspring only: pages 29-31
- Maternal BMI adjusted for paternal BMI – male offspring only: pages 33-34

Paternal BMI (unadjusted for maternal BMI): pages 3-4

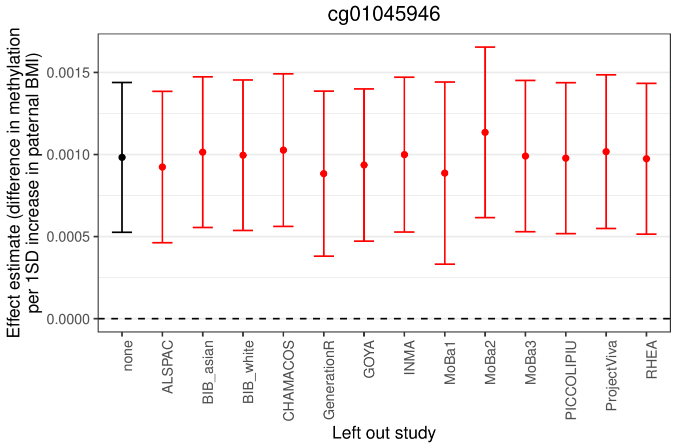
cg01045946
Effect estimate (difference in methylation per 1SD increase in paternal BMI)
0.0015
0.0010
0.0005
0.0000
none
ALSPAC
BIB_asian
BIB_white
CHAMACOS
GenerationR
GOYA
INMA
MoBa1
MoBa2
MoBa3
PICCOLIPIU
ProjectViva
RHEA
Left out study

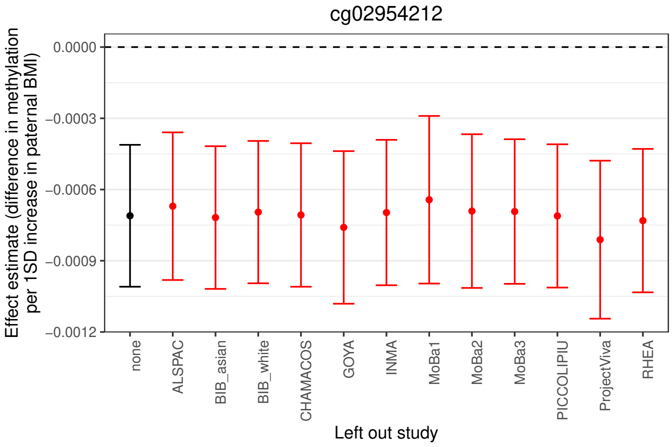
cg02954212
Effect estimate (difference in methylation per 1SD increase in paternal BMI)
0.0000
−0.0003
−0.0006
−0.0009
−0.0012
none
ALSPAC
BIB_asian
BIB_white
CHAMACOS
GOYA
INMA
MoBa1
MoBa2
MoBa3
PICCOLIPIU
ProjectViva
RHEA
Left out study

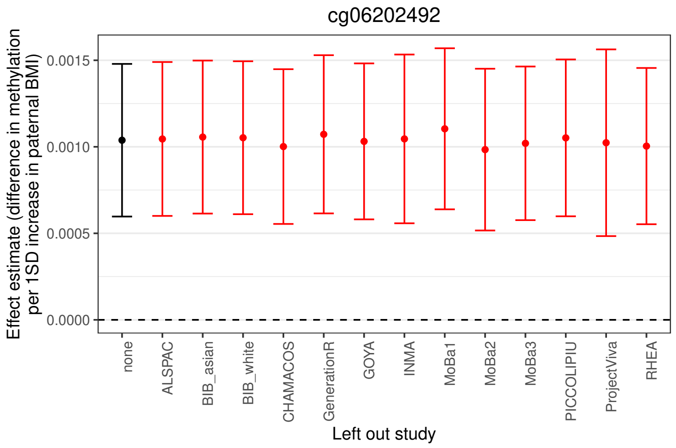
cg06202492
Effect estimate (difference in methylation per 1SD increase in paternal BMI)
0.0015
0.0010
0.0005
0.0000
none
ALSPAC
BIB_asian
BIB_white
CHAMACOS
GenerationR
GOYA
INMA
MoBa1
MoBa2
MoBa3
PICCOLIPIU
ProjectViva
RHEA
Left out study

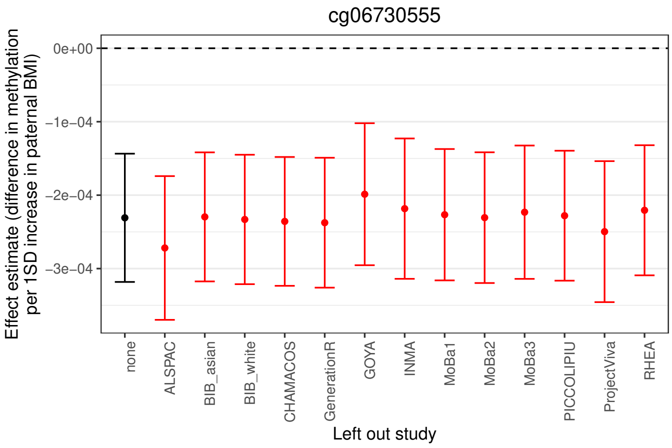
cg06730555
Effect estimate (difference in methylation per 1SD increase in paternal BMI)
0e+00
−1e−04
−2e−04
−3e−04
none
ALSPAC
BIB_asian
BIB_white
CHAMACOS
GenerationR
GOYA
INMA
MoBa1
MoBa2
MoBa3
PICCOLIPIU
ProjectViva
RHEA
Left out study

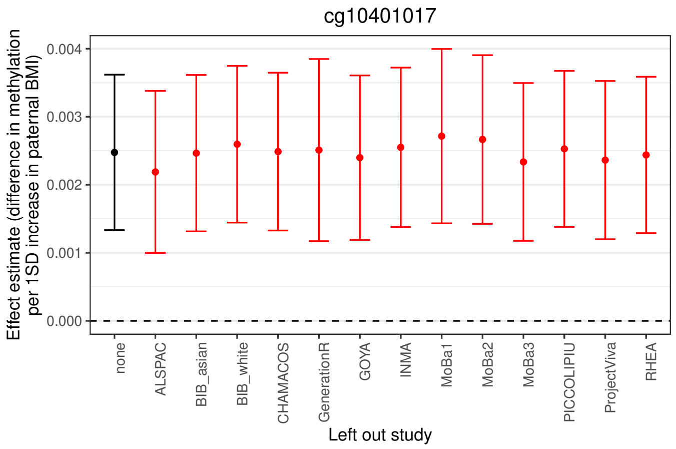
cg10401017
Effect estimate (difference in methylation per 1SD increase in paternal BMI)
0.004
0.003
0.002
0.001
0.000
none
ALSPAC
BIB_asian
BIB_white
CHAMACOS
GenerationR
GOYA
INMA
MoBa1
MoBa2
MoBa3
PICCOLIPIU
ProjectViva
RHEA
Left out study

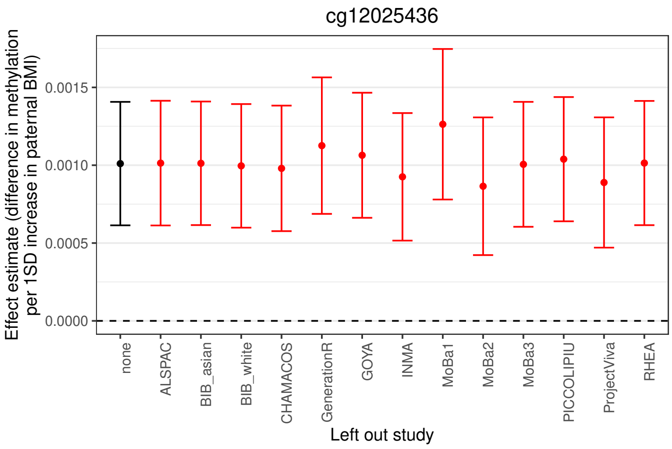
cg12025436
Effect estimate (difference in methylation per 1SD increase in paternal BMI)
0.0015
0.0010
0.0005
0.0000
none
ALSPAC
BIB_asian
BIB_white
CHAMACOS
GenerationR
GOYA
INMA
MoBa1
MoBa2
MoBa3
PICCOLIPIU
ProjectViva
RHEA
Left out study

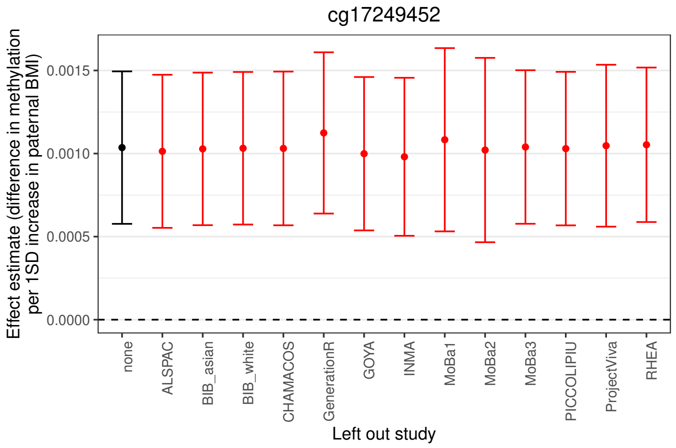
cg17249452
Effect estimate (difference in methylation per 1SD increase in paternal BMI)
0.0015
0.0010
0.0005
0.0000
none
ALSPAC
BIB_asian
BIB_white
CHAMACOS
GenerationR
GOYA
INMA
MoBa1
MoBa2
MoBa3
PICCOLIPIU
ProjectViva
RHEA
Left out study

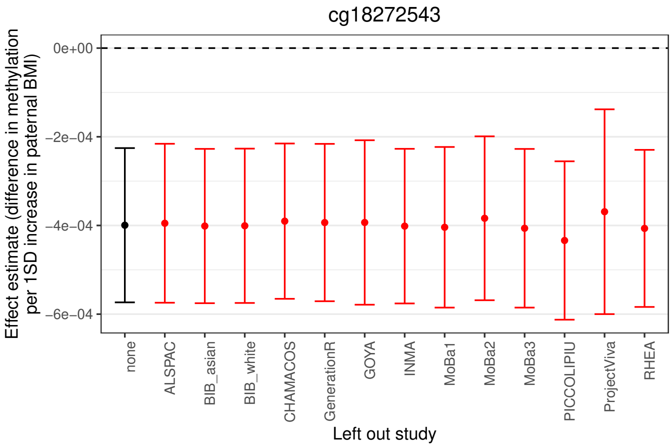
cg18272543
Effect estimate (difference in methylation per 1SD increase in paternal BMI)
0e+00
−2e−04
−4e−04
−6e−04
none
ALSPAC
BIB_asian
BIB_white
CHAMACOS
GenerationR
GOYA
INMA
MoBa1
MoBa2
MoBa3
PICCOLIPIU
ProjectViva
RHEA
Left out study

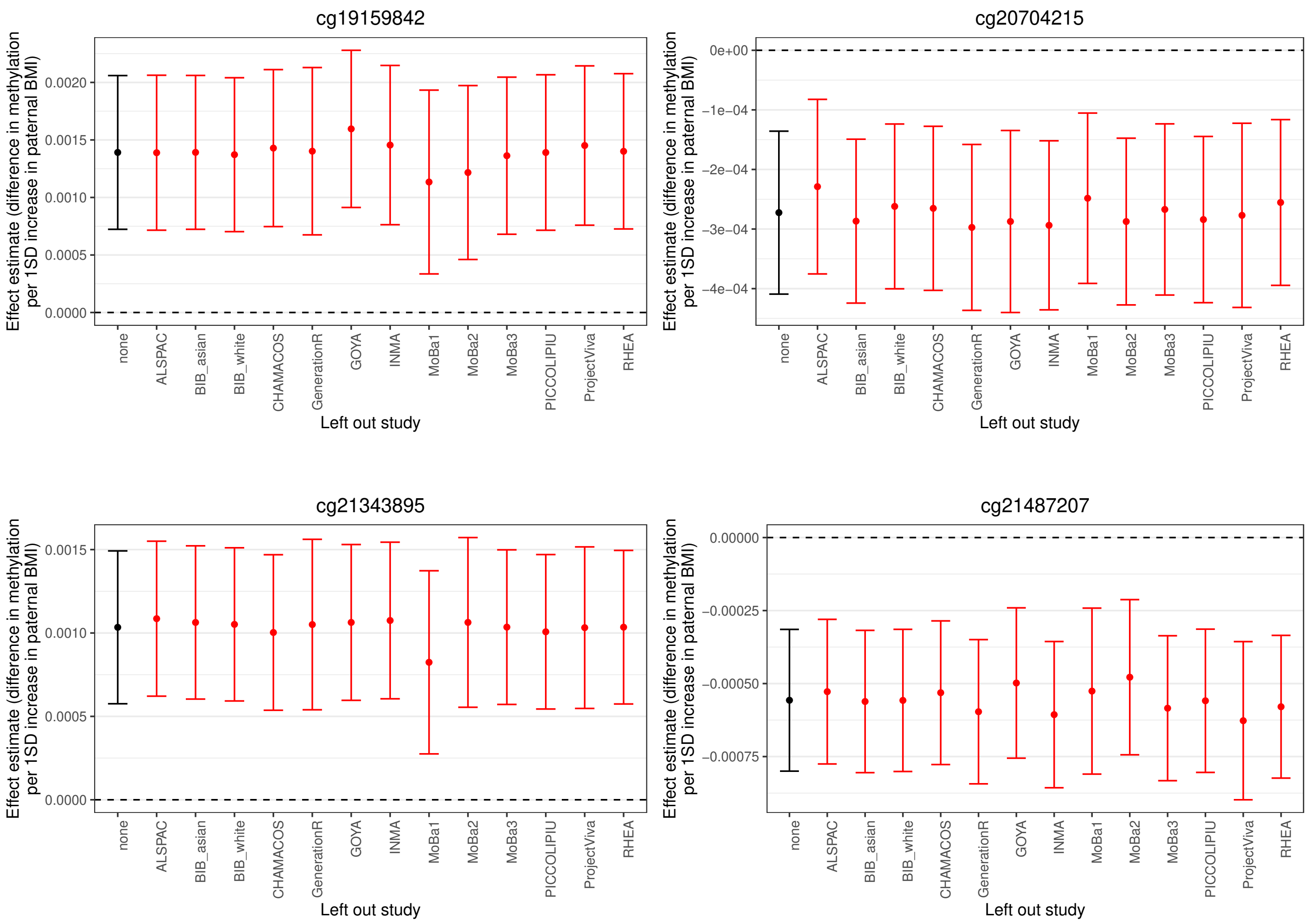
cg19159842
cg20704215
cg21343895
cg21487207
Effect estimate (difference in methylation per 1SD increase in paternal BMI)
Left out study
none
ALSPAC
BIB_asian
BIB_white
CHAMACOS
GenerationR
GOYA
INMA
MoBa1
MoBa2
MoBa3
PICCOLIPIU
ProjectViva
RHEA
0.0000
0.0005
0.0010
0.0015
0.0020
0e+00
−1e−04
−2e−04
−3e−04
−4e−04
0.00000
−0.00025
−0.00050
−0.00075

Paternal BMI (adjusted for maternal BMI): pages 6-7

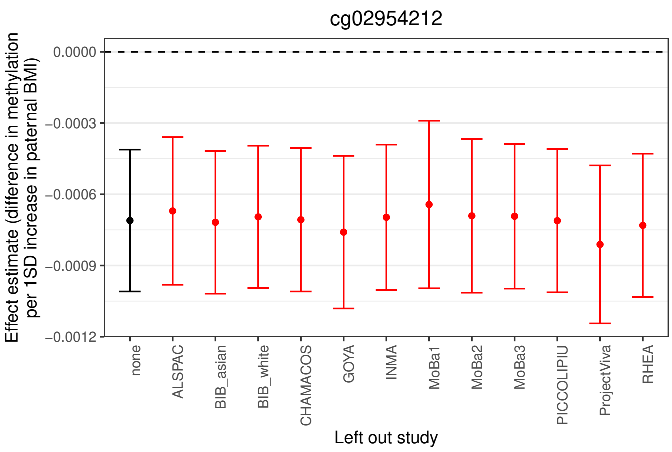
cg02954212
Effect estimate (difference in methylation per 1SD increase in paternal BMI)
0.0000
−0.0003
−0.0006
−0.0009
−0.0012
none
ALSPAC
BIB_asian
BIB_white
CHAMACOS
GOYA
INMA
MoBa1
MoBa2
MoBa3
PICCOLIPIU
ProjectViva
RHEA
Left out study

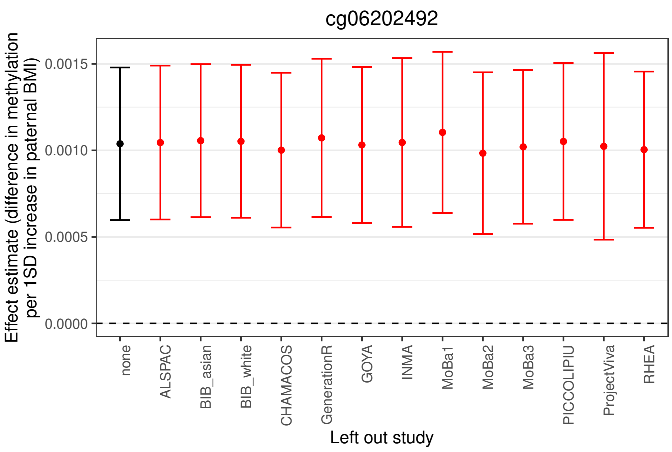
cg06202492
Effect estimate (difference in methylation per 1SD increase in paternal BMI)
0.0015
0.0010
0.0005
0.0000
none
ALSPAC
BIB_asian
BIB_white
CHAMACOS
GenerationR
GOYA
INMA
MoBa1
MoBa2
MoBa3
PICCOLIPIU
ProjectViva
RHEA
Left out study

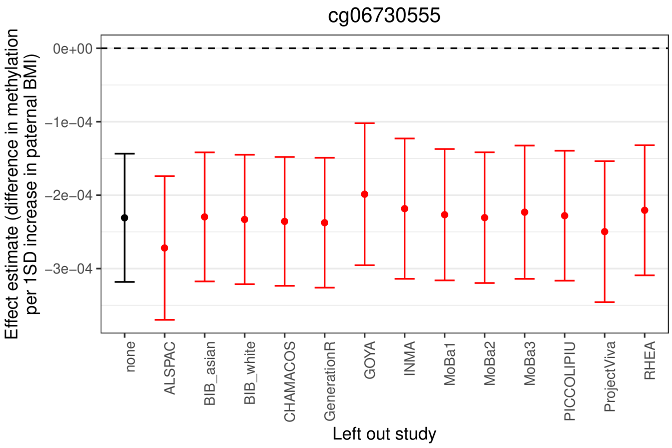
cg06730555
Effect estimate (difference in methylation per 1SD increase in paternal BMI)
0e+00
−1e−04
−2e−04
−3e−04
none
ALSPAC
BIB_asian
BIB_white
CHAMACOS
GenerationR
GOYA
INMA
MoBa1
MoBa2
MoBa3
PICCOLIPIU
ProjectViva
RHEA
Left out study

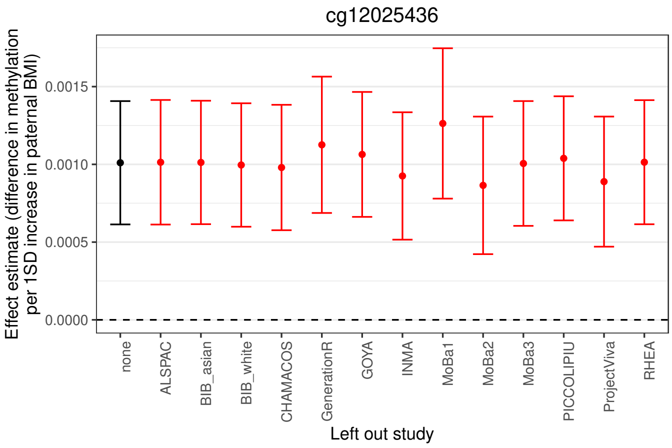
cg12025436
Effect estimate (difference in methylation per 1SD increase in paternal BMI)
0.0015
0.0010
0.0005
0.0000
none
ALSPAC
BIB_asian
BIB_white
CHAMACOS
GenerationR
GOYA
INMA
MoBa1
MoBa2
MoBa3
PICCOLIPIU
ProjectViva
RHEA
Left out study

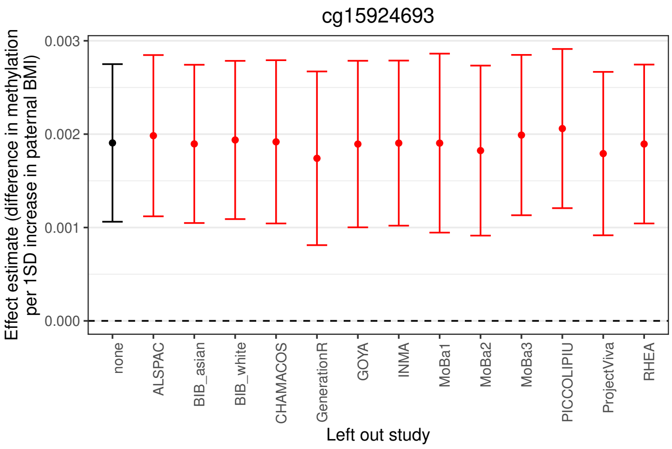
cg15924693
Effect estimate (difference in methylation per 1SD increase in paternal BMI)
0.003
0.002
0.001
0.000
none
ALSPAC
BIB_asian
BIB_white
CHAMACOS
GenerationR
GOYA
INMA
MoBa1
MoBa2
MoBa3
PICCOLIPIU
ProjectViva
RHEA
Left out study

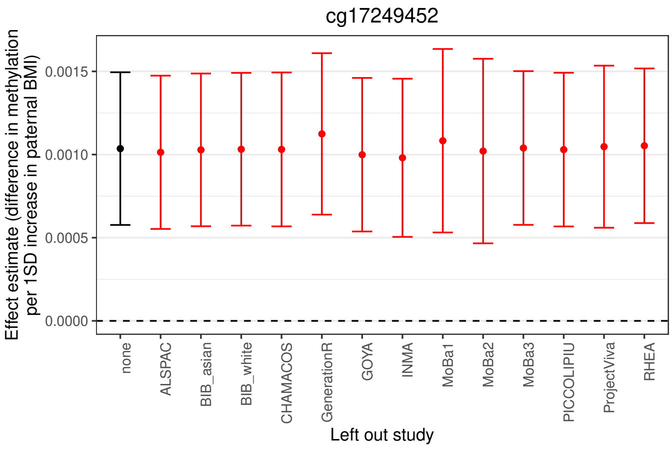
cg17249452
Effect estimate (difference in methylation per 1SD increase in paternal BMI)
0.0015
0.0010
0.0005
0.0000
none
ALSPAC
BIB_asian
BIB_white
CHAMACOS
GenerationR
GOYA
INMA
MoBa1
MoBa2
MoBa3
PICCOLIPIU
ProjectViva
RHEA
Left out study

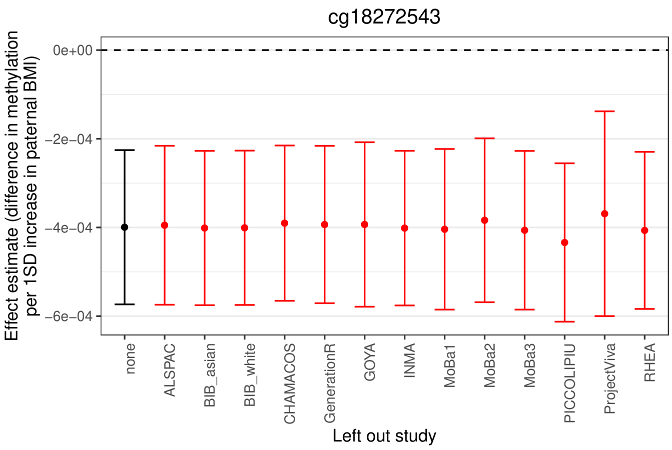
cg18272543
Effect estimate (difference in methylation per 1SD increase in paternal BMI)
0e+00
−2e−04
−4e−04
−6e−04
none
ALSPAC
BIB_asian
BIB_white
CHAMACOS
GenerationR
GOYA
INMA
MoBa1
MoBa2
MoBa3
PICCOLIPIU
ProjectViva
RHEA
Left out study

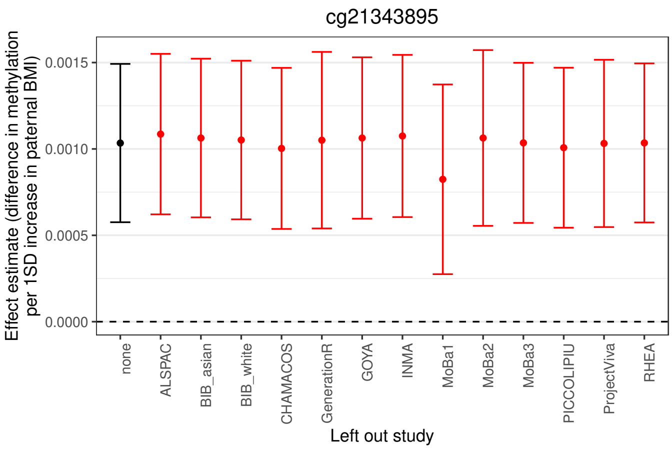
cg21343895
Effect estimate (difference in methylation per 1SD increase in paternal BMI)
0.0015
0.0010
0.0005
0.0000
none
ALSPAC
BIB_asian
BIB_white
CHAMACOS
GenerationR
GOYA
INMA
MoBa1
MoBa2
MoBa3
PICCOLIPIU
ProjectViva
RHEA
Left out study

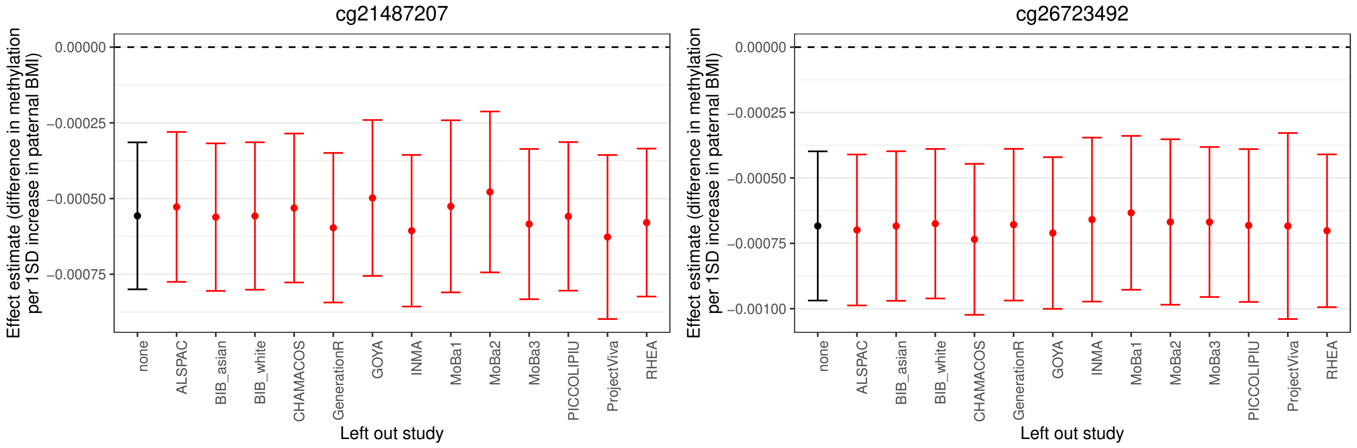
cg21487207
Effect estimate (difference in methylation per 1SD increase in paternal BMI)
0.00000
−0.00025
−0.00050
−0.00075
none
ALSPAC
BIB_asian
BIB_white
CHAMACOS
GenerationR
GOYA
INMA
MoBa1
MoBa2
MoBa3
PICCOLIPIU
ProjectViva
RHEA
Left out study
cg26723492
Effect estimate (difference in methylation per 1SD increase in paternal BMI)
0.00000
−0.00025
−0.00050
−0.00075
−0.00100
none
ALSPAC
BIB_asian
BIB_white
CHAMACOS
GenerationR
GOYA
INMA
MoBa1
MoBa2
MoBa3
PICCOLIPIU
ProjectViva
RHEA
Left out study

Paternal BMI adjusted for maternal BMI – female offspring only: pages 9-10

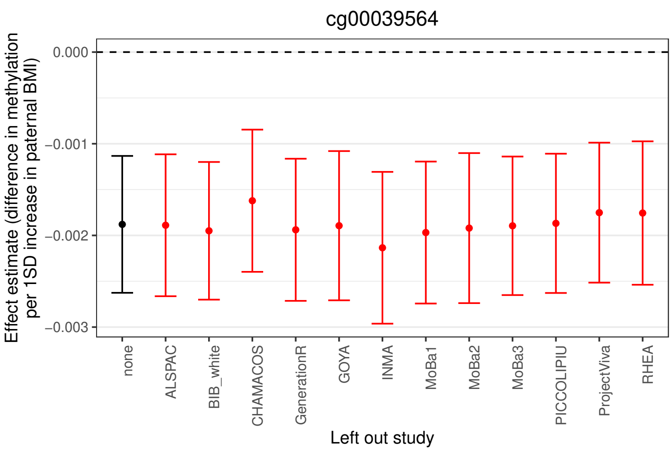
cg00039564
Effect estimate (difference in methylation per 1SD increase in paternal BMI)
0.000
−0.001
−0.002
−0.003
none
ALSPAC
BIB_white
CHAMACOS
GenerationR
GOYA
INMA
MoBa1
MoBa2
MoBa3
PICCOLIPIU
ProjectViva
RHEA
Left out study

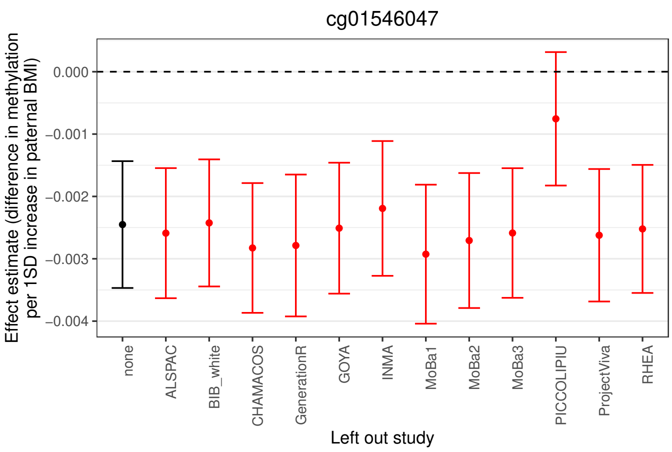
cg01546047
Effect estimate (difference in methylation per 1SD increase in paternal BMI)
0.000
−0.001
−0.002
−0.003
−0.004
none
ALSPAC
BIB_white
CHAMACOS
GenerationR
GOYA
INMA
MoBa1
MoBa2
MoBa3
PICCOLIPIU
ProjectViva
RHEA
Left out study

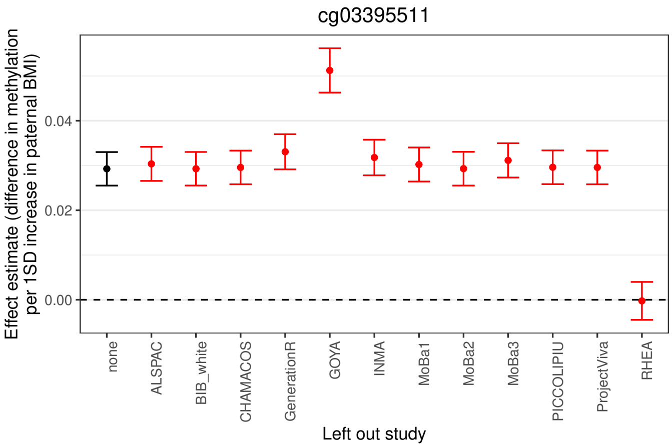
cg03395511
Effect estimate (difference in methylation per 1SD increase in paternal BMI)
0.04
0.02
0.00
none
ALSPAC
BIB_white
CHAMACOS
GenerationR
GOYA
INMA
MoBa1
MoBa2
MoBa3
PICCOLIPIU
ProjectViva
RHEA
Left out study

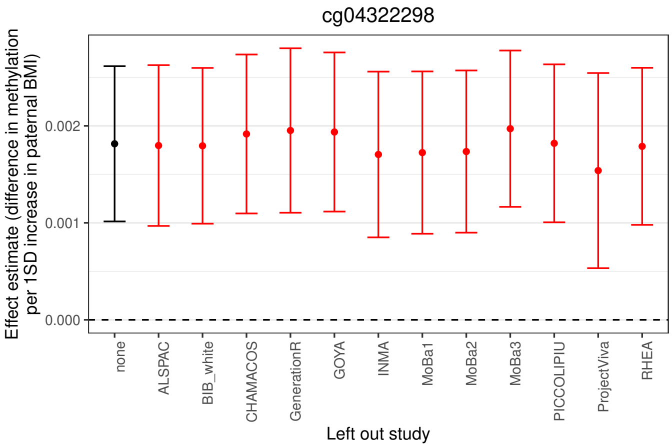
cg04322298
Effect estimate (difference in methylation per 1SD increase in paternal BMI)
0.002
0.001
0.000
none
ALSPAC
BIB_white
CHAMACOS
GenerationR
GOYA
INMA
MoBa1
MoBa2
MoBa3
PICCOLIPIU
ProjectViva
RHEA
Left out study

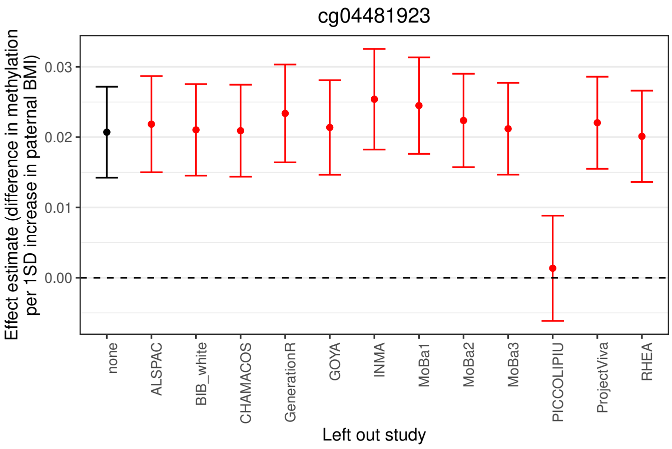
cg04481923
Effect estimate (difference in methylation per 1SD increase in paternal BMI)
0.03
0.02
0.01
0.00
none
ALSPAC
BIB_white
CHAMACOS
GenerationR
GOYA
INMA
MoBa1
MoBa2
MoBa3
PICCOLIPIU
ProjectViva
RHEA
Left out study

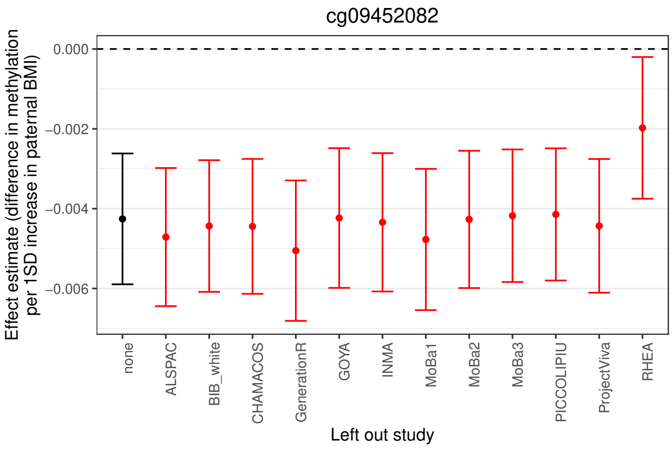
cg09452082
Effect estimate (difference in methylation per 1SD increase in paternal BMI)
0.000
−0.002
−0.004
−0.006
none
ALSPAC
BIB_white
CHAMACOS
GenerationR
GOYA
INMA
MoBa1
MoBa2
MoBa3
PICCOLIPIU
ProjectViva
RHEA
Left out study

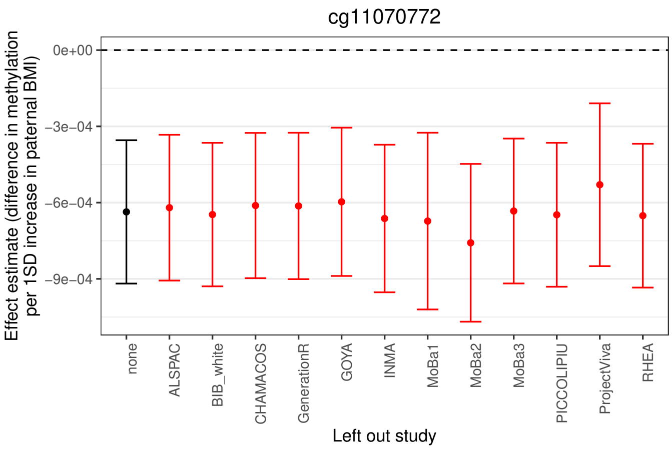
cg11070772
Effect estimate (difference in methylation per 1SD increase in paternal BMI)
0e+00
−3e−04
−6e−04
−9e−04
none
ALSPAC
BIB_white
CHAMACOS
GenerationR
GOYA
INMA
MoBa1
MoBa2
MoBa3
PICCOLIPIU
ProjectViva
RHEA
Left out study

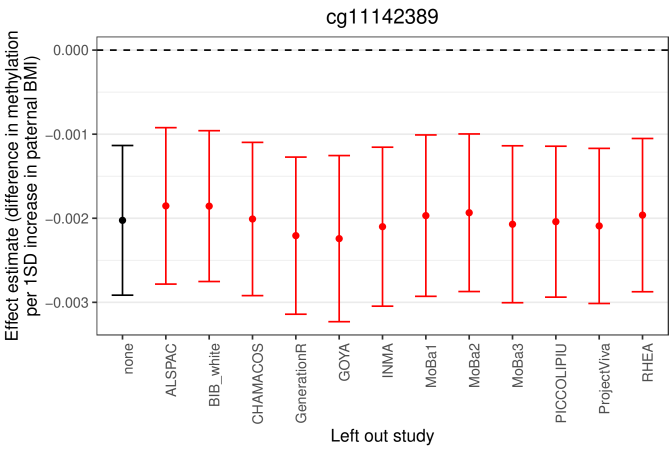
cg11142389
Effect estimate (difference in methylation per 1SD increase in paternal BMI)
0.000
−0.001
−0.002
−0.003
none
ALSPAC
BIB_white
CHAMACOS
GenerationR
GOYA
INMA
MoBa1
MoBa2
MoBa3
PICCOLIPIU
ProjectViva
RHEA
Left out study

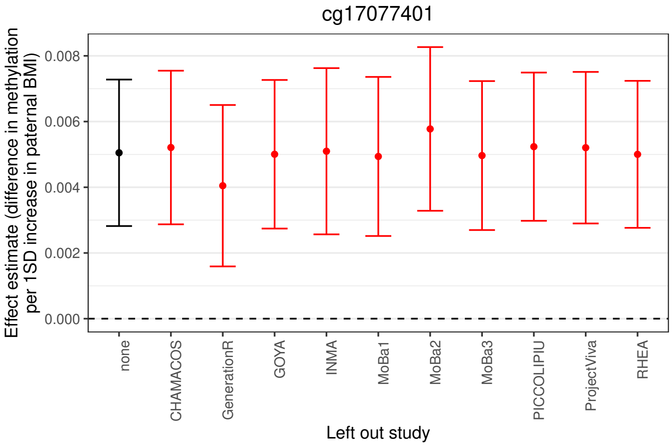
cg17077401
Effect estimate (difference in methylation per 1SD increase in paternal BMI)
0.008
0.006
0.004
0.002
0.000
none
CHAMACOS
GenerationR
GOYA
INMA
MoBa1
MoBa2
MoBa3
PICCOLIPIU
ProjectViva
RHEA
Left out study

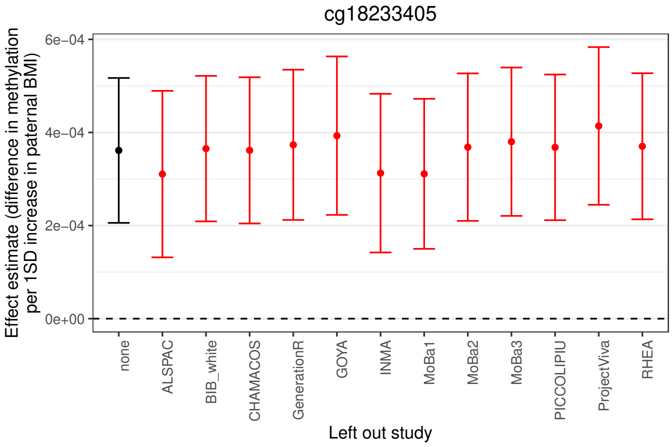
cg18233405
Effect estimate (difference in methylation per 1SD increase in paternal BMI)
6e−04
4e−04
2e−04
0e+00
none
ALSPAC
BIB_white
CHAMACOS
GenerationR
GOYA
INMA
MoBa1
MoBa2
MoBa3
PICCOLIPIU
ProjectViva
RHEA
Left out study

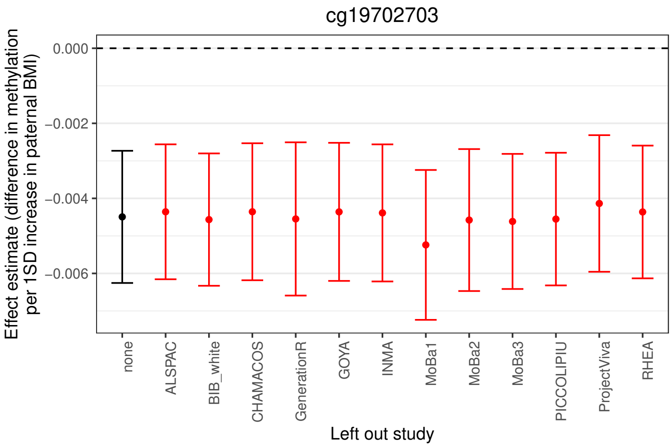
cg19702703
Effect estimate (difference in methylation per 1SD increase in paternal BMI)
0.000
−0.002
−0.004
−0.006
none
ALSPAC
BIB_white
CHAMACOS
GenerationR
GOYA
INMA
MoBa1
MoBa2
MoBa3
PICCOLIPIU
ProjectViva
RHEA
Left out study

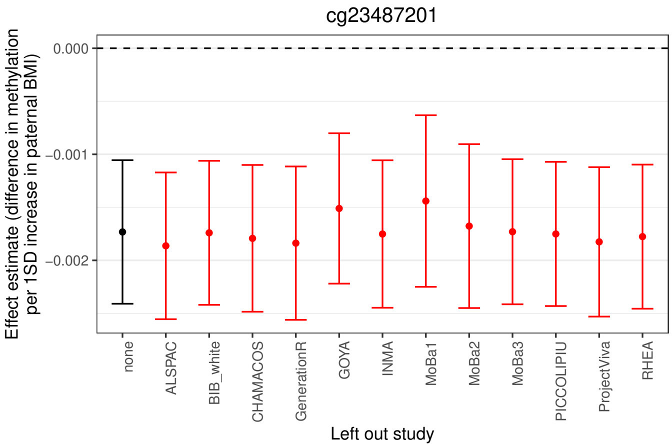
cg23487201
Effect estimate (difference in methylation per 1SD increase in paternal BMI)
0.000
−0.001
−0.002
none
ALSPAC
BIB_white
CHAMACOS
GenerationR
GOYA
INMA
MoBa1
MoBa2
MoBa3
PICCOLIPIU
ProjectViva
RHEA
Left out study

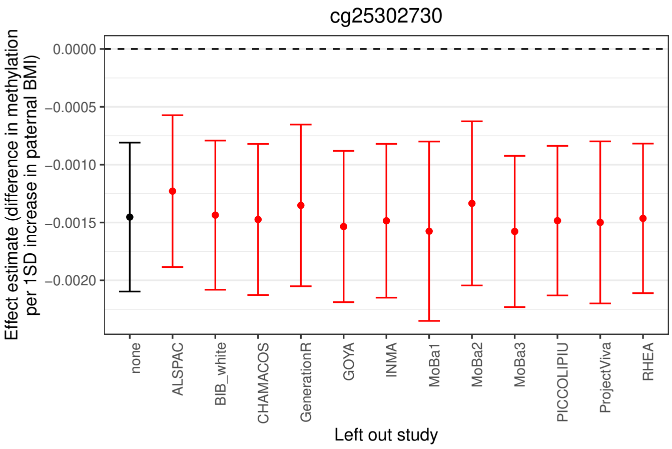
cg25302730
Effect estimate (difference in methylation per 1SD increase in paternal BMI)
0.0000
−0.0005
−0.0010
−0.0015
−0.0020
none
ALSPAC
BIB_white
CHAMACOS
GenerationR
GOYA
INMA
MoBa1
MoBa2
MoBa3
PICCOLIPIU
ProjectViva
RHEA
Left out study

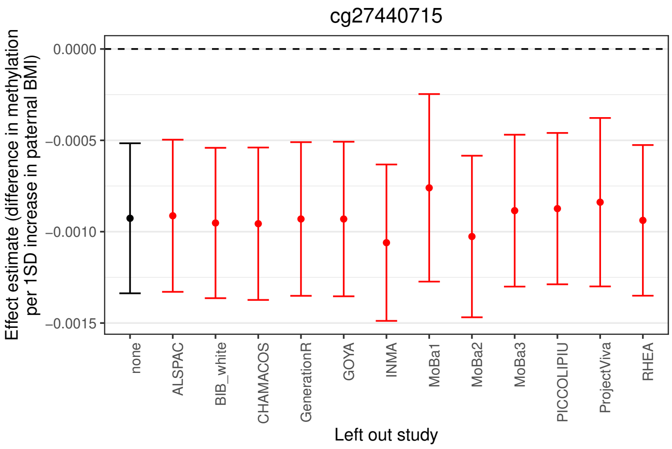
cg27440715
Effect estimate (difference in methylation per 1SD increase in paternal BMI)
0.0000
−0.0005
−0.0010
−0.0015
none
ALSPAC
BIB_white
CHAMACOS
GenerationR
GOYA
INMA
MoBa1
MoBa2
MoBa3
PICCOLIPIU
ProjectViva
RHEA
Left out study

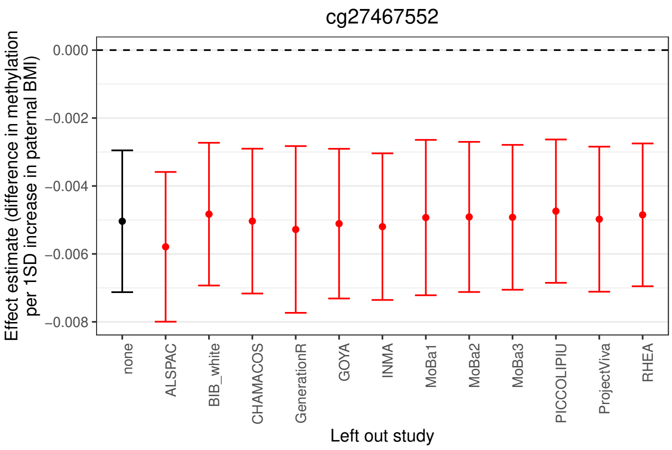
cg27467552
Effect estimate (difference in methylation per 1SD increase in paternal BMI)
0.000
−0.002
−0.004
−0.006
−0.008
none
ALSPAC
BIB_white
CHAMACOS
GenerationR
GOYA
INMA
MoBa1
MoBa2
MoBa3
PICCOLIPIU
ProjectViva
RHEA
Left out study

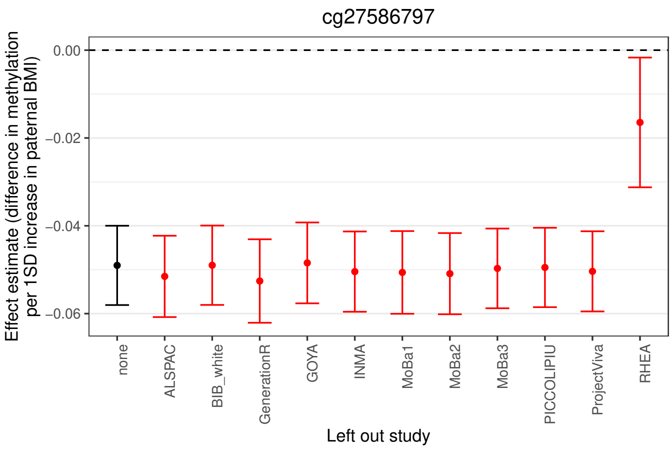
cg27586797
Effect estimate (difference in methylation per 1SD increase in paternal BMI)
0.00
−0.02
−0.04
−0.06
none
ALSPAC
BIB_white
GenerationR
GOYA
INMA
MoBa1
MoBa2
MoBa3
PICCOLIPIU
ProjectViva
RHEA
Left out study

Paternal BMI adjusted for maternal BMI – male offspring only: page 12

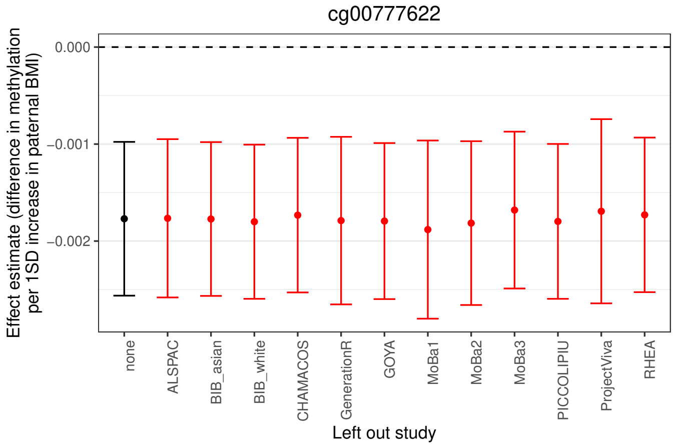
cg00777622
Effect estimate (difference in methylation per 1SD increase in paternal BMI)
0.000
-0.001
-0.002
none
ALSPAC
BIB_asian
BIB_white
CHAMACOS
GenerationR
GOYA
MoBa1
MoBa2
MoBa3
PICCOLIPIU
ProjectViva
RHEA
Left out study

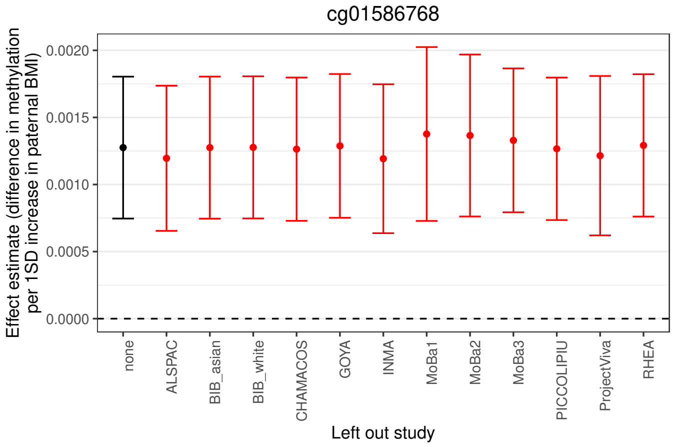
cg01586768
Effect estimate (difference in methylation per 1SD increase in paternal BMI)
0.0020
0.0015
0.0010
0.0005
0.0000
none
ALSPAC
BIB_asian
BIB_white
CHAMACOS
GOYA
INMA
MoBa1
MoBa2
MoBa3
PICCOLIPIU
ProjectViva
RHEA
Left out study

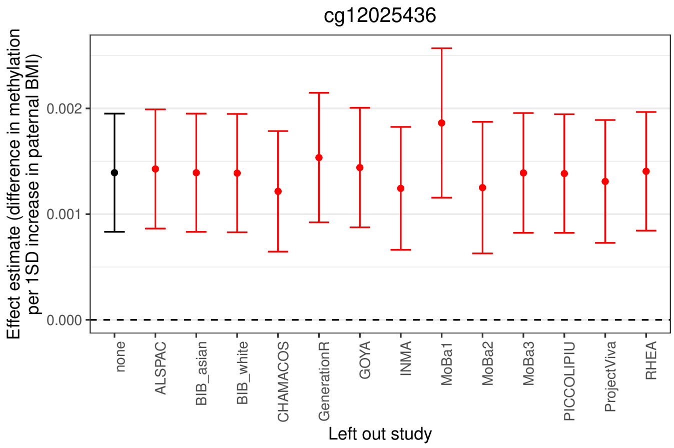
cg12025436
Effect estimate (difference in methylation per 1SD increase in paternal BMI)
0.002
0.001
0.000
none
ALSPAC
BIB_asian
BIB_white
CHAMACOS
GenerationR
GOYA
INMA
MoBa1
MoBa2
MoBa3
PICCOLIPIU
ProjectViva
RHEA
Left out study

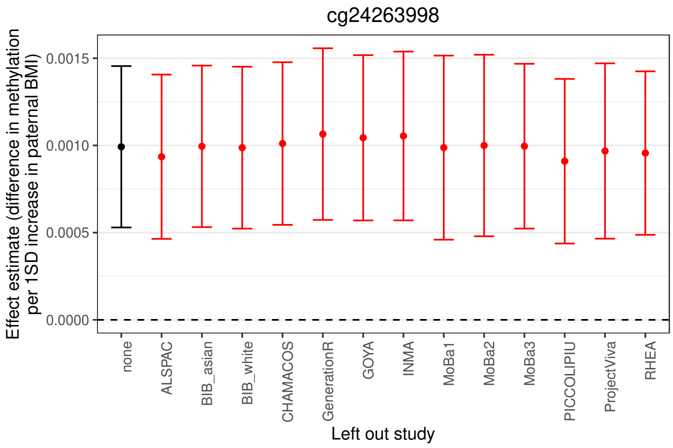
cg24263998
Effect estimate (difference in methylation per 1SD increase in paternal BMI)
0.0015
0.0010
0.0005
0.0000
none
ALSPAC
BIB_asian
BIB_white
CHAMACOS
GenerationR
GOYA
INMA
MoBa1
MoBa2
MoBa3
PICCOLIPIU
ProjectViva
RHEA
Left out study

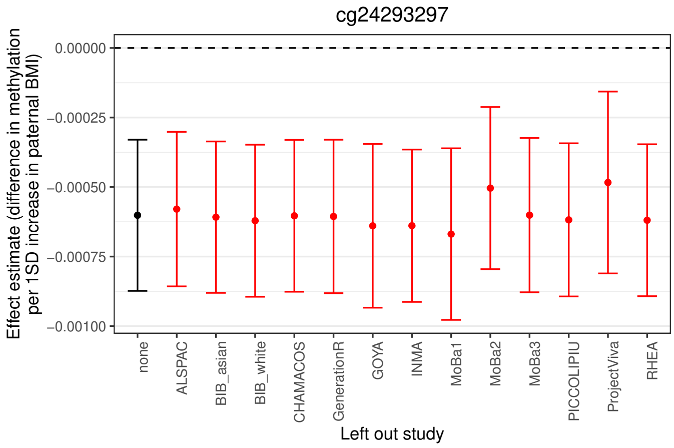
cg24293297
Effect estimate (difference in methylation per 1SD increase in paternal BMI)
0.00000
-0.00025
-0.00050
-0.00075
-0.00100
none
ALSPAC
BIB_asian
BIB_white
CHAMACOS
GenerationR
GOYA
INMA
MoBa1
MoBa2
MoBa3
PICCOLIPIU
ProjectViva
RHEA
Left out study

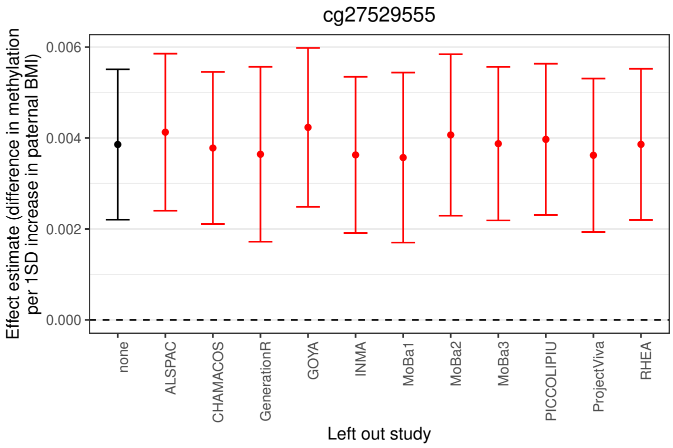
cg27529555
Effect estimate (difference in methylation per 1SD increase in paternal BMI)
0.006
0.004
0.002
0.000
none
ALSPAC
CHAMACOS
GenerationR
GOYA
INMA
MoBa1
MoBa2
MoBa3
PICCOLIPIU
ProjectViva
RHEA
Left out study

Maternal BMI (unadjusted for paternal BMI): pages 14-20

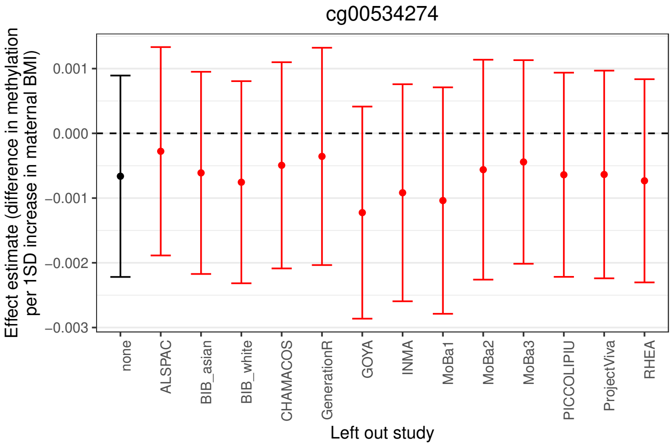
cg00534274
Effect estimate (difference in methylation per 1SD increase in maternal BMI)
0.001
0.000
−0.001
−0.002
−0.003
none
ALSPAC
BIB_asian
BIB_white
CHAMACOS
GenerationR
GOYA
INMA
MoBa1
MoBa2
MoBa3
PICCOLIPIU
ProjectViva
RHEA
Left out study

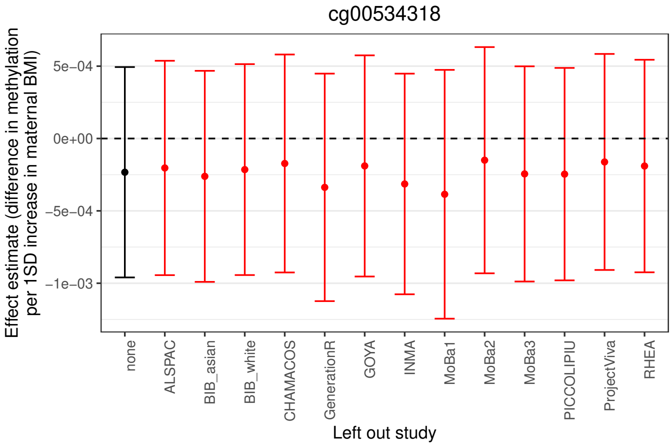
cg00534318
Effect estimate (difference in methylation per 1SD increase in maternal BMI)
5e−04
0e+00
−5e−04
−1e−03
none
ALSPAC
BIB_asian
BIB_white
CHAMACOS
GenerationR
GOYA
INMA
MoBa1
MoBa2
MoBa3
PICCOLIPIU
ProjectViva
RHEA
Left out study

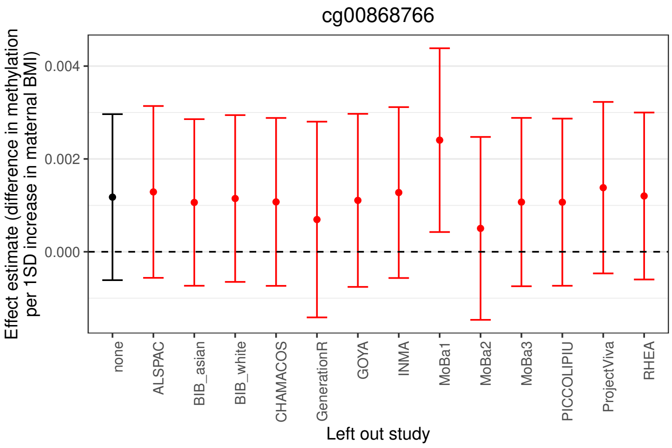
cg00868766
Effect estimate (difference in methylation per 1SD increase in maternal BMI)
0.004
0.002
0.000
none
ALSPAC
BIB_asian
BIB_white
CHAMACOS
GenerationR
GOYA
INMA
MoBa1
MoBa2
MoBa3
PICCOLIPIU
ProjectViva
RHEA
Left out study

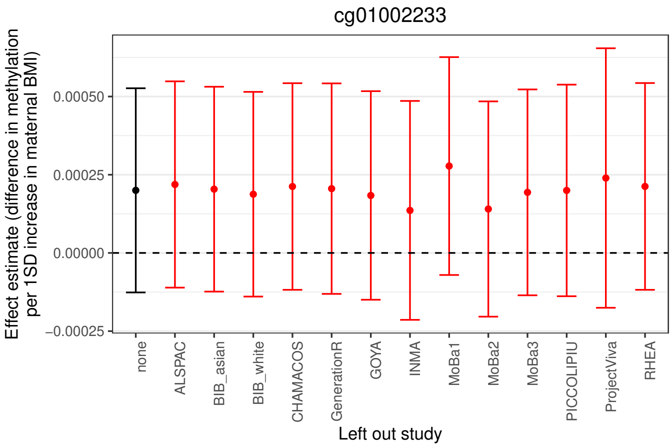
cg01002233
Effect estimate (difference in methylation per 1SD increase in maternal BMI)
0.00050
0.00025
0.00000
−0.00025
none
ALSPAC
BIB_asian
BIB_white
CHAMACOS
GenerationR
GOYA
INMA
MoBa1
MoBa2
MoBa3
PICCOLIPIU
ProjectViva
RHEA
Left out study

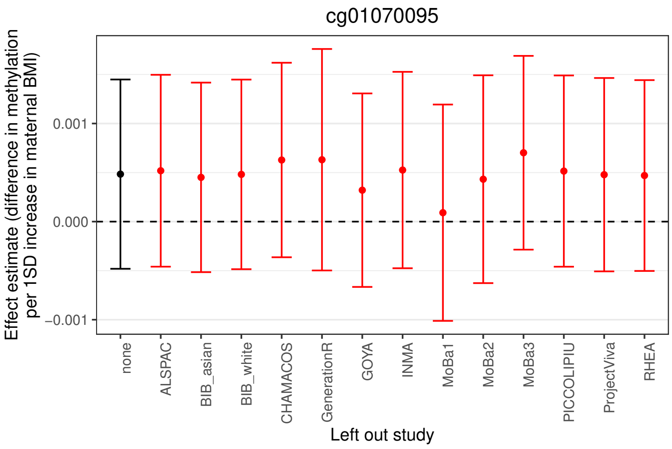
cg01070095
Effect estimate (difference in methylation per 1SD increase in maternal BMI)
0.001
0.000
−0.001
none
ALSPAC
BIB_asian
BIB_white
CHAMACOS
GenerationR
GOYA
INMA
MoBa1
MoBa2
MoBa3
PICCOLIPIU
ProjectViva
RHEA
Left out study

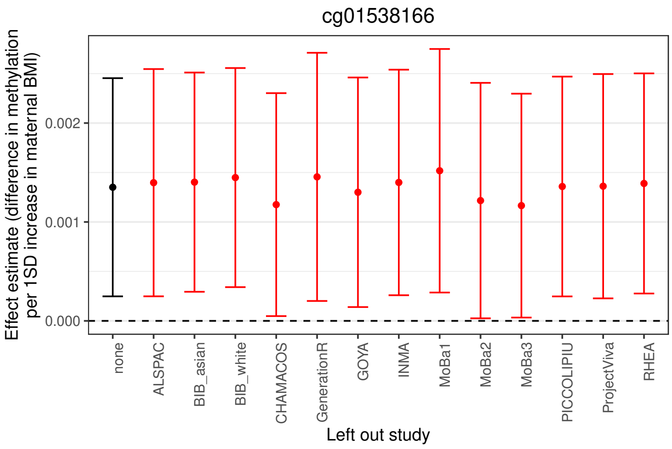
cg01538166
Effect estimate (difference in methylation per 1SD increase in maternal BMI)
0.002
0.001
0.000
none
ALSPAC
BIB_asian
BIB_white
CHAMACOS
GenerationR
GOYA
INMA
MoBa1
MoBa2
MoBa3
PICCOLIPIU
ProjectViva
RHEA
Left out study

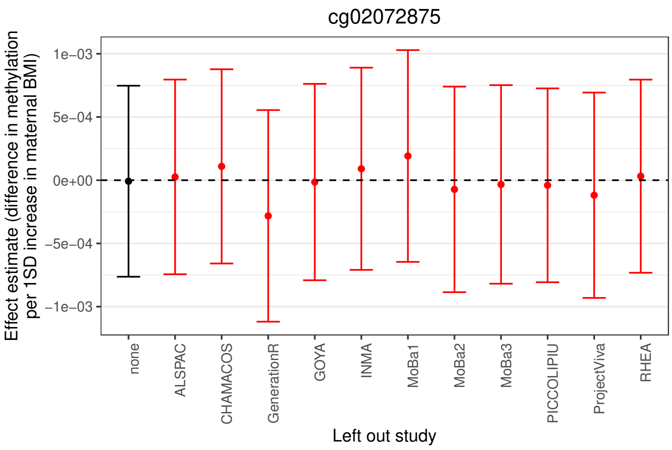
cg02072875
Effect estimate (difference in methylation per 1SD increase in maternal BMI)
1e−03
5e−04
0e+00
−5e−04
−1e−03
none
ALSPAC
CHAMACOS
GenerationR
GOYA
INMA
MoBa1
MoBa2
MoBa3
PICCOLIPIU
ProjectViva
RHEA
Left out study

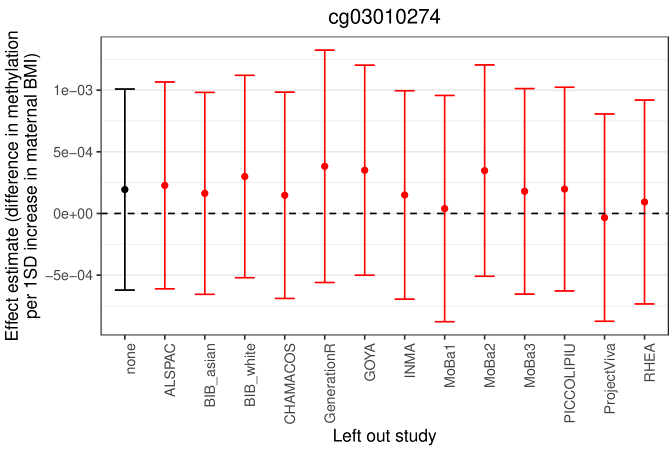
cg03010274
Effect estimate (difference in methylation per 1SD increase in maternal BMI)
1e−03
5e−04
0e+00
−5e−04
none
ALSPAC
BIB_asian
BIB_white
CHAMACOS
GenerationR
GOYA
INMA
MoBa1
MoBa2
MoBa3
PICCOLIPIU
ProjectViva
RHEA
Left out study

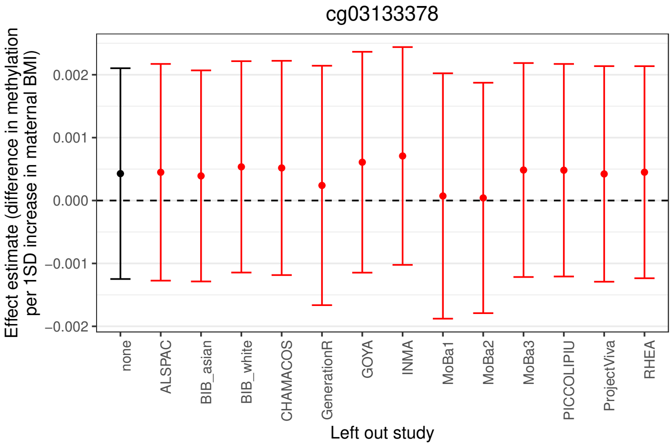
cg03133378
Effect estimate (difference in methylation per 1SD increase in maternal BMI)
0.002
0.001
0.000
−0.001
−0.002
none ALSPAC BIB_asian BIB_white CHAMACOS GenerationR GOYA INMA MoBa1 MoBa2 MoBa3 PICCOLIPIU ProjectViva RHEA
Left out study

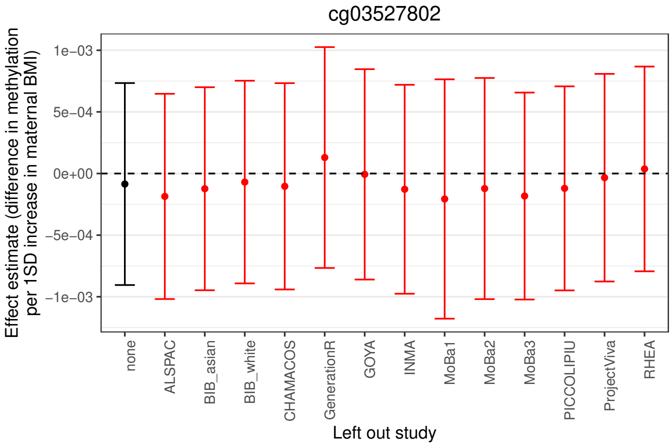
cg03527802
Effect estimate (difference in methylation per 1SD increase in maternal BMI)
1e−03
5e−04
0e+00
−5e−04
−1e−03
none ALSPAC BIB_asian BIB_white CHAMACOS GenerationR GOYA INMA MoBa1 MoBa2 MoBa3 PICCOLIPIU ProjectViva RHEA
Left out study

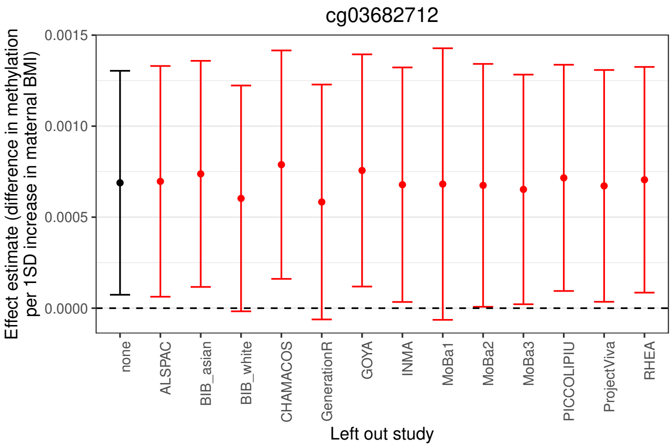
cg03682712
Effect estimate (difference in methylation per 1SD increase in maternal BMI)
0.0015
0.0010
0.0005
0.0000
none ALSPAC BIB_asian BIB_white CHAMACOS GenerationR GOYA INMA MoBa1 MoBa2 MoBa3 PICCOLIPIU ProjectViva RHEA
Left out study

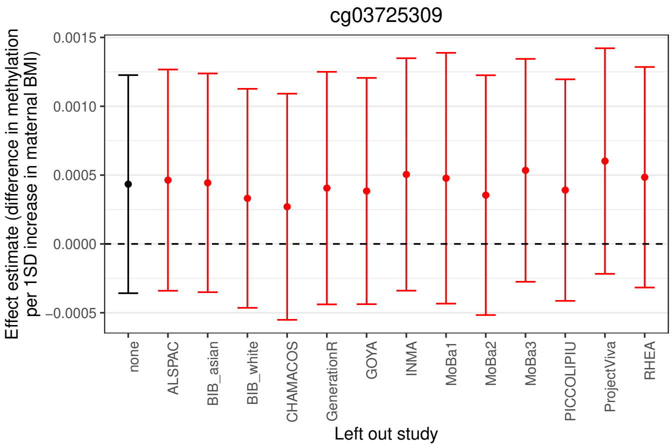
cg03725309
Effect estimate (difference in methylation per 1SD increase in maternal BMI)
0.0015
0.0010
0.0005
0.0000
−0.0005
none ALSPAC BIB_asian BIB_white CHAMACOS GenerationR GOYA INMA MoBa1 MoBa2 MoBa3 PICCOLIPIU ProjectViva RHEA
Left out study

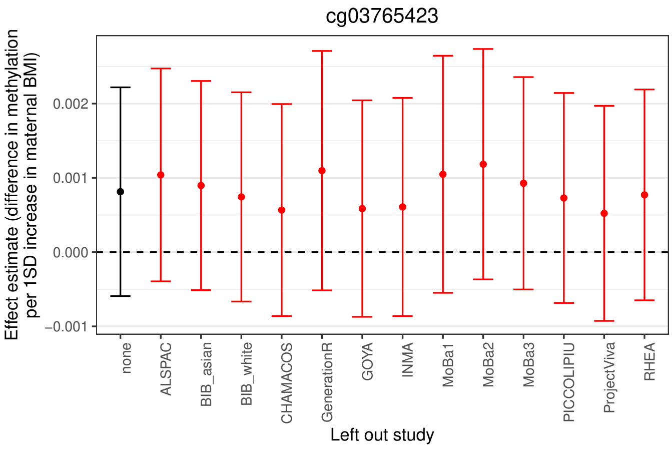
cg03765423
Effect estimate (difference in methylation per 1SD increase in maternal BMI)
0.002
0.001
0.000
−0.001
none ALSPAC BIB_asian BIB_white CHAMACOS GenerationR GOYA INMA MoBa1 MoBa2 MoBa3 PICCOLIPIU ProjectViva RHEA
Left out study

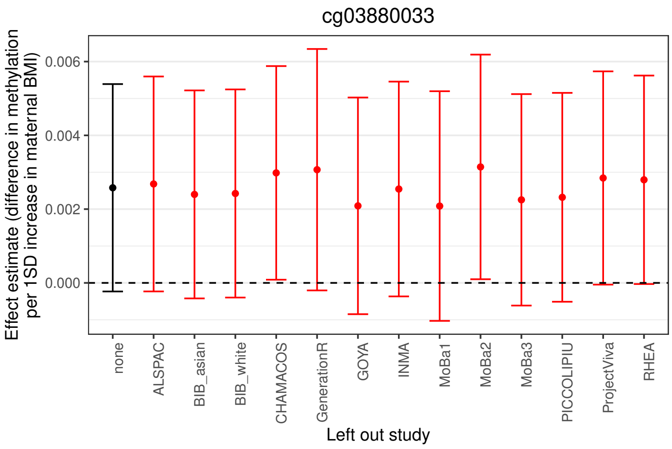
cg03880033
Effect estimate (difference in methylation per 1SD increase in maternal BMI)
0.006
0.004
0.002
0.000
none ALSPAC BIB_asian BIB_white CHAMACOS GenerationR GOYA INMA MoBa1 MoBa2 MoBa3 PICCOLIPIU ProjectViva RHEA
Left out study

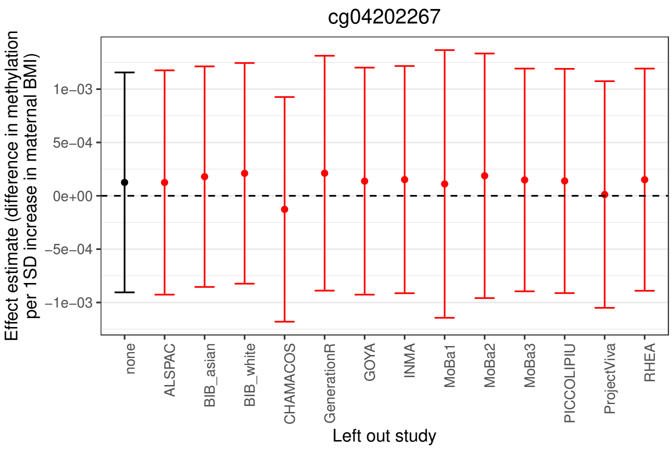
cg04202267
Effect estimate (difference in methylation per 1SD increase in maternal BMI)
1e−03
5e−04
0e+00
−5e−04
−1e−03
none ALSPAC BIB_asian BIB_white CHAMACOS GenerationR GOYA INMA MoBa1 MoBa2 MoBa3 PICCOLIPIU ProjectViva RHEA
Left out study

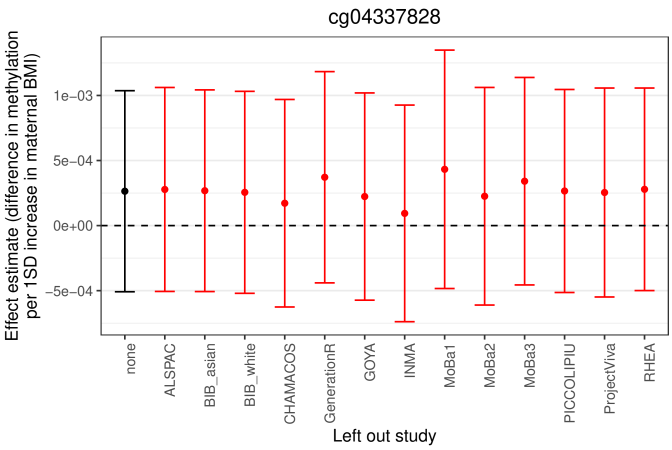
cg04337828
Effect estimate (difference in methylation per 1SD increase in maternal BMI)
1e−03
5e−04
0e+00
−5e−04
none ALSPAC BIB_asian BIB_white CHAMACOS GenerationR GOYA INMA MoBa1 MoBa2 MoBa3 PICCOLIPIU ProjectViva RHEA
Left out study

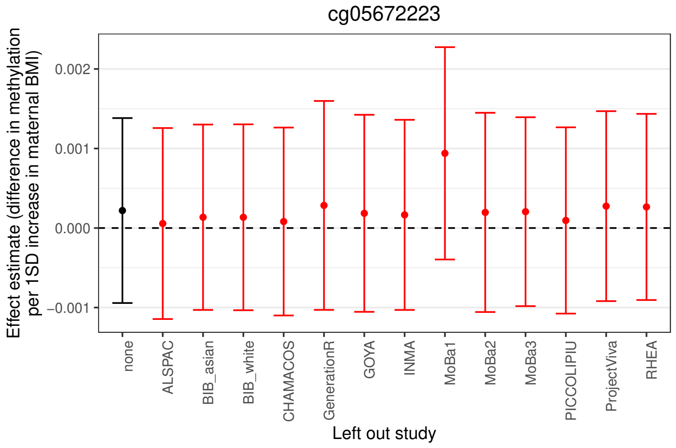
cg05672223
Effect estimate (difference in methylation per 1SD increase in maternal BMI)
0.002
0.001
0.000
−0.001
none
ALSPAC
BIB_asian
BIB_white
CHAMACOS
GenerationR
GOYA
INMA
MoBa1
MoBa2
MoBa3
PICCOLIPIU
ProjectViva
RHEA
Left out study

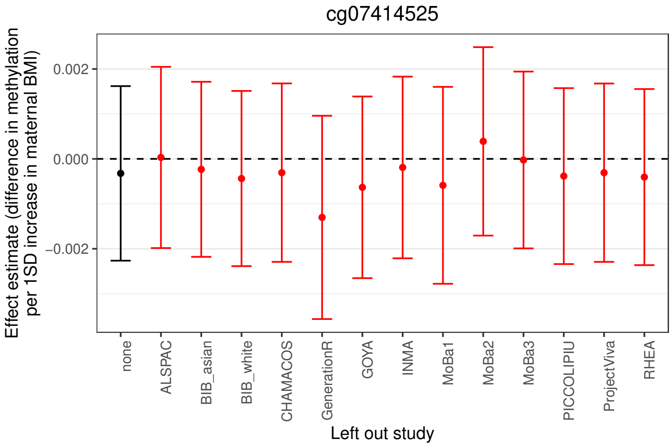
cg07414525
Effect estimate (difference in methylation per 1SD increase in maternal BMI)
0.002
0.000
−0.002
none
ALSPAC
BIB_asian
BIB_white
CHAMACOS
GenerationR
GOYA
INMA
MoBa1
MoBa2
MoBa3
PICCOLIPIU
ProjectViva
RHEA
Left out study

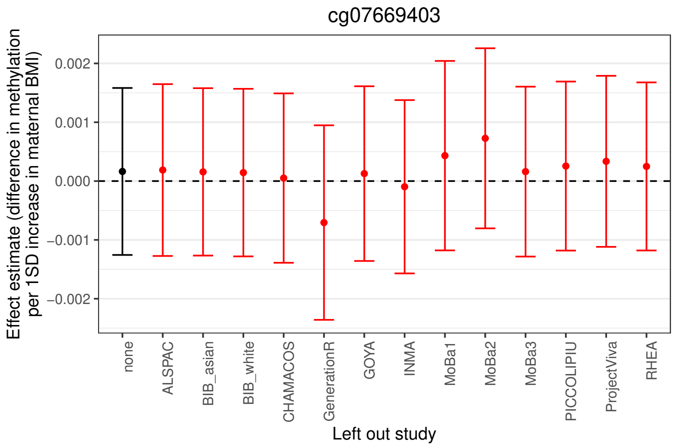
cg07669403
Effect estimate (difference in methylation per 1SD increase in maternal BMI)
0.002
0.001
0.000
−0.001
−0.002
none
ALSPAC
BIB_asian
BIB_white
CHAMACOS
GenerationR
GOYA
INMA
MoBa1
MoBa2
MoBa3
PICCOLIPIU
ProjectViva
RHEA
Left out study

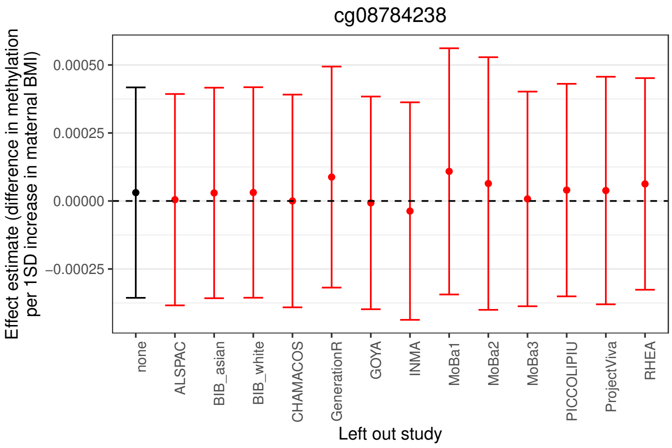
cg08784238
Effect estimate (difference in methylation per 1SD increase in maternal BMI)
0.00050
0.00025
0.00000
−0.00025
none
ALSPAC
BIB_asian
BIB_white
CHAMACOS
GenerationR
GOYA
INMA
MoBa1
MoBa2
MoBa3
PICCOLIPIU
ProjectViva
RHEA
Left out study

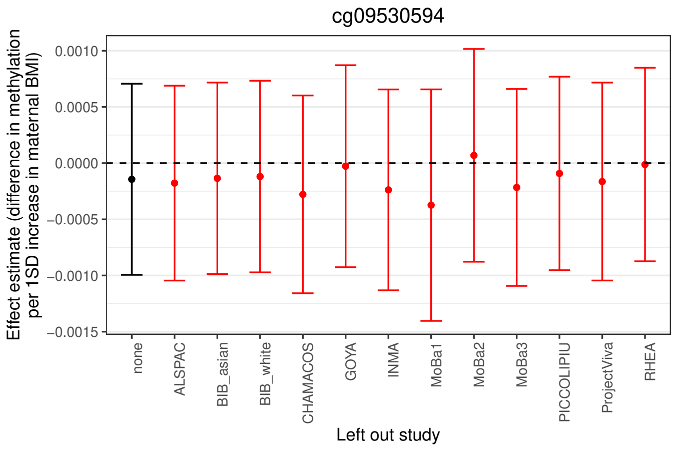
cg09530594
Effect estimate (difference in methylation per 1SD increase in maternal BMI)
0.0010
0.0005
0.0000
−0.0005
−0.0010
−0.0015
none
ALSPAC
BIB_asian
BIB_white
CHAMACOS
GOYA
INMA
MoBa1
MoBa2
MoBa3
PICCOLIPIU
ProjectViva
RHEA
Left out study

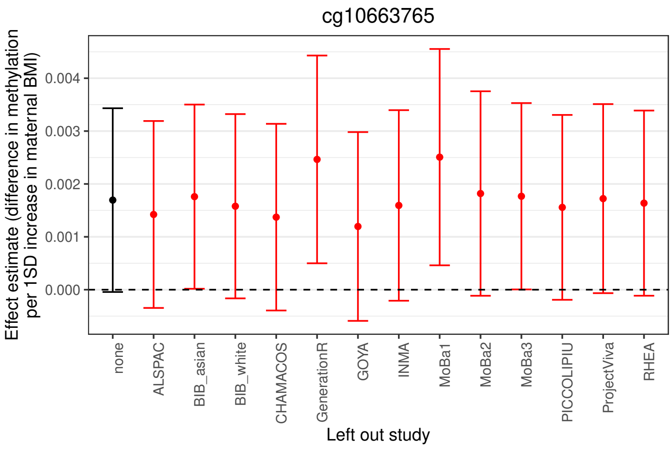
cg10663765
Effect estimate (difference in methylation per 1SD increase in maternal BMI)
0.004
0.003
0.002
0.001
0.000
none
ALSPAC
BIB_asian
BIB_white
CHAMACOS
GenerationR
GOYA
INMA
MoBa1
MoBa2
MoBa3
PICCOLIPIU
ProjectViva
RHEA
Left out study

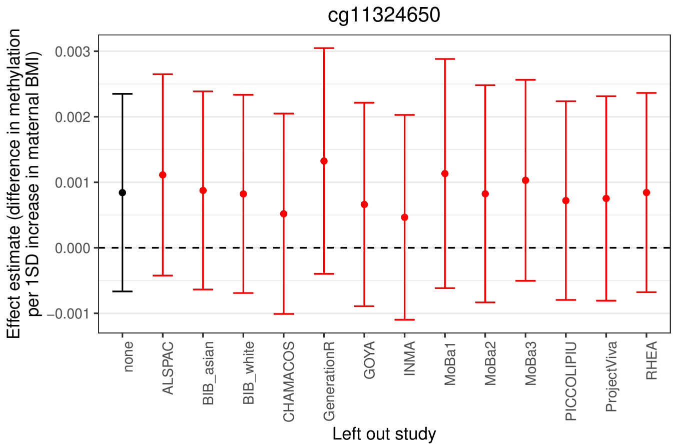
cg11324650
Effect estimate (difference in methylation per 1SD increase in maternal BMI)
0.003
0.002
0.001
0.000
−0.001
none
ALSPAC
BIB_asian
BIB_white
CHAMACOS
GenerationR
GOYA
INMA
MoBa1
MoBa2
MoBa3
PICCOLIPIU
ProjectViva
RHEA
Left out study

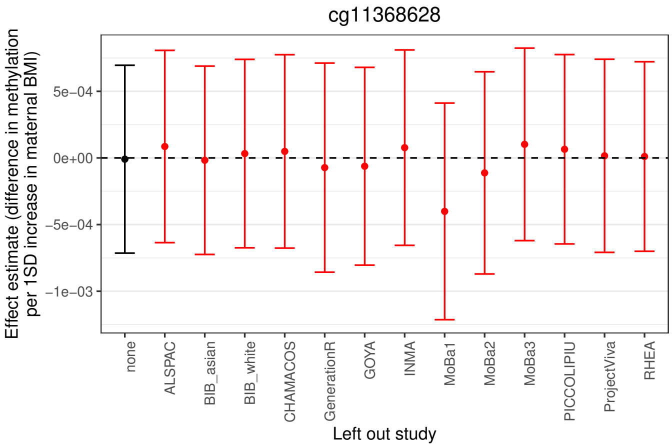
cg11368628
Effect estimate (difference in methylation per 1SD increase in maternal BMI)
5e−04
0e+00
−5e−04
−1e−03
none
ALSPAC
BIB_asian
BIB_white
CHAMACOS
GenerationR
GOYA
INMA
MoBa1
MoBa2
MoBa3
PICCOLIPIU
ProjectViva
RHEA
Left out study

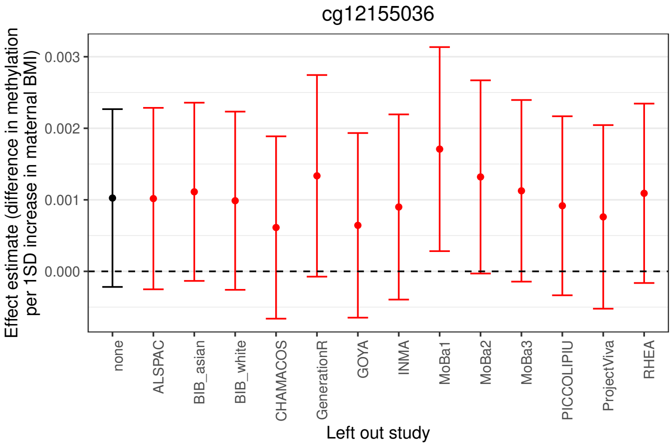
cg12155036
Effect estimate (difference in methylation per 1SD increase in maternal BMI)
0.003
0.002
0.001
0.000
none
ALSPAC
BIB_asian
BIB_white
CHAMACOS
GenerationR
GOYA
INMA
MoBa1
MoBa2
MoBa3
PICCOLIPIU
ProjectViva
RHEA
Left out study

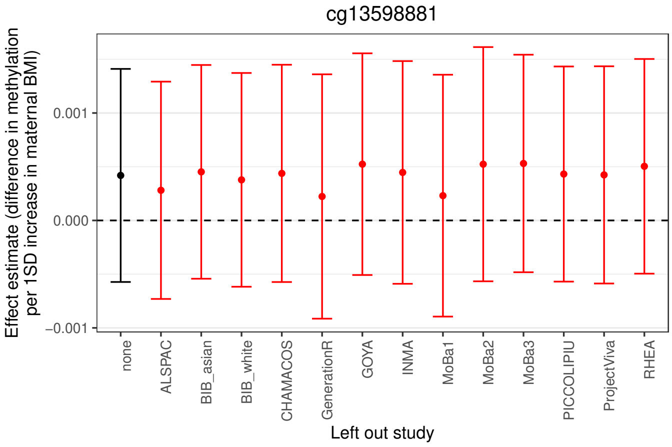
cg13598881
Effect estimate (difference in methylation per 1SD increase in maternal BMI)
0.001
0.000
−0.001
none
ALSPAC
BIB_asian
BIB_white
CHAMACOS
GenerationR
GOYA
INMA
MoBa1
MoBa2
MoBa3
PICCOLIPIU
ProjectViva
RHEA
Left out study

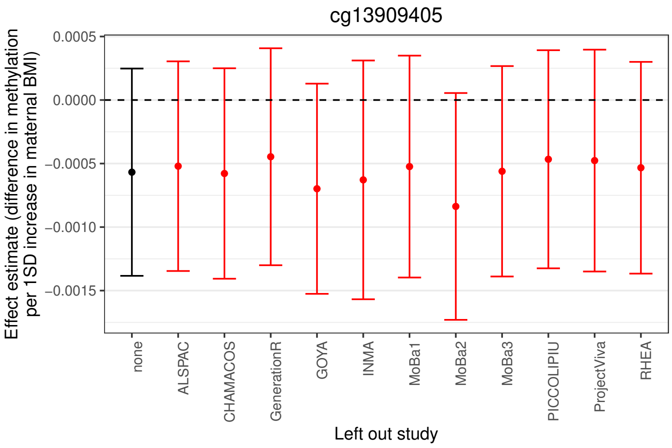
cg13909405
Effect estimate (difference in methylation per 1SD increase in maternal BMI)
0.0005
0.0000
−0.0005
−0.0010
−0.0015
none
ALSPAC
CHAMACOS
GenerationR
GOYA
INMA
MoBa1
MoBa2
MoBa3
PICCOLIPIU
ProjectViva
RHEA
Left out study

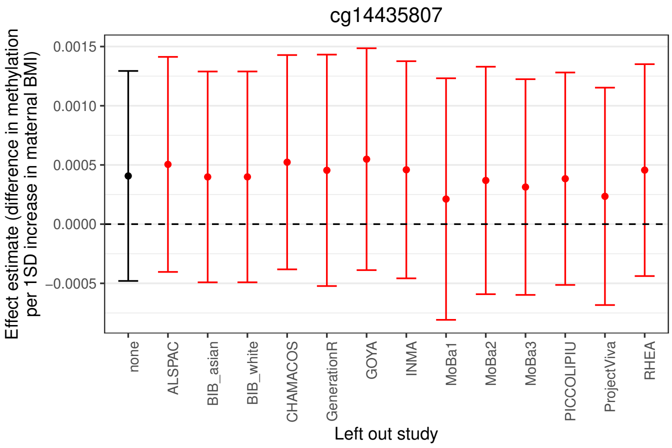
cg14435807
Effect estimate (difference in methylation per 1SD increase in maternal BMI)
0.0015
0.0010
0.0005
0.0000
−0.0005
none
ALSPAC
BIB_asian
BIB_white
CHAMACOS
GenerationR
GOYA
INMA
MoBa1
MoBa2
MoBa3
PICCOLIPIU
ProjectViva
RHEA
Left out study

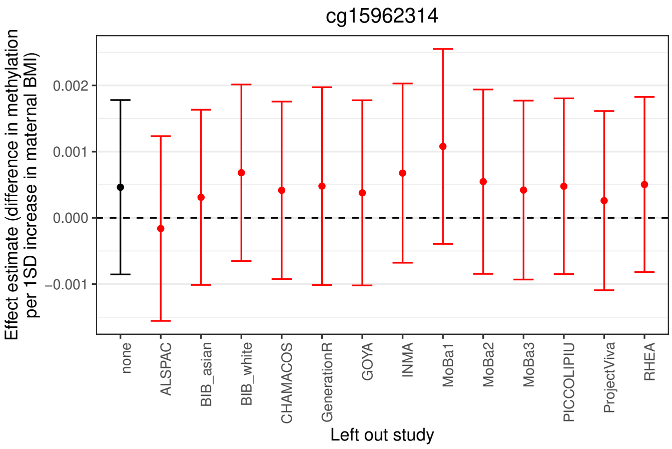
cg15962314
Effect estimate (difference in methylation per 1SD increase in maternal BMI)
0.002
0.001
0.000
−0.001
none
ALSPAC
BIB_asian
BIB_white
CHAMACOS
GenerationR
GOYA
INMA
MoBa1
MoBa2
MoBa3
PICCOLIPIU
ProjectViva
RHEA
Left out study

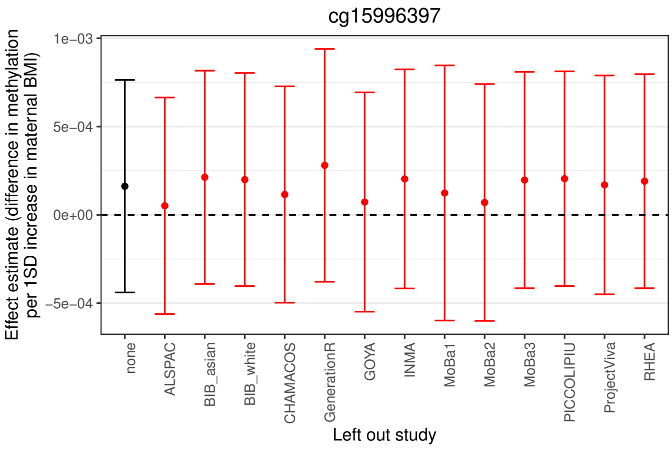
cg15996397
Effect estimate (difference in methylation per 1SD increase in maternal BMI)
1e−03
5e−04
0e+00
−5e−04
none
ALSPAC
BIB_asian
BIB_white
CHAMACOS
GenerationR
GOYA
INMA
MoBa1
MoBa2
MoBa3
PICCOLIPIU
ProjectViva
RHEA
Left out study

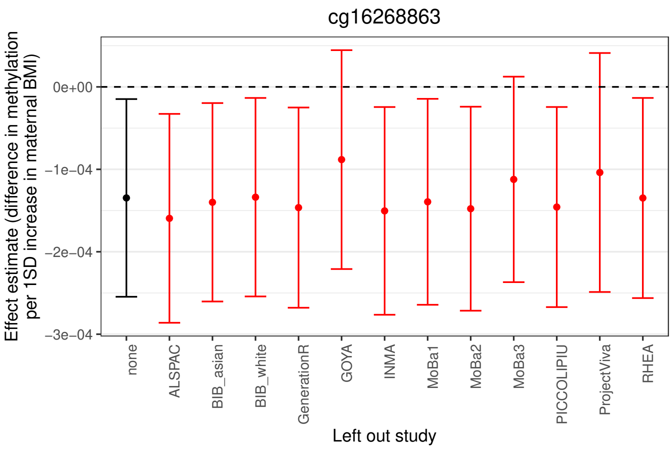
cg16268863
Effect estimate (difference in methylation per 1SD increase in maternal BMI)
0e+00
−1e−04
−2e−04
−3e−04
none
ALSPAC
BIB_asian
BIB_white
GenerationR
GOYA
INMA
MoBa1
MoBa2
MoBa3
PICCOLIPIU
ProjectViva
RHEA
Left out study

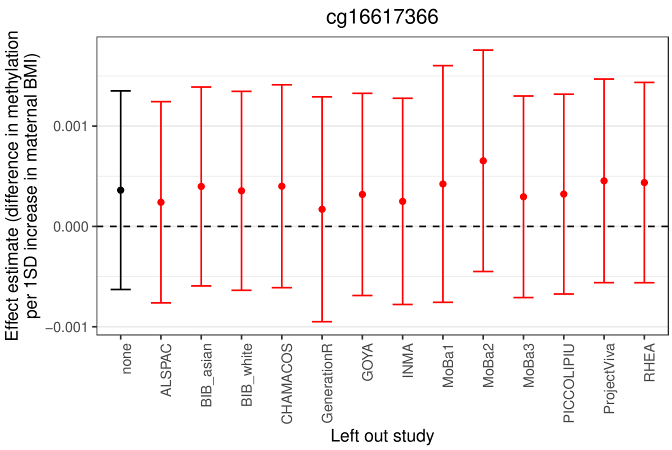
cg16617366
Effect estimate (difference in methylation per 1SD increase in maternal BMI)
0.001
0.000
−0.001
none
ALSPAC
BIB_asian
BIB_white
CHAMACOS
GenerationR
GOYA
INMA
MoBa1
MoBa2
MoBa3
PICCOLIPIU
ProjectViva
RHEA
Left out study

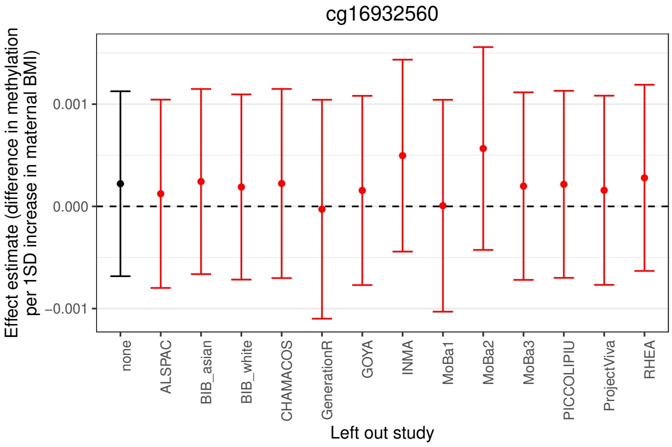
cg16932560
Effect estimate (difference in methylation per 1SD increase in maternal BMI)
0.001
0.000
−0.001
none
ALSPAC
BIB_asian
BIB_white
CHAMACOS
GenerationR
GOYA
INMA
MoBa1
MoBa2
MoBa3
PICCOLIPIU
ProjectViva
RHEA
Left out study

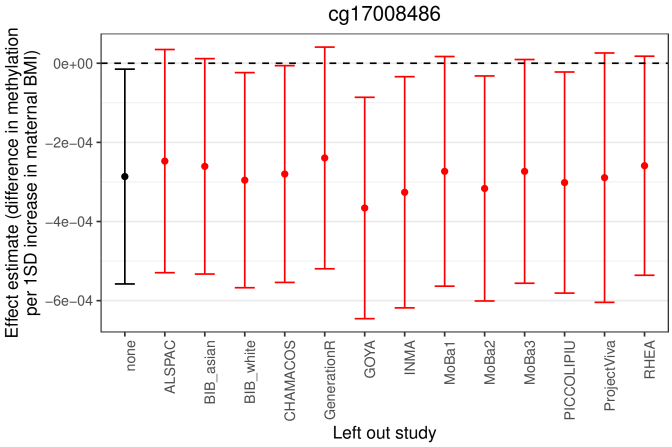
cg17008486
Effect estimate (difference in methylation per 1SD increase in maternal BMI)
0e+00
−2e−04
−4e−04
−6e−04
none
ALSPAC
BIB_asian
BIB_white
CHAMACOS
GenerationR
GOYA
INMA
MoBa1
MoBa2
MoBa3
PICCOLIPIU
ProjectViva
RHEA
Left out study

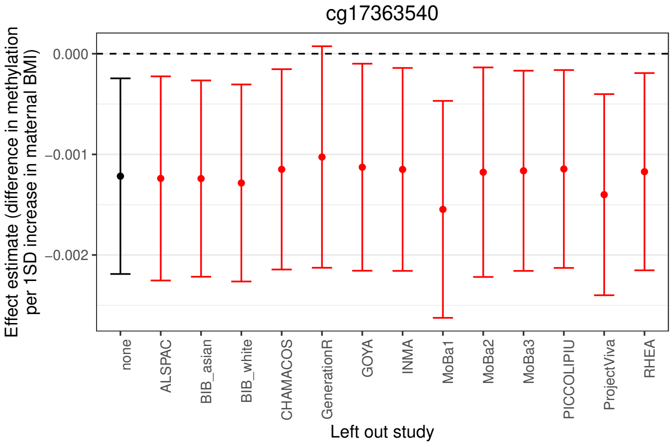
cg17363540
Effect estimate (difference in methylation per 1SD increase in maternal BMI)
0.000
−0.001
−0.002
none
ALSPAC
BIB_asian
BIB_white
CHAMACOS
GenerationR
GOYA
INMA
MoBa1
MoBa2
MoBa3
PICCOLIPIU
ProjectViva
RHEA
Left out study

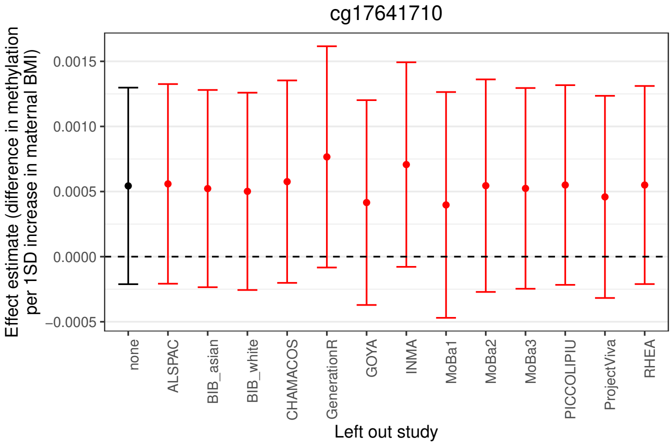
cg17641710
Effect estimate (difference in methylation per 1SD increase in maternal BMI)
0.0015
0.0010
0.0005
0.0000
−0.0005
none
ALSPAC
BIB_asian
BIB_white
CHAMACOS
GenerationR
GOYA
INMA
MoBa1
MoBa2
MoBa3
PICCOLIPIU
ProjectViva
RHEA
Left out study

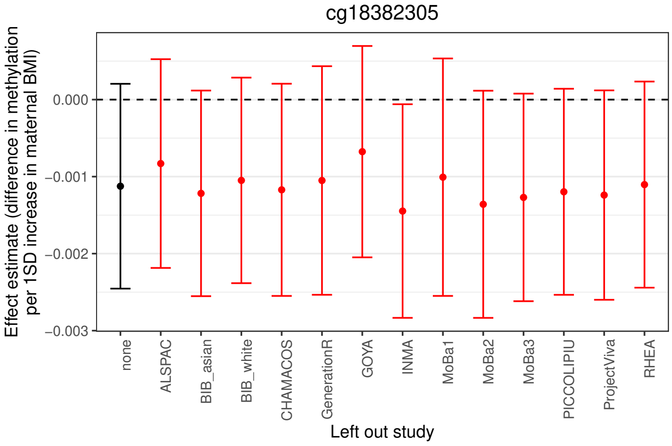
cg18382305
Effect estimate (difference in methylation per 1SD increase in maternal BMI)
0.000
−0.001
−0.002
−0.003
none
ALSPAC
BIB_asian
BIB_white
CHAMACOS
GenerationR
GOYA
INMA
MoBa1
MoBa2
MoBa3
PICCOLIPIU
ProjectViva
RHEA
Left out study

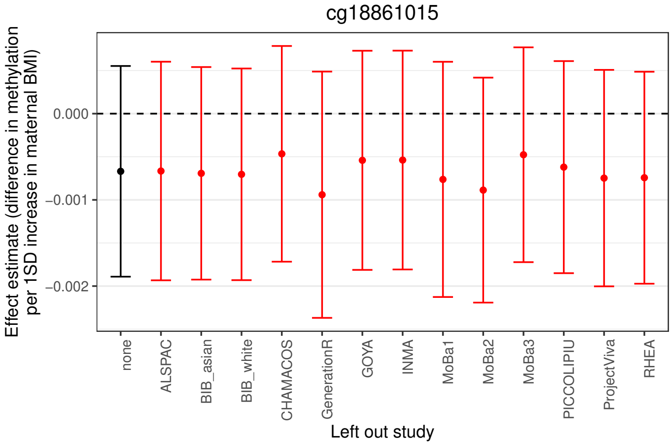
cg18861015
Effect estimate (difference in methylation per 1SD increase in maternal BMI)
0.000
−0.001
−0.002
none
ALSPAC
BIB_asian
BIB_white
CHAMACOS
GenerationR
GOYA
INMA
MoBa1
MoBa2
MoBa3
PICCOLIPIU
ProjectViva
RHEA
Left out study

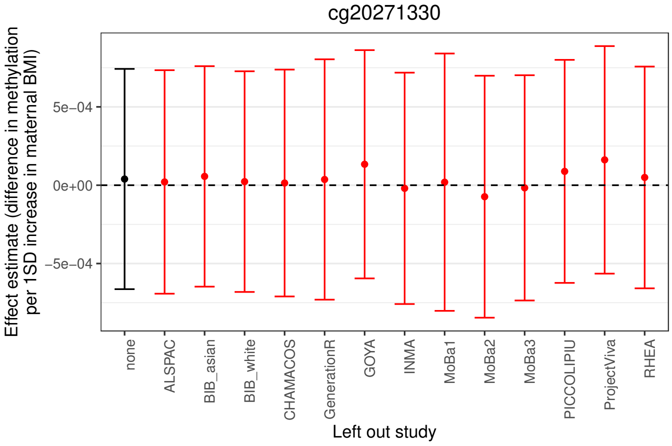
cg20271330
Effect estimate (difference in methylation per 1SD increase in maternal BMI)
5e−04
0e+00
−5e−04
none
ALSPAC
BIB_asian
BIB_white
CHAMACOS
GenerationR
GOYA
INMA
MoBa1
MoBa2
MoBa3
PICCOLIPIU
ProjectViva
RHEA
Left out study

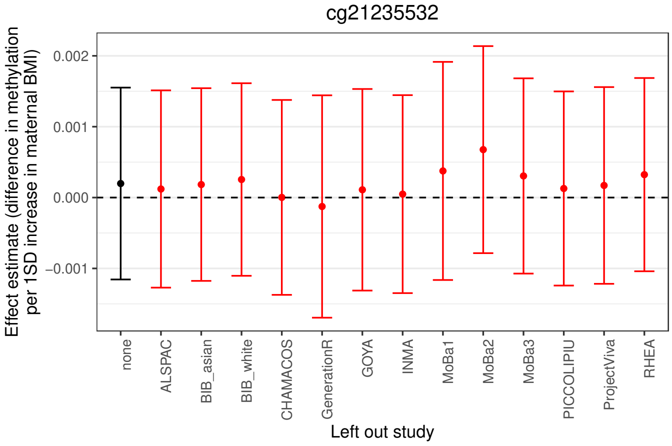
cg21235532
Effect estimate (difference in methylation per 1SD increase in maternal BMI)
0.002
0.001
0.000
−0.001
none
ALSPAC
BIB_asian
BIB_white
CHAMACOS
GenerationR
GOYA
INMA
MoBa1
MoBa2
MoBa3
PICCOLIPIU
ProjectViva
RHEA
Left out study

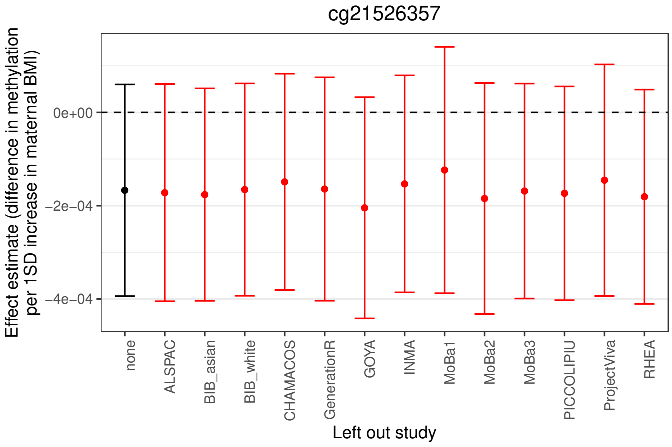
cg21526357
Effect estimate (difference in methylation per 1SD increase in maternal BMI)
0e+00
−2e−04
−4e−04
none
ALSPAC
BIB_asian
BIB_white
CHAMACOS
GenerationR
GOYA
INMA
MoBa1
MoBa2
MoBa3
PICCOLIPIU
ProjectViva
RHEA
Left out study

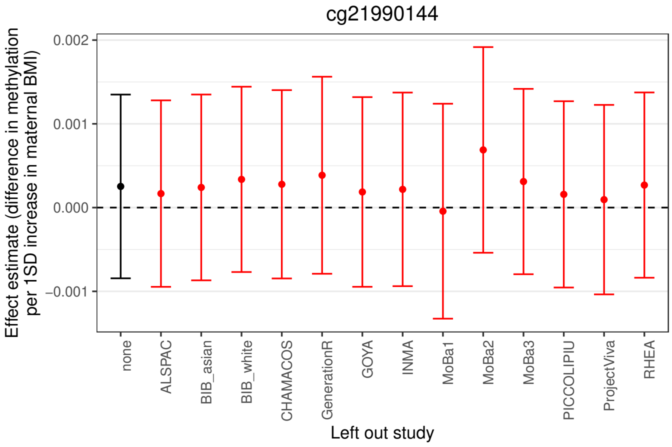
cg21990144
Effect estimate (difference in methylation per 1SD increase in maternal BMI)
0.002
0.001
0.000
−0.001
none
ALSPAC
BIB_asian
BIB_white
CHAMACOS
GenerationR
GOYA
INMA
MoBa1
MoBa2
MoBa3
PICCOLIPIU
ProjectViva
RHEA
Left out study

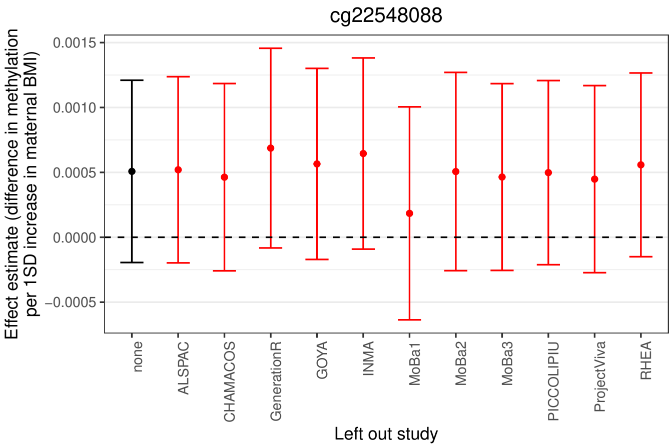
cg22548088
Effect estimate (difference in methylation per 1SD increase in maternal BMI)
0.0015
0.0010
0.0005
0.0000
−0.0005
none
ALSPAC
CHAMACOS
GenerationR
GOYA
INMA
MoBa1
MoBa2
MoBa3
PICCOLIPIU
ProjectViva
RHEA
Left out study

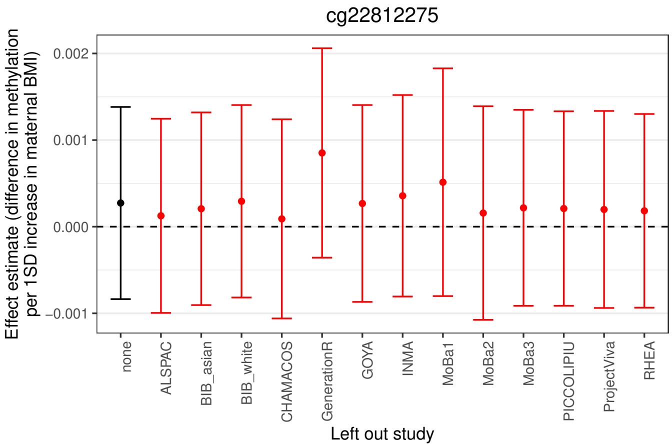
cg22812275
Effect estimate (difference in methylation per 1SD increase in maternal BMI)
0.002
0.001
0.000
−0.001
none
ALSPAC
BIB_asian
BIB_white
CHAMACOS
GenerationR
GOYA
INMA
MoBa1
MoBa2
MoBa3
PICCOLIPIU
ProjectViva
RHEA
Left out study

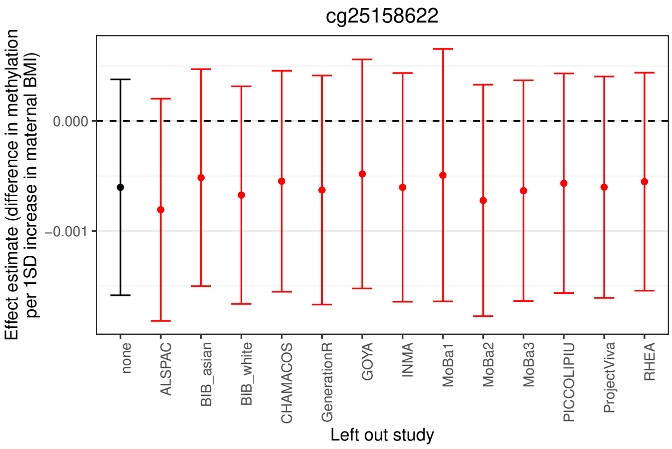
cg25158622
Effect estimate (difference in methylation per 1SD increase in maternal BMI)
0.000
−0.001
none
ALSPAC
BIB_asian
BIB_white
CHAMACOS
GenerationR
GOYA
INMA
MoBa1
MoBa2
MoBa3
PICCOLIPIU
ProjectViva
RHEA
Left out study

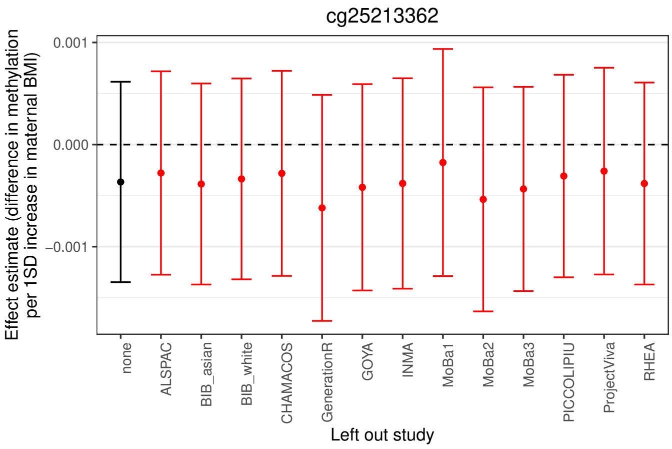
cg25213362
Effect estimate (difference in methylation per 1SD increase in maternal BMI)
0.001
0.000
−0.001
none
ALSPAC
BIB_asian
BIB_white
CHAMACOS
GenerationR
GOYA
INMA
MoBa1
MoBa2
MoBa3
PICCOLIPIU
ProjectViva
RHEA
Left out study

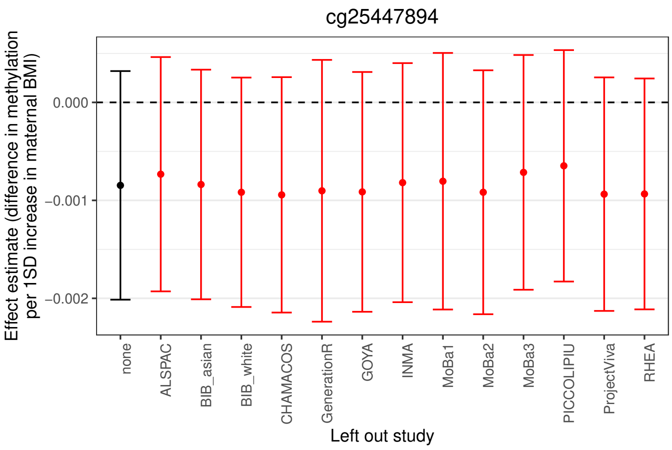
cg25447894
Effect estimate (difference in methylation per 1SD increase in maternal BMI)
0.000
−0.001
−0.002
none
ALSPAC
BIB_asian
BIB_white
CHAMACOS
GenerationR
GOYA
INMA
MoBa1
MoBa2
MoBa3
PICCOLIPIU
ProjectViva
RHEA
Left out study

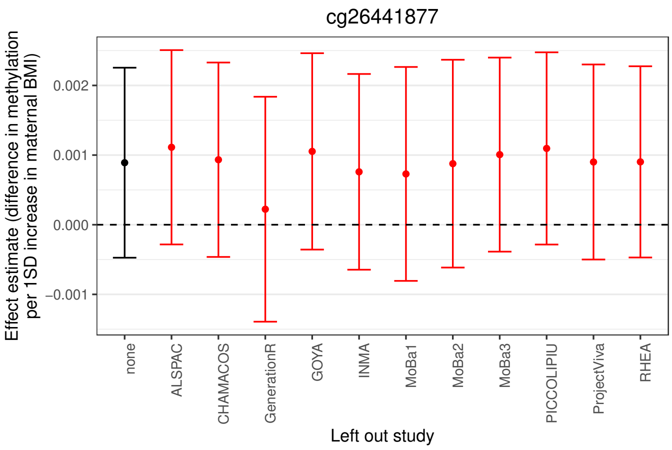
cg26441877
Effect estimate (difference in methylation per 1SD increase in maternal BMI)
0.002
0.001
0.000
−0.001
none
ALSPAC
CHAMACOS
GenerationR
GOYA
INMA
MoBa1
MoBa2
MoBa3
PICCOLIPIU
ProjectViva
RHEA
Left out study

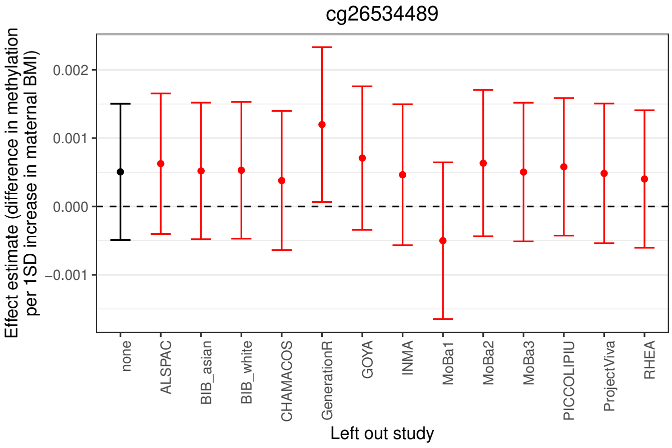
cg26534489
Effect estimate (difference in methylation per 1SD increase in maternal BMI)
0.002
0.001
0.000
−0.001
none
ALSPAC
BIB_asian
BIB_white
CHAMACOS
GenerationR
GOYA
INMA
MoBa1
MoBa2
MoBa3
PICCOLIPIU
ProjectViva
RHEA
Left out study

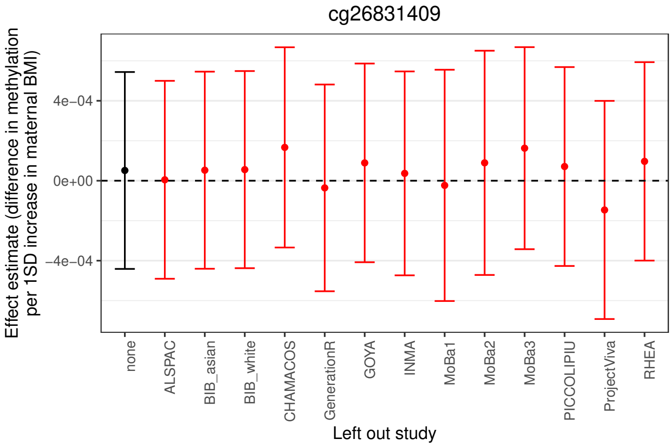
cg26831409
Effect estimate (difference in methylation per 1SD increase in maternal BMI)
4e−04
0e+00
−4e−04
none
ALSPAC
BIB_asian
BIB_white
CHAMACOS
GenerationR
GOYA
INMA
MoBa1
MoBa2
MoBa3
PICCOLIPIU
ProjectViva
RHEA
Left out study

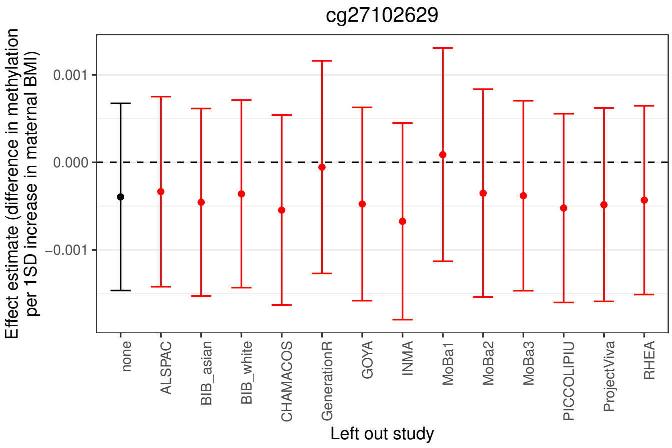
cg27102629
Effect estimate (difference in methylation per 1SD increase in maternal BMI)
0.001
0.000
−0.001
none
ALSPAC
BIB_asian
BIB_white
CHAMACOS
GenerationR
GOYA
INMA
MoBa1
MoBa2
MoBa3
PICCOLIPIU
ProjectViva
RHEA
Left out study

Maternal BMI (adjusted for paternal BMI): pages 22-27

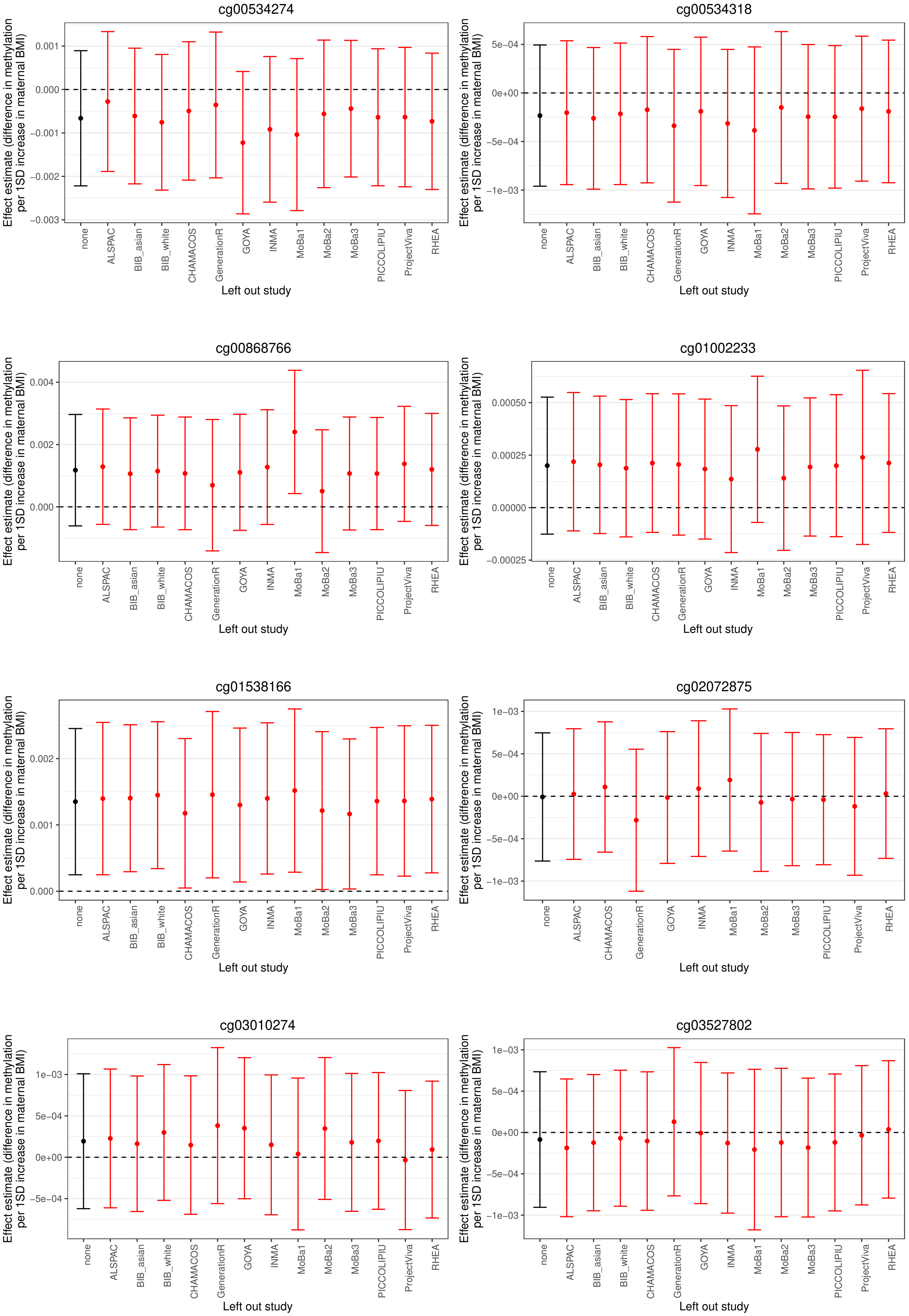
cg00534274
Effect estimate (difference in methylation per 1SD increase in maternal BMI)
0.001
0.000
−0.001
−0.002
−0.003
none
ALSPAC
BIB_asian
BIB_white
CHAMACOS
GenerationR
GOYA
INMA
MoBa1
MoBa2
MoBa3
PICCOLIPIU
ProjectViva
RHEA
Left out study
cg00534318
5e−04
0e+00
−5e−04
−1e−03
cg00868766
0.004
0.002
0.000
cg01002233
0.00050
0.00025
0.00000
−0.00025
cg01538166
0.002
0.001
0.000
cg02072875
1e−03
5e−04
0e+00
−5e−04
−1e−03
cg03010274
1e−03
5e−04
0e+00
−5e−04
cg03527802
1e−03
5e−04
0e+00
−5e−04
−1e−03

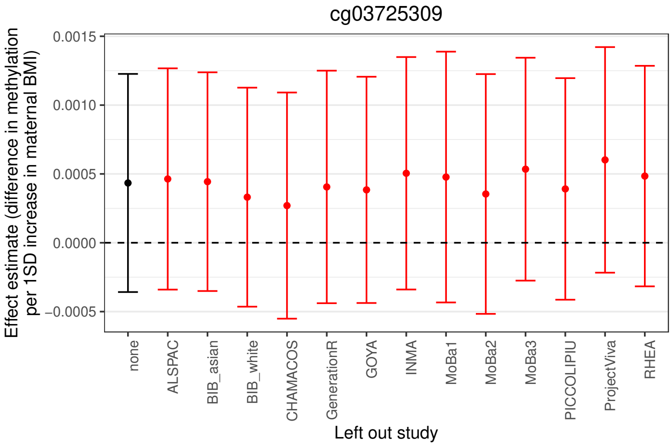
cg03725309
Effect estimate (difference in methylation per 1SD increase in maternal BMI)
0.0015
0.0010
0.0005
0.0000
−0.0005
none
ALSPAC
BIB_asian
BIB_white
CHAMACOS
GenerationR
GOYA
INMA
MoBa1
MoBa2
MoBa3
PICCOLIPIU
ProjectViva
RHEA
Left out study

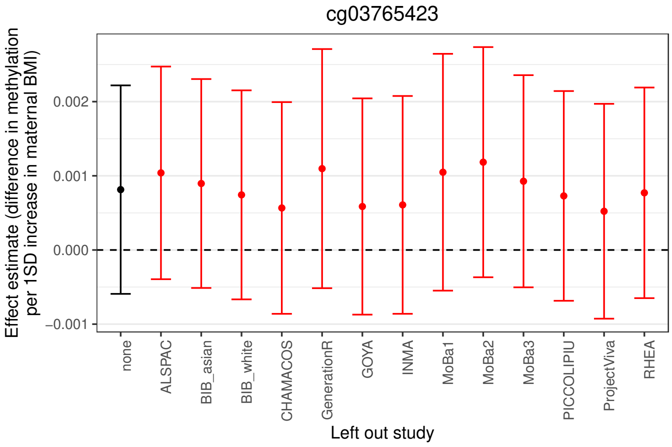
cg03765423
Effect estimate (difference in methylation per 1SD increase in maternal BMI)
0.002
0.001
0.000
−0.001
none
ALSPAC
BIB_asian
BIB_white
CHAMACOS
GenerationR
GOYA
INMA
MoBa1
MoBa2
MoBa3
PICCOLIPIU
ProjectViva
RHEA
Left out study

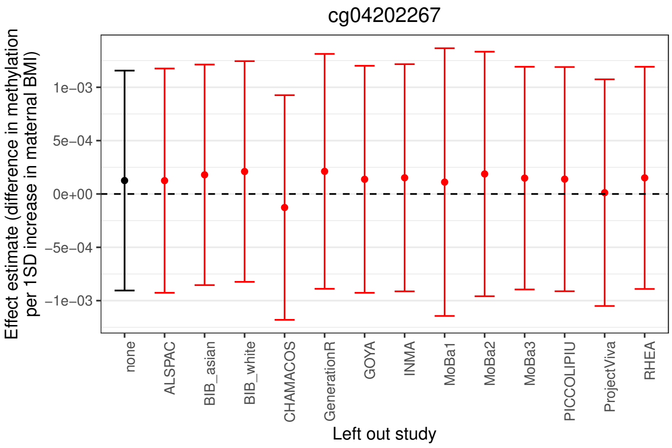
cg04202267
Effect estimate (difference in methylation per 1SD increase in maternal BMI)
1e−03
5e−04
0e+00
−5e−04
−1e−03
none
ALSPAC
BIB_asian
BIB_white
CHAMACOS
GenerationR
GOYA
INMA
MoBa1
MoBa2
MoBa3
PICCOLIPIU
ProjectViva
RHEA
Left out study

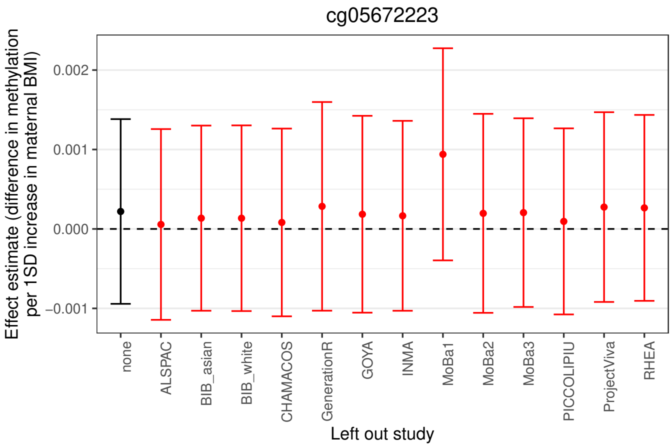
cg05672223
Effect estimate (difference in methylation per 1SD increase in maternal BMI)
0.002
0.001
0.000
−0.001
none
ALSPAC
BIB_asian
BIB_white
CHAMACOS
GenerationR
GOYA
INMA
MoBa1
MoBa2
MoBa3
PICCOLIPIU
ProjectViva
RHEA
Left out study

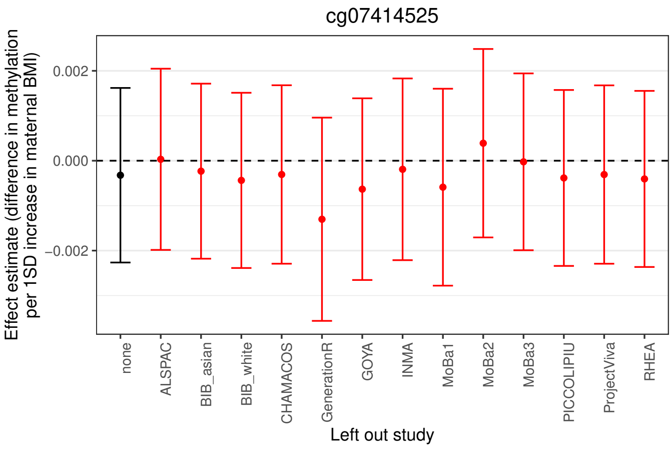
cg07414525
Effect estimate (difference in methylation per 1SD increase in maternal BMI)
0.002
0.000
−0.002
none
ALSPAC
BIB_asian
BIB_white
CHAMACOS
GenerationR
GOYA
INMA
MoBa1
MoBa2
MoBa3
PICCOLIPIU
ProjectViva
RHEA
Left out study

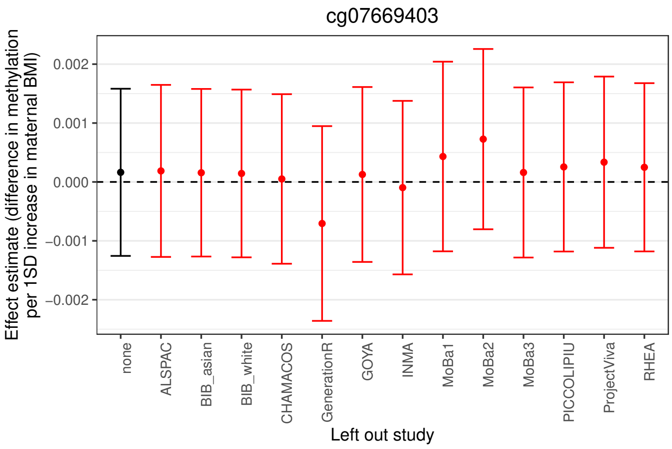
cg07669403
Effect estimate (difference in methylation per 1SD increase in maternal BMI)
0.002
0.001
0.000
−0.001
−0.002
none
ALSPAC
BIB_asian
BIB_white
CHAMACOS
GenerationR
GOYA
INMA
MoBa1
MoBa2
MoBa3
PICCOLIPIU
ProjectViva
RHEA
Left out study

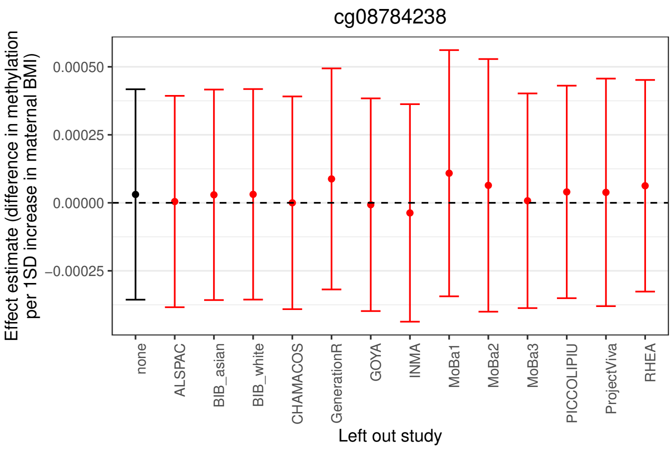
cg08784238
Effect estimate (difference in methylation per 1SD increase in maternal BMI)
0.00050
0.00025
0.00000
−0.00025
none
ALSPAC
BIB_asian
BIB_white
CHAMACOS
GenerationR
GOYA
INMA
MoBa1
MoBa2
MoBa3
PICCOLIPIU
ProjectViva
RHEA
Left out study

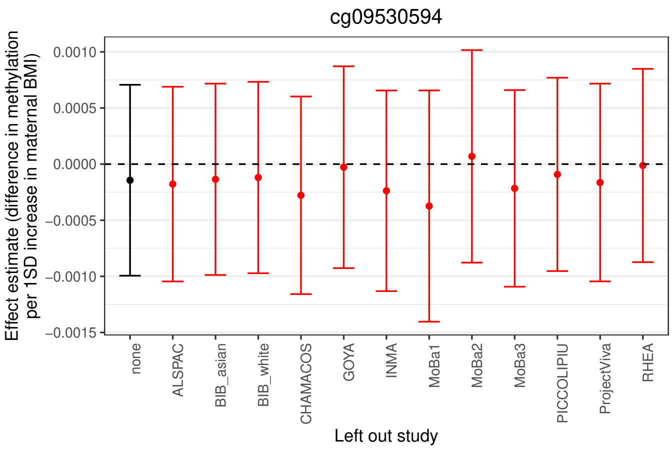
cg09530594
Effect estimate (difference in methylation per 1SD increase in maternal BMI)
0.0010
0.0005
0.0000
−0.0005
−0.0010
−0.0015
none
ALSPAC
BIB_asian
BIB_white
CHAMACOS
GOYA
INMA
MoBa1
MoBa2
MoBa3
PICCOLIPIU
ProjectViva
RHEA
Left out study

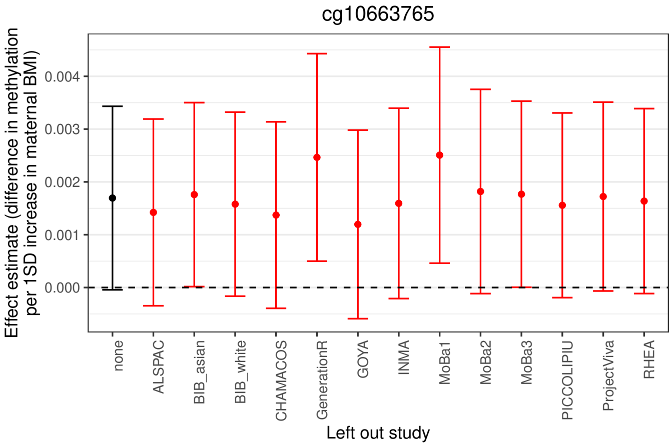
cg10663765
Effect estimate (difference in methylation per 1SD increase in maternal BMI)
0.004
0.003
0.002
0.001
0.000
none
ALSPAC
BIB_asian
BIB_white
CHAMACOS
GenerationR
GOYA
INMA
MoBa1
MoBa2
MoBa3
PICCOLIPIU
ProjectViva
RHEA
Left out study

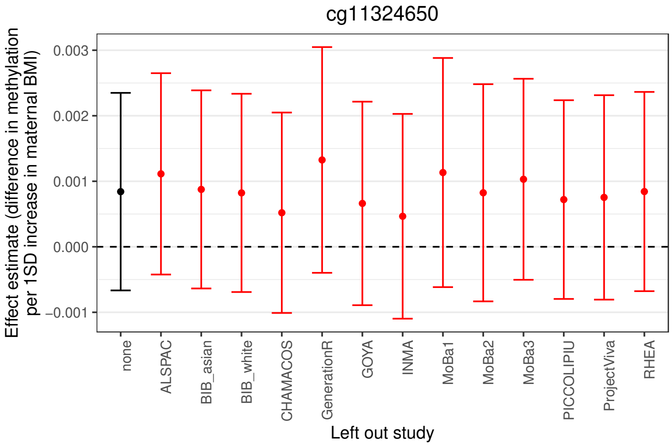
cg11324650
Effect estimate (difference in methylation per 1SD increase in maternal BMI)
0.003
0.002
0.001
0.000
−0.001
none
ALSPAC
BIB_asian
BIB_white
CHAMACOS
GenerationR
GOYA
INMA
MoBa1
MoBa2
MoBa3
PICCOLIPIU
ProjectViva
RHEA
Left out study

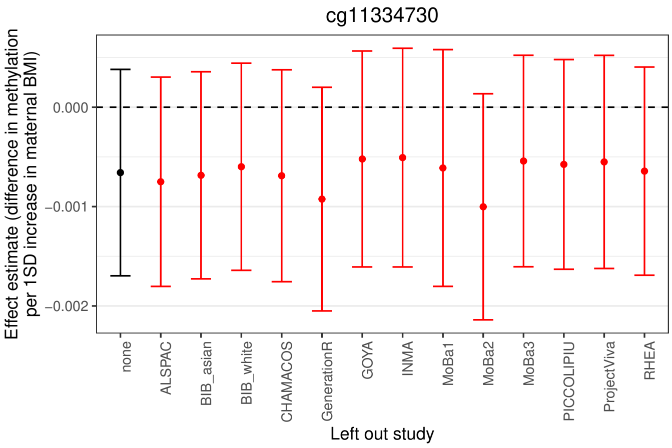
cg11334730
Effect estimate (difference in methylation per 1SD increase in maternal BMI)
0.000
−0.001
−0.002
none
ALSPAC
BIB_asian
BIB_white
CHAMACOS
GenerationR
GOYA
INMA
MoBa1
MoBa2
MoBa3
PICCOLIPIU
ProjectViva
RHEA
Left out study

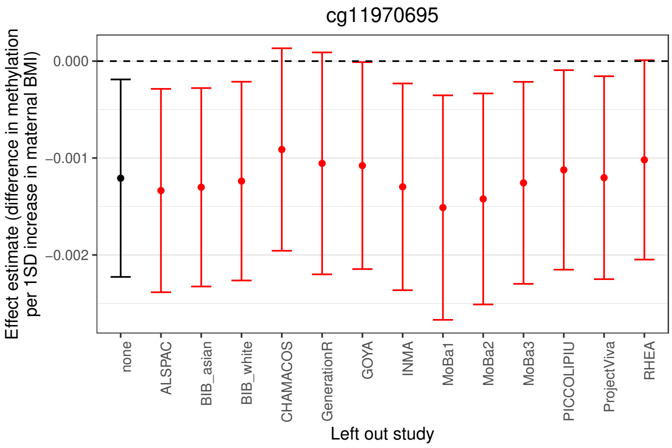
cg11970695
Effect estimate (difference in methylation per 1SD increase in maternal BMI)
0.000
−0.001
−0.002
none
ALSPAC
BIB_asian
BIB_white
CHAMACOS
GenerationR
GOYA
INMA
MoBa1
MoBa2
MoBa3
PICCOLIPIU
ProjectViva
RHEA
Left out study

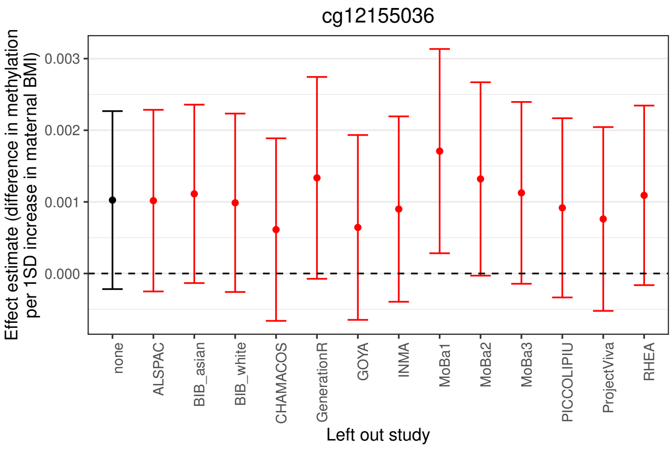
cg12155036
Effect estimate (difference in methylation per 1SD increase in maternal BMI)
0.003
0.002
0.001
0.000
none
ALSPAC
BIB_asian
BIB_white
CHAMACOS
GenerationR
GOYA
INMA
MoBa1
MoBa2
MoBa3
PICCOLIPIU
ProjectViva
RHEA
Left out study

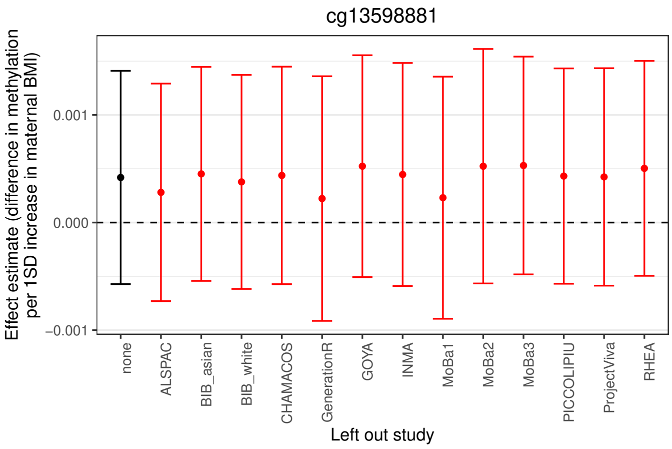
cg13598881
Effect estimate (difference in methylation per 1SD increase in maternal BMI)
0.001
0.000
−0.001
none
ALSPAC
BIB_asian
BIB_white
CHAMACOS
GenerationR
GOYA
INMA
MoBa1
MoBa2
MoBa3
PICCOLIPIU
ProjectViva
RHEA
Left out study

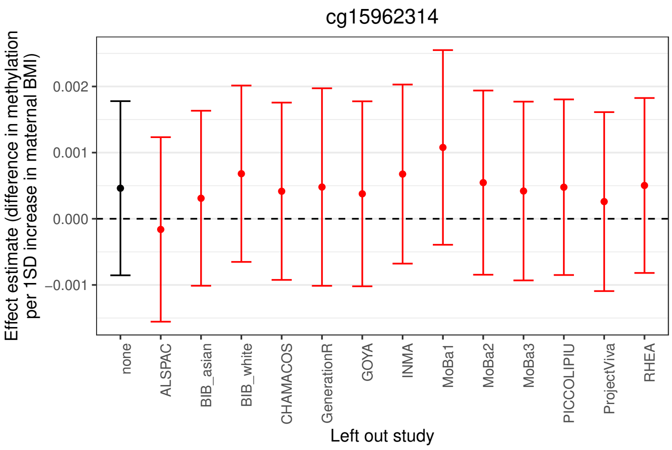
cg15962314
Effect estimate (difference in methylation per 1SD increase in maternal BMI)
0.002
0.001
0.000
−0.001
none
ALSPAC
BIB_asian
BIB_white
CHAMACOS
GenerationR
GOYA
INMA
MoBa1
MoBa2
MoBa3
PICCOLIPIU
ProjectViva
RHEA
Left out study

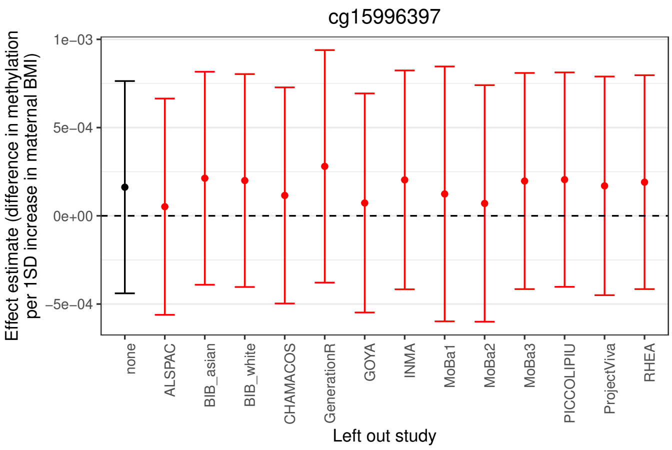
cg15996397
Effect estimate (difference in methylation per 1SD increase in maternal BMI)
1e−03
5e−04
0e+00
−5e−04
none
ALSPAC
BIB_asian
BIB_white
CHAMACOS
GenerationR
GOYA
INMA
MoBa1
MoBa2
MoBa3
PICCOLIPIU
ProjectViva
RHEA
Left out study

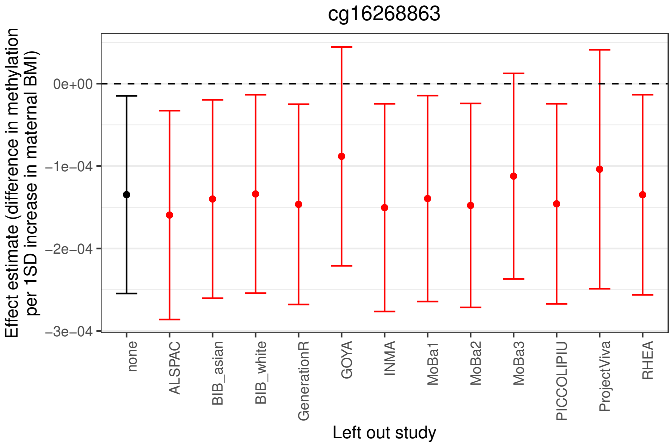
cg16268863
Effect estimate (difference in methylation per 1SD increase in maternal BMI)
0e+00
−1e−04
−2e−04
−3e−04
none
ALSPAC
BIB_asian
BIB_white
GenerationR
GOYA
INMA
MoBa1
MoBa2
MoBa3
PICCOLIPIU
ProjectViva
RHEA
Left out study

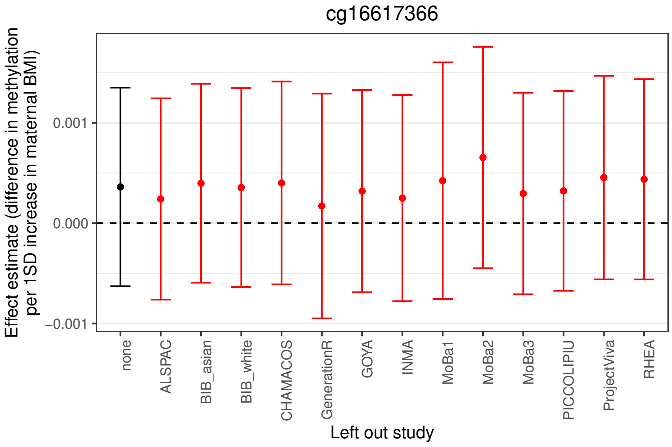
cg16617366
Effect estimate (difference in methylation per 1SD increase in maternal BMI)
0.001
0.000
−0.001
none
ALSPAC
BIB_asian
BIB_white
CHAMACOS
GenerationR
GOYA
INMA
MoBa1
MoBa2
MoBa3
PICCOLIPIU
ProjectViva
RHEA
Left out study

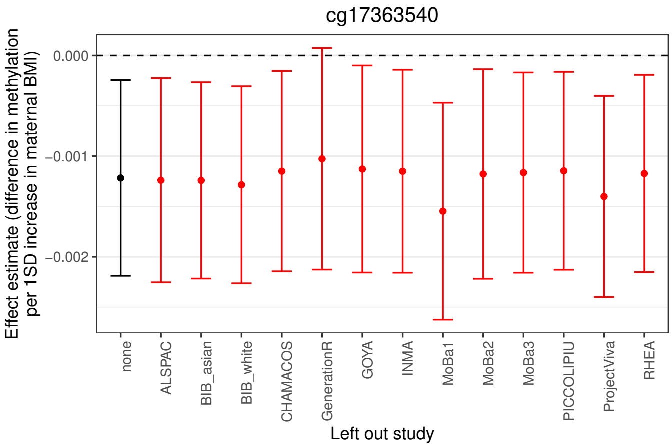
cg17363540
Effect estimate (difference in methylation per 1SD increase in maternal BMI)
0.000
−0.001
−0.002
none
ALSPAC
BIB_asian
BIB_white
CHAMACOS
GenerationR
GOYA
INMA
MoBa1
MoBa2
MoBa3
PICCOLIPIU
ProjectViva
RHEA
Left out study

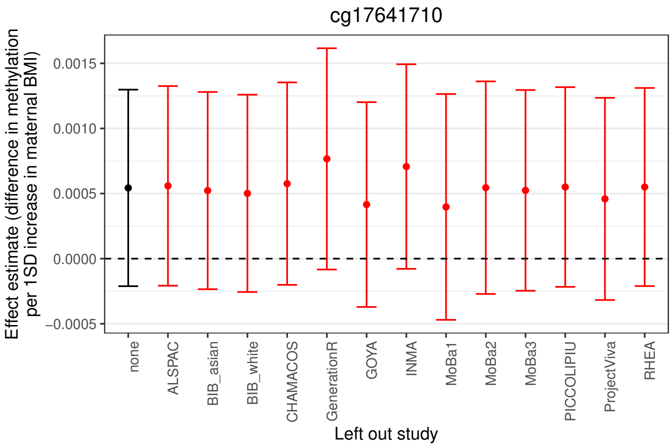
cg17641710
Effect estimate (difference in methylation per 1SD increase in maternal BMI)
0.0015
0.0010
0.0005
0.0000
−0.0005
none
ALSPAC
BIB_asian
BIB_white
CHAMACOS
GenerationR
GOYA
INMA
MoBa1
MoBa2
MoBa3
PICCOLIPIU
ProjectViva
RHEA
Left out study

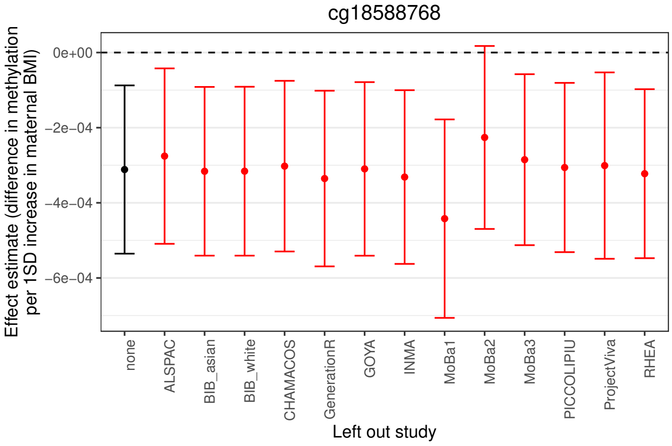
cg18588768
Effect estimate (difference in methylation per 1SD increase in maternal BMI)
0e+00
−2e−04
−4e−04
−6e−04
none
ALSPAC
BIB_asian
BIB_white
CHAMACOS
GenerationR
GOYA
INMA
MoBa1
MoBa2
MoBa3
PICCOLIPIU
ProjectViva
RHEA
Left out study

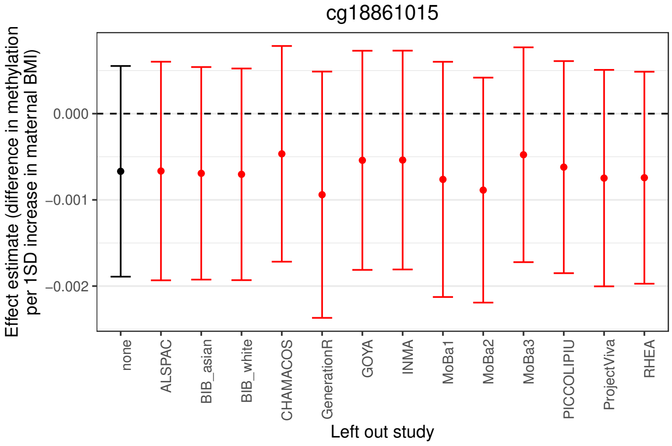
cg18861015
Effect estimate (difference in methylation per 1SD increase in maternal BMI)
0.000
−0.001
−0.002
none
ALSPAC
BIB_asian
BIB_white
CHAMACOS
GenerationR
GOYA
INMA
MoBa1
MoBa2
MoBa3
PICCOLIPIU
ProjectViva
RHEA
Left out study

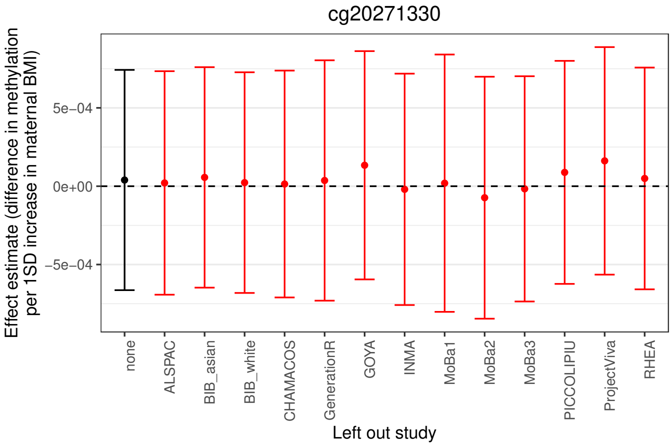
cg20271330
Effect estimate (difference in methylation per 1SD increase in maternal BMI)
5e−04
0e+00
−5e−04
none
ALSPAC
BIB_asian
BIB_white
CHAMACOS
GenerationR
GOYA
INMA
MoBa1
MoBa2
MoBa3
PICCOLIPIU
ProjectViva
RHEA
Left out study

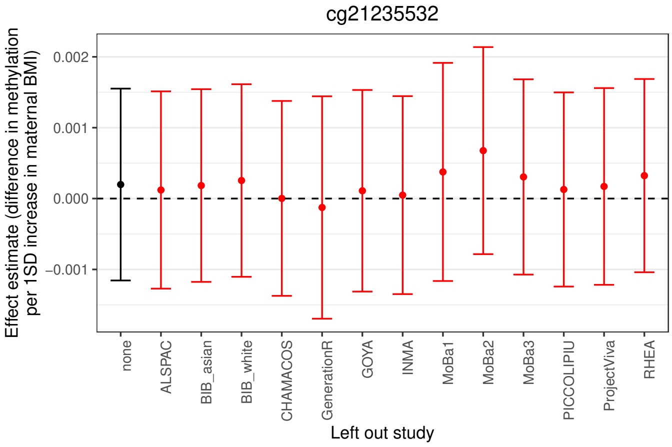
cg21235532
Effect estimate (difference in methylation per 1SD increase in maternal BMI)
0.002
0.001
0.000
−0.001
none
ALSPAC
BIB_asian
BIB_white
CHAMACOS
GenerationR
GOYA
INMA
MoBa1
MoBa2
MoBa3
PICCOLIPIU
ProjectViva
RHEA
Left out study

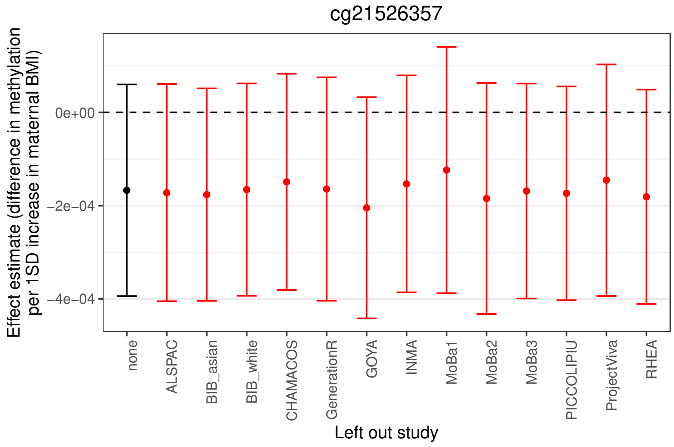
cg21526357
Effect estimate (difference in methylation per 1SD increase in maternal BMI)
0e+00
−2e−04
−4e−04
none
ALSPAC
BIB_asian
BIB_white
CHAMACOS
GenerationR
GOYA
INMA
MoBa1
MoBa2
MoBa3
PICCOLIPIU
ProjectViva
RHEA
Left out study

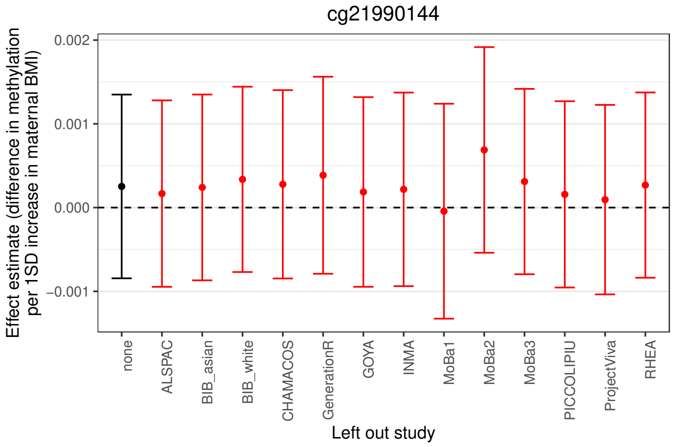
cg21990144
Effect estimate (difference in methylation per 1SD increase in maternal BMI)
0.002
0.001
0.000
−0.001
none
ALSPAC
BIB_asian
BIB_white
CHAMACOS
GenerationR
GOYA
INMA
MoBa1
MoBa2
MoBa3
PICCOLIPIU
ProjectViva
RHEA
Left out study

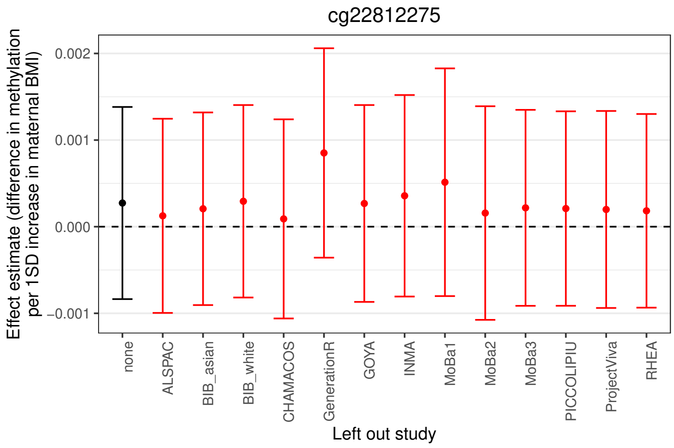
cg22812275
Effect estimate (difference in methylation per 1SD increase in maternal BMI)
0.002
0.001
0.000
−0.001
none
ALSPAC
BIB_asian
BIB_white
CHAMACOS
GenerationR
GOYA
INMA
MoBa1
MoBa2
MoBa3
PICCOLIPIU
ProjectViva
RHEA
Left out study

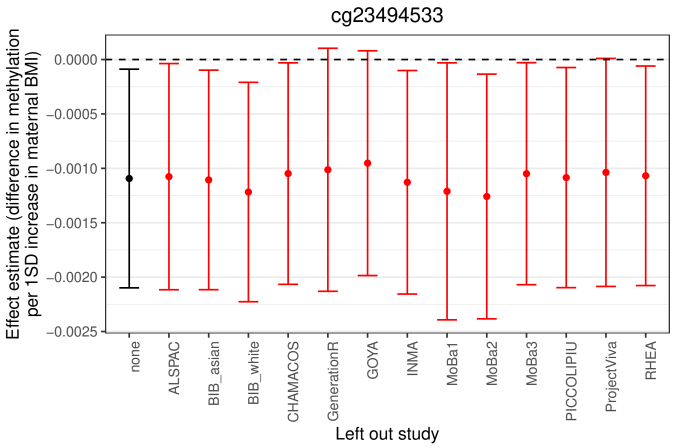
cg23494533
Effect estimate (difference in methylation per 1SD increase in maternal BMI)
0.0000
−0.0005
−0.0010
−0.0015
−0.0020
−0.0025
none
ALSPAC
BIB_asian
BIB_white
CHAMACOS
GenerationR
GOYA
INMA
MoBa1
MoBa2
MoBa3
PICCOLIPIU
ProjectViva
RHEA
Left out study

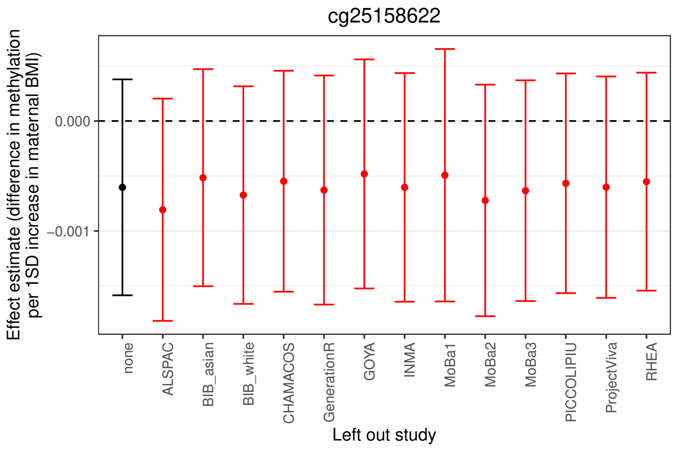
cg25158622
Effect estimate (difference in methylation per 1SD increase in maternal BMI)
0.000
−0.001
none
ALSPAC
BIB_asian
BIB_white
CHAMACOS
GenerationR
GOYA
INMA
MoBa1
MoBa2
MoBa3
PICCOLIPIU
ProjectViva
RHEA
Left out study

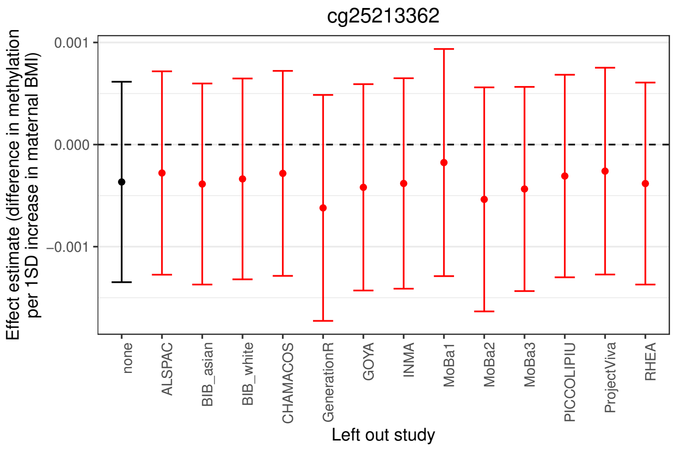
cg25213362
Effect estimate (difference in methylation per 1SD increase in maternal BMI)
0.001
0.000
−0.001
none
ALSPAC
BIB_asian
BIB_white
CHAMACOS
GenerationR
GOYA
INMA
MoBa1
MoBa2
MoBa3
PICCOLIPIU
ProjectViva
RHEA
Left out study

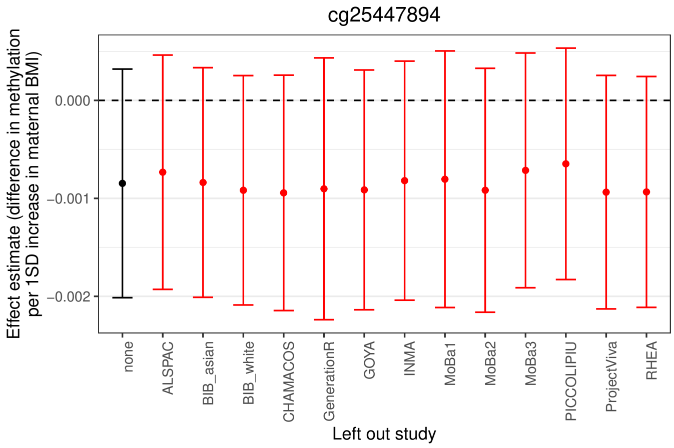
cg25447894
Effect estimate (difference in methylation per 1SD increase in maternal BMI)
0.000
−0.001
−0.002
none
ALSPAC
BIB_asian
BIB_white
CHAMACOS
GenerationR
GOYA
INMA
MoBa1
MoBa2
MoBa3
PICCOLIPIU
ProjectViva
RHEA
Left out study

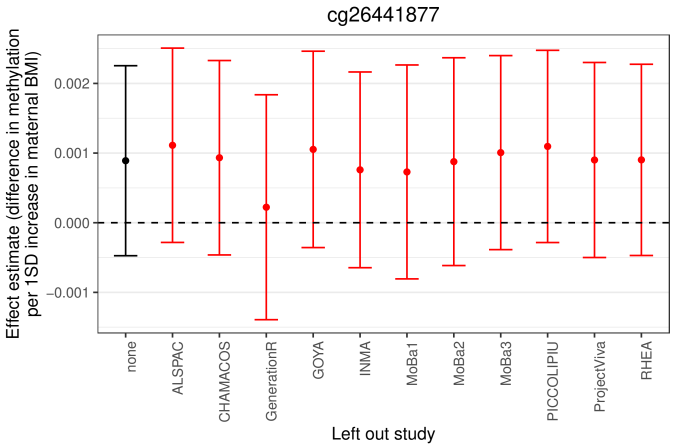
cg26441877
Effect estimate (difference in methylation per 1SD increase in maternal BMI)
0.002
0.001
0.000
−0.001
none
ALSPAC
CHAMACOS
GenerationR
GOYA
INMA
MoBa1
MoBa2
MoBa3
PICCOLIPIU
ProjectViva
RHEA
Left out study

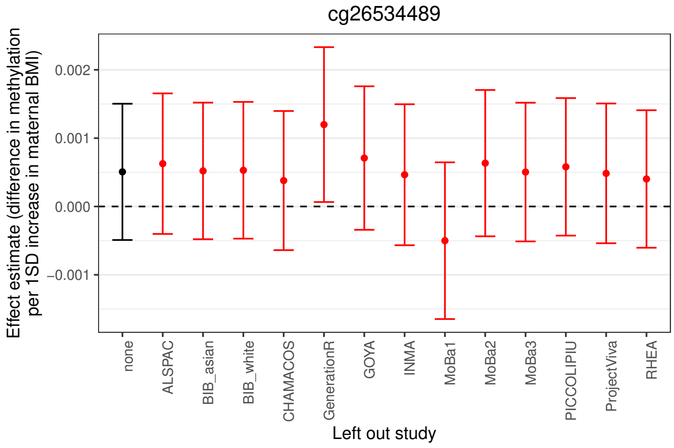
cg26534489
Effect estimate (difference in methylation per 1SD increase in maternal BMI)
0.002
0.001
0.000
−0.001
none
ALSPAC
BIB_asian
BIB_white
CHAMACOS
GenerationR
GOYA
INMA
MoBa1
MoBa2
MoBa3
PICCOLIPIU
ProjectViva
RHEA
Left out study

Maternal BMI adjusted for paternal BMI – female offspring only: pages 29-31

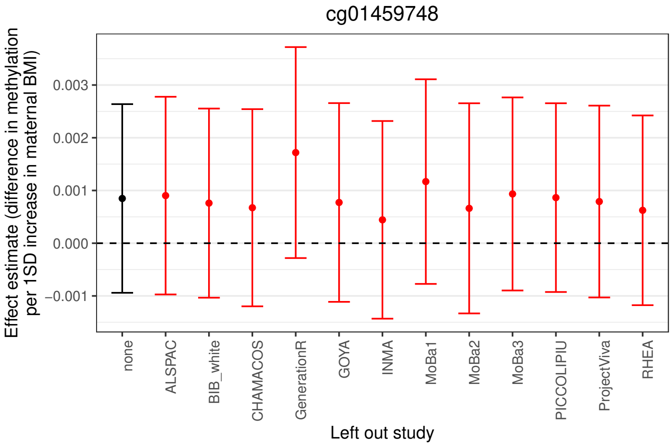
cg01459748
Effect estimate (difference in methylation per 1SD increase in maternal BMI)
0.003
0.002
0.001
0.000
−0.001
none
ALSPAC
BIB_white
CHAMACOS
GenerationR
GOYA
INMA
MoBa1
MoBa2
MoBa3
PICCOLIPIU
ProjectViva
RHEA
Left out study

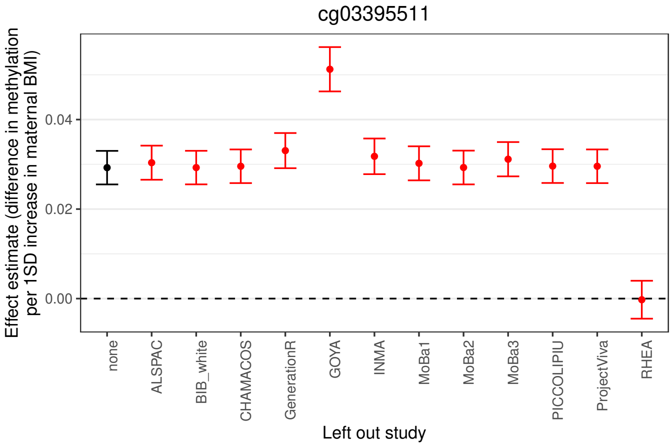
cg03395511
Effect estimate (difference in methylation per 1SD increase in maternal BMI)
0.04
0.02
0.00
none
ALSPAC
BIB_white
CHAMACOS
GenerationR
GOYA
INMA
MoBa1
MoBa2
MoBa3
PICCOLIPIU
ProjectViva
RHEA
Left out study

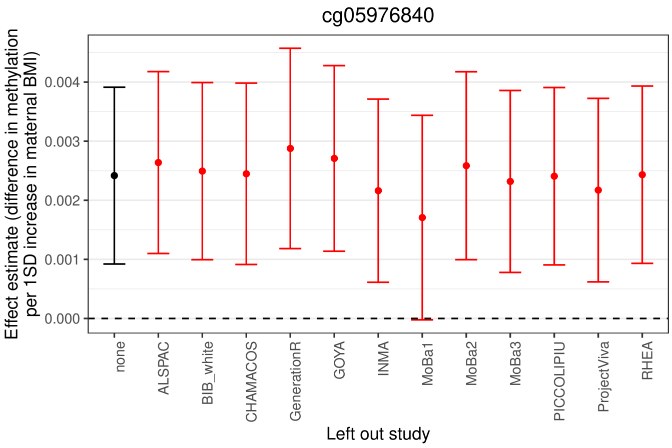
cg05976840
Effect estimate (difference in methylation per 1SD increase in maternal BMI)
0.004
0.003
0.002
0.001
0.000
none
ALSPAC
BIB_white
CHAMACOS
GenerationR
GOYA
INMA
MoBa1
MoBa2
MoBa3
PICCOLIPIU
ProjectViva
RHEA
Left out study

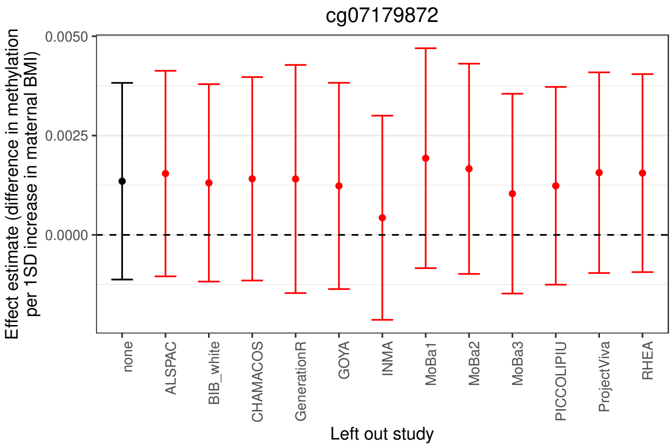
cg07179872
Effect estimate (difference in methylation per 1SD increase in maternal BMI)
0.0050
0.0025
0.0000
none
ALSPAC
BIB_white
CHAMACOS
GenerationR
GOYA
INMA
MoBa1
MoBa2
MoBa3
PICCOLIPIU
ProjectViva
RHEA
Left out study

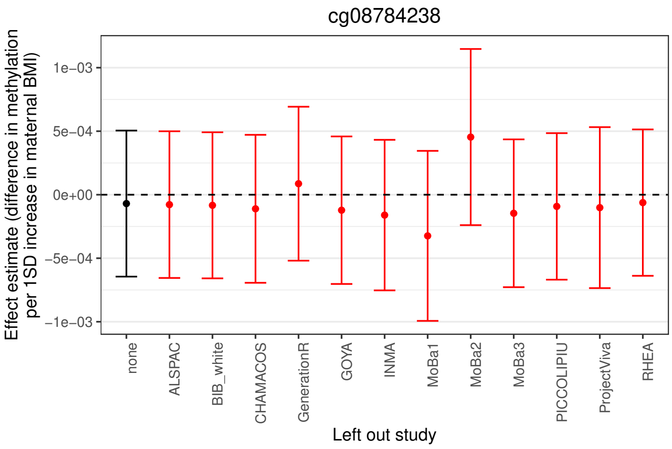
cg08784238
Effect estimate (difference in methylation per 1SD increase in maternal BMI)
1e−03
5e−04
0e+00
−5e−04
−1e−03
none
ALSPAC
BIB_white
CHAMACOS
GenerationR
GOYA
INMA
MoBa1
MoBa2
MoBa3
PICCOLIPIU
ProjectViva
RHEA
Left out study

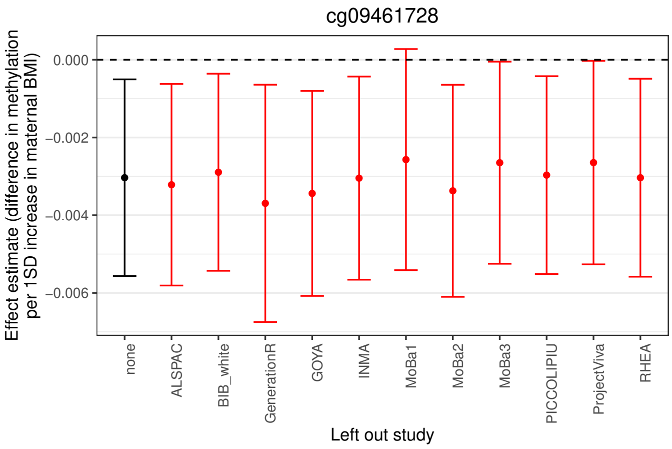
cg09461728
Effect estimate (difference in methylation per 1SD increase in maternal BMI)
0.000
−0.002
−0.004
−0.006
none
ALSPAC
BIB_white
GenerationR
GOYA
INMA
MoBa1
MoBa2
MoBa3
PICCOLIPIU
ProjectViva
RHEA
Left out study

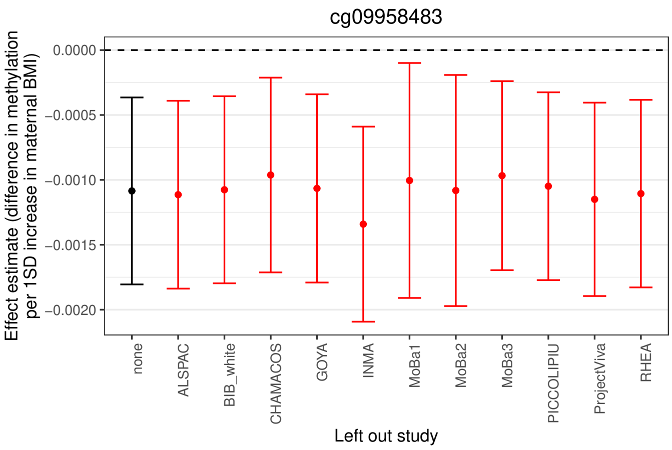
cg09958483
Effect estimate (difference in methylation per 1SD increase in maternal BMI)
0.0000
−0.0005
−0.0010
−0.0015
−0.0020
none
ALSPAC
BIB_white
CHAMACOS
GOYA
INMA
MoBa1
MoBa2
MoBa3
PICCOLIPIU
ProjectViva
RHEA
Left out study

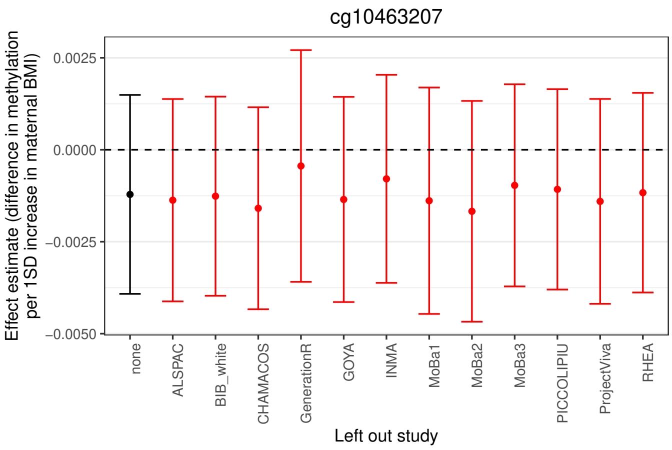
cg10463207
Effect estimate (difference in methylation per 1SD increase in maternal BMI)
0.0025
0.0000
−0.0025
−0.0050
none
ALSPAC
BIB_white
CHAMACOS
GenerationR
GOYA
INMA
MoBa1
MoBa2
MoBa3
PICCOLIPIU
ProjectViva
RHEA
Left out study

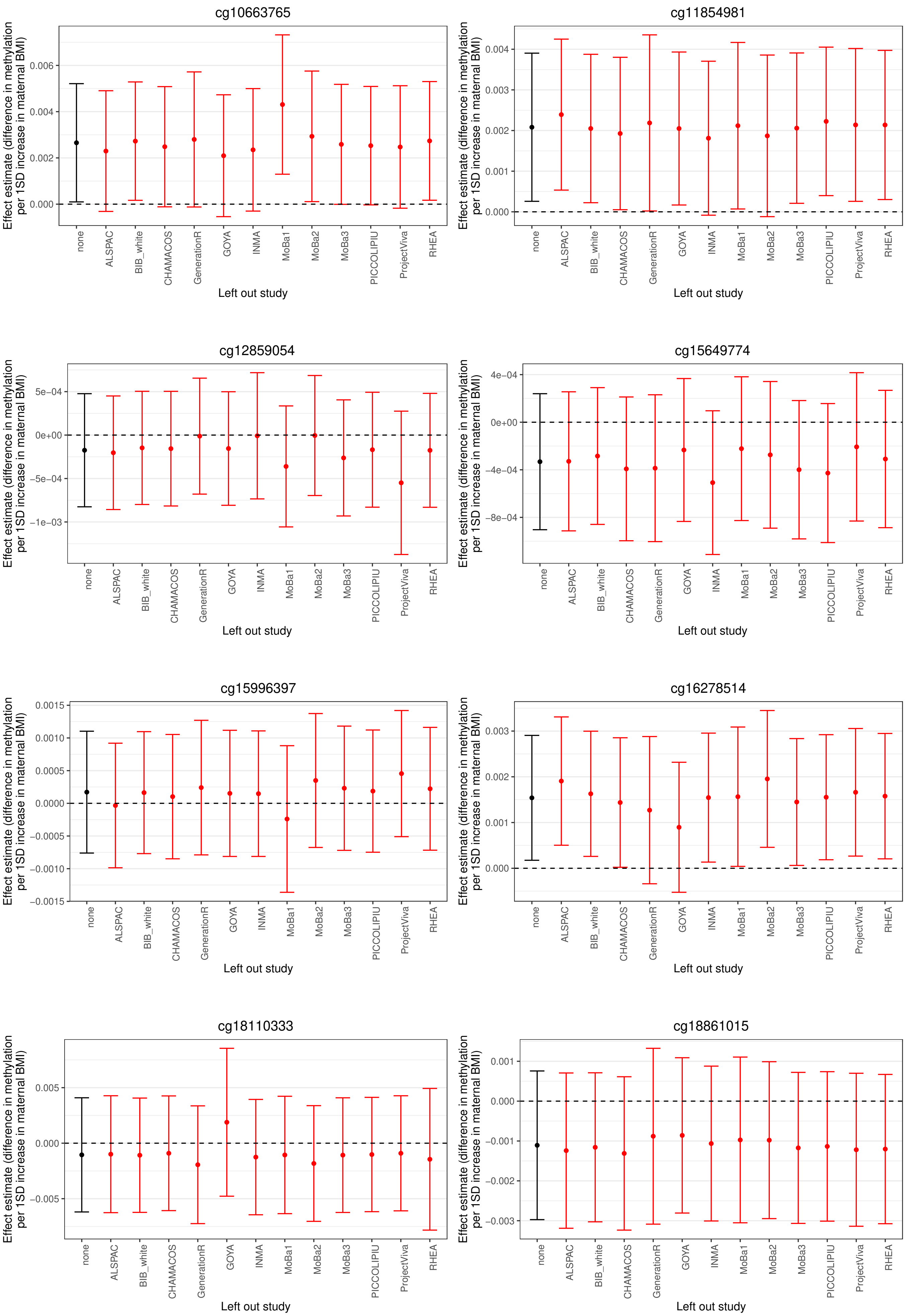
cg10663765
Effect estimate (difference in methylation per 1SD increase in maternal BMI)
0.000
0.002
0.004
0.006
none
ALSPAC
BIB_white
CHAMACOS
GenerationR
GOYA
INMA
MoBa1
MoBa2
MoBa3
PICCOLIPIU
ProjectViva
RHEA
Left out study
cg11854981
0.000
0.001
0.002
0.003
0.004
cg12859054
5e−04
0e+00
−5e−04
−1e−03
cg15649774
4e−04
0e+00
−4e−04
−8e−04
cg15996397
0.0015
0.0010
0.0005
0.0000
−0.0005
−0.0010
−0.0015
cg16278514
0.003
0.002
0.001
0.000
cg18110333
0.005
0.000
−0.005
cg18861015
0.001
0.000
−0.001
−0.002
−0.003

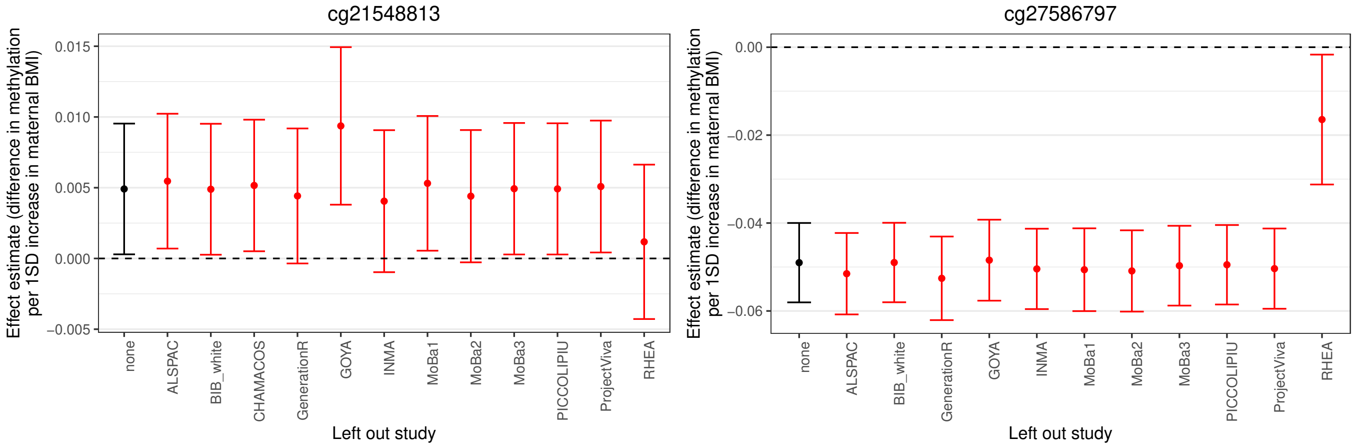
cg21548813
Effect estimate (difference in methylation per 1SD increase in maternal BMI)
0.015
0.010
0.005
0.000
−0.005
none
ALSPAC
BIB_white
CHAMACOS
GenerationR
GOYA
INMA
MoBa1
MoBa2
MoBa3
PICCOLIPIU
ProjectViva
RHEA
Left out study
cg27586797
Effect estimate (difference in methylation per 1SD increase in maternal BMI)
0.00
−0.02
−0.04
−0.06
none
ALSPAC
BIB_white
GenerationR
GOYA
INMA
MoBa1
MoBa2
MoBa3
PICCOLIPIU
ProjectViva
RHEA
Left out study

Maternal BMI adjusted for paternal BMI – male offspring only: pages 33-34

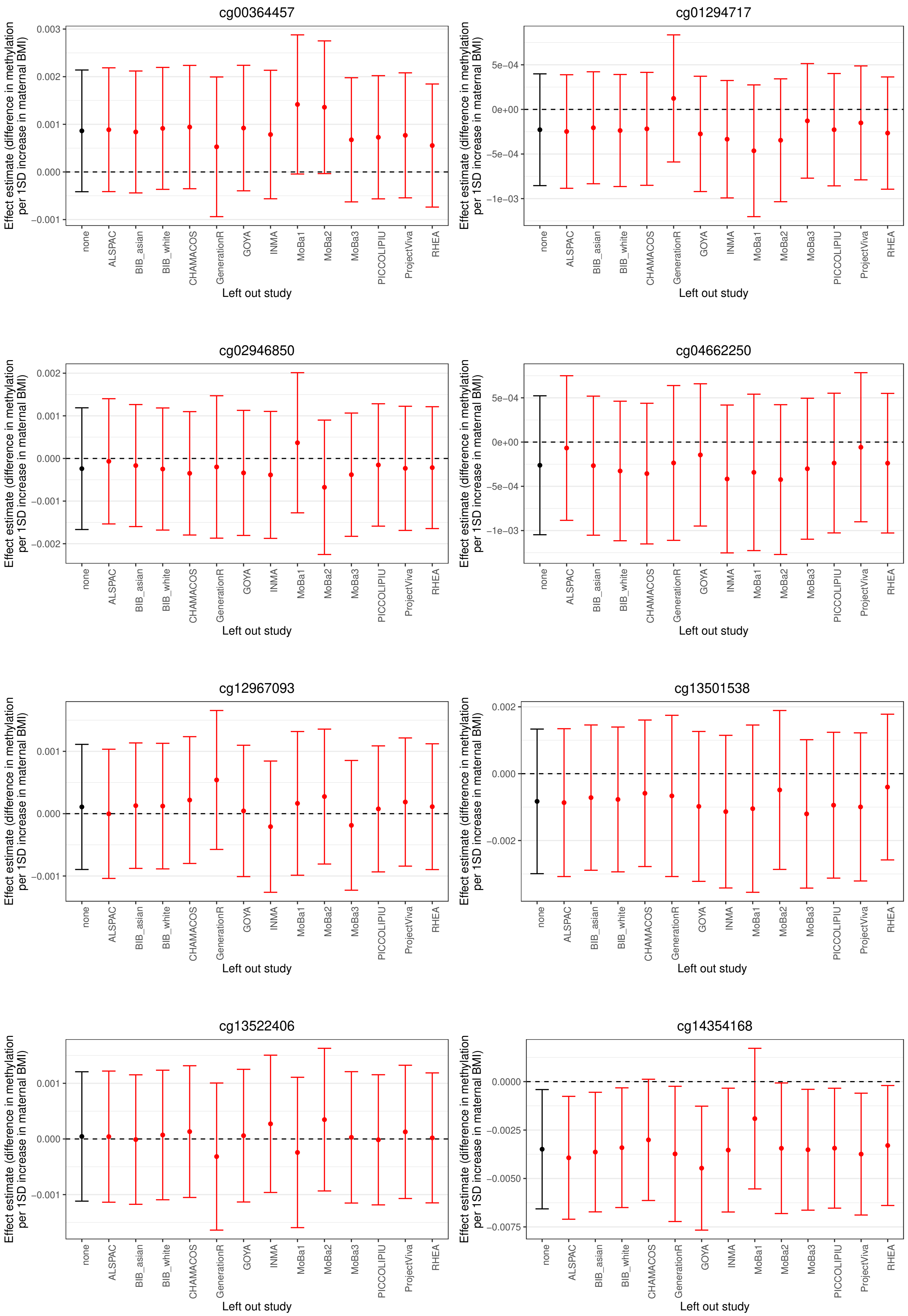
cg00364457
cg01294717
cg02946850
cg04662250
cg12967093
cg13501538
cg13522406
cg14354168
Effect estimate (difference in methylation per 1SD increase in maternal BMI)
Left out study
none
ALSPAC
BIB_asian
BIB_white
CHAMACOS
GenerationR
GOYA
INMA
MoBa1
MoBa2
MoBa3
PICCOLIPIU
ProjectViva
RHEA

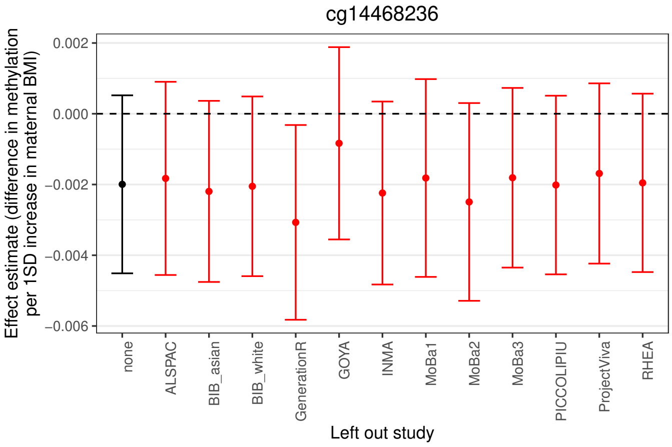
cg14468236
Effect estimate (difference in methylation per 1SD increase in maternal BMI)
0.002
0.000
−0.002
−0.004
−0.006
none
ALSPAC
BIB_asian
BIB_white
GenerationR
GOYA
INMA
MoBa1
MoBa2
MoBa3
PICCOLIPIU
ProjectViva
RHEA
Left out study

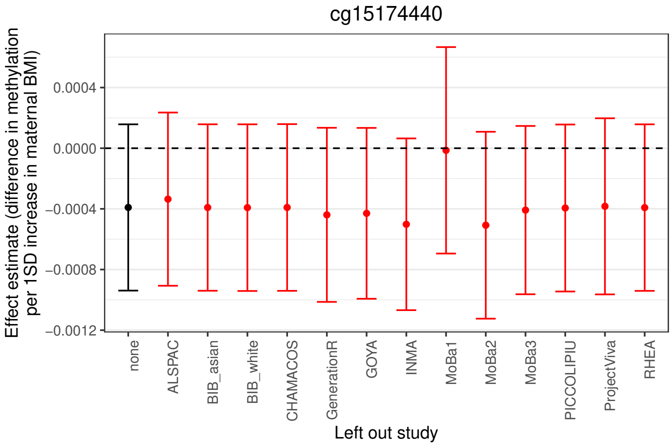
cg15174440
Effect estimate (difference in methylation per 1SD increase in maternal BMI)
0.0004
0.0000
−0.0004
−0.0008
−0.0012
none
ALSPAC
BIB_asian
BIB_white
CHAMACOS
GenerationR
GOYA
INMA
MoBa1
MoBa2
MoBa3
PICCOLIPIU
ProjectViva
RHEA
Left out study

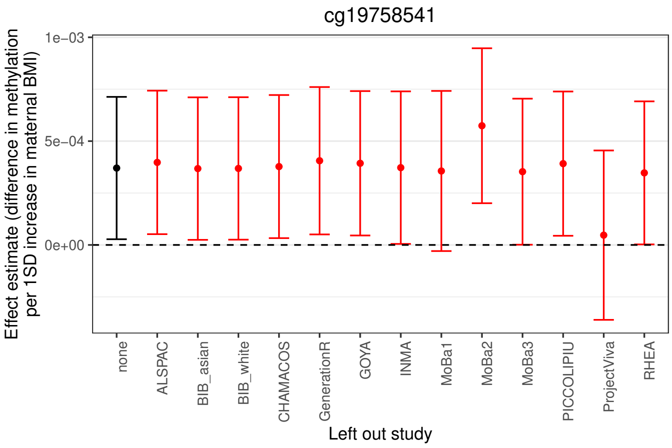
cg19758541
Effect estimate (difference in methylation per 1SD increase in maternal BMI)
1e−03
5e−04
0e+00
none
ALSPAC
BIB_asian
BIB_white
CHAMACOS
GenerationR
GOYA
INMA
MoBa1
MoBa2
MoBa3
PICCOLIPIU
ProjectViva
RHEA
Left out study

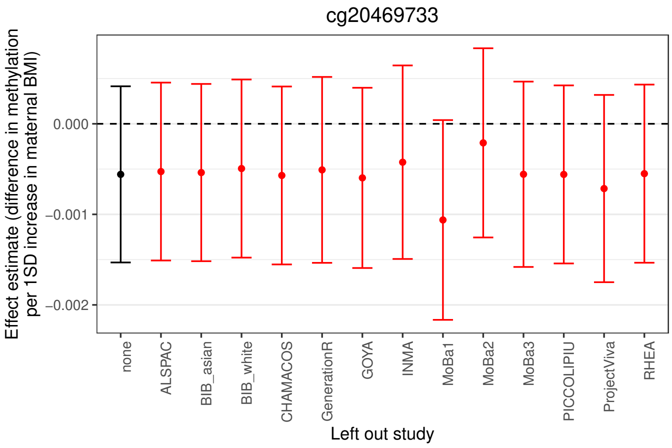
cg20469733
Effect estimate (difference in methylation per 1SD increase in maternal BMI)
0.000
−0.001
−0.002
none
ALSPAC
BIB_asian
BIB_white
CHAMACOS
GenerationR
GOYA
INMA
MoBa1
MoBa2
MoBa3
PICCOLIPIU
ProjectViva
RHEA
Left out study

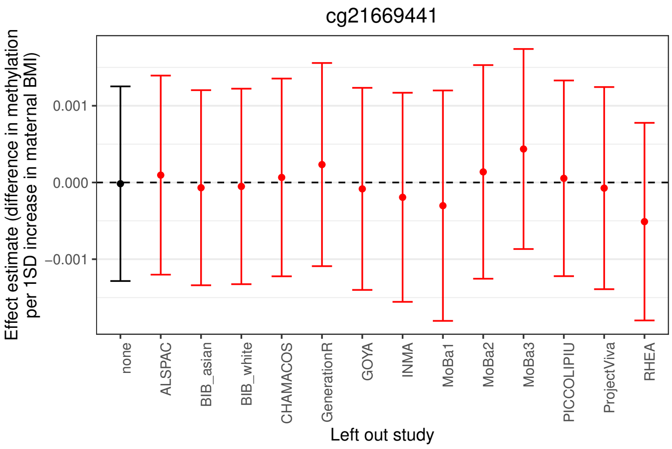
cg21669441
Effect estimate (difference in methylation per 1SD increase in maternal BMI)
0.001
0.000
−0.001
none
ALSPAC
BIB_asian
BIB_white
CHAMACOS
GenerationR
GOYA
INMA
MoBa1
MoBa2
MoBa3
PICCOLIPIU
ProjectViva
RHEA
Left out study

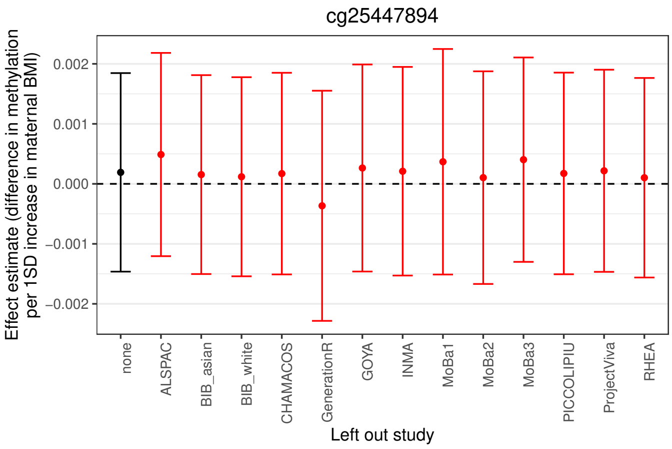
cg25447894
Effect estimate (difference in methylation per 1SD increase in maternal BMI)
0.002
0.001
0.000
−0.001
−0.002
none
ALSPAC
BIB_asian
BIB_white
CHAMACOS
GenerationR
GOYA
INMA
MoBa1
MoBa2
MoBa3
PICCOLIPIU
ProjectViva
RHEA
Left out study

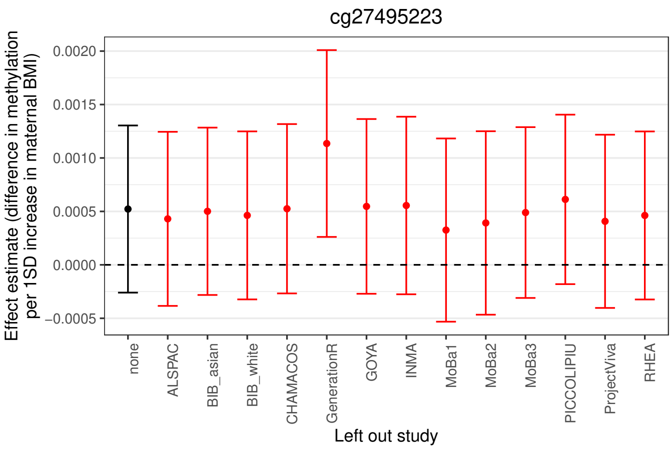
cg27495223
Effect estimate (difference in methylation per 1SD increase in maternal BMI)
0.0020
0.0015
0.0010
0.0005
0.0000
−0.0005
none
ALSPAC
BIB_asian
BIB_white
CHAMACOS
GenerationR
GOYA
INMA
MoBa1
MoBa2
MoBa3
PICCOLIPIU
ProjectViva
RHEA
Left out study
