# Supplementary material for: Paternal body mass index and offspring DNA methylation: findings from the PACE consortium
Source: Int J Epidemiol. 2021 Jan 29;50(4):1297–315. doi: 10.1093/ije/dyaa267 (PMC8407864; doi:10.1093/ije/dyaa267)
Supplement: dyaa267_Supplementary_Data [file dyaa267_supplementary_data.zip › ije-2020-05-0817-File013.docx]

Comparison of paternal and maternal BMI EWAS effect estimates (after mutual adjustment)

*Absolute effect estimates (y-axis) plotted against genomic location (x-axis; numbers indicate chromosome number). Paternal BMI meta-EWAS results are plotted on the top, with maternal meta-EWAS results on a mirrored axis below. Models were mutually adjusted for the other parent’s BMI.*
