# Supplementary material for: Paternal body mass index and offspring DNA methylation: findings from the PACE consortium
Source: Int J Epidemiol. 2021 Jan 29;50(4):1297–315. doi: 10.1093/ije/dyaa267 (PMC8407864; doi:10.1093/ije/dyaa267)

**Cohort-specific EWAS results quality checks: childhood**

For each cohort, we plotted QQ plots and a correlation matrix showing correlations of EWAS effect estimates for each model (pages 3-14). “Precision plots” comparing 1/median(standard error) to sample size are shown on page 15. Page 16 shows the distribution of effect estimates for each cohort and each model.

**EWAS models:**

**Covariates-adjusted: Paternal BMI + Covariates + Cells (model name: covs.pat)**

Methylation ~ Paternal BMI + SVs for batch + Paternal age + Paternal smoking status + Paternal socioeconomic status + Maternal age + Maternal smoking status + Parity + Estimated cell counts

**Covariates-adjusted: Maternal BMI + Covariates + Cells (model name: covs.mat)**

Methylation ~ Maternal BMI + SVs for batch + Paternal age + Paternal smoking status + Paternal socioeconomic status + Maternal age + Maternal smoking status + Parity + Estimated cell counts

**Covariates-adjusted: Paternal adjusted for maternal BMI, covariates and cells (model name: covs.patmat)**

Methylation ~ Paternal BMI + Maternal BMI + SVs for batch + Paternal age + Paternal smoking status + Paternal socioeconomic status + Maternal age + Maternal smoking status + Parity + Estimated cell counts

**Covariates-adjusted: Maternal adjusted for paternal BMI, covariates and cells (model name: covs.matpat)**

Methylation ~ Paternal BMI + Maternal BMI + SVs for batch + Paternal age + Paternal smoking status + Paternal socioeconomic status + Maternal age + Maternal smoking status + Parity + Estimated cell counts

**Sex-stratified: Paternal adjusted for maternal in males (model name: boys.patmat)**

Methylation ~ Paternal BMI + Maternal BMI + SVs for batch + Paternal age + Paternal smoking status + Paternal socioeconomic status + Maternal age + Maternal smoking status + Parity + Estimated cell counts

**Sex-stratified: Maternal adjusted for paternal in males (model name: boys.matpat)**

Methylation ~ Paternal BMI + Maternal BMI + SVs for batch + Paternal age + Paternal smoking status + Paternal socioeconomic status + Maternal age + Maternal smoking status + Parity + Estimated cell counts

**Sex-stratified: Paternal adjusted for maternal in males (model name: girls.patmat)**

Methylation ~ Paternal BMI + Maternal BMI + SVs for batch + Paternal age + Paternal smoking status + Paternal socioeconomic status + Maternal age + Maternal smoking status + Parity + Estimated cell counts

**Sex-stratified: Maternal adjusted for paternal in females(model name: girls.matpat)**

Methylation ~ Paternal BMI + Maternal BMI + SVs for batch + Paternal age + Paternal smoking status + Paternal socioeconomic status + Maternal age + Maternal smoking status + Parity + Estimated cell counts

Note: the following “minimally-adjusted” models are included in the plots below for completeness and transparency but were not included in downstream analyses or discussed in the maintext of this study. The decision to omit these models was made before assessment of the results, based on the observation that all participating cohorts had the necessary data to enable them to take part in the covariates-adjusted analyses. We favoured the covariates-adjusted models over the minimally-adjusted models because they explicitly adjust for important potential confounders. As the plots below show, results from the minimally-adjusted and covariate-adjusted models were similar and it is very unlikely that our conclusions from this study would be different had we focused on the minimally-adjusted models instead of the covariate-adjusted models.

**Minimally-adjusted: Paternal BMI + Cells (model name: min.pat)**

Methylation ~ Paternal BMI + SVs for batch + Estimated cell counts

**Minimally-adjusted: Maternal BMI + Cells (model name: min.mat)**

Methylation ~ Maternal BMI + SVs for batch + Estimated cell counts

**Minimally-adjusted: Paternal adjusted for maternal BMI + cells (model name: min.patmat)**

Methylation ~ Paternal BMI + Maternal BMI + SVs for batch + Estimated cell counts

**Minimally-adjusted: Maternal adjusted for paternal + cells (model name: min.matpat)**

Methylation ~ Paternal BMI + Maternal BMI + SVs for batch + Estimated cell counts


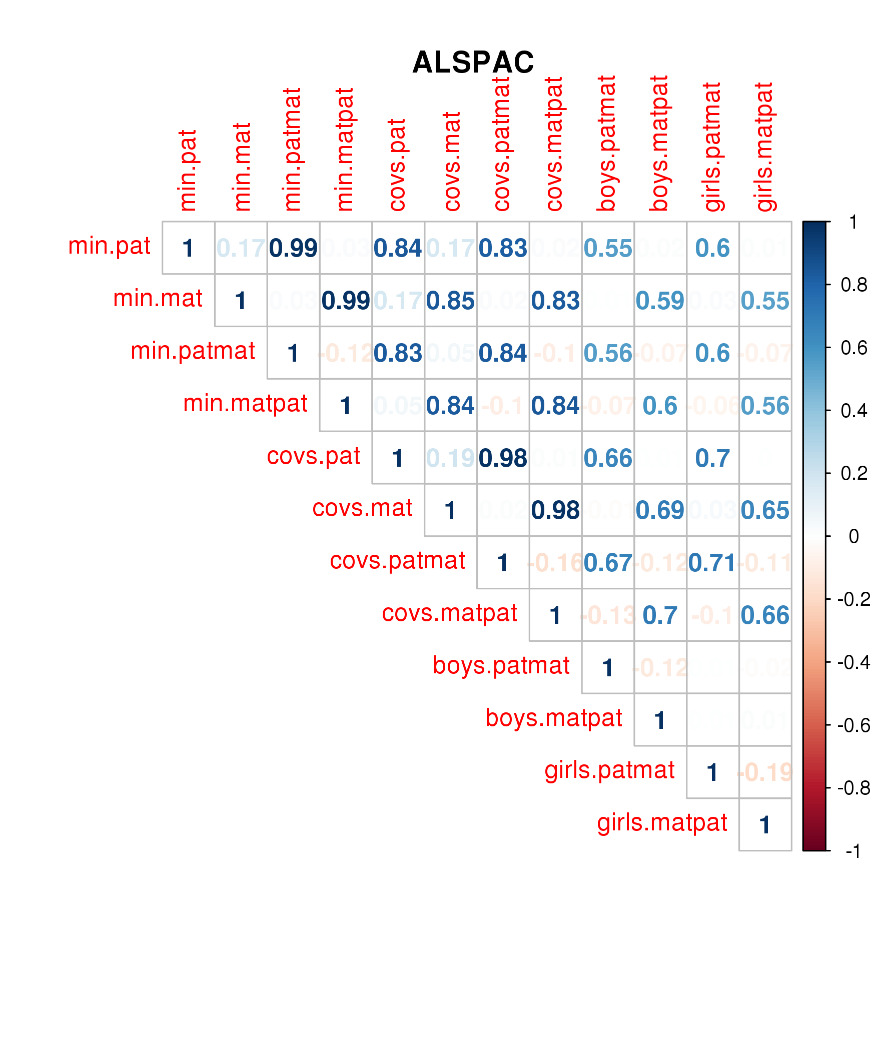

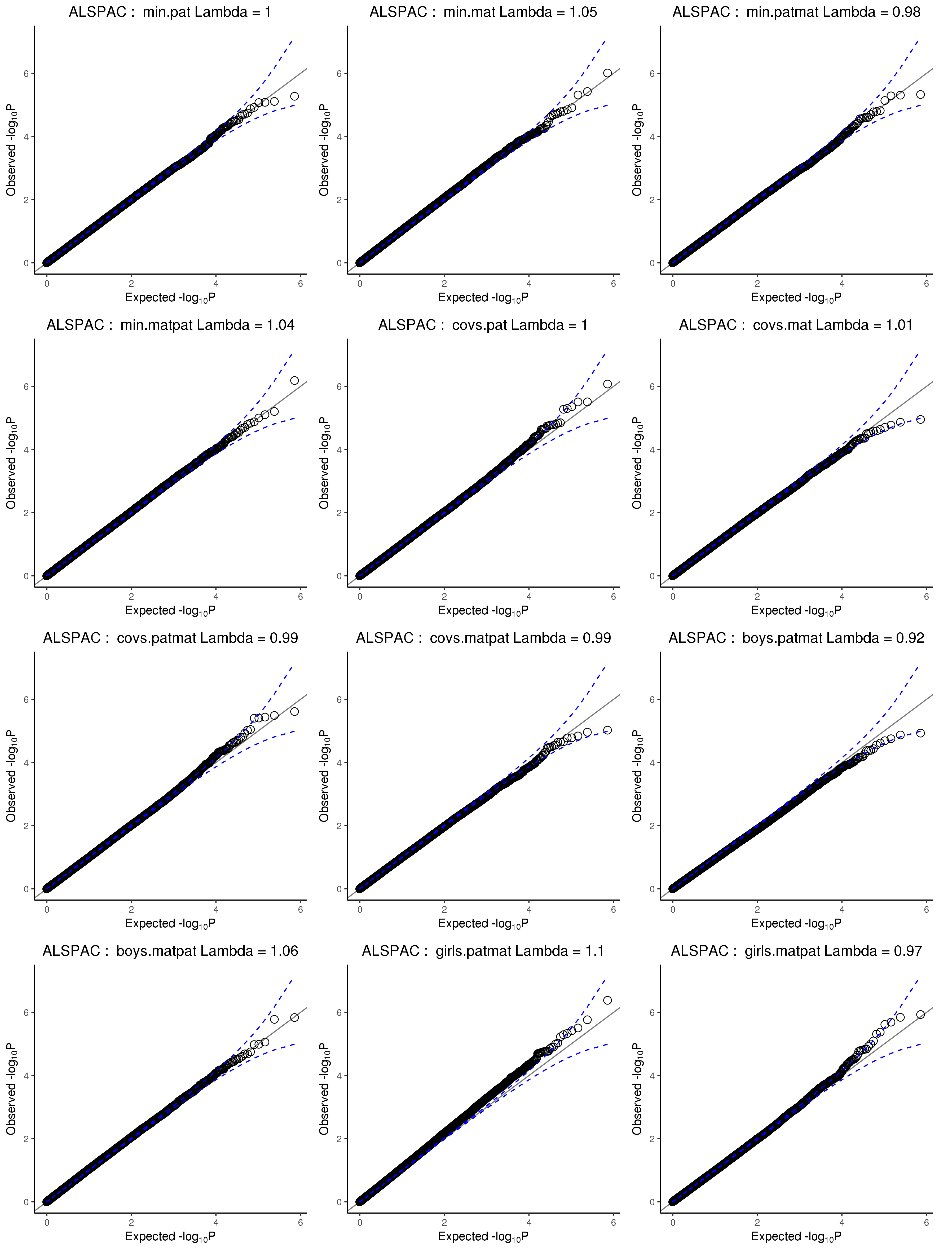

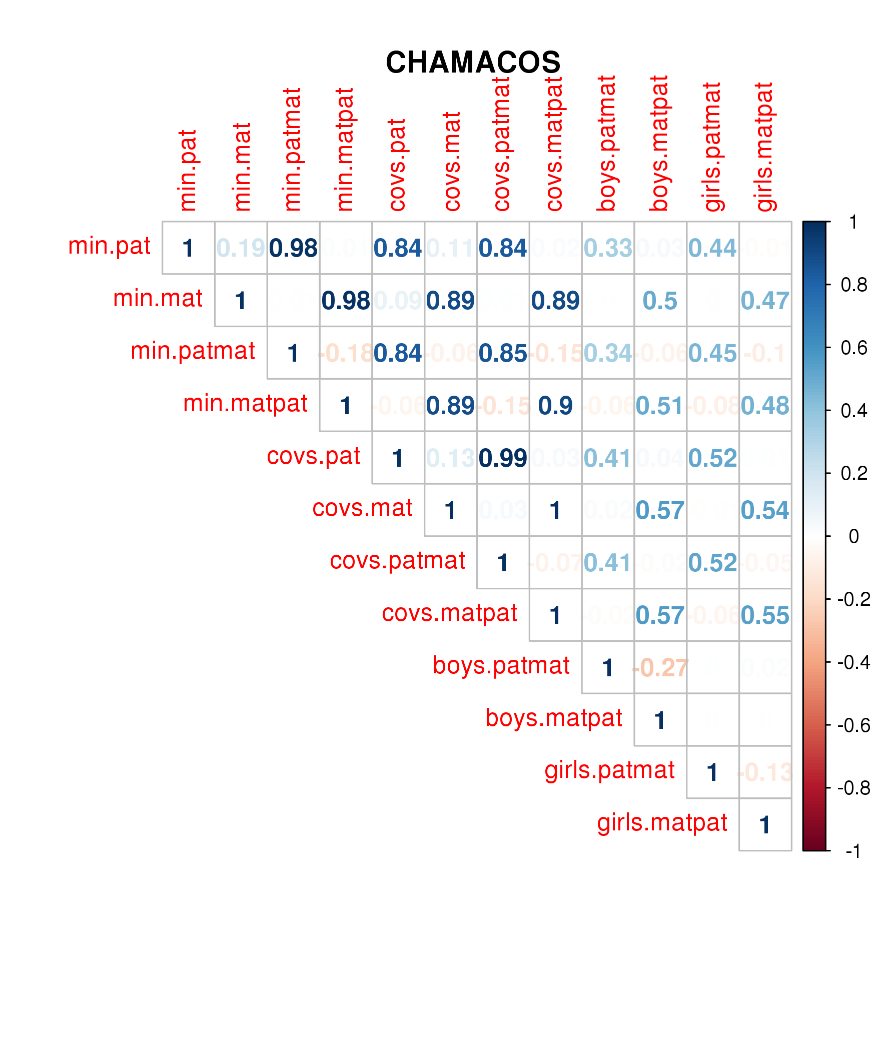

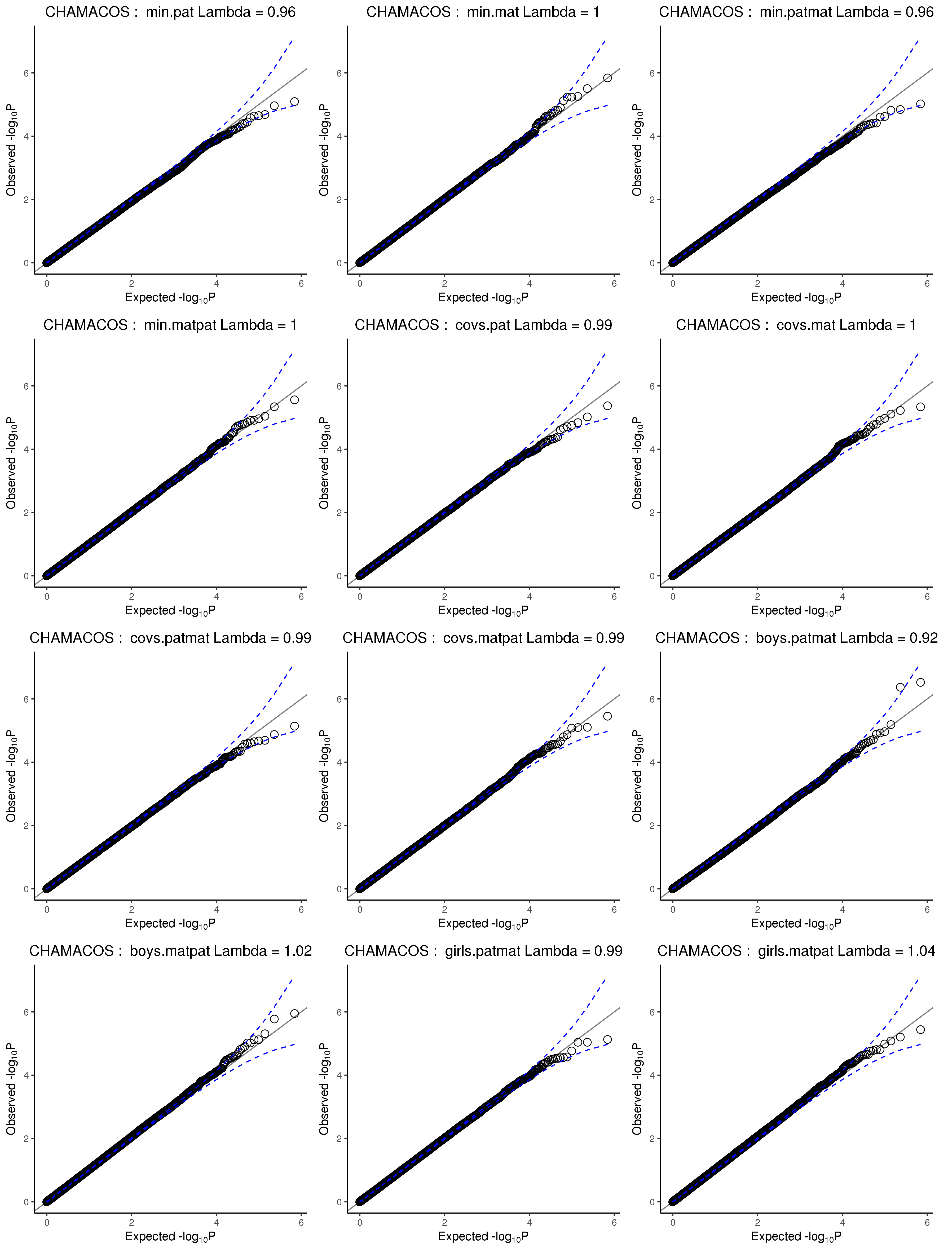

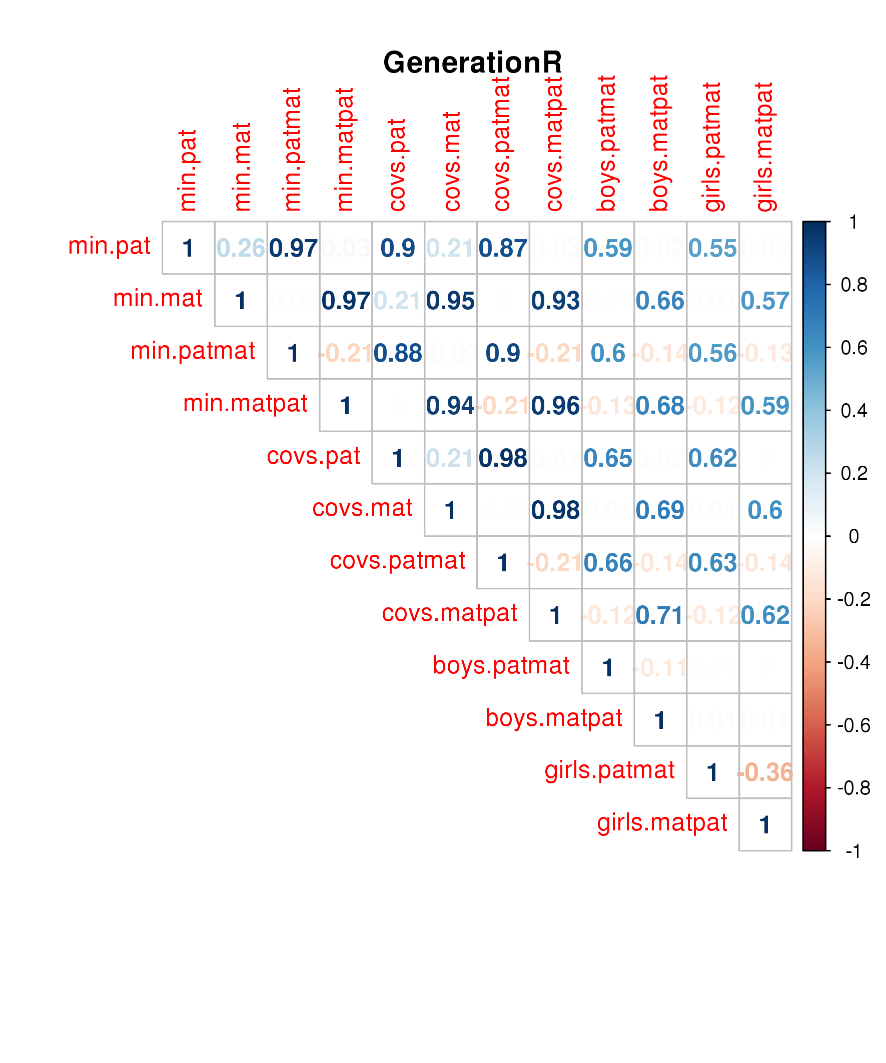

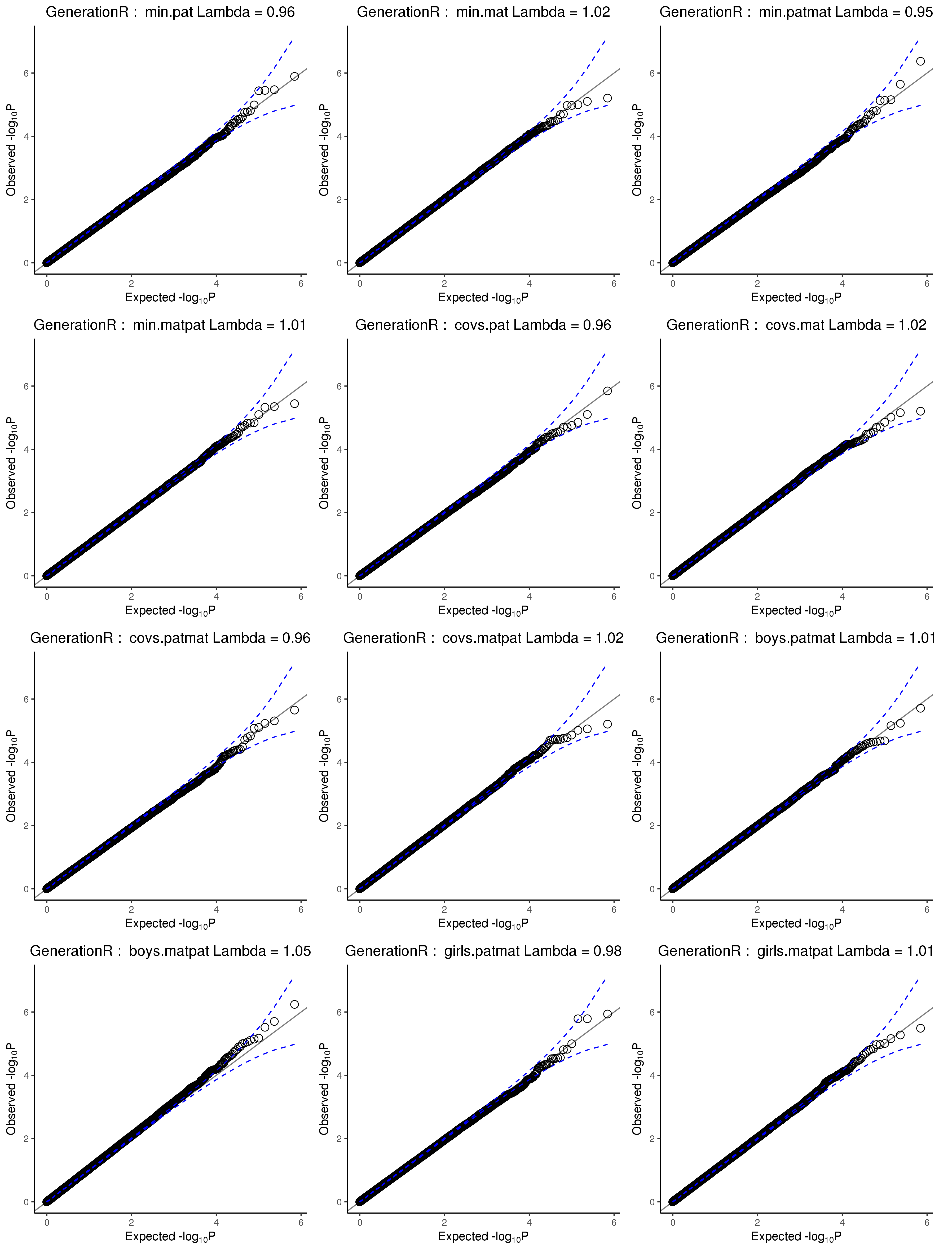

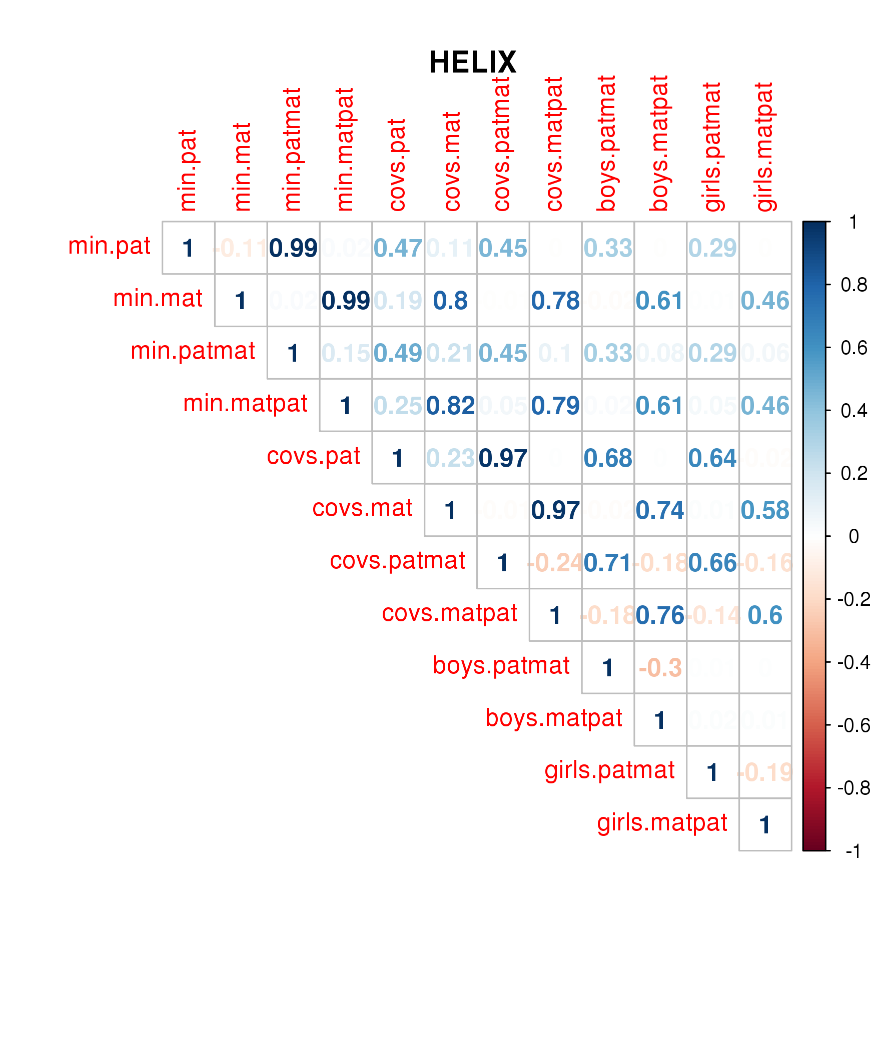

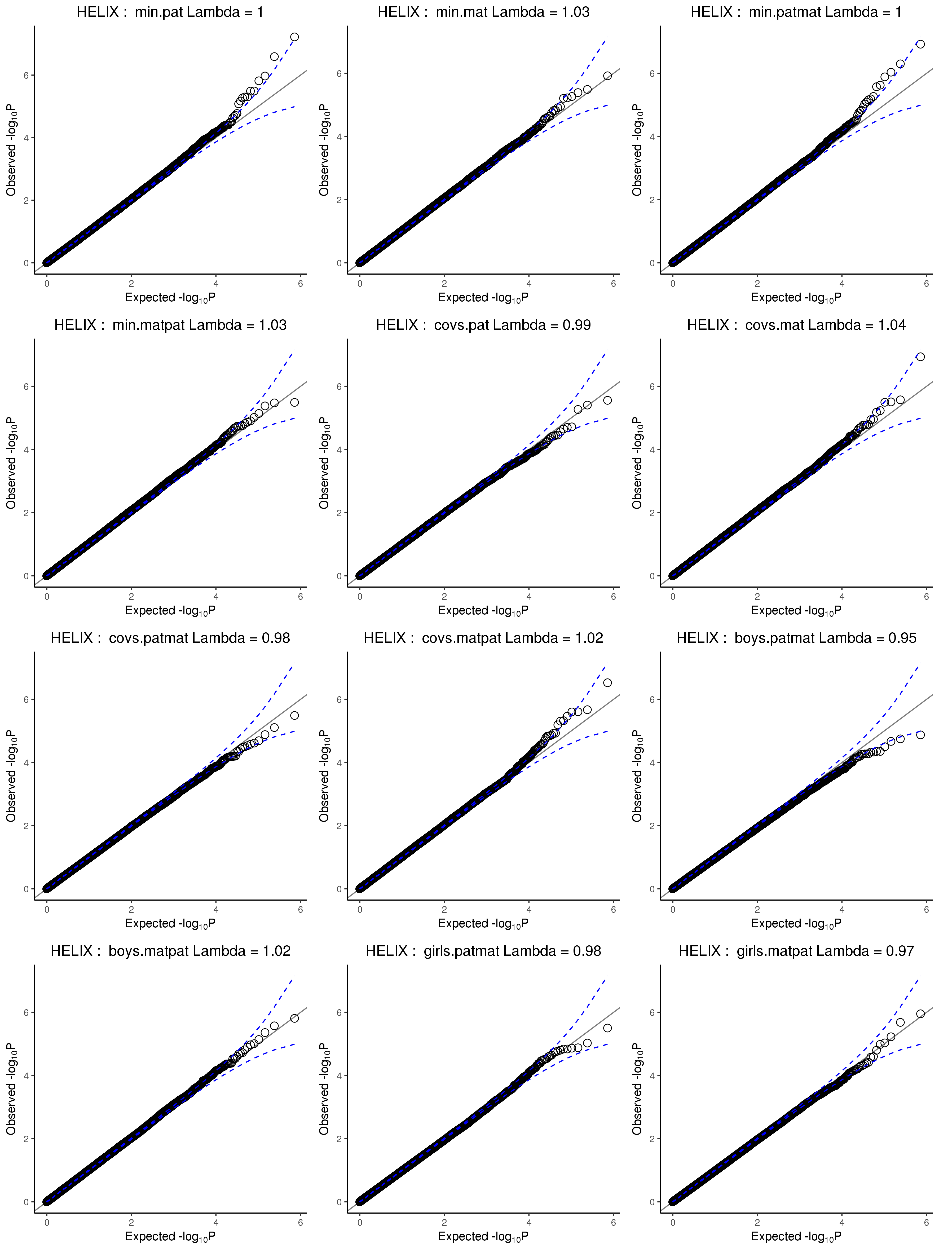

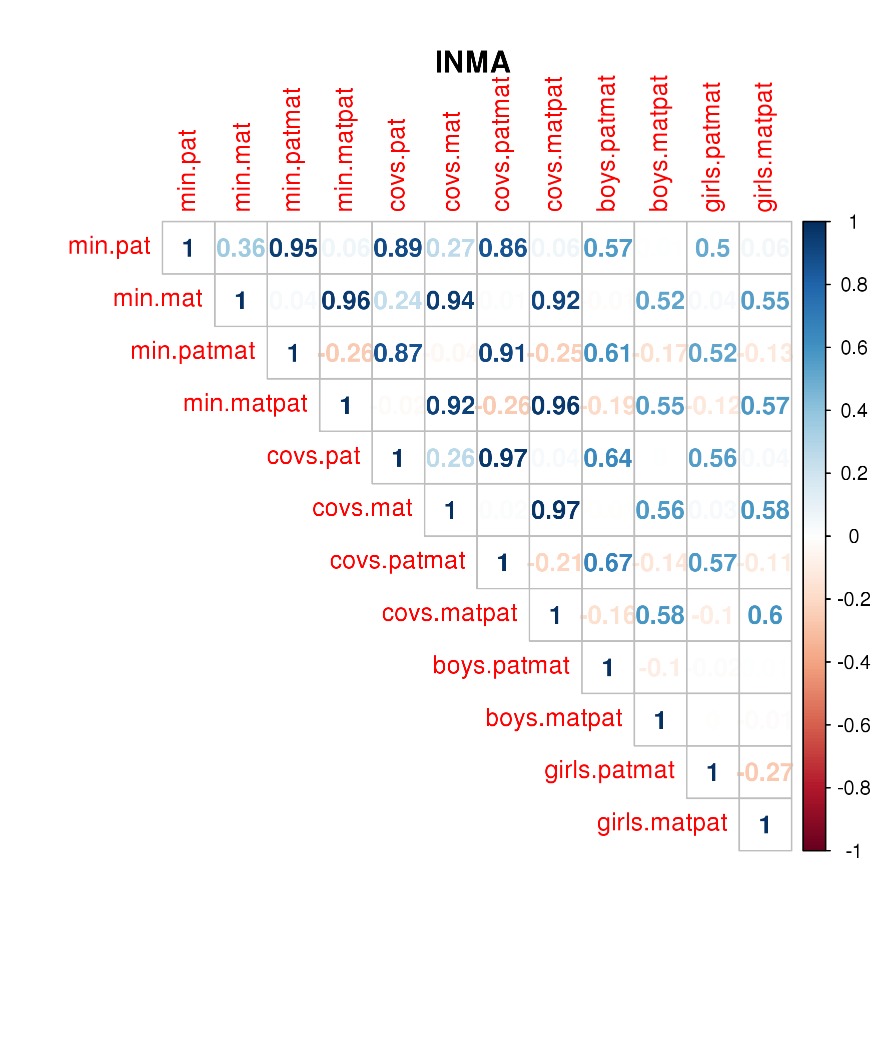

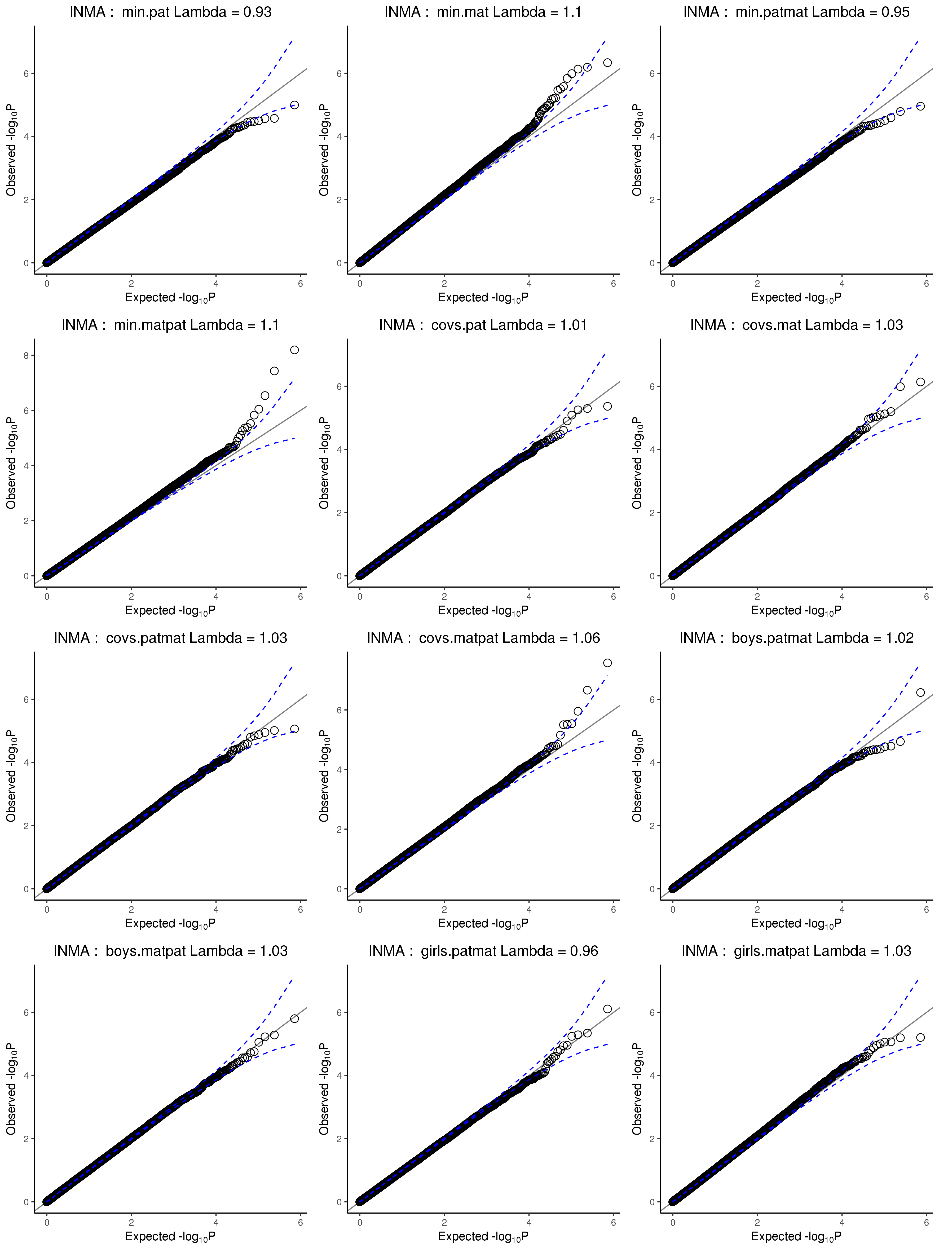

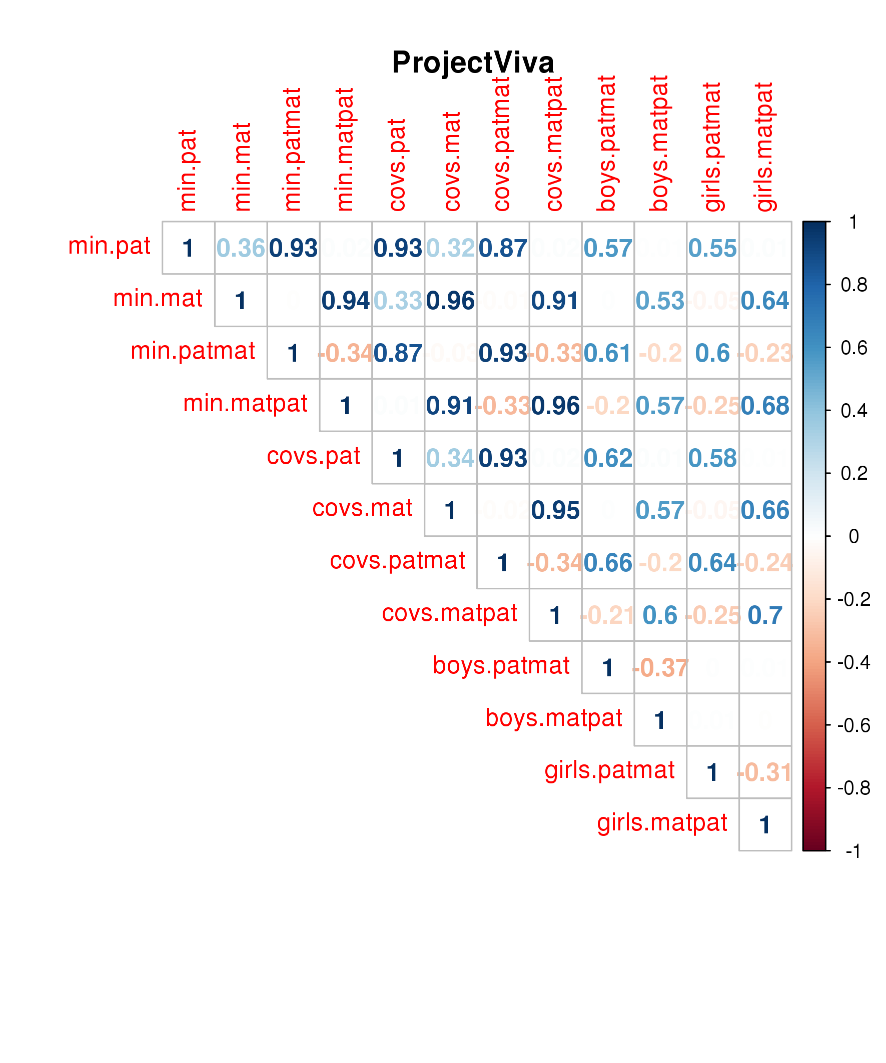

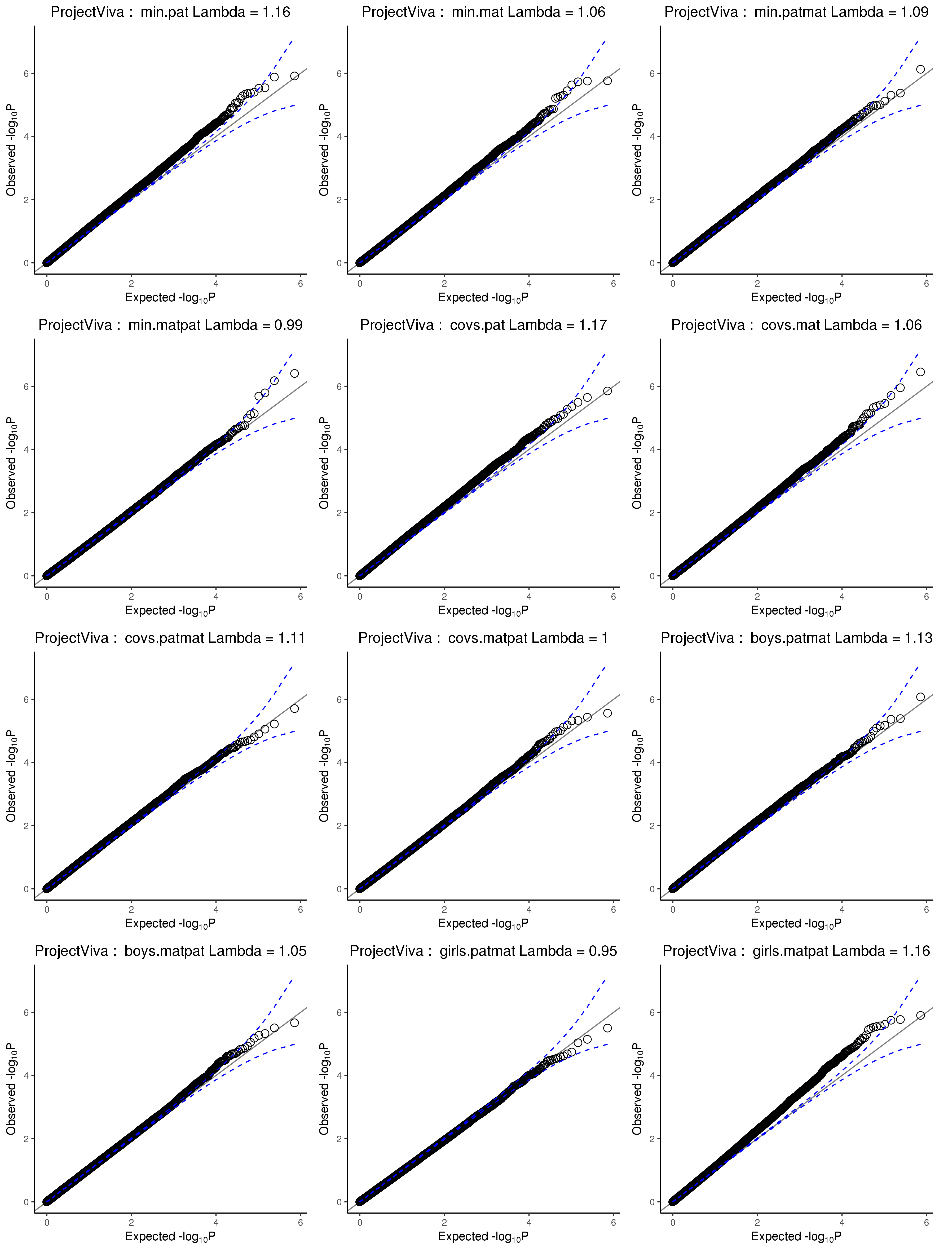


Abbreviations: AL, ALSPAC; CH, CHAMACOS; GR, GenerationR; HE, HELIX; IN, INMA; PV, Project Viva


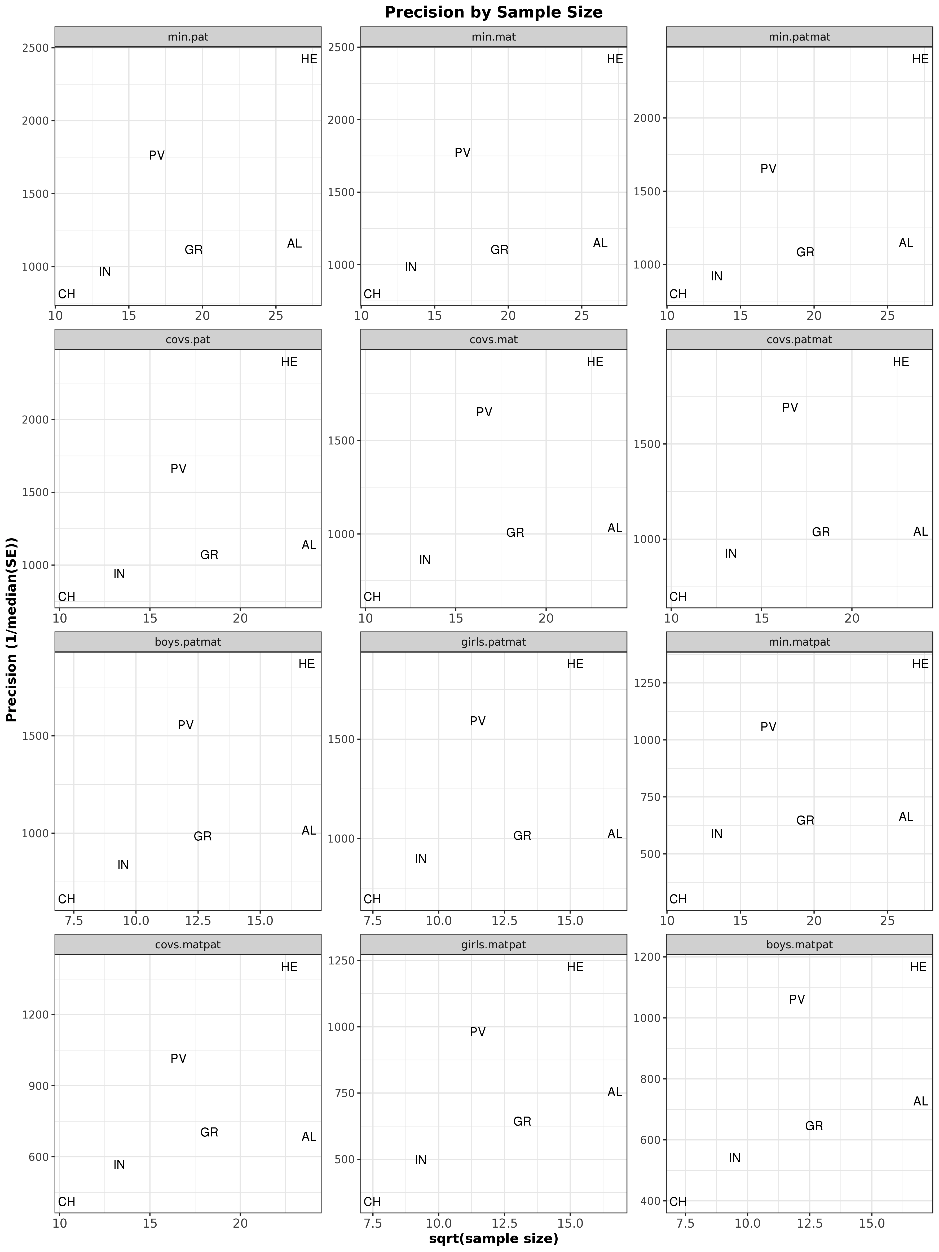


Abbreviations: AL, ALSPAC; CH, CHAMACOS; GR, GenerationR; HE, HELIX; IN, INMA; PV, Project Viva


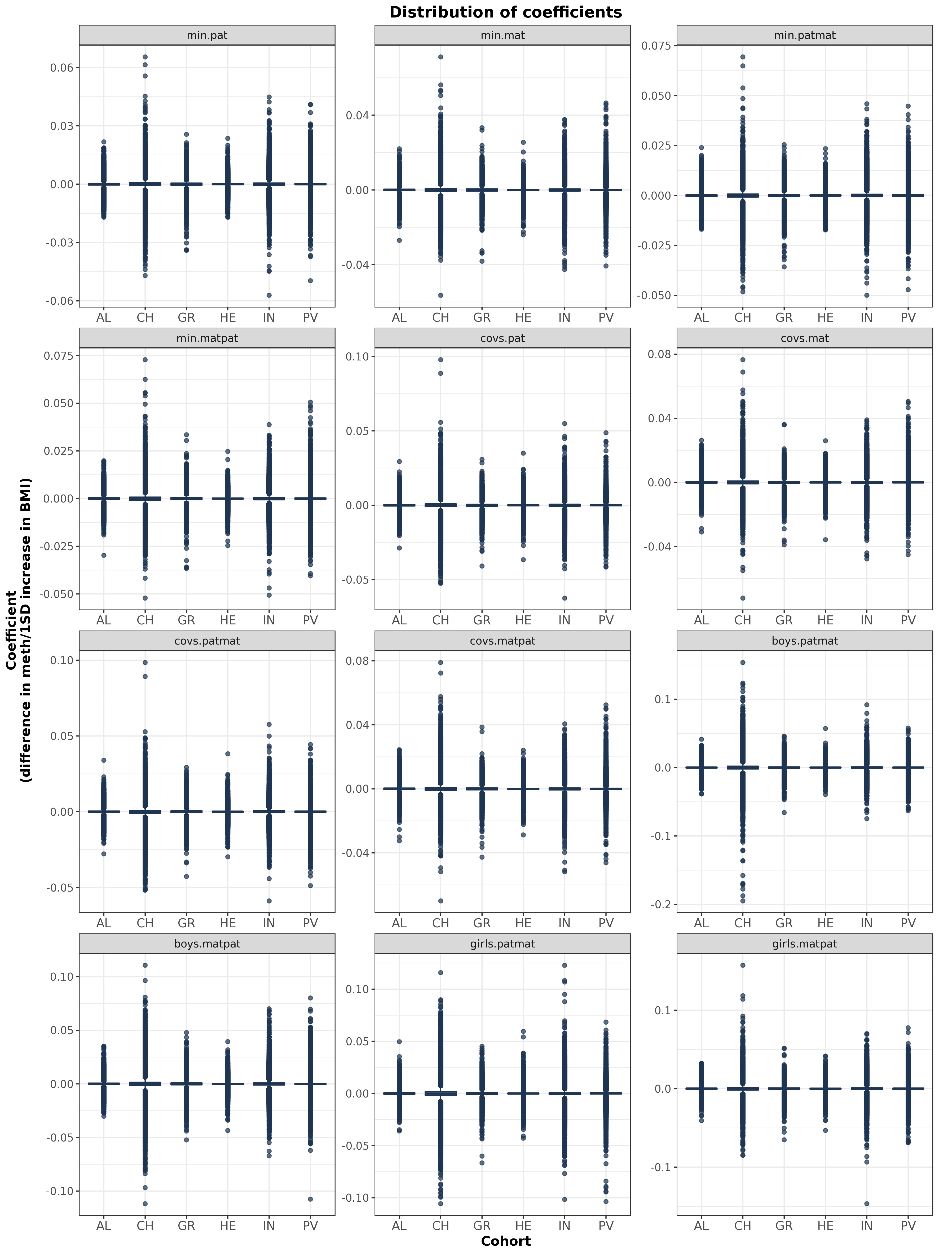

Supplement: dyaa267_Supplementary_Data [file dyaa267_supplementary_data.zip › ije-2020-05-0817-File014.docx]
