# Supplementary material for: Paternal body mass index and offspring DNA methylation: findings from the PACE consortium
Source: Int J Epidemiol. 2021 Jan 29;50(4):1297–315. doi: 10.1093/ije/dyaa267 (PMC8407864; doi:10.1093/ije/dyaa267)

**Meta-EWAS results quality checks: childhood**

We plotted QQ plots (page 2) and a correlation matrix (page 3) showing correlations of EWAS effect estimates for each model.

**EWAS models:**

**Covariates-adjusted: Paternal BMI + Covariates + Cells (model name: covs.pat)**

Methylation ~ Paternal BMI + SVs for batch + Paternal age + Paternal smoking status + Paternal socioeconomic status + Maternal age + Maternal smoking status + Parity + Estimated cell counts

**Covariates-adjusted: Maternal BMI + Covariates + Cells (model name: covs.mat)**

Methylation ~ Maternal BMI + SVs for batch + Paternal age + Paternal smoking status + Paternal socioeconomic status + Maternal age + Maternal smoking status + Parity + Estimated cell counts

**Covariates-adjusted: Paternal adjusted for maternal BMI, covariates and cells (model name: covs.patmat)**

Methylation ~ Paternal BMI + Maternal BMI + SVs for batch + Paternal age + Paternal smoking status + Paternal socioeconomic status + Maternal age + Maternal smoking status + Parity + Estimated cell counts

**Covariates-adjusted: Maternal adjusted for paternal BMI, covariates and cells (model name: covs.matpat)**

Methylation ~ Paternal BMI + Maternal BMI + SVs for batch + Paternal age + Paternal smoking status + Paternal socioeconomic status + Maternal age + Maternal smoking status + Parity + Estimated cell counts

**Sex-stratified: Paternal adjusted for maternal in males (model name: boys.patmat)**

Methylation ~ Paternal BMI + Maternal BMI + SVs for batch + Paternal age + Paternal smoking status + Paternal socioeconomic status + Maternal age + Maternal smoking status + Parity + Estimated cell counts

**Sex-stratified: Maternal adjusted for paternal in males (model name: boys.matpat)**

Methylation ~ Paternal BMI + Maternal BMI + SVs for batch + Paternal age + Paternal smoking status + Paternal socioeconomic status + Maternal age + Maternal smoking status + Parity + Estimated cell counts

**Sex-stratified: Paternal adjusted for maternal in males (model name: girls.patmat)**

Methylation ~ Paternal BMI + Maternal BMI + SVs for batch + Paternal age + Paternal smoking status + Paternal socioeconomic status + Maternal age + Maternal smoking status + Parity + Estimated cell counts

**Sex-stratified: Maternal adjusted for paternal in females(model name: girls.matpat)**

Methylation ~ Paternal BMI + Maternal BMI + SVs for batch + Paternal age + Paternal smoking status + Paternal socioeconomic status + Maternal age + Maternal smoking status + Parity + Estimated cell counts


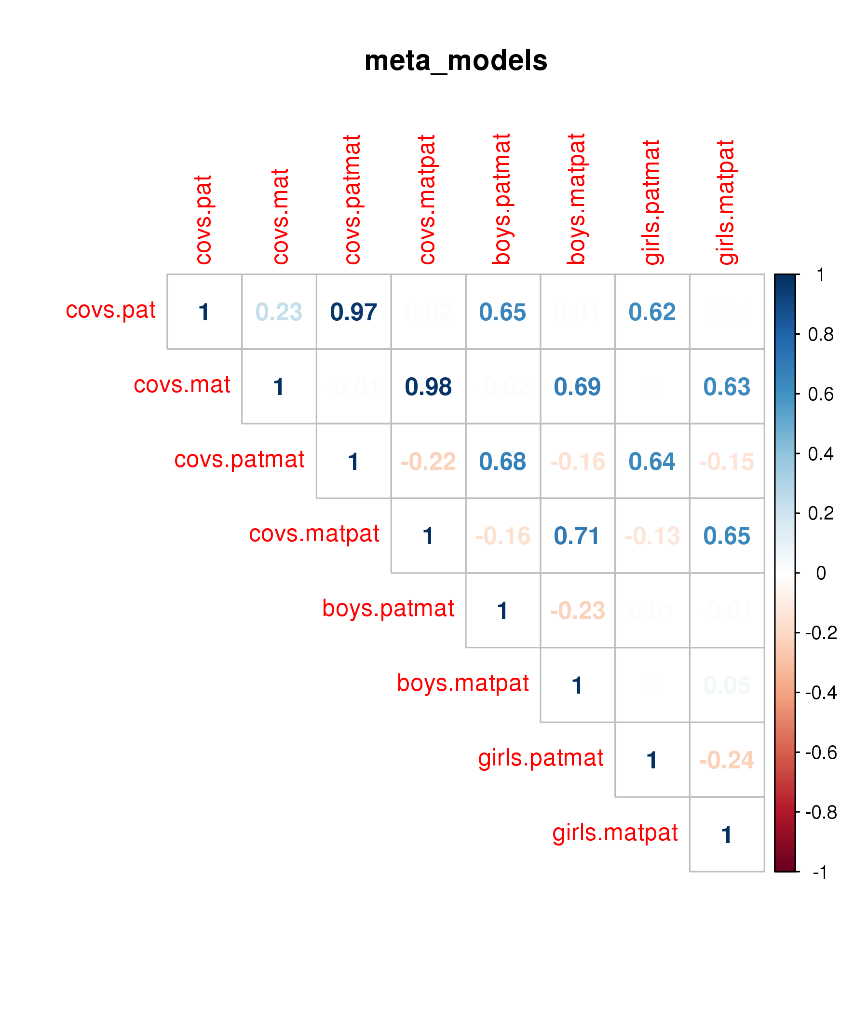


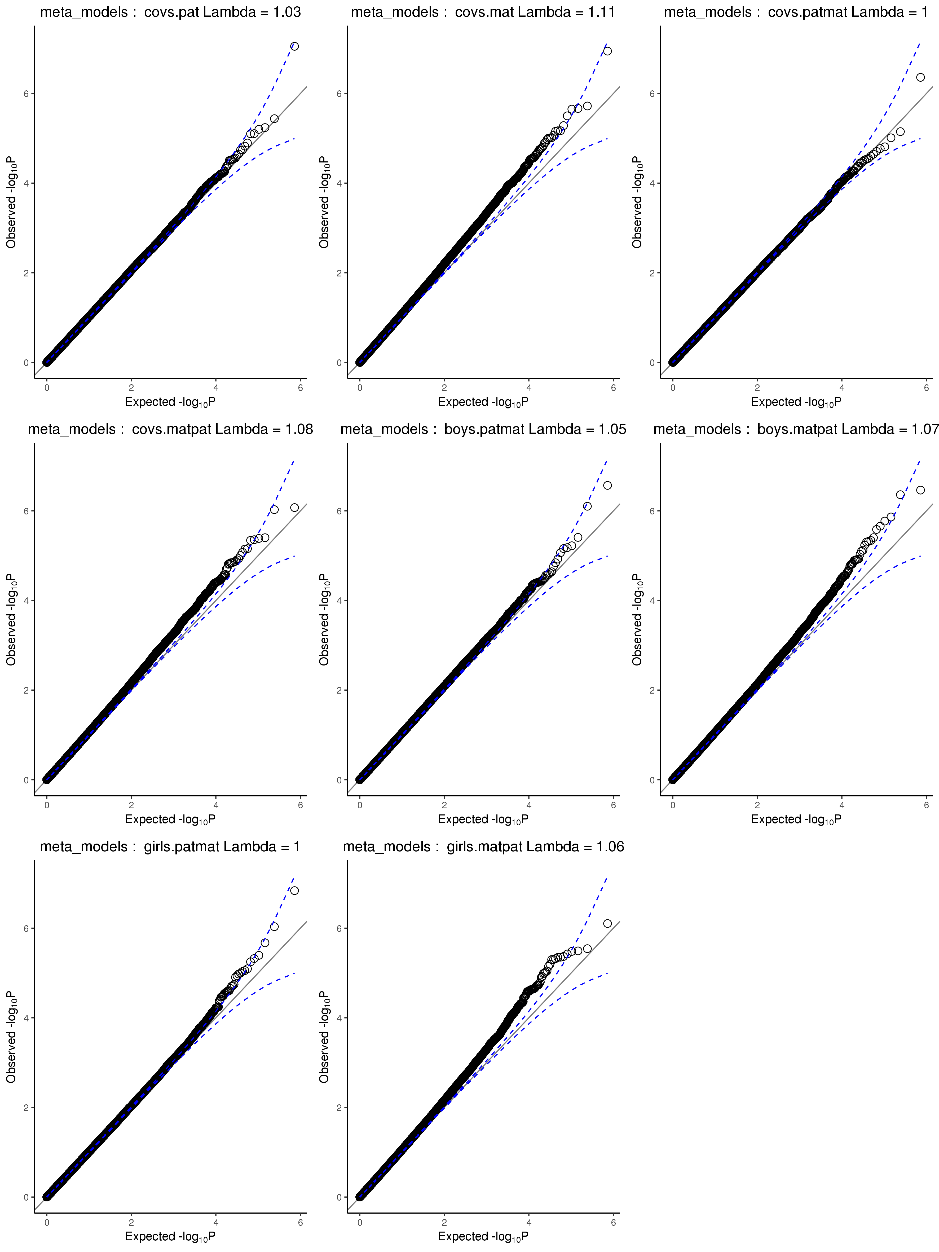

Supplement: dyaa267_Supplementary_Data [file dyaa267_supplementary_data.zip › ije-2020-05-0817-File015.docx]
