# Supplementary material for: Paternal body mass index and offspring DNA methylation: findings from the PACE consortium
Source: Int J Epidemiol. 2021 Jan 29;50(4):1297–315. doi: 10.1093/ije/dyaa267 (PMC8407864; doi:10.1093/ije/dyaa267)
Supplement: dyaa267_Supplementary_Data [file dyaa267_supplementary_data.zip › ije-2020-05-0817-File016.pdf]

Sensitivity analyses: childhood

For each model, at CpGs with P<1e-5, we conducted a leave-one-out analysis (systematically leaving one cohort at a time out of the meta-analysis) and plotted the resulting effect estimates and 95% confidence intervals (pages 3-22).
For the childhood timepoint, we also conducted a meta-regression to assess the strength of association between age at methylation sample on the meta-EWAS effect estimates. The coefficients, confidence intervals and p-values for these analyses are provided in the tables on pages 23-25

Contents:

Leave one out plots:

- Paternal BMI (unadjusted for maternal BMI): page 3
- Paternal BMI (adjusted for maternal BMI): page 5
- Paternal BMI adjusted for maternal BMI – female offspring only: pages 7-8
- Paternal BMI adjusted for maternal BMI – male offspring only: page 10

- Maternal BMI (unadjusted for paternal BMI): pages 12-13
- Maternal BMI (adjusted for paternal BMI): pages 15-16
- Maternal BMI adjusted for paternal BMI – female offspring only: pages 18-19
- Maternal BMI adjusted for paternal BMI – male offspring only: pages 21-22

- Meta-regression tables: page 23-25

Paternal BMI (unadjusted for maternal BMI): page 3

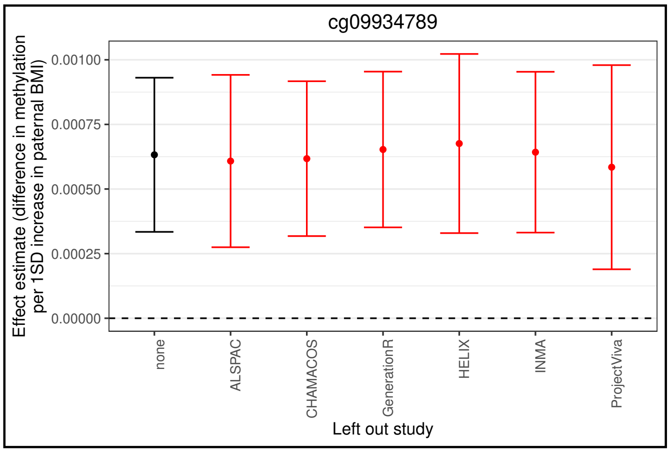
cg09934789
Effect estimate (difference in methylation per 1SD increase in paternal BMI)
0.00100
0.00075
0.00050
0.00025
0.00000
none
ALSPAC
CHAMACOS
GenerationR
HELIX
INMA
ProjectViva
Left out study

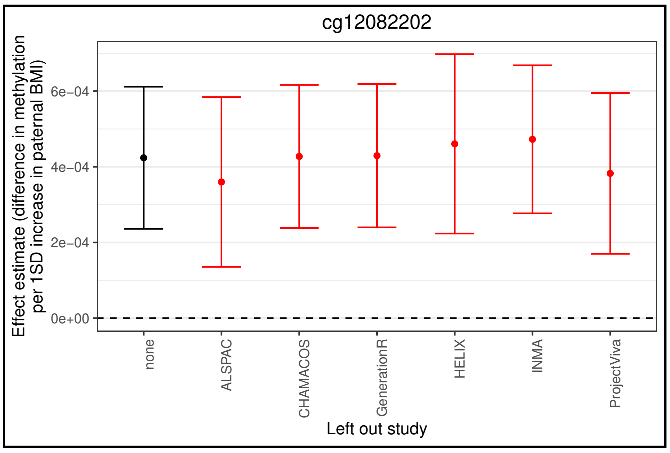
cg12082202
Effect estimate (difference in methylation per 1SD increase in paternal BMI)
6e−04
4e−04
2e−04
0e+00
none
ALSPAC
CHAMACOS
GenerationR
HELIX
INMA
ProjectViva
Left out study

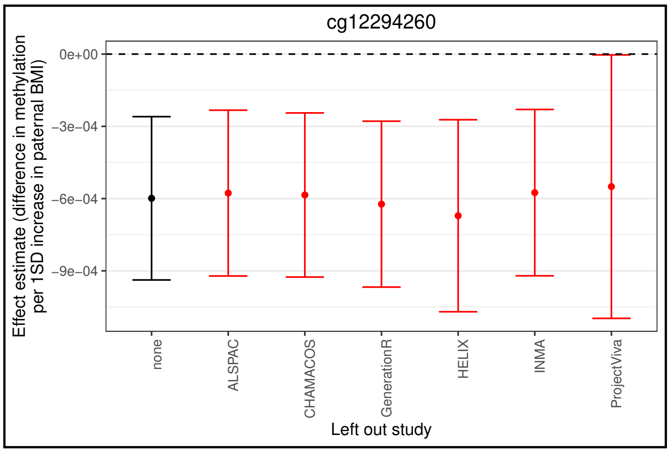
cg12294260
Effect estimate (difference in methylation per 1SD increase in paternal BMI)
0e+00
−3e−04
−6e−04
−9e−04
none
ALSPAC
CHAMACOS
GenerationR
HELIX
INMA
ProjectViva
Left out study

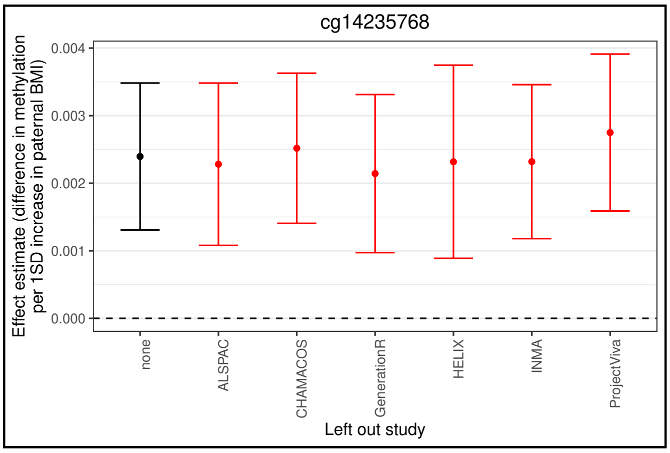
cg14235768
Effect estimate (difference in methylation per 1SD increase in paternal BMI)
0.004
0.003
0.002
0.001
0.000
none
ALSPAC
CHAMACOS
GenerationR
HELIX
INMA
ProjectViva
Left out study

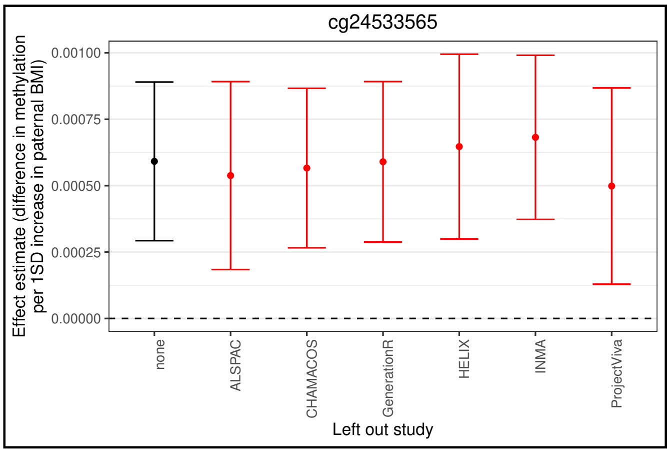
cg24533565
Effect estimate (difference in methylation per 1SD increase in paternal BMI)
0.00100
0.00075
0.00050
0.00025
0.00000
none
ALSPAC
CHAMACOS
GenerationR
HELIX
INMA
ProjectViva
Left out study

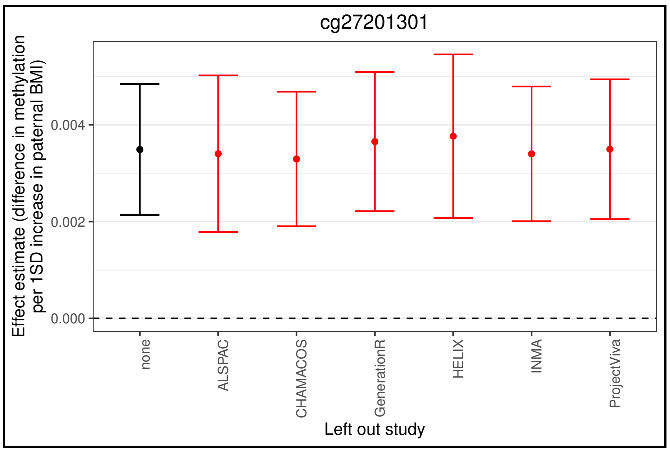
cg27201301
Effect estimate (difference in methylation per 1SD increase in paternal BMI)
0.004
0.002
0.000
none
ALSPAC
CHAMACOS
GenerationR
HELIX
INMA
ProjectViva
Left out study

Paternal BMI (adjusted for maternal BMI): page 5

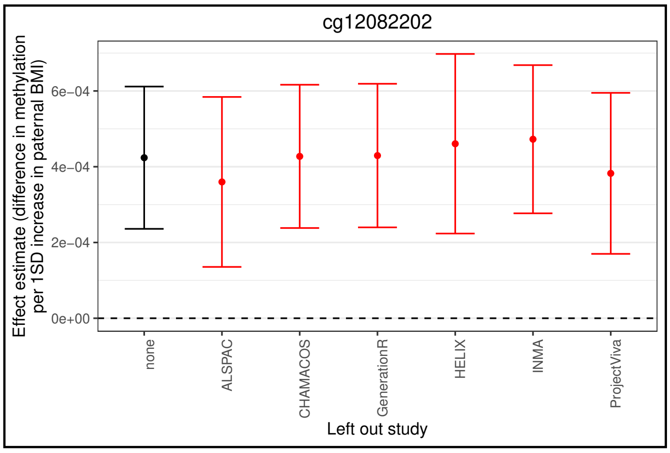
cg12082202
Effect estimate (difference in methylation per 1SD increase in paternal BMI)
6e−04
4e−04
2e−04
0e+00
none
ALSPAC
CHAMACOS
GenerationR
HELIX
INMA
ProjectViva
Left out study

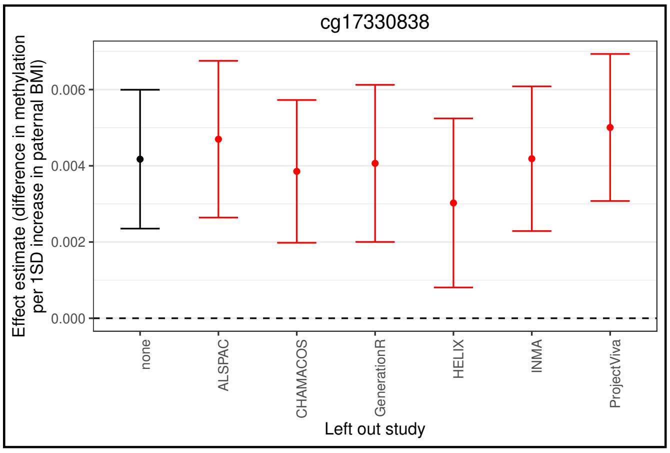
cg17330838
Effect estimate (difference in methylation per 1SD increase in paternal BMI)
0.006
0.004
0.002
0.000
none
ALSPAC
CHAMACOS
GenerationR
HELIX
INMA
ProjectViva
Left out study

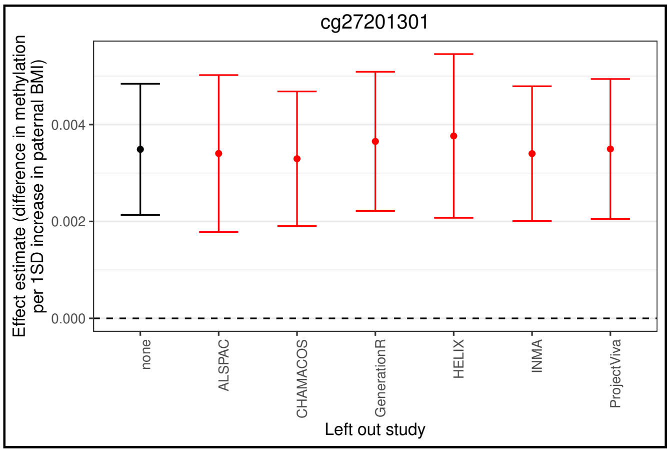
cg27201301
Effect estimate (difference in methylation per 1SD increase in paternal BMI)
0.004
0.002
0.000
none
ALSPAC
CHAMACOS
GenerationR
HELIX
INMA
ProjectViva
Left out study

Paternal BMI adjusted for maternal BMI – female offspring only: pages 7-8

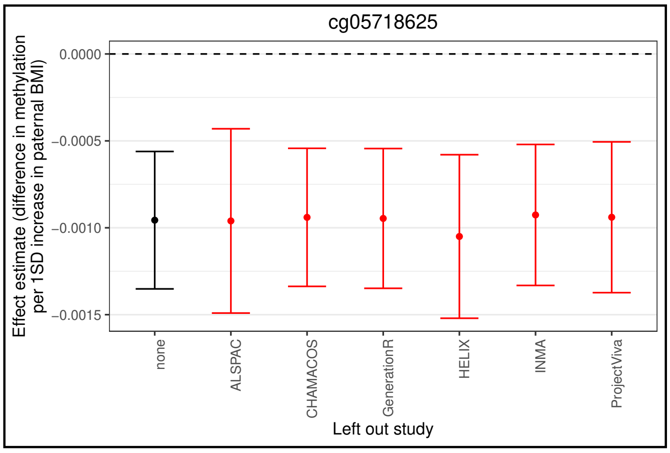
cg05718625
Effect estimate (difference in methylation per 1SD increase in paternal BMI)
0.0000
−0.0005
−0.0010
−0.0015
none
ALSPAC
CHAMACOS
GenerationR
HELIX
INMA
ProjectViva
Left out study

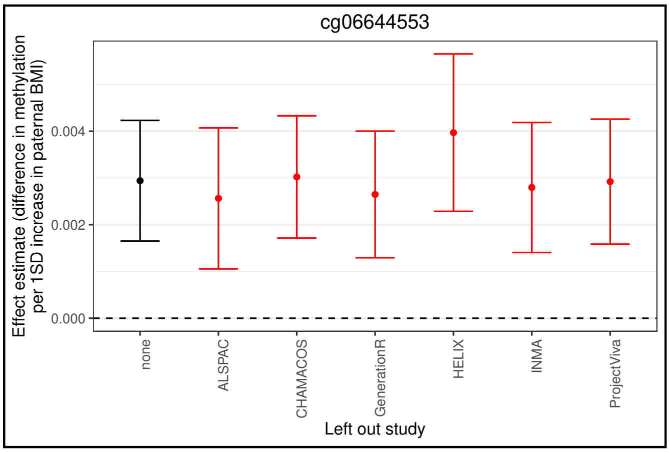
cg06644553
Effect estimate (difference in methylation per 1SD increase in paternal BMI)
0.004
0.002
0.000
none
ALSPAC
CHAMACOS
GenerationR
HELIX
INMA
ProjectViva
Left out study

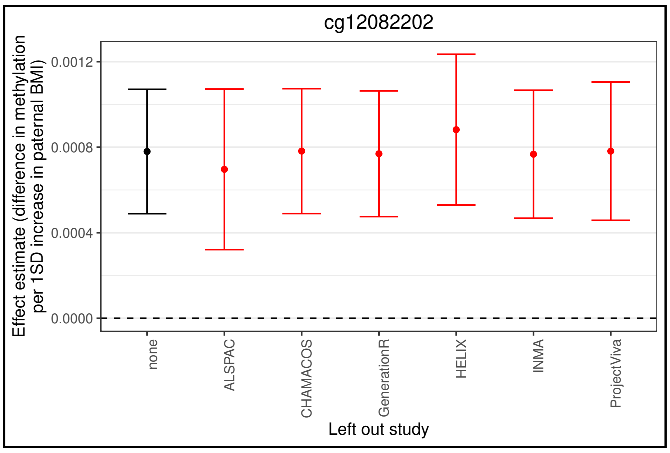
cg12082202
Effect estimate (difference in methylation per 1SD increase in paternal BMI)
0.0012
0.0008
0.0004
0.0000
none
ALSPAC
CHAMACOS
GenerationR
HELIX
INMA
ProjectViva
Left out study

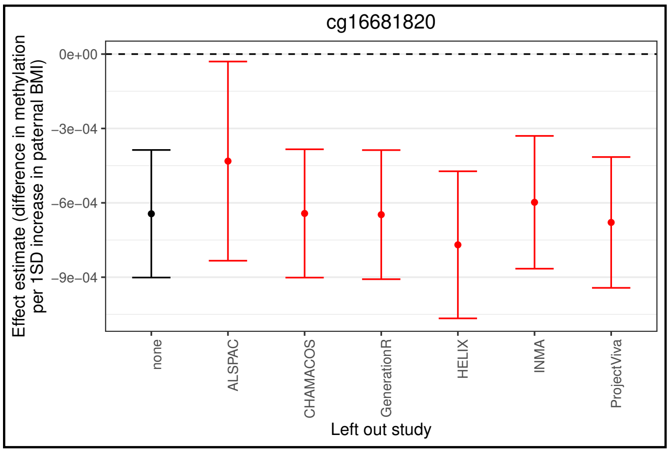
cg16681820
Effect estimate (difference in methylation per 1SD increase in paternal BMI)
0e+00
−3e−04
−6e−04
−9e−04
none
ALSPAC
CHAMACOS
GenerationR
HELIX
INMA
ProjectViva
Left out study

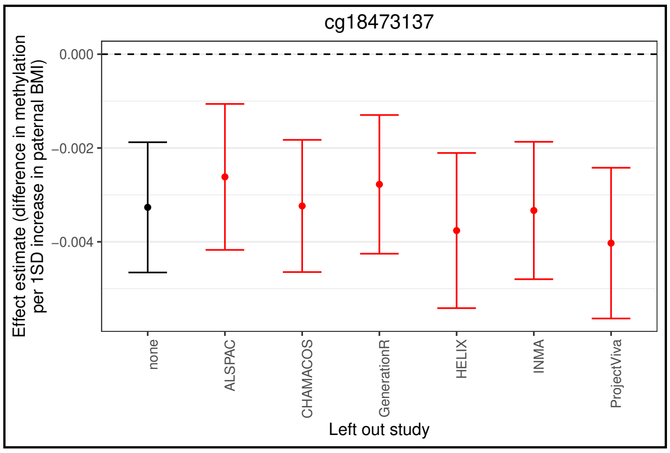
cg18473137
Effect estimate (difference in methylation per 1SD increase in paternal BMI)
0.000
−0.002
−0.004
none
ALSPAC
CHAMACOS
GenerationR
HELIX
INMA
ProjectViva
Left out study

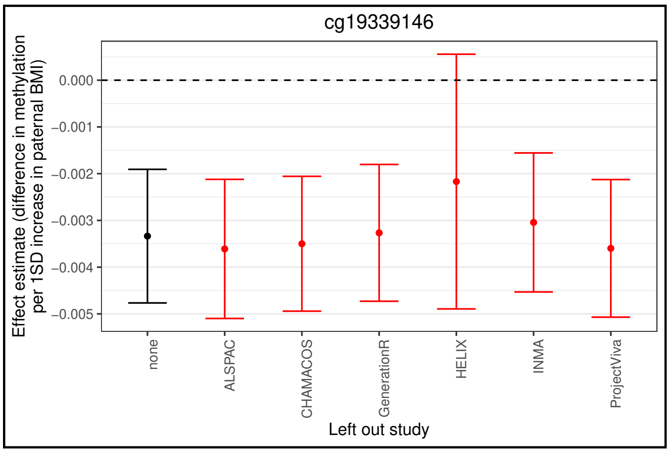
cg19339146
Effect estimate (difference in methylation per 1SD increase in paternal BMI)
0.000
−0.001
−0.002
−0.003
−0.004
−0.005
none
ALSPAC
CHAMACOS
GenerationR
HELIX
INMA
ProjectViva
Left out study

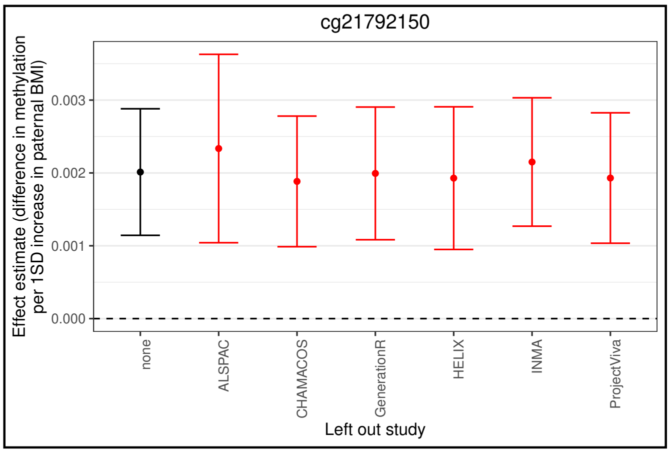
cg21792150
Effect estimate (difference in methylation per 1SD increase in paternal BMI)
0.003
0.002
0.001
0.000
none
ALSPAC
CHAMACOS
GenerationR
HELIX
INMA
ProjectViva
Left out study

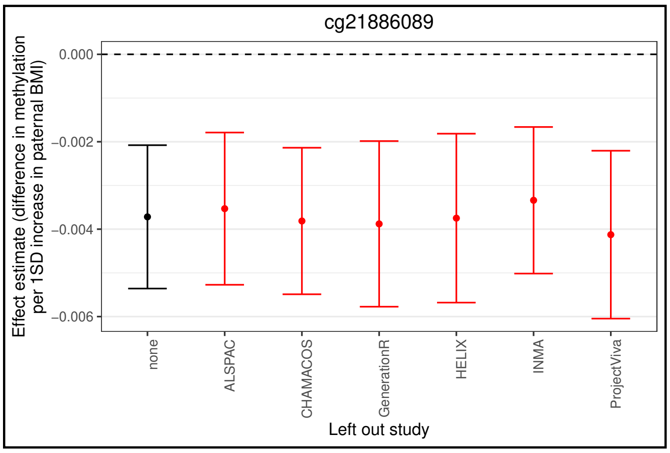
cg21886089
Effect estimate (difference in methylation per 1SD increase in paternal BMI)
0.000
−0.002
−0.004
−0.006
none
ALSPAC
CHAMACOS
GenerationR
HELIX
INMA
ProjectViva
Left out study

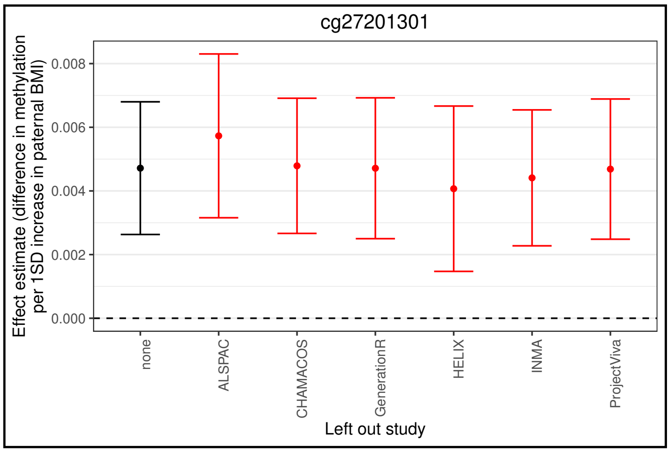
cg27201301
Effect estimate (difference in methylation per 1SD increase in paternal BMI)
0.008
0.006
0.004
0.002
0.000
none
ALSPAC
CHAMACOS
GenerationR
HELIX
INMA
ProjectViva
Left out study

Paternal BMI adjusted for maternal BMI – male offspring only: page 10

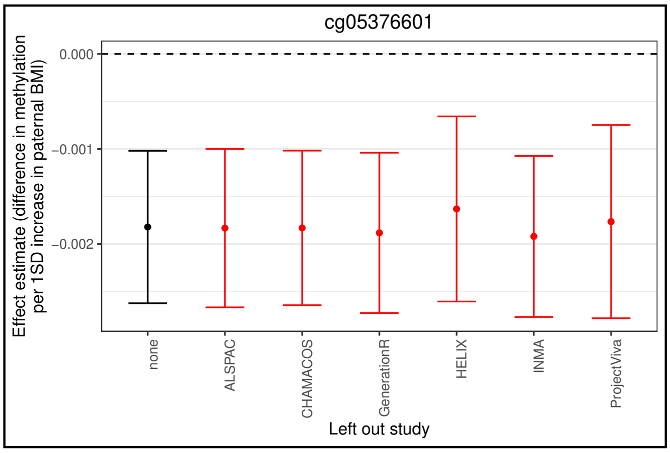
cg05376601
Effect estimate (difference in methylation per 1SD increase in paternal BMI)
0.000
−0.001
−0.002
none
ALSPAC
CHAMACOS
GenerationR
HELIX
INMA
ProjectViva
Left out study

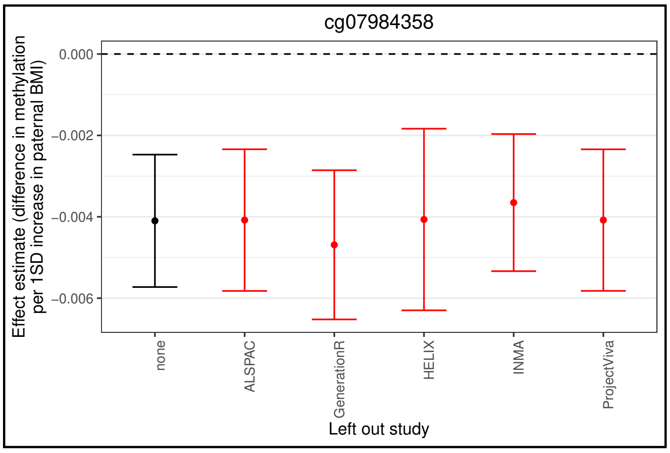
cg07984358
Effect estimate (difference in methylation per 1SD increase in paternal BMI)
0.000
−0.002
−0.004
−0.006
none
ALSPAC
GenerationR
HELIX
INMA
ProjectViva
Left out study

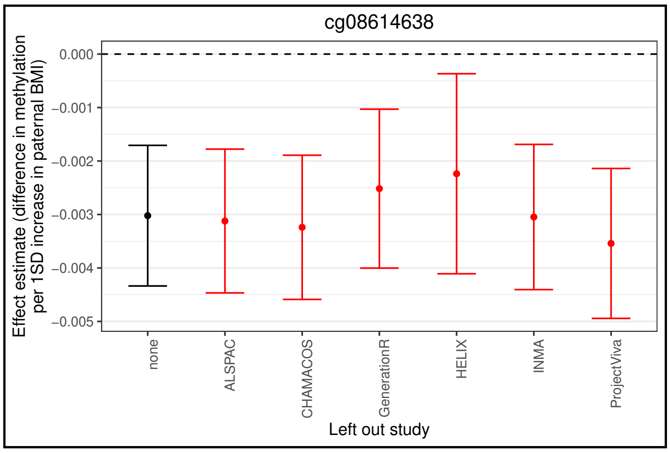
cg08614638
Effect estimate (difference in methylation per 1SD increase in paternal BMI)
0.000
−0.001
−0.002
−0.003
−0.004
−0.005
none
ALSPAC
CHAMACOS
GenerationR
HELIX
INMA
ProjectViva
Left out study

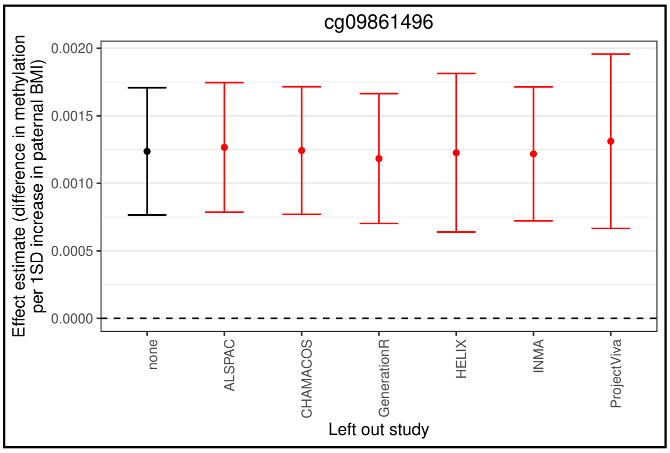
cg09861496
Effect estimate (difference in methylation per 1SD increase in paternal BMI)
0.0020
0.0015
0.0010
0.0005
0.0000
none
ALSPAC
CHAMACOS
GenerationR
HELIX
INMA
ProjectViva
Left out study

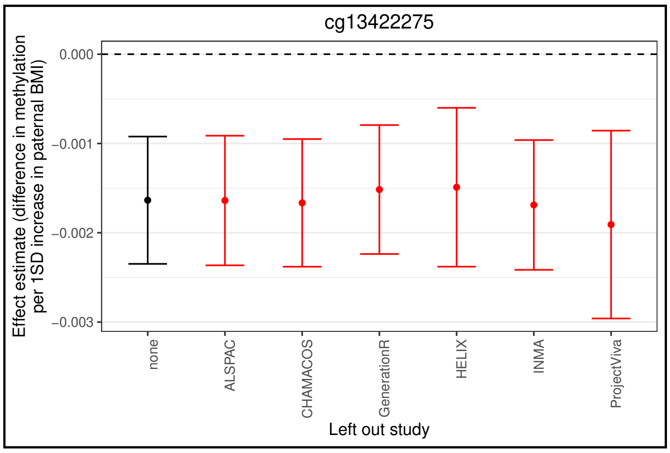
cg13422275
Effect estimate (difference in methylation per 1SD increase in paternal BMI)
0.000
−0.001
−0.002
−0.003
none
ALSPAC
CHAMACOS
GenerationR
HELIX
INMA
ProjectViva
Left out study

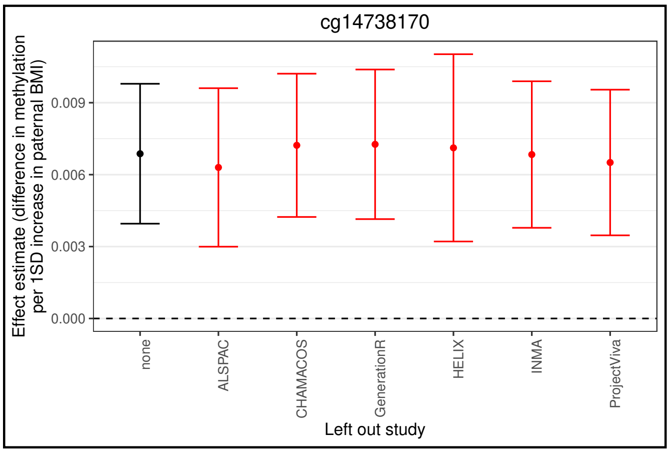
cg14738170
Effect estimate (difference in methylation per 1SD increase in paternal BMI)
0.009
0.006
0.003
0.000
none
ALSPAC
CHAMACOS
GenerationR
HELIX
INMA
ProjectViva
Left out study

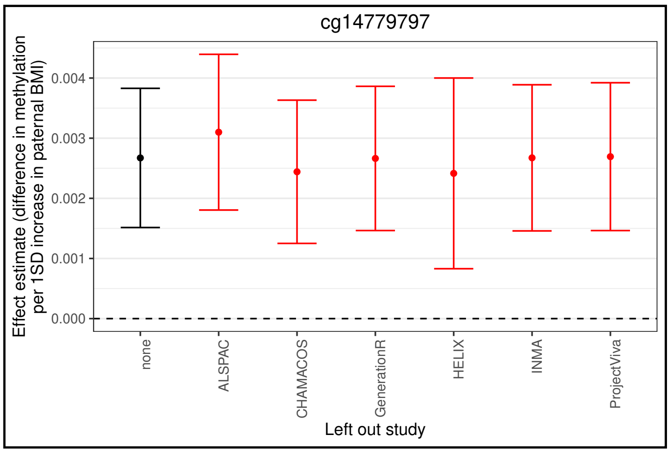
cg14779797
Effect estimate (difference in methylation per 1SD increase in paternal BMI)
0.004
0.003
0.002
0.001
0.000
none
ALSPAC
CHAMACOS
GenerationR
HELIX
INMA
ProjectViva
Left out study

Maternal BMI (unadjusted for paternal BMI): pages 12-13

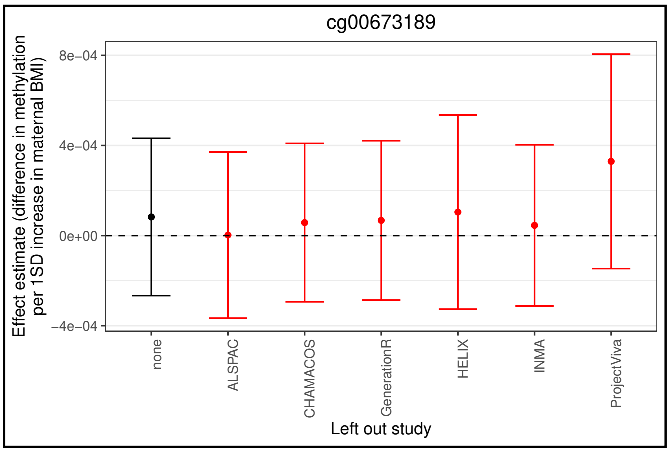
cg00673189
Effect estimate (difference in methylation per 1SD increase in maternal BMI)
8e−04
4e−04
0e+00
−4e−04
none
ALSPAC
CHAMACOS
GenerationR
HELIX
INMA
ProjectViva
Left out study

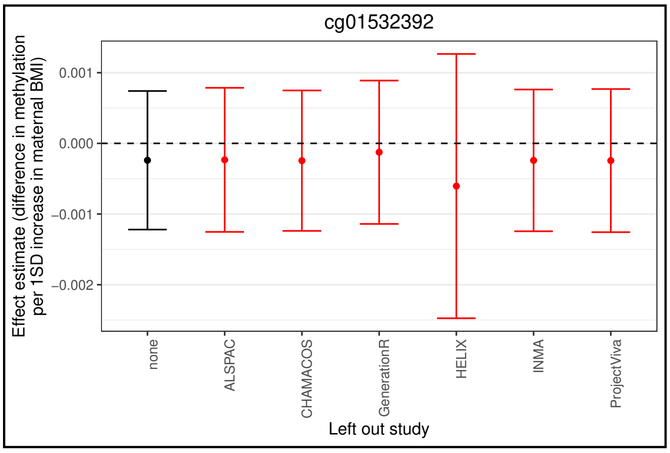
cg01532392
Effect estimate (difference in methylation per 1SD increase in maternal BMI)
0.001
0.000
−0.001
−0.002
none
ALSPAC
CHAMACOS
GenerationR
HELIX
INMA
ProjectViva
Left out study

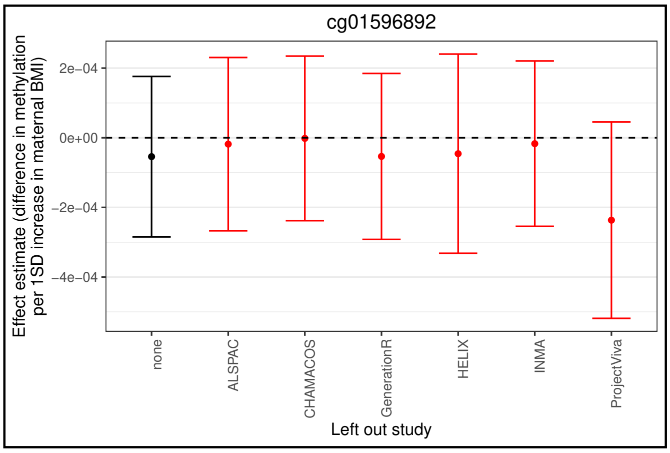
cg01596892
Effect estimate (difference in methylation per 1SD increase in maternal BMI)
2e−04
0e+00
−2e−04
−4e−04
none
ALSPAC
CHAMACOS
GenerationR
HELIX
INMA
ProjectViva
Left out study

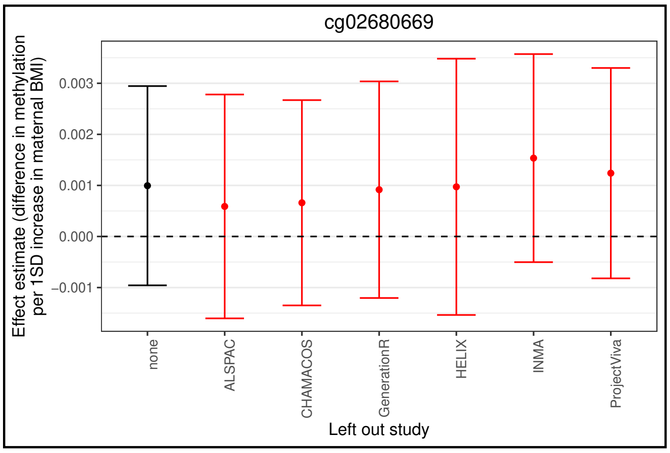
cg02680669
Effect estimate (difference in methylation per 1SD increase in maternal BMI)
0.003
0.002
0.001
0.000
−0.001
none
ALSPAC
CHAMACOS
GenerationR
HELIX
INMA
ProjectViva
Left out study

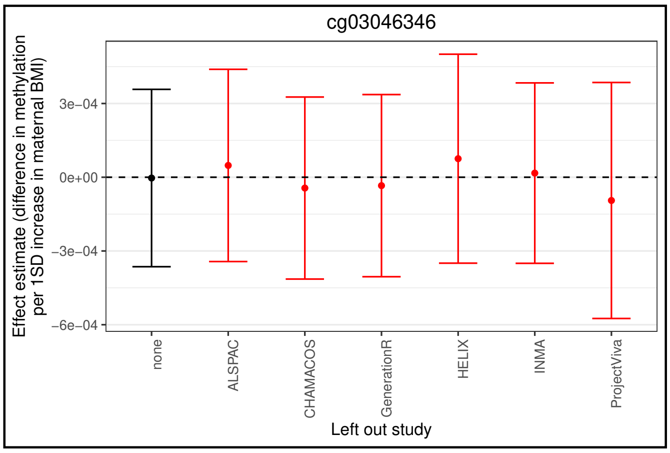
cg03046346
Effect estimate (difference in methylation per 1SD increase in maternal BMI)
3e−04
0e+00
−3e−04
−6e−04
none
ALSPAC
CHAMACOS
GenerationR
HELIX
INMA
ProjectViva
Left out study

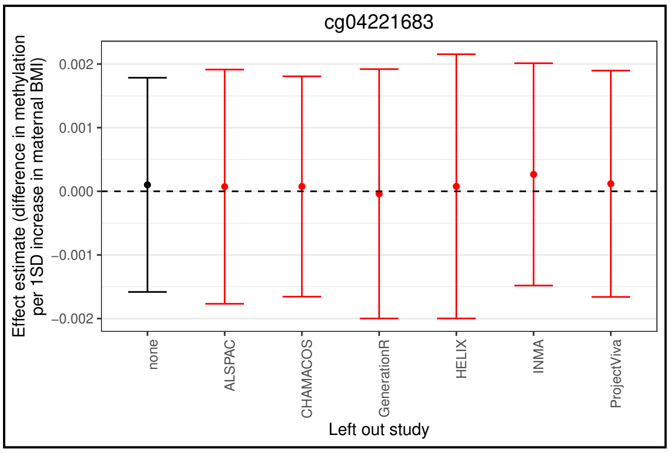
cg04221683
Effect estimate (difference in methylation per 1SD increase in maternal BMI)
0.002
0.001
0.000
−0.001
−0.002
none
ALSPAC
CHAMACOS
GenerationR
HELIX
INMA
ProjectViva
Left out study

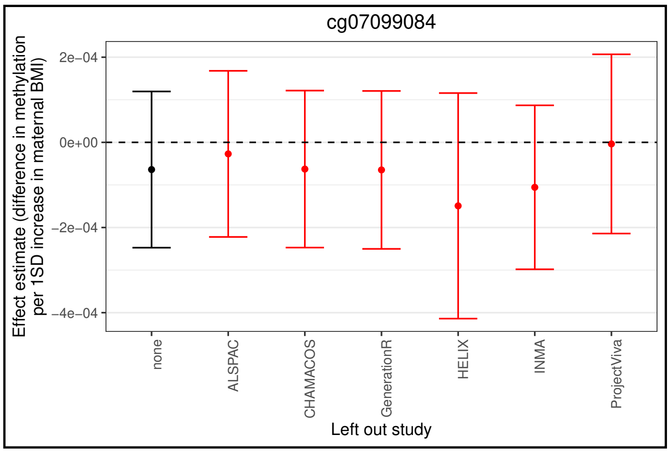
cg07099084
Effect estimate (difference in methylation per 1SD increase in maternal BMI)
2e−04
0e+00
−2e−04
−4e−04
none
ALSPAC
CHAMACOS
GenerationR
HELIX
INMA
ProjectViva
Left out study

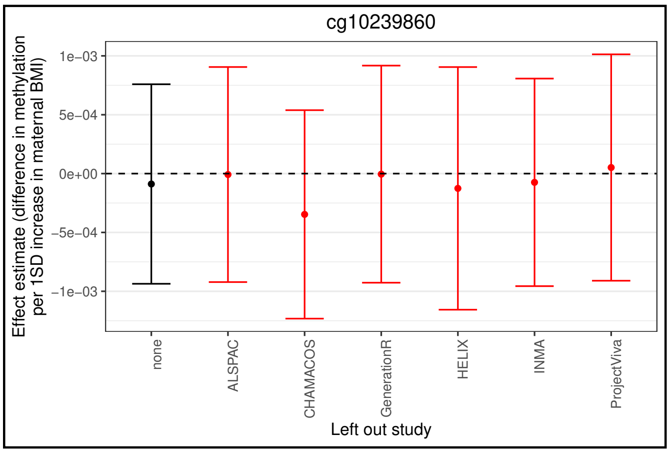
cg10239860
Effect estimate (difference in methylation per 1SD increase in maternal BMI)
1e−03
5e−04
0e+00
−5e−04
−1e−03
none
ALSPAC
CHAMACOS
GenerationR
HELIX
INMA
ProjectViva
Left out study

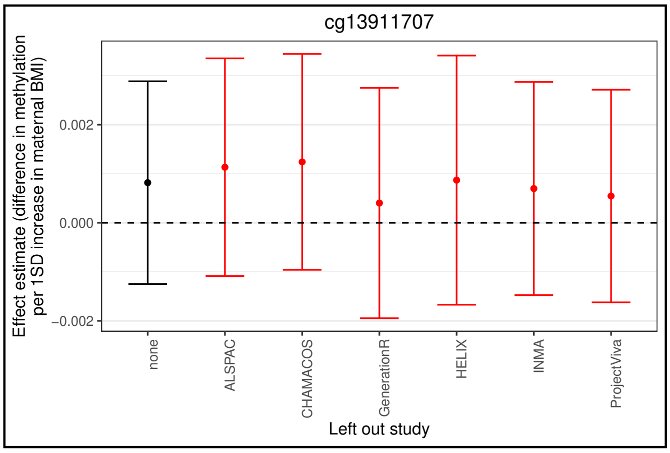
cg13911707
Effect estimate (difference in methylation per 1SD increase in maternal BMI)
0.002
0.000
−0.002
none
ALSPAC
CHAMACOS
GenerationR
HELIX
INMA
ProjectViva
Left out study

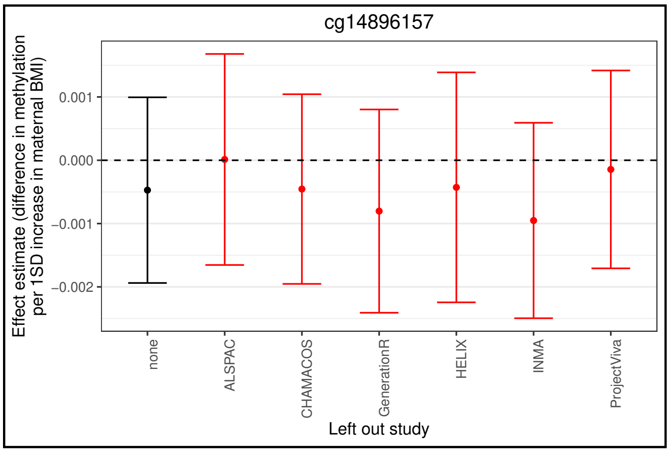
cg14896157
Effect estimate (difference in methylation per 1SD increase in maternal BMI)
0.001
0.000
−0.001
−0.002
none
ALSPAC
CHAMACOS
GenerationR
HELIX
INMA
ProjectViva
Left out study

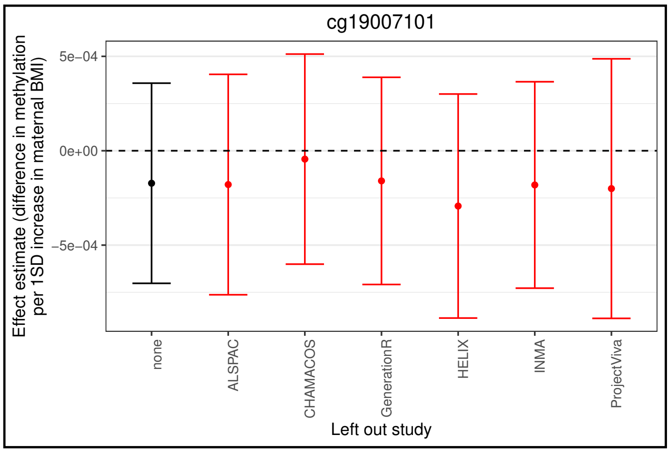
cg19007101
Effect estimate (difference in methylation per 1SD increase in maternal BMI)
5e−04
0e+00
−5e−04
none
ALSPAC
CHAMACOS
GenerationR
HELIX
INMA
ProjectViva
Left out study

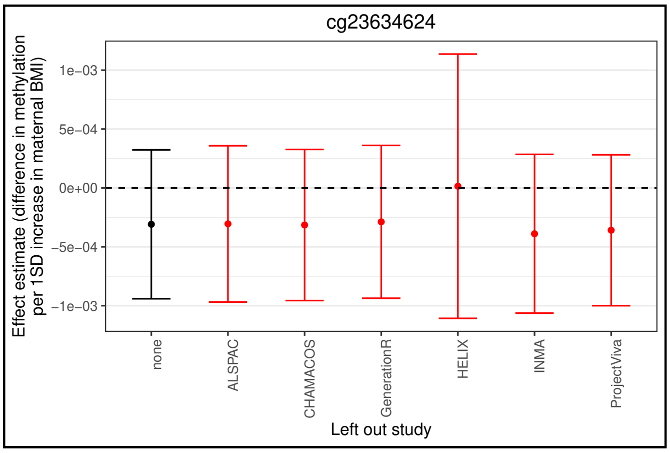
cg23634624
Effect estimate (difference in methylation per 1SD increase in maternal BMI)
1e−03
5e−04
0e+00
−5e−04
−1e−03
none
ALSPAC
CHAMACOS
GenerationR
HELIX
INMA
ProjectViva
Left out study

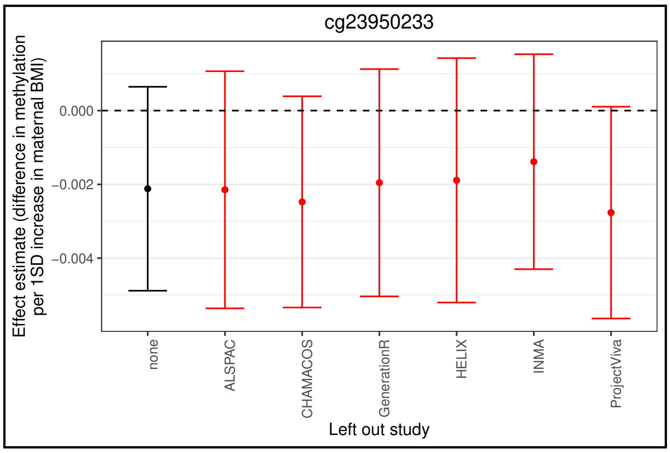
cg23950233
Effect estimate (difference in methylation per 1SD increase in maternal BMI)
0.000
−0.002
−0.004
none
ALSPAC
CHAMACOS
GenerationR
HELIX
INMA
ProjectViva
Left out study

Maternal BMI (adjusted for paternal BMI): pages 15-16

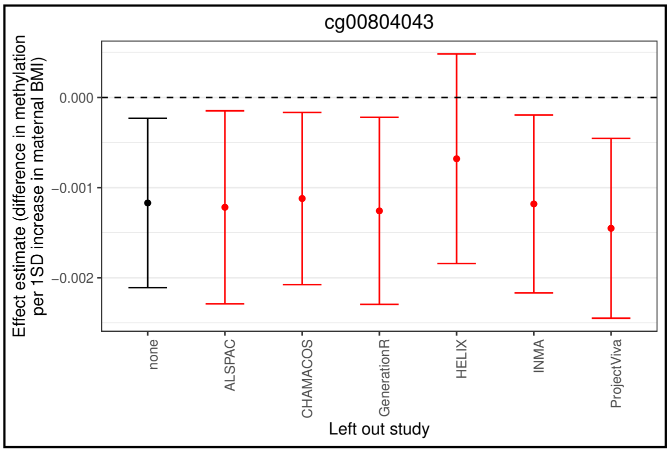
cg00804043
Effect estimate (difference in methylation per 1SD increase in maternal BMI)
0.000
−0.001
−0.002
none
ALSPAC
CHAMACOS
GenerationR
HELIX
INMA
ProjectViva
Left out study

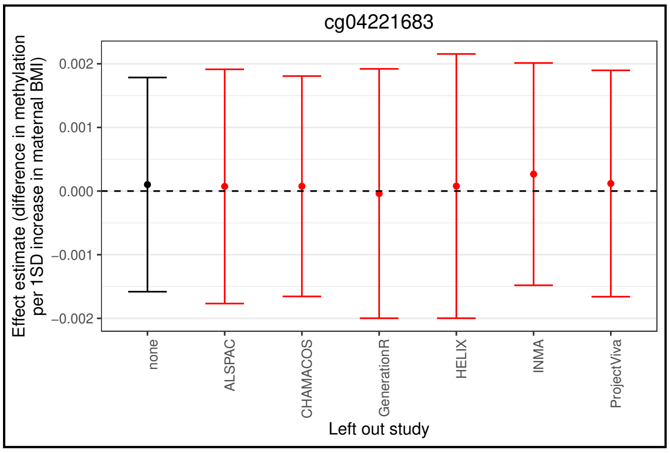
cg04221683
Effect estimate (difference in methylation per 1SD increase in maternal BMI)
0.002
0.001
0.000
−0.001
−0.002
none
ALSPAC
CHAMACOS
GenerationR
HELIX
INMA
ProjectViva
Left out study

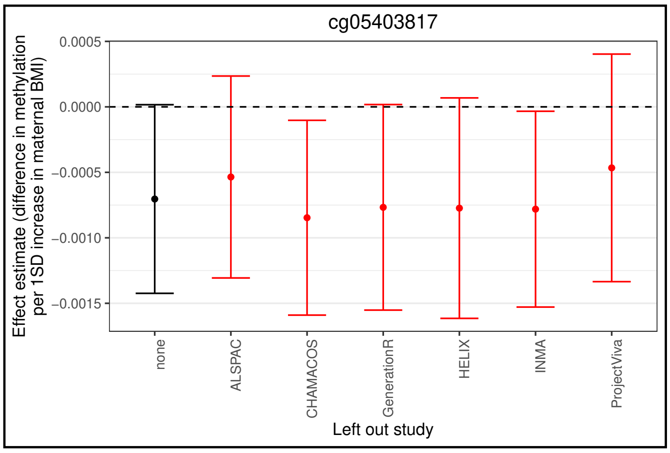
cg05403817
Effect estimate (difference in methylation per 1SD increase in maternal BMI)
0.0005
0.0000
−0.0005
−0.0010
−0.0015
none
ALSPAC
CHAMACOS
GenerationR
HELIX
INMA
ProjectViva
Left out study

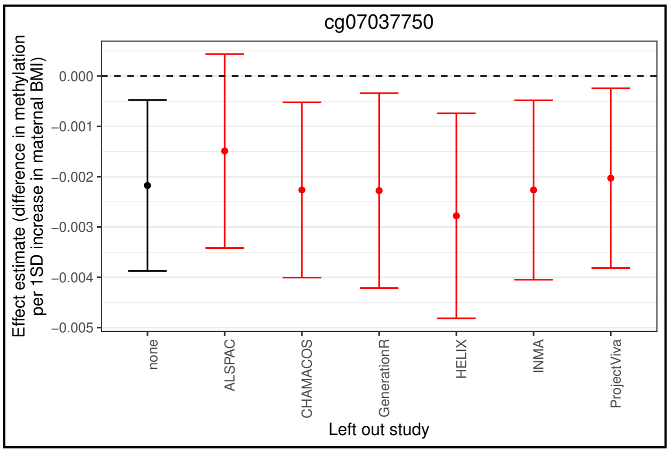
cg07037750
Effect estimate (difference in methylation per 1SD increase in maternal BMI)
0.000
−0.001
−0.002
−0.003
−0.004
−0.005
none
ALSPAC
CHAMACOS
GenerationR
HELIX
INMA
ProjectViva
Left out study

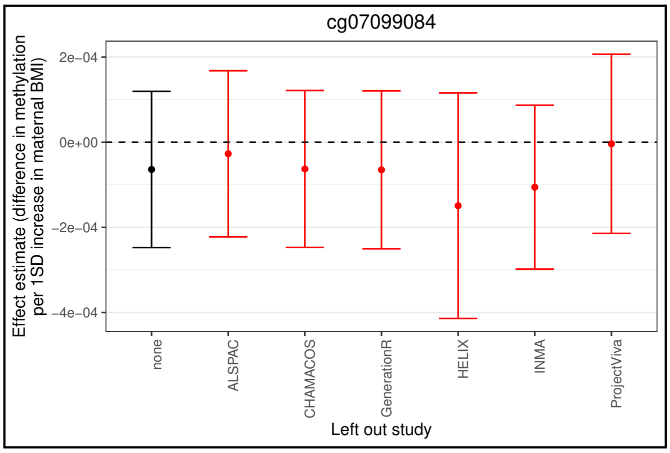
cg07099084
Effect estimate (difference in methylation per 1SD increase in maternal BMI)
2e−04
0e+00
−2e−04
−4e−04
none
ALSPAC
CHAMACOS
GenerationR
HELIX
INMA
ProjectViva
Left out study

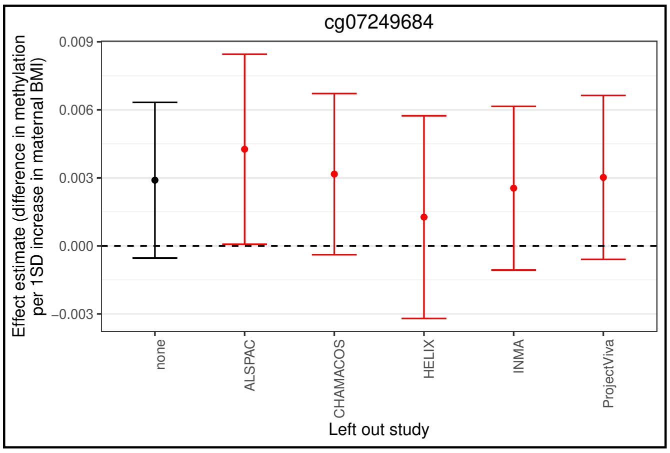
cg07249684
Effect estimate (difference in methylation per 1SD increase in maternal BMI)
0.009
0.006
0.003
0.000
−0.003
none
ALSPAC
CHAMACOS
HELIX
INMA
ProjectViva
Left out study

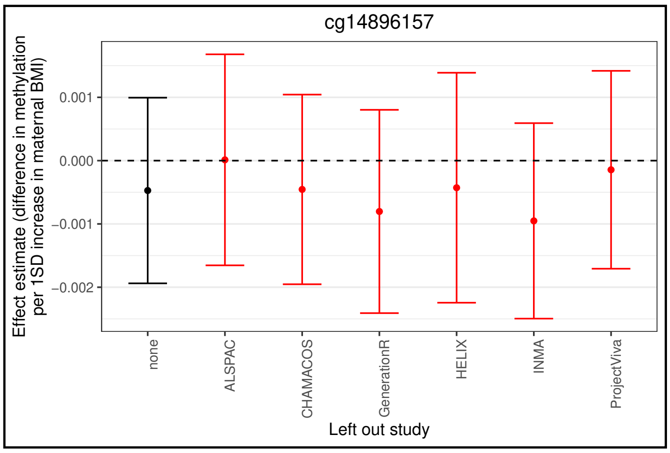
cg14896157
Effect estimate (difference in methylation per 1SD increase in maternal BMI)
0.001
0.000
−0.001
−0.002
none
ALSPAC
CHAMACOS
GenerationR
HELIX
INMA
ProjectViva
Left out study

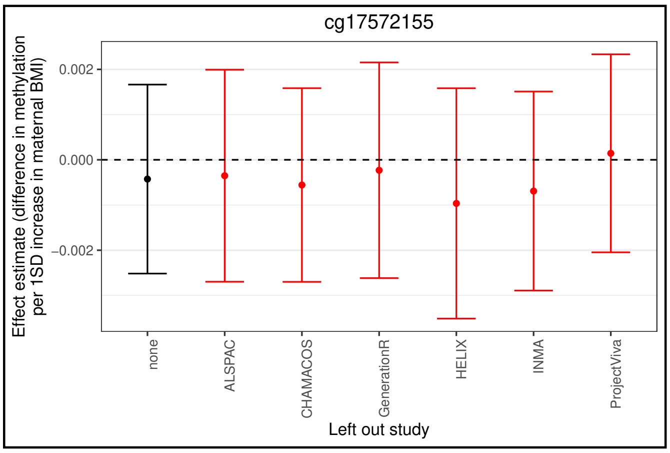
cg17572155
Effect estimate (difference in methylation per 1SD increase in maternal BMI)
0.002
0.000
−0.002
none
ALSPAC
CHAMACOS
GenerationR
HELIX
INMA
ProjectViva
Left out study

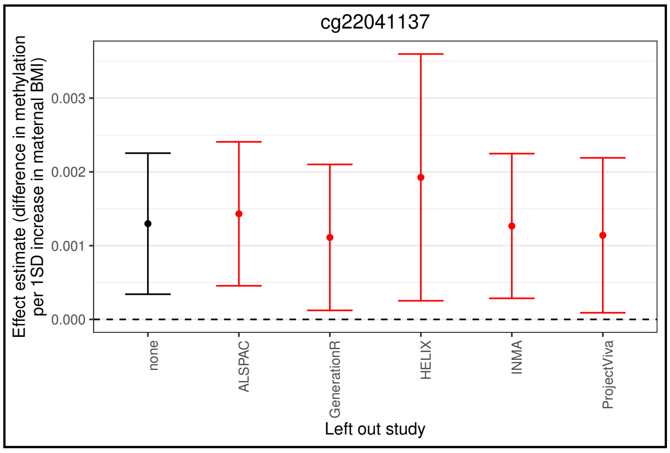
cg22041137
Effect estimate (difference in methylation per 1SD increase in maternal BMI)
0.003
0.002
0.001
0.000
none
ALSPAC
GenerationR
HELIX
INMA
ProjectViva
Left out study

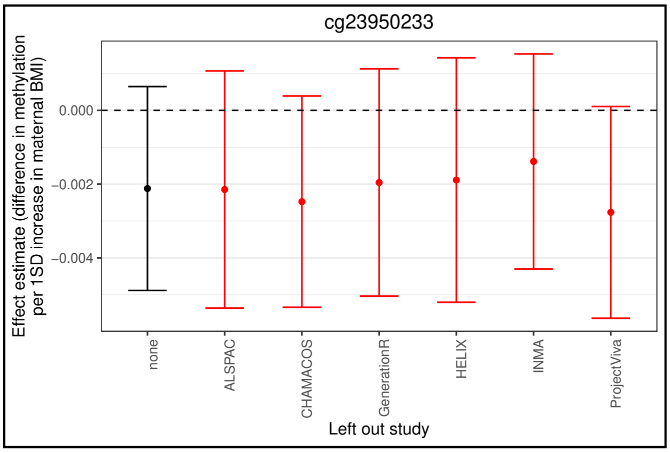
cg23950233
Effect estimate (difference in methylation per 1SD increase in maternal BMI)
0.000
−0.002
−0.004
none
ALSPAC
CHAMACOS
GenerationR
HELIX
INMA
ProjectViva
Left out study

Maternal BMI adjusted for paternal BMI – female offspring only:
pages 18-19

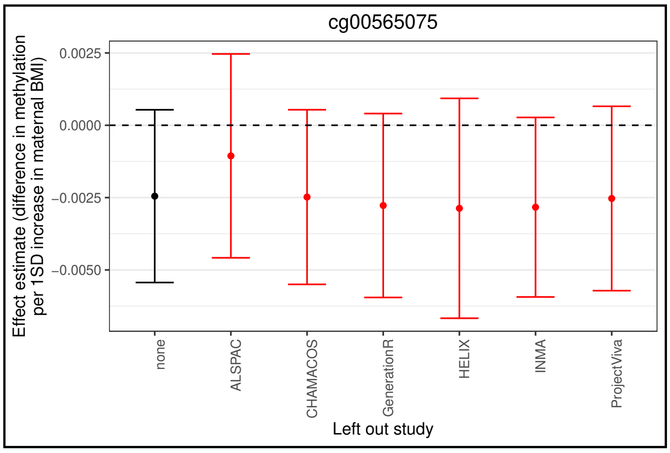
cg00565075
Effect estimate (difference in methylation per 1SD increase in maternal BMI)
0.0025
0.0000
−0.0025
−0.0050
none
ALSPAC
CHAMACOS
GenerationR
HELIX
INMA
ProjectViva
Left out study

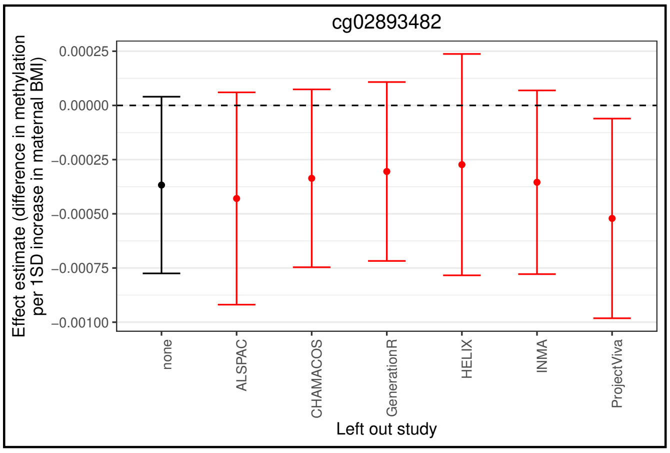
cg02893482
Effect estimate (difference in methylation per 1SD increase in maternal BMI)
0.00025
0.00000
−0.00025
−0.00050
−0.00075
−0.00100
none
ALSPAC
CHAMACOS
GenerationR
HELIX
INMA
ProjectViva
Left out study

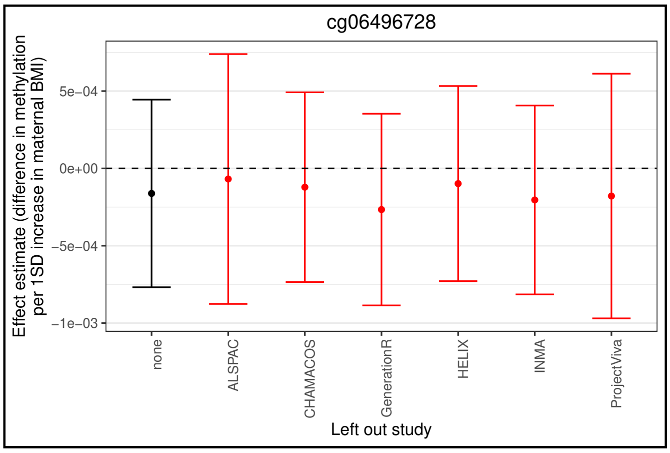
cg06496728
Effect estimate (difference in methylation per 1SD increase in maternal BMI)
5e−04
0e+00
−5e−04
−1e−03
none
ALSPAC
CHAMACOS
GenerationR
HELIX
INMA
ProjectViva
Left out study

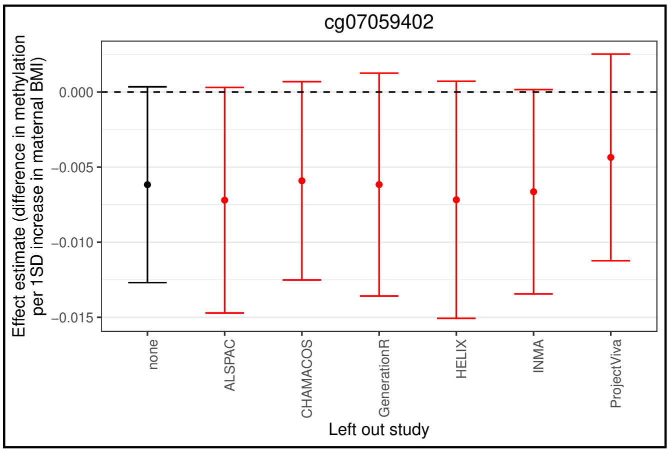
cg07059402
Effect estimate (difference in methylation per 1SD increase in maternal BMI)
0.000
−0.005
−0.010
−0.015
none
ALSPAC
CHAMACOS
GenerationR
HELIX
INMA
ProjectViva
Left out study

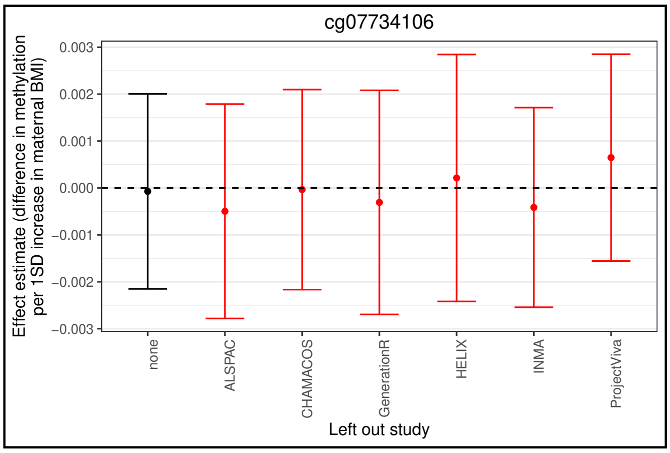
cg07734106
Effect estimate (difference in methylation per 1SD increase in maternal BMI)
0.003
0.002
0.001
0.000
−0.001
−0.002
−0.003
none
ALSPAC
CHAMACOS
GenerationR
HELIX
INMA
ProjectViva
Left out study

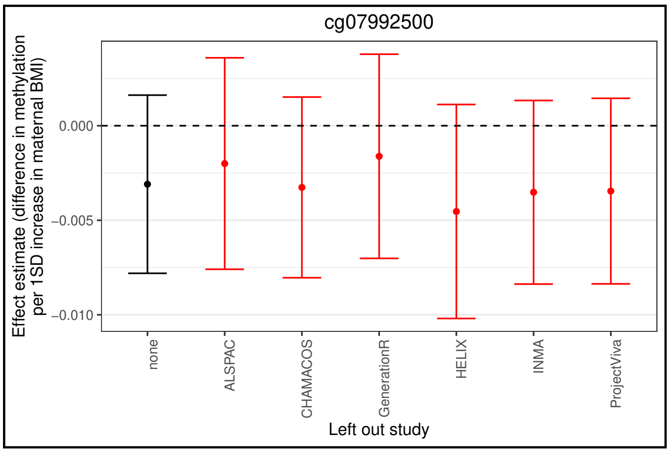
cg07992500
Effect estimate (difference in methylation per 1SD increase in maternal BMI)
0.000
−0.005
−0.010
none
ALSPAC
CHAMACOS
GenerationR
HELIX
INMA
ProjectViva
Left out study

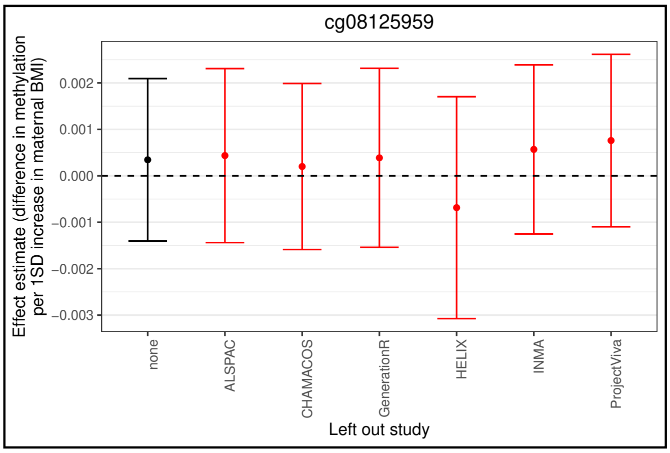
cg08125959
Effect estimate (difference in methylation per 1SD increase in maternal BMI)
0.002
0.001
0.000
−0.001
−0.002
−0.003
none
ALSPAC
CHAMACOS
GenerationR
HELIX
INMA
ProjectViva
Left out study

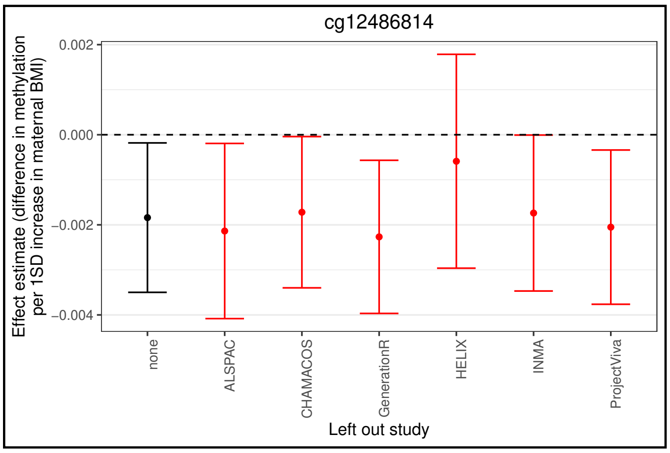
cg12486814
Effect estimate (difference in methylation per 1SD increase in maternal BMI)
0.002
0.000
−0.002
−0.004
none
ALSPAC
CHAMACOS
GenerationR
HELIX
INMA
ProjectViva
Left out study

Page 18 of 25

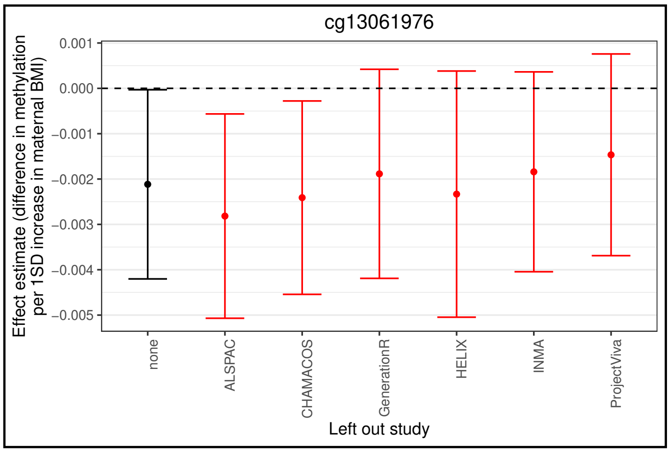
cg13061976
Effect estimate (difference in methylation per 1SD increase in maternal BMI)
0.001
0.000
−0.001
−0.002
−0.003
−0.004
−0.005
none
ALSPAC
CHAMACOS
GenerationR
HELIX
INMA
ProjectViva
Left out study

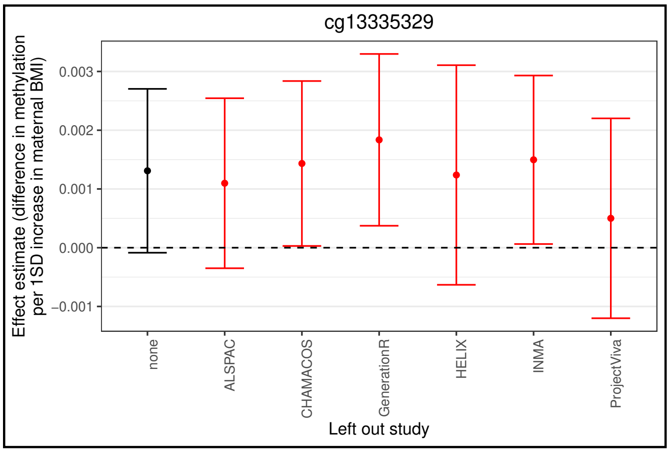
cg13335329
Effect estimate (difference in methylation per 1SD increase in maternal BMI)
0.003
0.002
0.001
0.000
−0.001
none
ALSPAC
CHAMACOS
GenerationR
HELIX
INMA
ProjectViva
Left out study

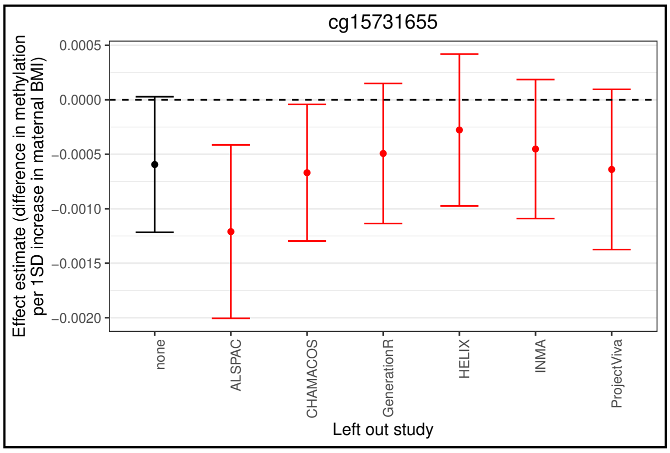
cg15731655
Effect estimate (difference in methylation per 1SD increase in maternal BMI)
0.0005
0.0000
−0.0005
−0.0010
−0.0015
−0.0020
none
ALSPAC
CHAMACOS
GenerationR
HELIX
INMA
ProjectViva
Left out study

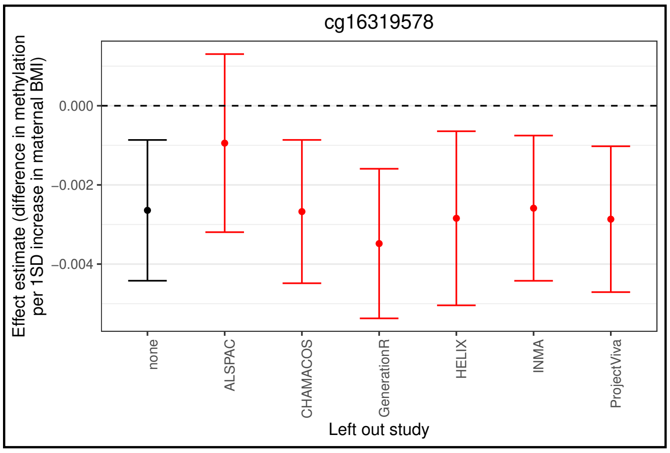
cg16319578
Effect estimate (difference in methylation per 1SD increase in maternal BMI)
0.000
−0.002
−0.004
none
ALSPAC
CHAMACOS
GenerationR
HELIX
INMA
ProjectViva
Left out study

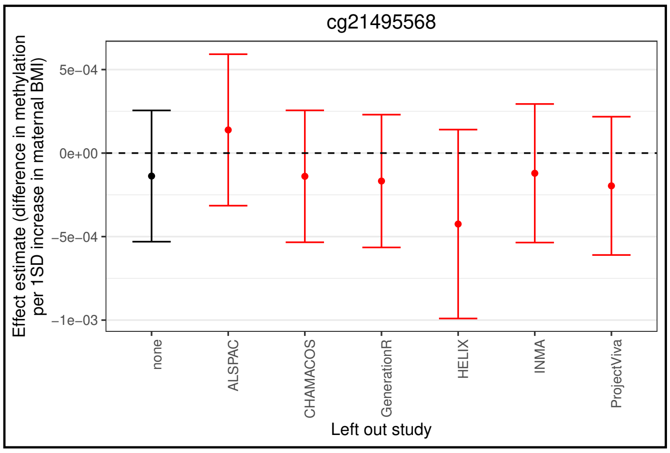
cg21495568
Effect estimate (difference in methylation per 1SD increase in maternal BMI)
5e−04
0e+00
−5e−04
−1e−03
none
ALSPAC
CHAMACOS
GenerationR
HELIX
INMA
ProjectViva
Left out study

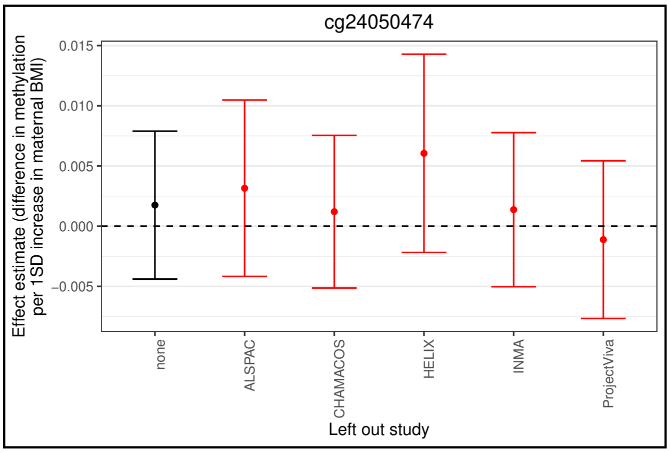
cg24050474
Effect estimate (difference in methylation per 1SD increase in maternal BMI)
0.015
0.010
0.005
0.000
−0.005
none
ALSPAC
CHAMACOS
HELIX
INMA
ProjectViva
Left out study

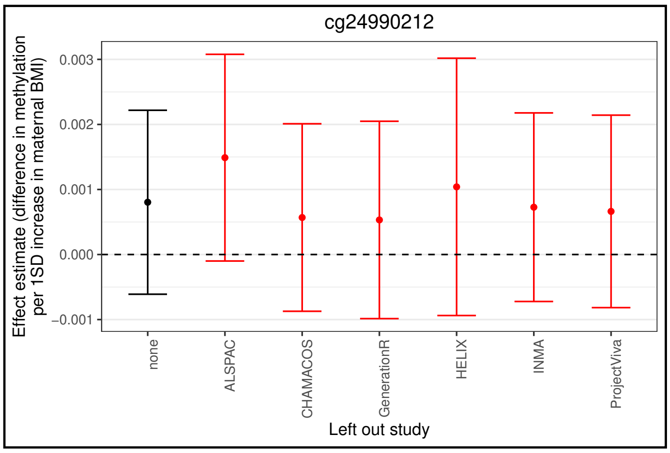
cg24990212
Effect estimate (difference in methylation per 1SD increase in maternal BMI)
0.003
0.002
0.001
0.000
−0.001
none
ALSPAC
CHAMACOS
GenerationR
HELIX
INMA
ProjectViva
Left out study

Maternal BMI adjusted for paternal BMI – male offspring only: pages 21-22

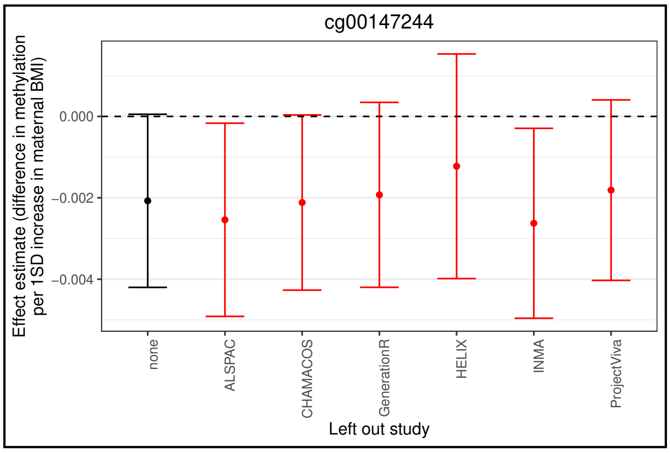
cg00147244
Effect estimate (difference in methylation per 1SD increase in maternal BMI)
0.000
−0.002
−0.004
none
ALSPAC
CHAMACOS
GenerationR
HELIX
INMA
ProjectViva
Left out study

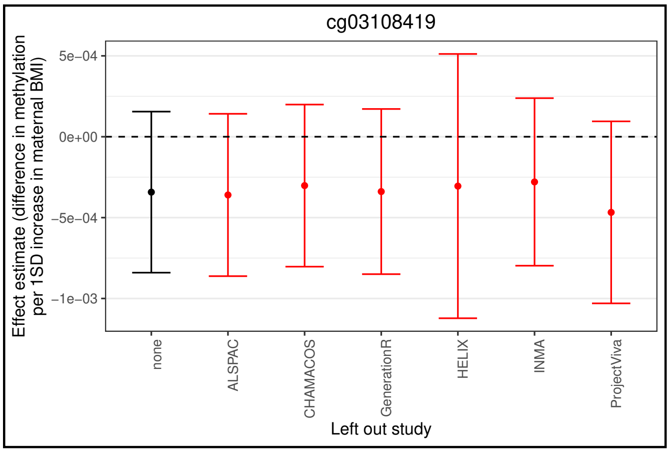
cg03108419
Effect estimate (difference in methylation per 1SD increase in maternal BMI)
5e−04
0e+00
−5e−04
−1e−03
none
ALSPAC
CHAMACOS
GenerationR
HELIX
INMA
ProjectViva
Left out study

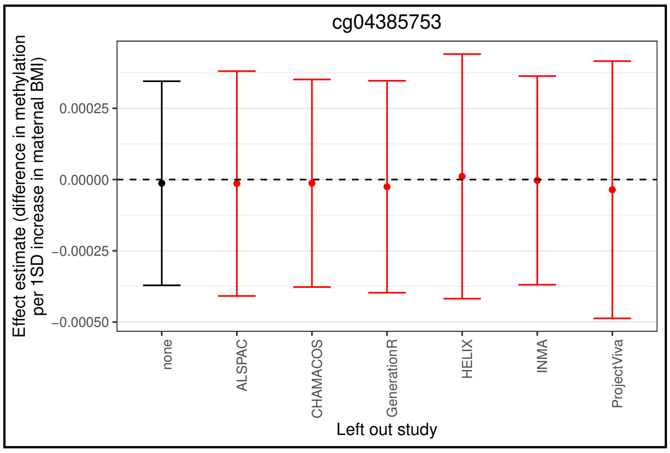
cg04385753
Effect estimate (difference in methylation per 1SD increase in maternal BMI)
0.00025
0.00000
−0.00025
−0.00050
none
ALSPAC
CHAMACOS
GenerationR
HELIX
INMA
ProjectViva
Left out study

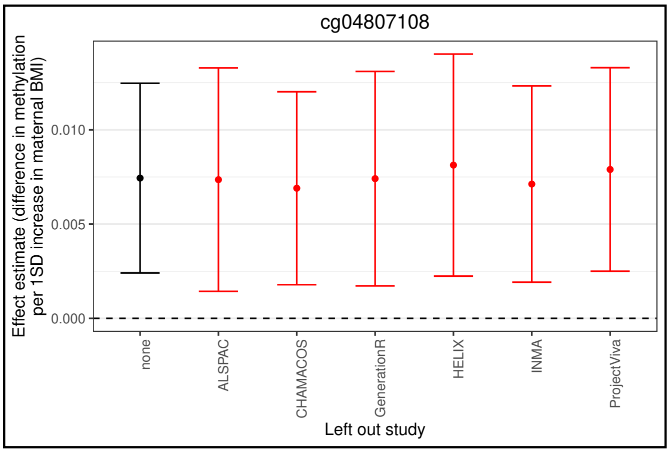
cg04807108
Effect estimate (difference in methylation per 1SD increase in maternal BMI)
0.010
0.005
0.000
none
ALSPAC
CHAMACOS
GenerationR
HELIX
INMA
ProjectViva
Left out study

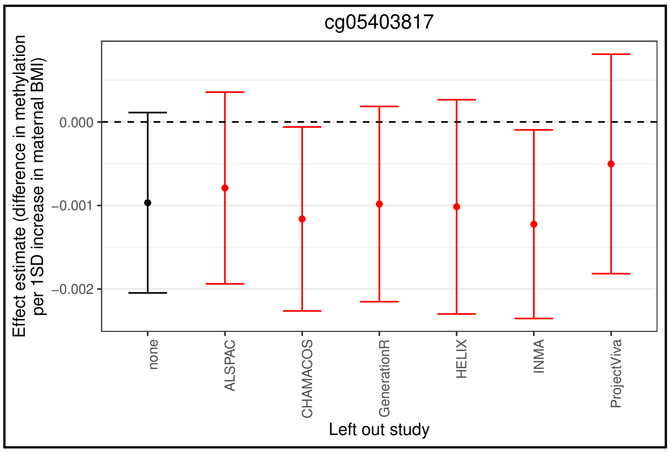
cg05403817
Effect estimate (difference in methylation per 1SD increase in maternal BMI)
0.000
−0.001
−0.002
none
ALSPAC
CHAMACOS
GenerationR
HELIX
INMA
ProjectViva
Left out study

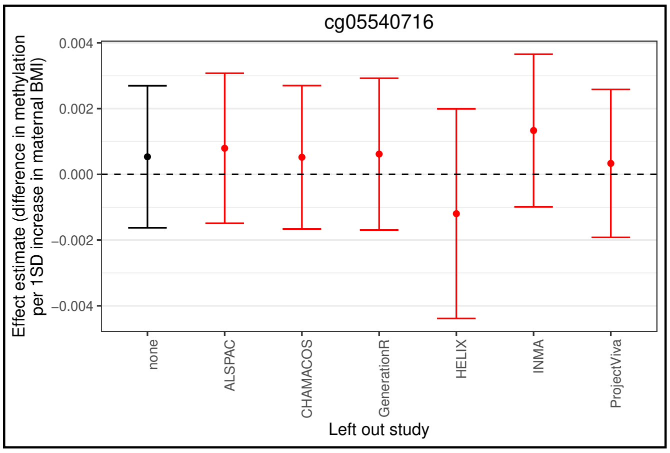
cg05540716
Effect estimate (difference in methylation per 1SD increase in maternal BMI)
0.004
0.002
0.000
−0.002
−0.004
none
ALSPAC
CHAMACOS
GenerationR
HELIX
INMA
ProjectViva
Left out study

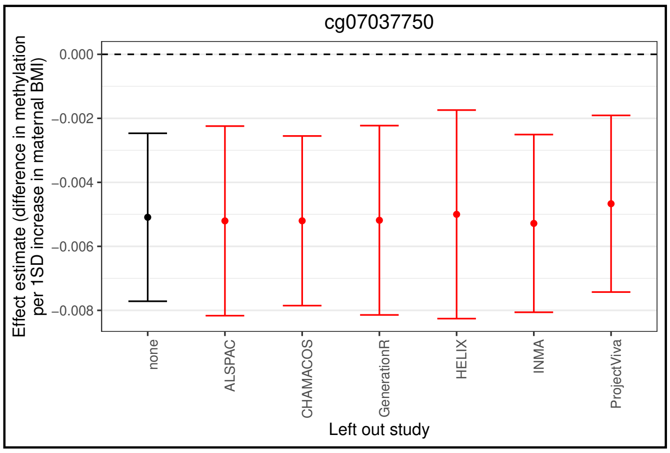
cg07037750
Effect estimate (difference in methylation per 1SD increase in maternal BMI)
0.000
−0.002
−0.004
−0.006
−0.008
none
ALSPAC
CHAMACOS
GenerationR
HELIX
INMA
ProjectViva
Left out study

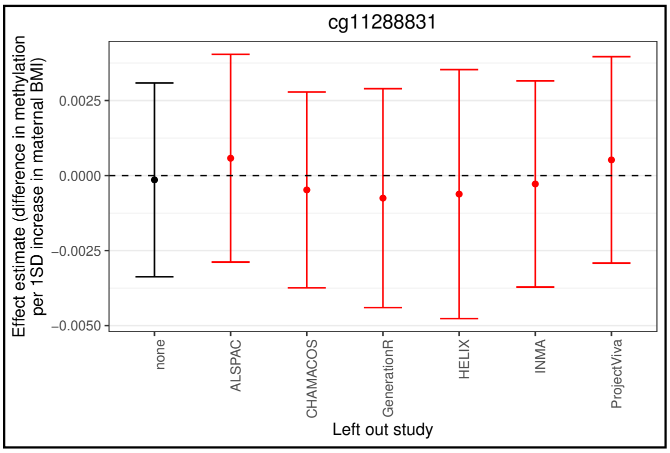
cg11288831
Effect estimate (difference in methylation per 1SD increase in maternal BMI)
0.0025
0.0000
−0.0025
−0.0050
none
ALSPAC
CHAMACOS
GenerationR
HELIX
INMA
ProjectViva
Left out study

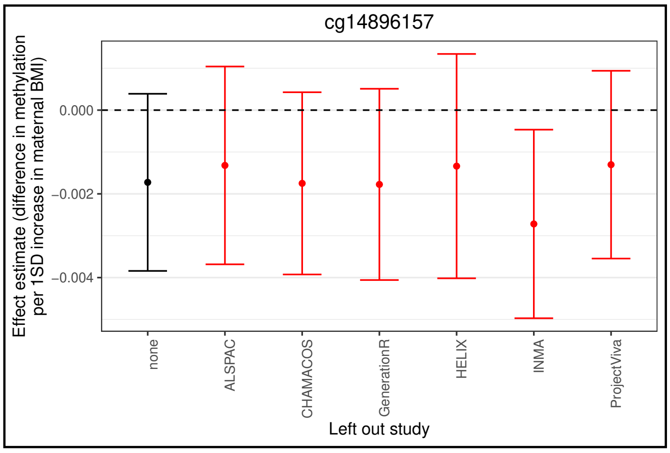
cg14896157
Effect estimate (difference in methylation per 1SD increase in maternal BMI)
0.000
−0.002
−0.004
none
ALSPAC
CHAMACOS
GenerationR
HELIX
INMA
ProjectViva
Left out study

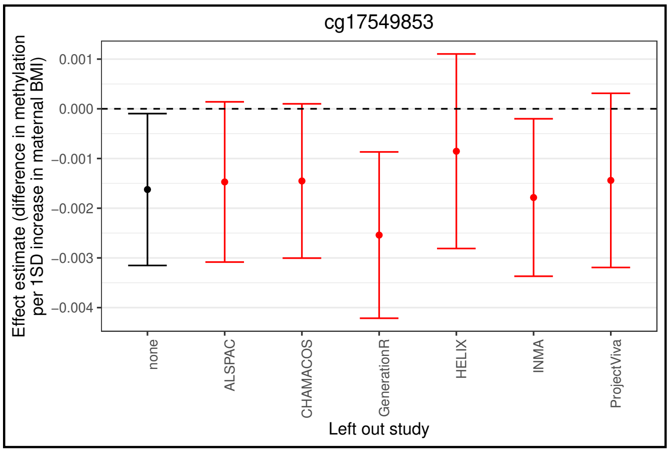
cg17549853
Effect estimate (difference in methylation per 1SD increase in maternal BMI)
0.001
0.000
−0.001
−0.002
−0.003
−0.004
none
ALSPAC
CHAMACOS
GenerationR
HELIX
INMA
ProjectViva
Left out study

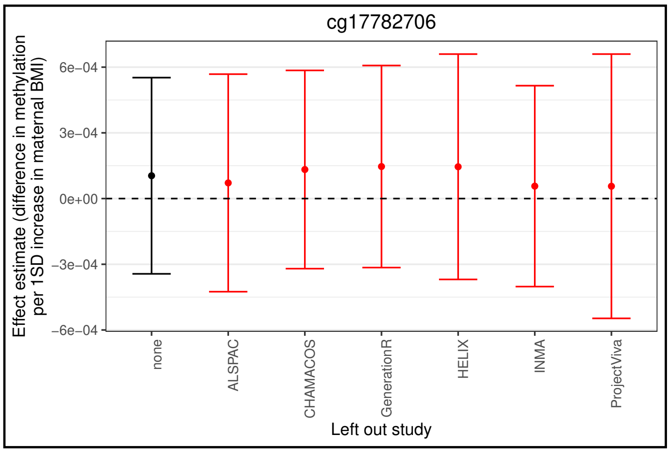
cg17782706
Effect estimate (difference in methylation per 1SD increase in maternal BMI)
6e−04
3e−04
0e+00
−3e−04
−6e−04
none
ALSPAC
CHAMACOS
GenerationR
HELIX
INMA
ProjectViva
Left out study

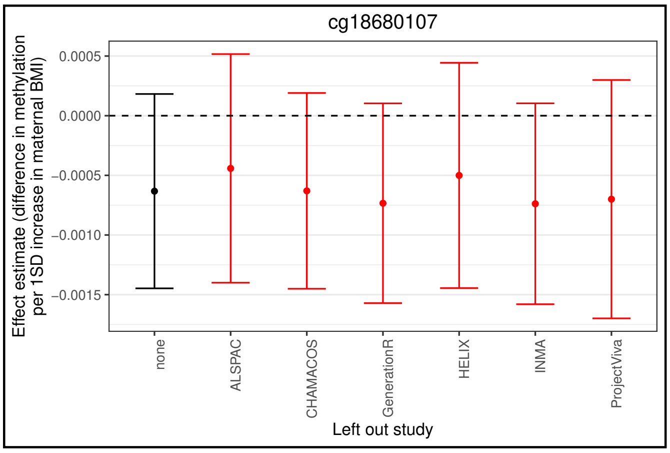
cg18680107
Effect estimate (difference in methylation per 1SD increase in maternal BMI)
0.0005
0.0000
−0.0005
−0.0010
−0.0015
none
ALSPAC
CHAMACOS
GenerationR
HELIX
INMA
ProjectViva
Left out study

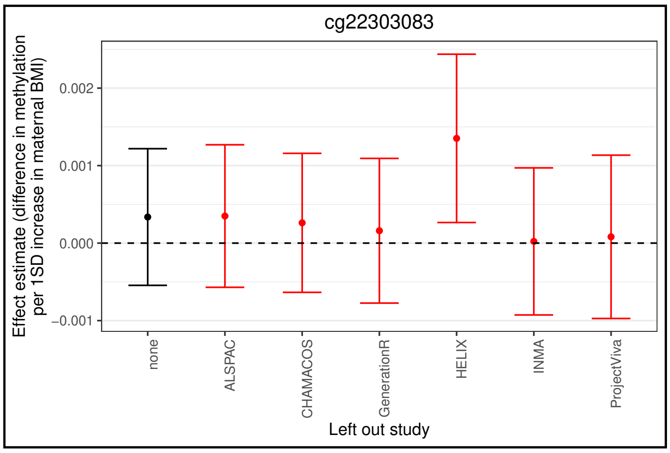
cg22303083
Effect estimate (difference in methylation per 1SD increase in maternal BMI)
0.002
0.001
0.000
−0.001
none
ALSPAC
CHAMACOS
GenerationR
HELIX
INMA
ProjectViva
Left out study

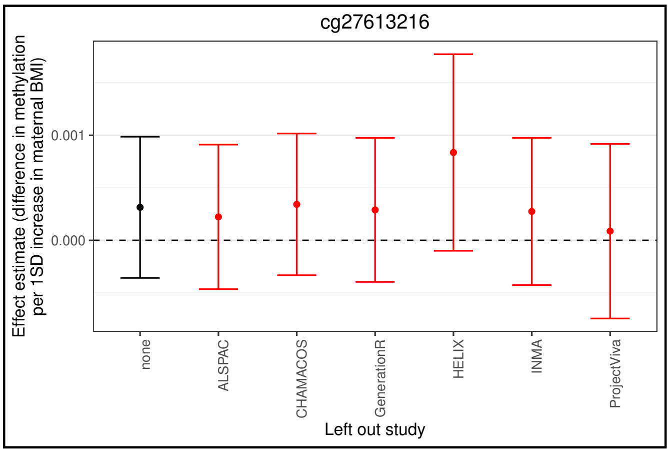
cg27613216
Effect estimate (difference in methylation per 1SD increase in maternal BMI)
0.001
0.000
none
ALSPAC
CHAMACOS
GenerationR
HELIX
INMA
ProjectViva
Left out study

Meta-regression tables: pages 23-25

Paternal BMI (unadjusted for maternal BMI)

CpG	Effect estimate (change in EWAS effect size per year)	Lower limit to CI	Upper limit to CI	P-value
**cg09934789**	0.005	-0.024	0.034	0.728
**cg12082202**	0.012	-0.006	0.030	0.192
**cg12294260**	0.009	-0.033	0.051	0.672
**cg14235768**	-0.036	-0.123	0.051	0.415
**cg24533565**	0.031	-0.001	0.063	0.059
**cg27201301**	0.010	-0.117	0.138	0.875

Paternal BMI (adjusted for maternal BMI)

CpG	Effect estimate (change in EWAS effect size per year)	Lower limit to CI	Upper limit to CI	P-value
**cg12082202**	0.012	-0.006	0.030	0.192
**cg17330838**	0.072	-0.146	0.289	0.519
**cg27201301**	0.010	-0.117	0.138	0.875

Paternal BMI adjusted for maternal BMI – female offspring only

CpG	Effect estimate (change in EWAS effect size per year)	Lower limit to CI	Upper limit to CI	P-value
**cg05718625**	0.016	-0.028	0.060	0.470
**cg06644553**	-0.064	-0.166	0.037	0.215
**cg12082202**	-0.011	-0.042	0.020	0.495
**cg16681820**	0.016	-0.012	0.044	0.253
**cg18473137**	0.008	-0.154	0.170	0.921
**cg19339146**	0.144	-0.046	0.334	0.138
**cg21792150**	0.082	-0.018	0.182	0.107
**cg21886089**	0.097	-0.058	0.253	0.219
**cg27201301**	-0.097	-0.310	0.116	0.371

Paternal BMI adjusted for maternal BMI – male offspring only

CpG	Effect estimate (change in EWAS effect size per year)	Lower limit to CI	Upper limit to CI	P-value
**cg05376601**	-0.029	-0.095	0.037	0.392
**cg07984358**	0.089	-0.091	0.268	0.334
**cg08614638**	0.053	-0.109	0.215	0.520
**cg09861496**	-0.009	-0.051	0.032	0.658
**cg13422275**	-0.010	-0.096	0.076	0.819
**cg14738170**	-0.022	-0.257	0.214	0.858
**cg14779797**	0.026	-0.067	0.119	0.587

Maternal BMI (unadjusted for paternal BMI)

CpG	Effect estimate (change in EWAS effect size per year)	Lower limit to CI	Upper limit to CI	P-value
**cg00673189**	-0.008	-0.051	0.036	0.729
**cg01532392**	0.022	-0.079	0.124	0.665
**cg01596892**	-0.001	-0.029	0.028	0.965
**cg02680669**	0.145	-0.012	0.303	0.071
**cg03046346**	0.004	-0.037	0.044	0.866
**cg04221683**	0.029	-0.109	0.166	0.683
**cg07099084**	-0.009	-0.028	0.009	0.333
**cg10239860**	0.038	-0.031	0.108	0.279
**cg13911707**	-0.082	-0.232	0.068	0.282
**cg14896157**	-0.113	-0.228	0.002	0.055
**cg19007101**	-0.013	-0.062	0.037	0.615
**cg23634624**	-0.016	-0.063	0.031	0.512
**cg23950233**	0.152	-0.058	0.362	0.157

Maternal BMI (adjusted for paternal BMI)

CpG	Effect estimate (change in EWAS effect size per year)	Lower limit to CI	Upper limit to CI	P-value
**cg00804043**	-0.032	-0.032	-0.107	0.043
**cg04221683**	0.029	0.029	-0.109	0.166
**cg05403817**	-0.002	-0.002	-0.064	0.060
**cg07037750**	0.000	0.000	-0.135	0.134
**cg07099084**	-0.009	-0.009	-0.028	0.009
**cg07249684**	-0.072	-0.072	-0.365	0.222
**cg14896157**	-0.113	-0.113	-0.228	0.002
**cg17572155**	0.001	0.001	-0.160	0.162
**cg22041137**	-0.055	-0.055	-0.152	0.042
**cg23950233**	0.152	0.152	-0.058	0.362

Maternal BMI adjusted for paternal BMI – female offspring only

CpG	Effect estimate (change in EWAS effect size per year)	Lower limit to CI	Upper limit to CI	P-value
**cg00565075**	-0.120	-0.385	0.145	0.375
**cg02893482**	0.000	-0.039	0.038	0.988
**cg06496728**	-0.106	-0.206	-0.006	0.038
**cg07059402**	-0.050	-0.593	0.494	0.857
**cg07734106**	-0.099	-0.287	0.089	0.300
**cg07992500**	0.069	-0.353	0.491	0.749
**cg08125959**	0.097	-0.046	0.241	0.184
**cg12486814**	-0.059	-0.234	0.116	0.508
**cg13061976**	0.116	-0.056	0.288	0.185
**cg13335329**	0.085	-0.110	0.281	0.393
**cg15731655**	0.089	-0.004	0.183	0.060
**cg16319578**	-0.049	-0.279	0.181	0.676
**cg21495568**	-0.001	-0.047	0.046	0.982
**cg24050474**	-0.121	-0.860	0.618	0.748
**cg24990212**	-0.022	-0.182	0.139	0.789

Maternal BMI adjusted for paternal BMI – male offspring only

CpG	Effect estimate (change in EWAS effect size per year)	Lower limit to CI	Upper limit to CI	P-value
**cg00147244**	-0.068	-0.211	0.076	0.357
**cg03108419**	0.007	-0.037	0.051	0.745
**cg04385753**	0.000	-0.038	0.038	0.996
**cg04807108**	-0.027	-0.470	0.416	0.906
**cg05403817**	-0.028	-0.122	0.066	0.555
**cg05540716**	0.146	-0.009	0.300	0.064
**cg07037750**	-0.023	-0.228	0.181	0.822
**cg11288831**	-0.012	-0.254	0.231	0.924
**cg14896157**	-0.165	-0.320	-0.009	0.039
**cg17549853**	-0.158	-0.290	-0.025	0.020
**cg17782706**	-0.021	-0.071	0.029	0.409
**cg18680107**	-0.057	-0.140	0.025	0.171
**cg22303083**	-0.072	-0.149	0.006	0.072
**cg27613216**	-0.034	-0.095	0.027	0.272
