# Supplementary material for: Paternal body mass index and offspring DNA methylation: findings from the PACE consortium
Source: Int J Epidemiol. 2021 Jan 29;50(4):1297–315. doi: 10.1093/ije/dyaa267 (PMC8407864; doi:10.1093/ije/dyaa267)

Meta-analysis of paternal BMI in relation to cellular heterogeneity

We meta-analysed cohort specific associations between paternal BMI and offspring estimated cell proportions at birth (page 1-2) and childhood (page 3-4).

Birth


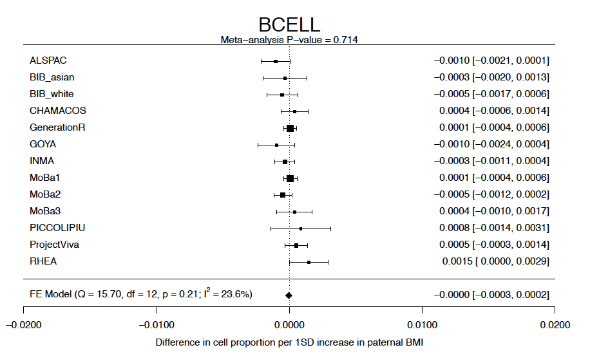


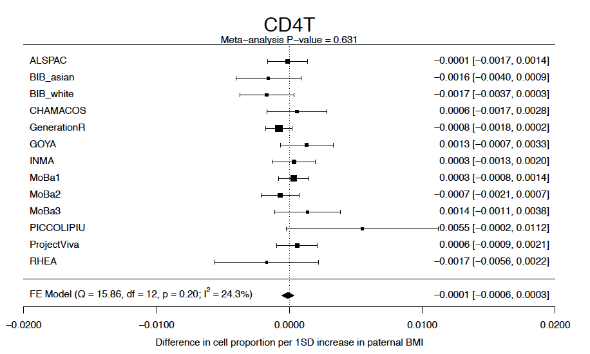


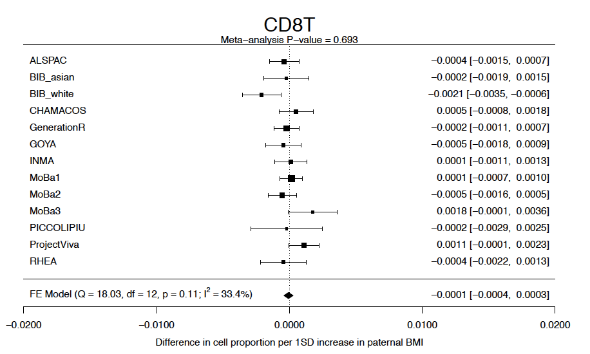


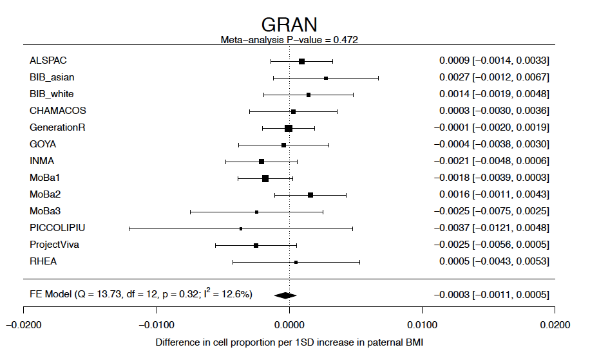


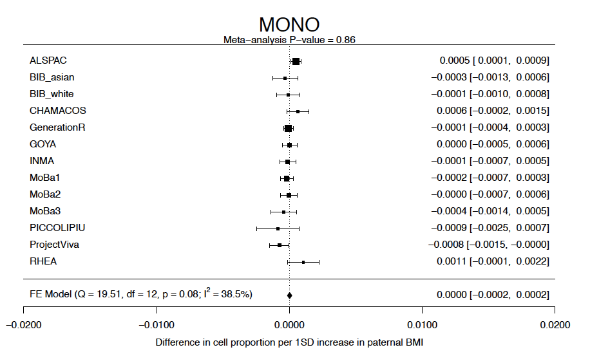


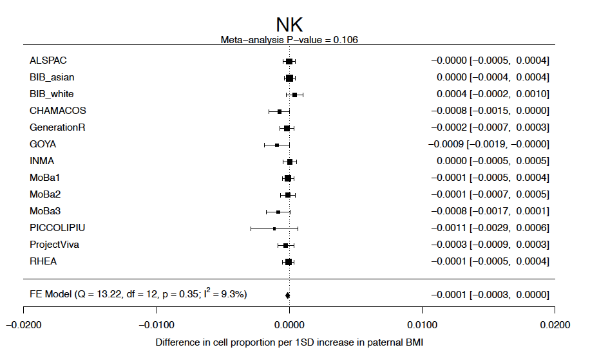


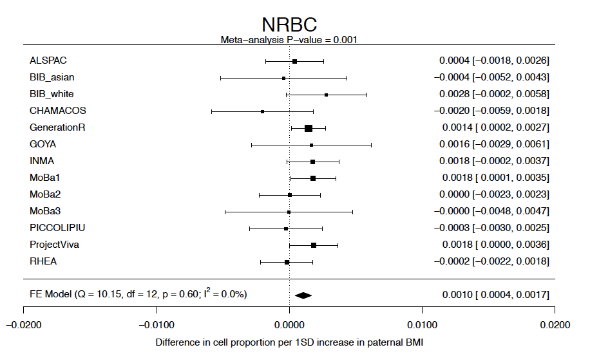


Childhood


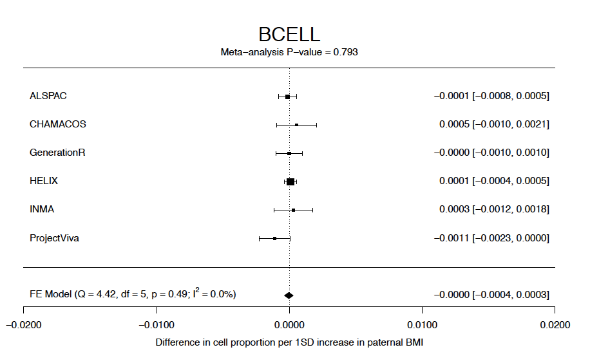


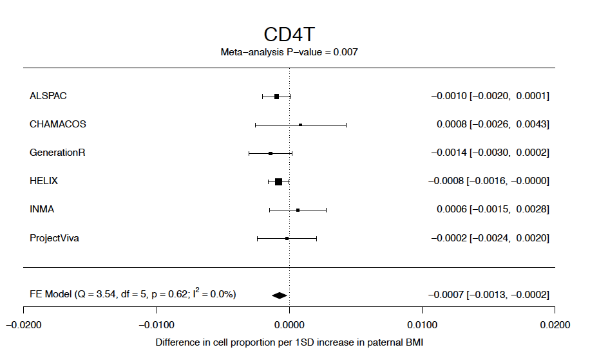


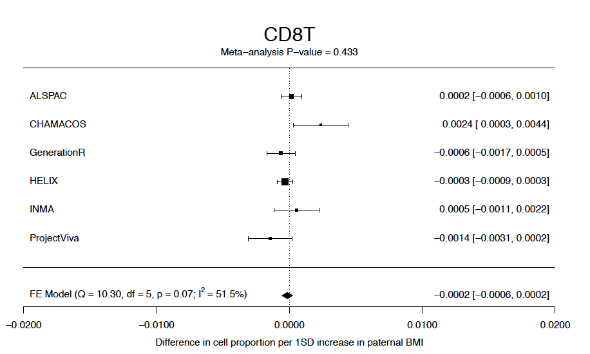


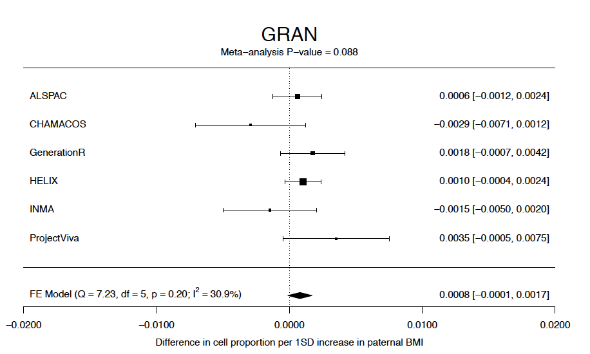


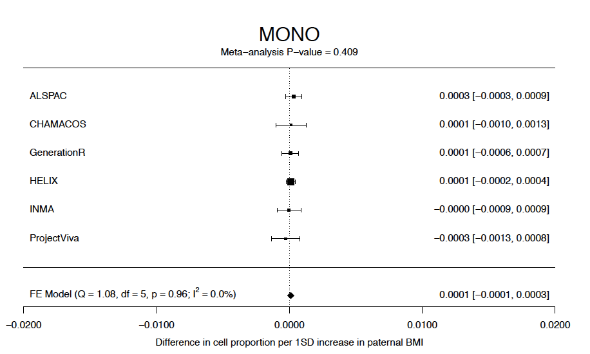


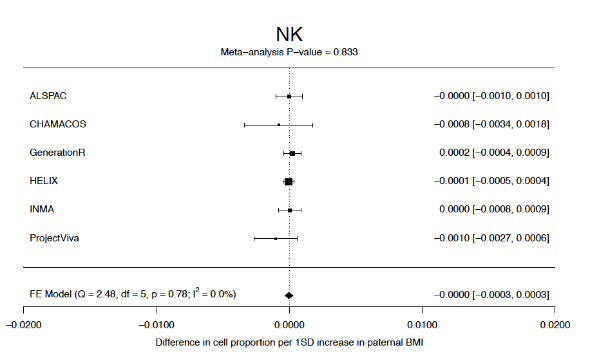

Supplement: dyaa267_Supplementary_Data [file dyaa267_supplementary_data.zip › ije-2020-05-0817-File017.docx]
