# Supplementary material for: Paternal body mass index and offspring DNA methylation: findings from the PACE consortium
Source: Int J Epidemiol. 2021 Jan 29;50(4):1297–315. doi: 10.1093/ije/dyaa267 (PMC8407864; doi:10.1093/ije/dyaa267)
Supplement: dyaa267_Supplementary_Data [file dyaa267_supplementary_data.zip › ije-2020-05-0817-File018.docx]

Further comparison to the literature

In the main text we describe a look-up in our paternal BMI (adjusted for maternal BMI) meta-EWAS results of: 1) sites in imprinted regions, and 2) a paper by Donkin et al. Here, we summarise the results of these look-ups using the paternal BMI *unadjusted for maternal BMI* model.

Imprinted regions:

*Effect estimates for the* *birth meta-EWAS of paternal BMI at CpGs within imprinted regions. The blue ribbon shows the 95% confidence intervals. All results are NOT adjusted for maternal BMI.*

QQ plots showing comparison to results from Donkin et al:


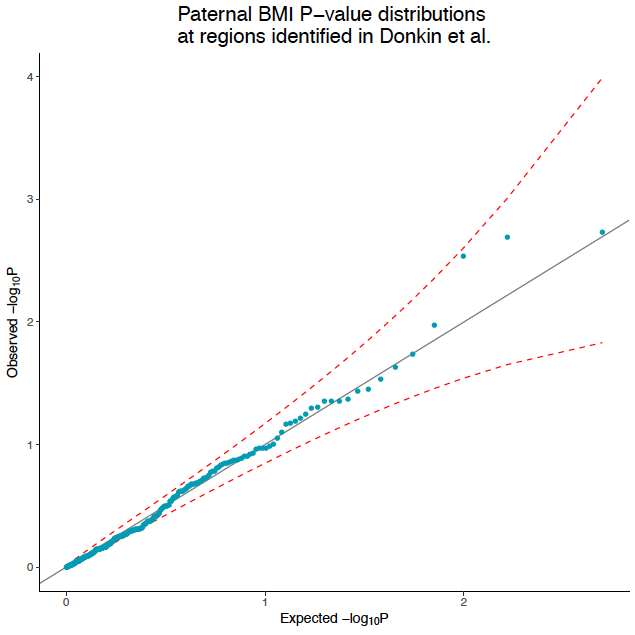


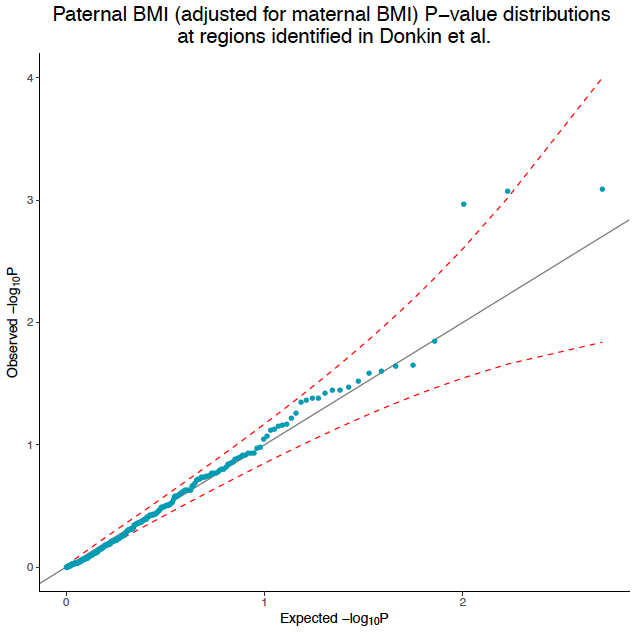


*The observed versus null distributions of P-values from the paternal BMI birth meta-EWAS at CpGs identified as associated with obesity in sperm in a study by Donkin et al. These plots were produced using the paternal BMI meta-EWAS P-values from the models that were adjusted (bottom) and unadjusted (top) for maternal BMI.*
